# Time-resolved fluorescence of tryptophan characterizes membrane perturbation by cyclic lipopeptides

**DOI:** 10.1016/j.bpj.2024.06.022

**Published:** 2024-06-22

**Authors:** Iulia Carabadjac, Jessica Steigenberger, Niels Geudens, Vic De Roo, Penthip Muangkaew, Annemieke Madder, José C. Martins, Heiko Heerklotz

**Affiliations:** 1 Institute of Pharmaceutical Sciences, University of Freiburg, Freiburg, Germany; 2 NMR and Structure Analysis Research Group, Department of Organic and Macromolecular Chemistry, Ghent University, Ghent, Belgium; 3 Organic and Biomimetic Chemistry Research Group, Department of Organic and Macromolecular Chemistry, Ghent University, Ghent, Belgium; 4 Center for Biological Signaling Studies (BIOSS), University of Freiburg, Freiburg, Germany; 5 Leslie Dan Faculty of Pharmacy, University of Toronto, Toronto, ON, Canada

## Abstract

Viscosin is a membrane-permeabilizing, cyclic lipopeptide (CLiP) produced by *Pseudomonas* species. Here, we have studied four synthetic analogs (L1W, V4W, L5W, and L7W), each with one leucine (Leu; L) or valine residue exchanged for tryptophan (Trp; W) by means of time-resolved fluorescence spectroscopy of Trp. To this end, we recorded the average fluorescence lifetime, rotational correlation time and limiting anisotropy, dipolar relaxation time and limiting extent of relaxation, rate constant of acrylamide quenching, effect of H_2_O-D_2_O exchange, and time-resolved half-width of the spectrum in the absence and presence of POPC (1-palmitoyl-2-oleoyl-*sn*-glycero-3-phosphocholine) liposomes. Structure, localization, and hydration of the peptides were described by molecular dynamics simulations. The combination of the parameters provides a good description of the molecular environments of the Trp positions and the behavior of viscosin as a whole. Of particular value for characterizing the impact of viscosin on the membrane is the dipolar relaxation of Trp4 in V4W, which is deeply embedded in the hydrophobic core. The limiting relaxation level represents the membrane perturbation—unlike typical membrane probes—at the site of the perturbant. Fractions of Trp4 relax at different rates; the one not in contact with water upon excitation relaxes via recruitment of a water molecule on the 10-ns timescale. This rate is sensitive to the concerted membrane perturbation by more than one lipopeptide, which appears at high lipopeptide concentration and is assumed a prerequisite for the final formation of a membrane-permeabilizing defect. Trp7 relaxes primarily with respect to neighboring Ser residues. Trp5 flips between a membrane-inserted and surface-exposed orientation.

## Significance

Tryptophan (Trp) is a very advantageous—intrinsic, or at the very least, conservative—label for peptide or protein studies. In particular, time-resolved experiments yield a multitude of observable parameters which, in turn, provide a wealth of information on structural and dynamic properties. At the same time, the low excitation wavelength and complex, multi-exponential fluorescence may render it challenging to handle. Here we demonstrate the diverse effects seen for Trp labels at four positions of a small cyclic lipopeptide and how all these parameters can be combined to provide a consistent picture of its membrane insertion and perturbation. This approach should be generally applicable to studying membrane effects of peptides and proteins.

## Introduction

Time-resolved (TR) fluorescence has been utilized for decades to analyze the biological activity of membrane-active biomolecules and agents. In contrast to steady-state methods, TR measurements are more informative and less prone to artifacts, such as inner filter or light-scattering effects (1). Molecular processes on the timescale of nanoseconds are accessible through TR measurements. Understanding such molecular movements helps elucidate the mechanisms of action of active compounds.

A multitude of assays and methods using TR methods exist. Calcein leakage assays are used to characterize membrane permeabilization by antimicrobial peptides (2,3,4), synthetic alternatives (5,6,7), and (bio)surfactants (8,9,10). Here, the amount of dye trapped in a model membrane such as a large unilamellar vesicle (LUV) is measured in parallel with the amount of released dye. This is possible due to the self-quenching property of calcein, whereby the lifetime of the dye indicates its proximity to another dye molecule. Such assays accurately quantify membrane-damaging activity while offering some insights into mechanisms of action (11). Furthermore, subtler changes in membrane properties can be tracked by direct labeling of the membrane. TR-anisotropy measurements of diphenylhexatriene (DPH) and its derivates spread out over the membrane indicate a general membrane disordering by (bio)surfactants but may miss very local perturbations (12). TR emission spectral shift of Laurdan, Prodan, and other solvent-sensitive dyes was shown to reflect the polarity and mobility of their environment, depending on the localization of the dye in the membrane (13,14,15,16).

The disadvantage of these assays is associated with their highly specialized purpose. For instance, calcein cannot report on membrane order, DPH does not reflect membrane hydration, Laurdan cannot gauge membrane perturbation, and so forth. As such, each dye provides narrowly focused and specific information. Therefore, obtaining a comprehensive picture of membrane-targeting activity requires a multitude of measurements with different samples labeled with different dyes. Additionally, none of these dyes gives any direct insights into the molecular movement of the active compound, instead providing information about the lipid environment. Finally, the observations of the membrane-damaging activity are limited by averaging of the fluorescence signal across all areas of the membrane affected and not affected by the active compound. Since membrane damage is often highly localized, the main signal of unperturbed membrane will dominate the signal. The last problem may be resolved by labeling the active compound directly with a fluorescent dye so that the fluorescent signal originates from the compounds’ vicinity. However, it can be expected that any kind of label attached to a small antimicrobial peptide also introduces a potential artifact into the measurements. The presence of such artifacts has been suggested by techniques other than fluorescence (17,18,19).

The use of tryptophan (Trp) as an intrinsic fluorophore eliminates the need for peptide labeling altogether if the peptide of interest naturally contains a Trp residue. Trp is metabolically “expensive” and as such is a rare amino acid, but it is strongly enriched in membrane or transmembrane proteins (20,21,22). Many studies suggest it plays an important role as a membrane anchor, as the indole ring is mostly found to be positioned near the glycerol backbone in the polar-non-polar interface of a lipid bilayer (23,24,25). The presence of Trp in a membrane-active peptide would provide signal directly and only from the affected regions of the membrane. If Trp is not present by nature, site-directed mutagenesis makes it possible to exchange a hydrophobic amino acid in the peptide sequence for Trp. While this introduces a relatively small interference in comparison to the attachment of a big fluorescent dye, the position of the “Trp label” still may matter for the biological function or the experimental observations. In some membrane-active peptides, introduction of Trp into the sequence increased biological activity and/or selectivity against specific bacterial strains (26,27,28,29,30). These effects were related to the number and position of Trp residues. The position and environment of Trp may matter for the observed fluorescence signal as well as its interpretation. This is why one of the aims of this study is to demonstrate how the position of Trp affects the observations regarding peptide mobility, peptide environment, and permeabilizing activity.

The fluorescence properties of Trp are highly sensitive to its surroundings (31,32,33). In comparison to the techniques introduced above, the spectroscopic properties of Trp allow measurements of TR anisotropy, quenching, and spectral shift with a single dye (i.e., Trp). Such a high density of obtained information makes Trp a very powerful fluorophore. At the same time, the complexity of interwoven observation of the movement of Trp, of the movement and polarity of the peptide, and the mobility and polarity of the environment of Trp all at once also comes with highly challenging analysis. With that, the second aim of this study is to dissect the different contributions to their corresponding factors and produce a clear readout of Trp TR fluorescence.

The peptides studied here are synthetic variants of the naturally occurring cyclic lipopeptide (CLiP) viscosin produced by *Pseudomonas fluorescens* (34), in which one hydrophobic amino acid is exchanged for Trp (further referred to as W-viscosins). CLiPs are amphiphilic, membrane-active compounds, produced by bacteria for host defense, swarming motility, or quorum sensing. Their great diversity in activity and structure makes them interesting lead compounds for antimicrobials (35,36,37,38,39) and biocontrol agents (40,41,42,43). However, their exact mode of action on a molecular level is mostly unclear. Information about the specific position and orientation of CLiPs in the membrane, and thus about possible interaction partners or mechanism of action, is essential.

In summary, we use the TR intrinsic fluorescence of the amino acid Trp introduced into a membrane-active peptide in four different positions. We compare the differences between the four chemically synthesized, single-Trp peptide analogs (W-viscosins) to each other, showing the relevance of the position of the label in the active compound or the membrane. We provide a comprehensive toolbox for the analysis of peptide mobility, localization, and orientation of the peptide in the membrane and changes in the peptide environment and verify them by comparison to molecular dynamics (MD) simulations. We include experiments for the determination of possible artifacts/distortions of the experimental readout depending on the Trp position in the peptide and the membrane. A combination of TR anisotropy, spectral shift, quenching, and other experiments with MD simulations makes it possible to create a full picture of peptide-membrane effects.

## Materials and methods

### Chemicals

The zwitterionic phospholipid 1-palmitoyl-2-oleoyl-*sn*-glycero-3-phosphocholine (POPC) was a kind gift of Lipoid (Ludwigshafen, Germany) and used for liposome preparation. For lipid quantification, hydrogen peroxide solution (30 wt %), H_2_SO_4_, (NH_4_)_6_Mo_7_O_24_·4H_2_O, and Fiske-Subbarow reducer were purchased from Sigma-Aldrich (St. Louis, MO). K_2_HPO_4_ was purchased from VWR (Leuven, Belgium). *N*-Acetyl-L-tryptophan amide (NATA) was purchased from Sigma-Aldrich. Tris-H_2_O and Tris/D_2_O buffer were prepared with EDTA purchased from Sigma-Aldrich, and NaCl, NaOH, HCl, and Tris were purchased from Carl Roth (Karlsruhe, Germany). Deuterium oxide (99.8% D) was purchased from Fisher Scientific (Schwerte, Germany). The solvents CHCl_3_ and ethanol HPLC grade were bought from Carl Roth. Ultrapure water was prepared by the Arium Pro system (Sartorius, Göttingen, Germany).

### Synthesis of the W-viscosins

The four W-viscosins L1W, V4W, L5W, and L7W were synthesized in 2- to 5-mg quantities via a total synthesis approach as described previously for viscosin and other *Pseudomonas* CLiP group members (44,45,46) (for more details see Section S1 of supporting material). The respective names indicate the type and position of the respective amino acid in naturally occurring viscosin which is replaced by tryptophan (Trp) (Table 1). The details of synthesis, purification, and complete structural characterization are collected in supporting material, Section S1. All W-viscosins were quantified by NMR spectroscopy (ERETIC methodology based on PULCON as described in (47)) and solubilized as 20 mM DMSO (Carl Roth) stock solutions.

**Table 1:** Amino acid sequence of the cyclic lipopeptides viscosin, L1W, V4W, L5W, and L7W in one-letter code

CLiP	Amino acid sequence								
	1	2	3	4	5	6	7	8	9
Viscosin	L (L)	E (D)	aT (D)	V (D)	L (L)	S (D)	L (L)	S (D)	I (L)
L1W	**W** (L)	E (D)	aT (D)	V (D)	L (L)	S (D)	L (L)	S (D)	I (L)
V4W	L (L)	E (D)	aT (D)	**W** (D)	L (L)	S (D)	L (L)	S (D)	I (L)
L5W	L (L)	E (D)	aT (D)	V (D)	**W** (L)	S (D)	L (L)	S (D)	I (L)
L7W	L (L)	E (D)	aT (D)	V (D)	L (L)	S (D)	**W** (L)	S (D)	I (L)

The macrocycle involves cyclization via ester formation between the C-terminal carbonyl and the side-chain alcohol of D-aThr. Changes in the sequence related to viscosin are highlighted in bold. L/D conformation of amino acids is indicated in parentheses. The β-OH C_10_ fatty acid, attached to the N-terminus of all viscosins, is not mentioned explicitly.

### Liposome preparation

POPC was dissolved in chloroform for thin lipid film preparation. Chloroform was removed by vacuum centrifugation at 36°C (RVC 2–18 Cdplus; Martin Christ, Osterode am Harz, Germany), and the lipid films were further dried overnight under high vacuum. The air in the vials was replaced by inert argon gas and the lids sealed with parafilm. Lipid films were stored at −20°C.

The lipid films were hydrated with either Tris buffer (110 mM NaCl, 10 mM Tris, 0.5 mM EDTA, pH 7.4 at 25°C) or heavy Tris buffer (110 mM NaCl, 10 mM Tris, 0.5 mM EDTA dissolved in D_2_O instead of water, pH 7.4, measured with a glass electrode with all chambers filled with aqueous (H_2_O) potassium chloride solution at 25°C without further corrections (48)) at room temperature. Four freeze-thaw cycles were then performed using dry ice and a water bath set at 50°C to increase the unilamellarity of vesicles in the lipid dispersion. The vesicle dispersion was then extruded through two stacked 100-nm polycarbonate membranes (Whatman Nuclepore) using a LIPEX Thermobarrel extruder (Evonik Industries, Essen, Germany) at 25 bar to produce large unilamellar liposomes (LUVs).

All LUVs were characterized with respect to their size (*z*-average of the hydrodynamic diameter 100–120 nm), polydispersity index (<0.1), and lipid content before use. The lipid content was determined according to the Bartlett assay (49), and all LUVs were subsequently adjusted to a common lipid content of 6 mM.

### Sample preparation

All samples were prepared according to the “lipid-into-peptide” mixing protocol (8,50,51). Samples for TR fluorescence measurements contained always 6.6 *μ*M of the respective W-viscosin in buffer, with or without the addition of 30, 60, or 90 *μ*M POPC-LUVs. Samples for Trp quenching experiments contained 6.6 *μ*M W-viscosin, 30 *μ*M POPC-LUVs, and increasing amounts of acrylamide (0.1–0.4 mol L^−1^). Sample concentrations were verified by weighing all pipetted volumes with a precision balance. Samples were prepared and measured in 10 × 10-mm quartz cuvettes (high-precision cell; Hellma Analytics, Müllheim, Germany). Before measurement, all samples containing W-viscosin with POPC-LUVs were incubated for 2 h at 400 rpm at 25°C protected from light. All samples were measured at 25°C and stirred during measurement.

NATA samples were prepared by dissolving the desired amount of dry NATA powder in Tris buffer. The solution was then further diluted so that the attenuation of light (absorbance) by passing through the sample was less than 0.1. The degree of dilution was calculated using Lambert-Beer’s law, assuming an extinction coefficient of 5500 L mol^−1^ cm^−1^ for NATA (52) (resulting concentration of 17–20 mM).

### Time-resolved fluorescence measurements

All measurements of TR fluorescence decays except calcein leakage assays were performed using time-correlated single-photon counting (TCSPC) with the high-performance fluorescence lifetime spectrometer FluoTime300 (FT300) by PicoQuant (Berlin, Germany). For the measurements at FT300, the VisUV laser (PicoQuant) was used in 280-nm mode. Instrument settings were controlled, and initial data analysis was performed by the EasyTau 2.2.3293 system software (PicoQuant).

### Time-resolved emission spectra

Detailed information on settings and measurement procedures are collected in Section S2 of supporting material. Peak-normalized time-resolved emission spectra (TRES) for W-viscosins were calculated at distinct time points after excitation (example in Fig. ESI2). For each point after excitation, the spectral width at half-maximum intensity was determined. Subsequently, the spectral center of gravity in wavenumbers, *ν*, of each spectrum was calculated according to Eq. 1 for TRES analysis (time-dependent fluorescence shift (TDFS), Fig. ESI3). The spectral center of gravity is proportional to the average energy emission, and its time-dependent change, *ν*(*t*), characterizes the extent and the timescale of the environment relaxation process taking place (1,53,54). We call *ν*(*t*), therefore, the relaxation level.

(1) $$v\left(t\right)=\frac{\Sigma_{\lambda }I\left(\lambda ,t\right)\times \lambda^{-1}}{\Sigma_{\lambda }I\left(\lambda ,t\right)}\text{.}$$

In the next step, *ν*(*t*) was fitted monoexponentially according to Eq. 2 to determine the dipolar relaxation time, *t*_relax_, and the maximum shift of the spectral center of gravity produced by the fluorophore in the fully relaxed excited state, *ν*_∞_. Because the energy shift ends at the value *ν*_∞_, we call it the limiting relaxation level. *C* is a pre-exponential factor representing the detectable part of Δ*ν* (see below).

(2) $$v\left(t\right)=Ce^{-\frac{t}{t_{\text{relax}}}}+v_{\infty }\text{.}$$

The stronger the shift Δ*ν*, the higher the polarity of the surrounding molecules. This has been shown as a linear proportionality between Δ*ν* and the dielectric measure of the solvent polarity (55). Usually, this time-dependent Stokes shift is quantified by

(3) $$\Delta v=v\left(0\right)-v_{\infty }\text{,}$$

where *ν*(0) represents the spectral center of gravity of the spectrum produced by the initial Frank-Condon state, i.e., the spectrum before any molecular reorientation has yet occurred. In general, *ν*(0) can be estimated by measurements of the absorption and emission spectra in completely non-polar solvents (55,56,57). However, this procedure is not applicable for W-viscosins (for details, consult Section S3 of supporting material). Instead of Δ*ν*, the limiting relaxation level, *ν*_∞_, was used for the analysis. Note that in a heterogeneous system, not the predominant component but the component with the longest lifetime will dominate *ν*_∞_.

### Time-resolved anisotropy

For detailed information on measurement settings, consult Section S4 of supporting material. In brief, the measurements of fluorescence decays were performed at 340 nm with the emission polarizer set to vertical, horizontal, or magic-angle conditions. The resulting decays were reconvoluted with the instrument response function and fitted according to Eq. 4:

(4) $$I\left(\theta ,t\right)=\frac{k\left(\theta \right)}{3}\sum_{i=1}^{3}A_{i}e^{-\frac{t}{\tau_{i}}}\times \left[\left(1+\left(3\cos^{2}\;\theta -1\right)\times \left(Be^{-\frac{t}{\varphi }}+r_{\infty }\right)\right)\right]\text{.}$$

The correlational relaxation time *φ* of the anisotropy decay and the limiting anisotropy *r*_∞_ were set globally. Lifetimes of the fluorophore as determined by TRES or VM curve analysis (i.e., eliminating polarization effects by vertical excitation and magic angle emission polarizers), *τ*_1_–*τ*_3_, were used as input data for the fitting process. *A*_*i*_ describes the amplitude of the fluorescence decay component and *B* the amplitude of the anisotropy decay. k(*θ*) is an experimental matching factor for the polarized fluorescence decay collected at the emission polarizer angle *θ*. The term $\frac{k\left(\theta \right)}{3}$ is defined by Eq. 5, yielding $\frac{k\left(\theta \right)}{3}=1$ for vertical emission polarizer alignment (*θ* = 0°), $\frac{k\left(\theta \right)}{3}=\frac{1}{G}$ for horizontal emission polarizer alignment (*θ* = 90°), and $\frac{k\left(\theta \right)}{3}=\left(\frac{2+G}{3\cdot G}\right)$ for magic-angle alignment (*θ* = 54.7°).

(5) $$\frac{k\left(\theta \right)}{3}=\cos^{2}\;\theta +\frac{1}{G}\sin^{2}\;\theta \text{.}$$

### Time-resolved quenching assay

For detailed information of measurement settings, consult Section S5 of supporting material. For simplicity, we evaluated the decays in two steps, beginning with the very good, empirical fit of each decay by the “reconvolution” of a tri-exponential curve (Eq. 6) with the IRF. The “deconvoluted” decays were then reconstructed and fitted globally using a Stern-Volmer model (Eq. 7).

In more detail, the reconvoluted decays were reconstructed with the parameters *A*_1–3_ and *τ*_1–3_ acquired by Eq. ESI3 according to Eq. 6,

(6) $$F\left(t\right)=A_{1}e^{-\frac{t}{\tau_{1}}}+A_{2}e^{-\frac{t}{\tau_{2}}}+A_{3}e^{-\frac{t}{\tau_{3}}}\text{,}$$

where the fluorescence intensity as a function of time is given by *F*(*t*), *t* is the time, and *A* is the pre-exponential factor of the component with the corresponding lifetime *τ*. The decay in the absence of quencher acrylamide, [Q] = 0, is denoted *F*_0_(*t*) and properly represented by Eq. 6.

The reconstructed decays obtained in the presence of five different acrylamide concentrations [Q] were then fitted globally with Eq. 7,

(7) $$F_{q}\left(t\right)=F_{0}\left(t\right)\times {\left(e^{-tk_{q}}\right)}^{\left[Q\right]}\text{,}$$

keeping *F*_0_(*t*) fixed and adjusting the parameter *k*_q_, which is the quenching rate constant in ns^−1^.

For a highly effective collisional quencher and fully exposed dye in solution, *k*_q_ represents the bimolecular quenching constant of the Stern-Volmer equation (58), then approaching the intrinsic rate constant of a diffusion-controlled reaction. In our case, the local quencher concentration and diffusion coefficient at the site of a given Trp differ from that in the bulk. Hence, Eq. 7 using bulk concentrations [Q] will yield an apparent *k*_q_ value is the lower; the lower is the exposure of the Trp to the quencher (i.e., local [Q]).

The fit procedure was performed in Excel (Microsoft, Redmond, WA) using its built-in generalized reduced gradient non-linear fit algorithm (Solver).

### Membrane permeabilization and kinetics by TCSPC

Peptide-triggered membrane permeabilization was quantified by the changes in the fluorescence lifetime of the self-quenching fluorescent dye calcein encapsulated in LUVs (calcein-LUVs). The theory underlying the calcein leakage assay is explained in detail elsewhere (2,11). In brief, the fluorescence lifetime of calcein depends on its local concentration due to collisional and static self-quenching effects. Therefore, calcein entrapped in liposomes (i.e., high local calcein concentration, shortened fluorescence lifetime) and released calcein (i.e., lower local calcein concentration, longer fluorescence lifetime) can be distinguished from each other and quantified independently. In our case, 0% and 100% membrane permeabilization correspond to fluorescence lifetimes of ≈0.4 ns and ≈4 ns (*c*_calcein_ ≈ 70 mM and *c*_calcein_ ≈ 6 *μ*M), respectively. Encapsulated but increasingly diluted calcein due to leakage events yields lifetimes between 0.4 ns and 4 ns. For a detailed description of settings, measurement procedures, and analysis, consult Section S6 of supporting material.

Subsequently, calcein leakage *L* can be calculated by Eq. 8. $Q_{\text{Stat}}=1.2\pm 0.2$ (11) is an empirical correction factor accounting for amplitude reduction due to some static quenching next to the predominant collisional quenching effects. Not all calcein can be completely removed by size-exclusion chromatography during liposome preparation. This amount of free calcein is determined by a sample without peptide additive and accounted for by *B*_F0_.

(8) $$L=\frac{B_{F}-B_{F0}}{B_{F}-B_{F0}+Q_{\text{Stat}}B_{E}}\text{.}$$

### Membrane partitioning

The membrane distribution behavior of the W-viscosins was determined according to a procedure published in (59) with slight modifications to instrument settings and data analysis. Steady-state emission spectra of W-viscosins and NATA with either 0, 15, 30, 60, or 90 *μ*M POPC-LUVs were recorded with the FT300 (Fig. ESI4). Detailed measurement settings, measurement, and analysis procedures can be found in Section S7 of supporting material. In brief, the change in fluorescence intensity at a given wavelength correlates linearly with the amount of peptide bound to the membrane (59). Subsequently, the intensities corrected for the scattering light effects (*I*′_W_) for W-viscosins at a certain *c*_L_ and six arbitrarily chosen wavelengths (310, 320, 330, 340, 350, and 360 nm) were fitted globally to calculate the membrane partitioning coefficient *K* (*μ*M^−1^) of the W-viscosins by Eq. 9. *I*_W100_ and *I*_W0_ represent the fluorescence intensity upon complete peptide binding (100%) and no peptide binding at all to the lipid membrane (0%), respectively.

(9) $$I_{W}^{'}\left(c_{L}\right)=\frac{Kc_{L}}{1+Kc_{L}}I_{W100}^{'}+\left(1-\frac{Kc_{L}}{1+Kc_{L}}\right)I_{W0}^{'}\text{.}$$

The dissociation constant *K*_d_ (*μ*M) was then calculated as the reciprocal of *K* and used for analysis. High values for *K*_d_ represent a low membrane affinity of the peptide and vice versa. The goodness of fit was assessed by performing a support plane analysis and evaluated by the normalized sum of squared residuals (NSSR) derived from the quotient of individual SSR values and the SSR value of the best fit. The procedure used was described by Kemmer and Keller in 2010 (60). An arbitrary cutoff value of NSSR = 1.5 was chosen. Some NSSR plots showed rather flat slopes around the minimum, which resulted in wide error bars.

### Molecular dynamics simulation of W-viscosins-POPC bilayer interaction

MD simulations of viscosin and W-viscosin analogs L1W, V4W, L5W, and L7W with a POPC membrane were performed and analyzed as follows. The three-dimensional structure of the tryptophan analogs of viscosin were generated by replacement of the original Leu or Val side chain by a Trp side chain, starting from the NMR-derived structure of “wild-type” viscosin (unpublished data). The LIPID14 and ff14SB force fields (61,62) were used for the unrestrained simulations. Structural parameters of the ester bond as well as the fatty acid chain were identical to those used previously (63).

CHARMM-GUI software (64) was used to create the pre-equilibrated POPC lipid system in a rectangular simulation box. The lipid system consisted of 294 lipids (147 per leaflet) at 300 K (temperature above the phase-transition temperature of POPC) at a NaCl concentration of 0.1 M where approximately 40 TIP3P (65) water molecules per lipid were added for system solvation. In the simulations, individual Trp analogs were placed with their molecular center at the level of the phosphatidylcholine headgroups at the water-bilayer interface of the pre-equilibrated and solvated POPC bilayer. The initial orientation was defined using the stable orientation observed for viscosin at the end of a 100-ns MD simulation using otherwise identical starting conditions. The system was neutralized by the addition of Na^+^ ions such that the overall charge of the simulation box is zero. Usage of the SHAKE algorithm kept hydrogen-involving bond lengths constant such that a time step of 2 fs could be used. Langevin dynamics (collision frequency of 1.0 ps^−1^) was used for temperature scaling. The particle-mesh Ewald summation computed electrostatic interactions (cutoff for non-bonded interactions of 9 Å). The equilibration protocol before simulation consisted of an initial minimization step relaxing the initially generated structure (fixation of solute molecules with strong positional restraint at 100 kJ mol^−1^ Å^−2^, only positions of solvent molecules and ions were minimized) followed by a two-step heating process (first: sequential heating of system from 0 to 100 K; second: gradual heating to 300 K) while keeping the total volume constant. Lipids were fixated by applying a mild potential energy constraint of 10 kcal mol^−1^ Å^−2^. Finally, GPU-accelerated MD simulation production runs were carried out using isotropic position scaling (PMEMD) at constant-pressure periodic boundary conditions using anisotropic pressure scaling in the membrane bilayer (*xy*) plane as part of AMBER18 (66). The total simulation time was 100 ns. This timescale was sufficient to avoid bias or trapping of the lipopeptide coordinates toward the starting structure while ensuring convergence of the MD simulations (for more details see supporting material, Section S11 and following). The lack of bias was established by monitoring a number of parameters in a separate set of MD simulations (E) where either 1) viscosin was placed at different starting positions but with the same orientation with respect to the POPC membrane, and 2) a simulation whereby viscosin was placed upside down at the water-bilayer interface position. From these it was found that viscosin adopts highly similar end locations within the POPC bilayer and this well within the initial 50 ns of the simulation, with reorientation requiring less than 100 ns, thus eliminating concerns for potential bias toward starting conditions. Also, extending the simulation time from 100 ns to 500 ns for viscosin did not result in the occurrence of any additional events or significant departures from the behavior witnessed in the shorter simulations, alleviating potential concerns regarding the simulation timescale (Section S12 of supporting material). Trajectories were visualized with VMD 1.9.3 (67) and analyzed with PTRAJ or CPPTRAJ (68) (both included in AMBER software). The “WATERSHELL” command of CPPTRAJ was adapted and used for the quantification of water molecules within the first and second solvation shell (cutoff set to 3.4 Å and 5.0 Å, respectively) of the Trp residues. Moreover, the distance between the Trp and nearby POPC headgroups was analyzed using radial distribution function analysis. Bilayer thickness could be extracted from the electron density plots of the phosphate groups. Likewise, insertion depth of an individual lipopeptide as a whole, or of an individual Trp residue, was done by performing a Gaussian fitting to their respective backbone electron densities for a selection of points along the simulation trajectory. This provides a measure for their distance from the membrane center. In all analyses, a simulation of wild-type viscosin was used as a reference. Relevant information on the MD simulations of the W-viscosins is collected in Section S14 of supporting material.

## Results and discussion

### W-viscosins share similar structure and localization in the membrane

Members of the viscosin group exhibit a shared overall conformation that is maintained in organic solvents and dodecylphosphocholine micelle solutions (63,69,70). This conformation is characterized by a left-handed *α*-helix extending from L-Leu1 to D-Ser6, followed by a loop connecting the C-terminus to the middle of the *α*-helix through an ester (or depsi) bond with the side chain of D-aThr3. This distinctive fold results in a pronounced segregation between hydrophilic and hydrophobic residues, generating an amphipathic surface. Consequently, this structural configuration leads to physicochemical properties pushing the molecule to interact with a membrane surface. The structure, orientation, and insertion depth of the W-viscosins were investigated by MD simulations. All W-viscosins were initially positioned exactly in the same orientation and position, placing its center at the level of the phosphatidyl headgroups and with an orientation in line with its amphipathic character. The duration of the MD simulation was set to 100 ns, giving the peptides sufficient time to adopt their preferred orientation in the POPC membrane bilayer (first 50 ns) before the analysis (last 50 ns).

**Figure 1:**
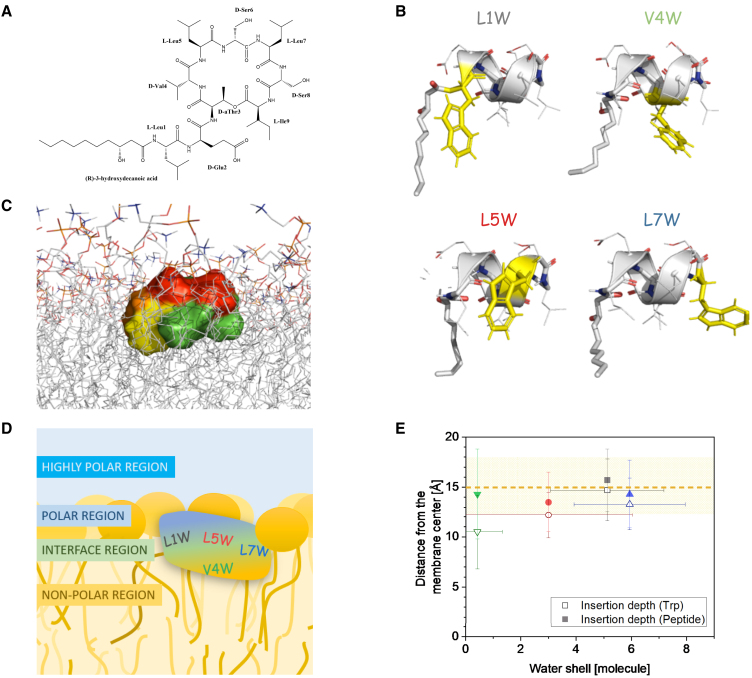
Overview of viscosin and the different W-viscosin analogs and of their proposed orientation in the POPC membrane. (*A*) Chemical structure of viscosin. (*B*) Snapshots of the conformation of the W-viscosins reached during MD simulations in the POPC membrane. Trp is shown in thick yellow stick representation. All W-viscosins share a very similar peptide conformation. (*C*) Snapshot of L1W as embedded in a POPC membrane bilayer. The lipid chains of POPC are colored gray, and the headgroup region orange (phosphate), red (glycerol), and blue (choline). The peptide surface map is colored as follows: green, hydrophobic; red, hydrophilic; orange, fatty acid; yellow, Trp. (*D*) Cartoon showing W-viscosins orientation and position in POPC membrane. The peptide body is marked by a tricolored oval. The color transition from blue to yellow indicates the separation of polar (*blue*) and non-polar (*yellow*) amino acids in this conformation of the peptide. The acyl chain is depicted as a brown line. The position of Trp for four W-viscosin peptides is marked as L1W (*gray*), L5W (*red*), L7W (*blue*), and V4W (*green*). The different regions of the membrane are labeled by the expected polarity of the environment. (*E*) Position of the Trp (*open symbols*) or the whole W-viscosin (*solid symbols*) in the POPC membrane and the corresponding number of water molecules in the solvent shell of Trp (within a 5 Å radius around indole) derived from MD simulations. The standard deviation bars indicate the variation during 50 ns of MD trajectory analysis. Color code for Trp as in (*D*). The dashed orange line indicates the average level of the glycerol moiety of lipids. The yellow shaded area marks the standard deviation of the glycerol position. To see this figure in color, go online.

The POPC bilayer remained planar throughout the simulation, showing no buckling, curving, or other asymmetry manifestations. All W-viscosins maintained the left-handed *α*-helix extending from the first to the sixth amino acid, as observed for viscosin. The W-viscosins also retained the amphipathic conformation in which the hydrophobic residues of the peptide chain are spatially separated from the hydrophilic ones. Consequently, all W-viscosins inserted into the membrane similarly to the wild type, with the hydrophilic residues located between the phospholipid headgroups and the hydrophobic residues positioned between the lipid tails of the lipids, as exemplarily shown in Fig. 1 *C* for L1W.

The localization of the Trp residues follows their attachment to the backbone; the orientation from there is likely affected by the preference of Trp for the hydrophobic-hydrophilic interface (23,24,25). Snapshots of all four W-viscosins are shown in Fig. 1 *B*; they are schematically represented by the cartoon in Fig. 1 *D*. Trp1 and Trp7 are found in the carbonyl region at intermediate polarity (the hydrophobic-hydrophilic interface). Note that Trp1 is located close to a glutamate residue and the lipidic chain attachment site; Trp7 is flanked by two serine residues (Fig. 1 *A*). Trp4 is embedded into the membrane’s acyl chain region, “below” the carbonyl region. Trp5 alternates between two orientations in the simulation, one closer to the aqueous phase (snorkeling “up,” represented in the cartoon Fig. 1 *D*) and one directed more toward the membrane core (reaching “down,” snapshot shown in Fig. 1 *B*).

The cartoon representation in Fig. 1 *B* is confirmed by the primary localization of the Trp residues in the membrane (ordinate) and the average number of water molecules in its hydration shell (abscissa) as derived from the MD simulation (Fig. 1 *E*). Naturally, a deeper insertion correlates with a weaker hydration. Here, the point for L5W represents the average orientation of the Trp during the MD run (whereby the “down” orientation is more populated).

### The differences in TR fluorescence readout of Trp in W-viscosins are not based on structural labeling artifacts

Since the Trp residues in this study are not native but used as low-impact labels with respect to molecular function, it needs to be shown that labeling does not change the molecular properties of the parent peptide viscosin significantly. To evaluate the effects of the exchange of a wild-type residue for Trp in different positions in the sequence, the general features of the W-peptides are compared with each other and their wild-type parent peptide.

The structure and positioning of the W-viscosins within the membrane are essentially conserved upon W-labeling, as demonstrated above via MD simulations.

**Figure 2:**
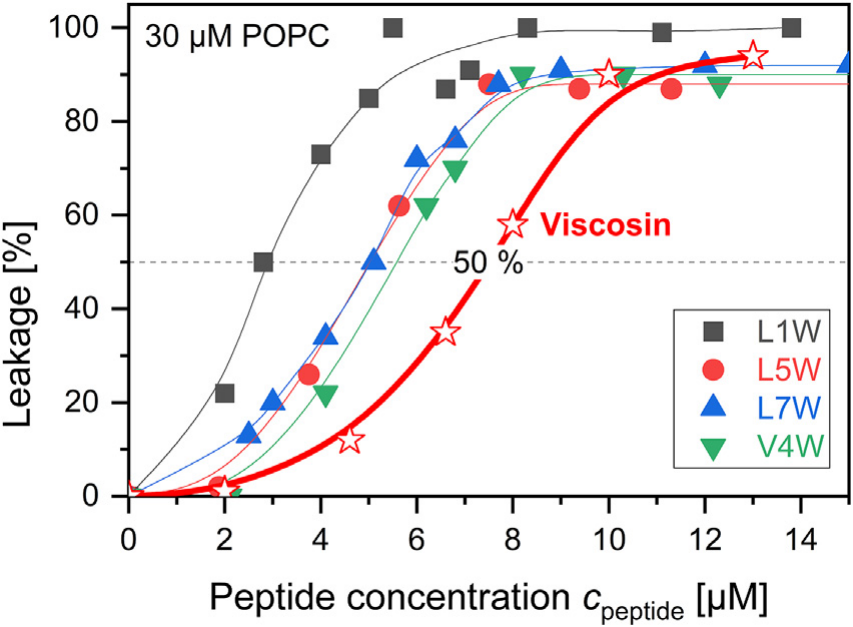
Calcein leakage (%) after 2 h of incubation plotted as a function of increasing W-peptide concentration *c*_peptide_. All experiments were performed with 30 *μ*M POPC-LUVs. Gray squares represent data of L1W, downward green triangles V4W, red circles L5W, upward blue triangles L7W, and red stars wild-type viscosin. Lines were fitted by Hill equation and are to guide the eye only. The dashed line marks the value of 50% leakage. To see this figure in color, go online.

Membrane perturbation activity of W-viscosins and wild-type viscosin was tested by the calcein leakage assay of POPC-LUVs (Fig. 2) as demonstrated for viscosin and other analogs previously (4). All W-viscosins cause increasing leakage of 30 *μ*M POPC-LUVs in the low-micromolar range of 2–10 *μ*M. At a concentration of ≈7 *μ*M W-viscosins, the membrane is disturbed to a level where essentially all initially trapped calcein leaks out of the vesicle within 2 h. The W-peptides are slightly more active than their parent peptide viscosin, which reaches this level of membrane distortion at 12 *μ*M. Note that activity tests (such as for determination of the minimal inhibitory concentration, MIC) are typically done on a log scale with a twofold dilution series—a factor of 2 in half-active concentration means a minor change on biological activity scales.

Similar observations were reported for the MIC of synthetic W-pseudodesmins, where the activity was mostly conserved within a factor 2 of the parent peptide (46). The structure of pseudodesmin A is related to that of the viscosin with small differences in sequence in position 2 (Glu instead of Gln) and chirality in position 5 (D instead of L).

**Figure 3:**
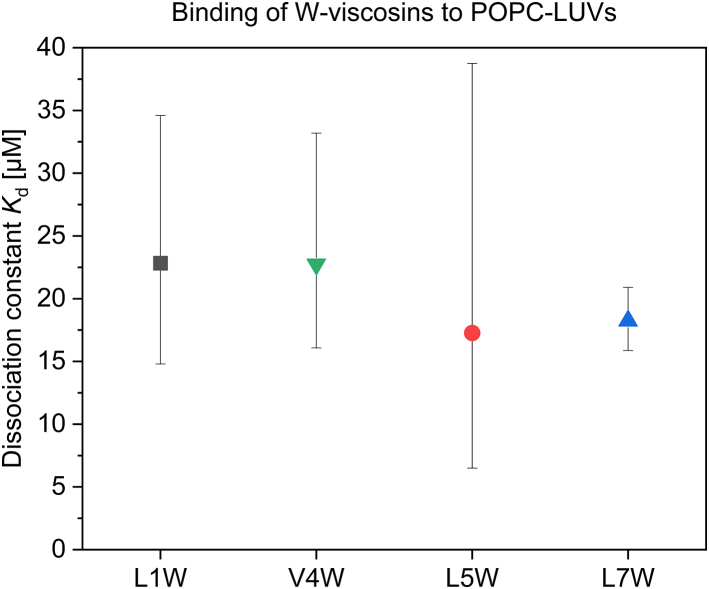
The dissociation constant *K*_d_ of L1W (*gray square*), V4W (*downward green triangle*), L5W (*red circle*), and L7W (*upward blue triangle*) from the POPC membrane is shown. The error bars are derived by support plane analysis (60) and represent an arbitrary cutoff value of normalized sum of square residuals of 1.5. To see this figure in color, go online.

All W-peptides were shown to have an apparent membrane-water partitioning similar to that of a POPC membrane (Fig. 3) as quantified by a dissociation constant *K*_d_ ≈ 20 ± 15 *μ*M. For comparison with standard leakage experiments showing membrane-permeabilizing activity, we have performed the experiments here at 30–90 *μ*M lipid. The *K*_d_ implies that under these conditions, the peptides are predominantly but not completely membrane-inserted.

Additional dynamic light-scattering experiments were performed with samples of about 6.6 *μ*M W-viscosin at 30 *μ*M lipid (Fig. ESI4). The *z*-average (the intensity-weighted mean hydrodynamic size) of the liposomes and the polydispersity index of the samples did not change significantly before and after the addition of the W-viscosins to the liposomes. This suggests that detergent effects such as vesicle disruption, fusion, or solubilization to mixed micelles are not yet triggered to a detectable extent at these concentrations.

In summary, the structure of the W-viscosins in the membrane is not affected by the substitution of the selected residue by Trp. The degree of damage caused to the membrane by the peptides is similar for all W-viscosins, as is their partitioning between membrane and buffer.

### Limiting anisotropy and relaxation level of Trp report on local effects of W-viscosins

**Figure 4:**
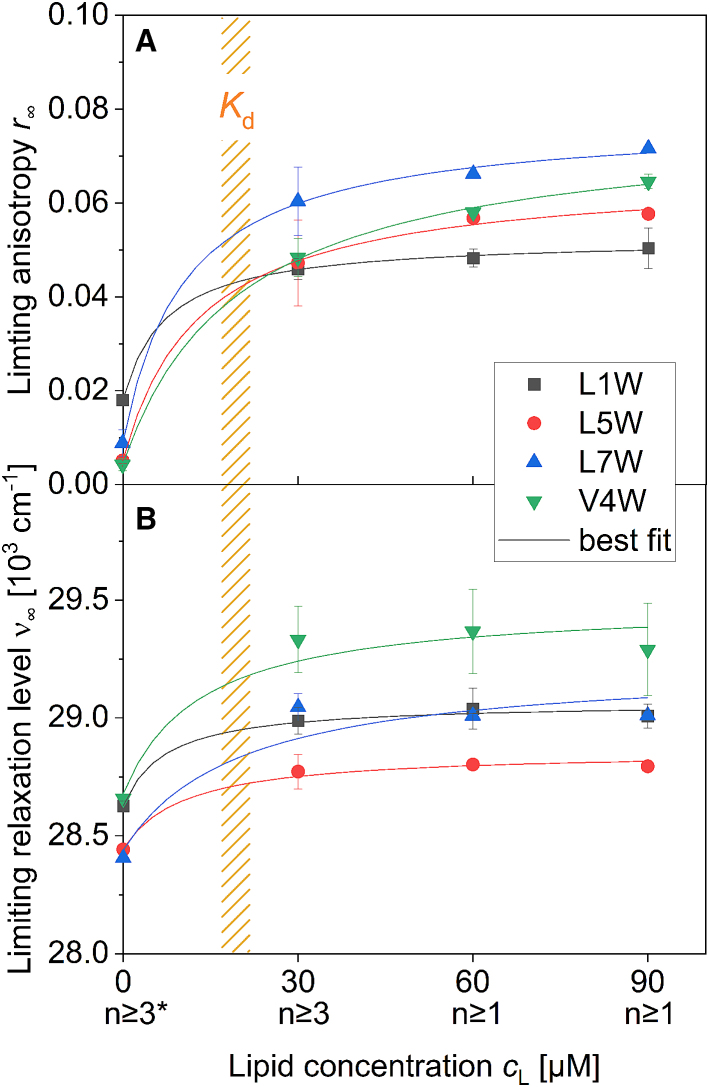
Change in properties of Trp (*A*) and the environment of Trp (*B*) depending on the amount of present POPC-LUVs. (*A*) The limiting anisotropy, *r*_∞_, of W-viscosins and (*B*) the limiting level of relaxation depicted as the spectral center of gravity of time-resolved emission spectra (TRES) in wavenumbers, *ν*_∞_, as a function of lipid concentration, *c*_L_. Number of replicates is displayed under the respective *x*-axis label. Error bars are standard errors of the mean of replicates. The solid lines show the best fit calculated for a W-viscosin population distributed between the buffer and the membrane according to Eqs. 10 and 11. The orange shaded area marks the lipid concentration range at which half of the W-viscosins are bound to the membrane, the dissociation constant *K*_d_. ^∗^The values of the limiting relaxation level at 0 *μ*M lipid are approximate. The monoexponential fit was limited from 0 to 2 ns due to the subsequent rise of *ν*(t) at longer times and does therefore not represent the full extent of the TR fluorescent shift. For more detail read the last section. To see this figure in color, go online.

Membrane perturbation is expected to result in membrane disorder, which may also have an impact on membrane hydration and kinetics. The last two mentioned membrane properties are usually detectable by TR anisotropy (12) and TRES (55). Fig. 4 shows the limiting anisotropy, *r*_∞_, and limiting level of relaxation, *ν*_∞_, as a function of lipid concentration, *c*_L_. The values at zero lipid represent the behavior of W-viscosins in an aqueous solution or dispersion. With increasing lipid concentration, increasing fractions of the W-viscosins are taken up into the membranes and, at the same time, the relative content of peptide (per lipid) in the membranes decreases. Although both *r*_∞_ and *ν*_∞_ are not strictly linear-response functions of partitioning (59), the very similar amplitudes and lifetimes of fluorescence decays from the buffer-exposed and the membrane-bound W-viscosins encouraged us to treat them as such. This means that the parameters as observed (superscript obs) were fitted as a linear combination of characteristic contributions from aqueous (superscript aq) and membrane-bound (superscript m) W-viscosins, weighted by the respective fractions (*Χ*) of the peptide in water and membrane:

(10) $$r_{\infty }^{\text{obs}}\left(c_{L}\right)\approx X_{p}^{m}r_{\infty }^{m}+\left(1-X_{p}^{m}\right)r_{\infty }^{\text{aq}}$$

with

(11) $$X_{p}^{m}=\frac{c_{L}}{K_{d}\left(1+\frac{c_{L}}{K_{d}}\right)}\text{,}$$

where the dissociation constant *K*_d_ is defined as the reciprocal of the mole-ratio partition coefficient (71). For stable fit parameters, *K*_d_ was fixed at the values derived from the steady-state experiment described above. The fit results are presented in Table 2.

**Table 2:** The fit values for the limiting anisotropy in aqueous and lipid environment *r*_∞_^aq^ and *r*_∞_^m^, respectively, and for the limiting relaxation level in buffer, *ν*_∞_^aq^, and the membrane, *ν*_∞_^m^, of the four W-viscosins

	*K*_d_, fixed (*μ*M)	*r*_∞_^aq^	*r*_∞_^m^	*ν*_∞_^aq^ (10^3^ cm^−1^)	*ν*_∞_^m^ (10^3^ cm^−1^)
L1W	23	0.019	0.061	28.64	29.17
L5W	17	0.005	0.070	28.45	28.90
L7W	18	0.010	0.086	28.44	29.21
V4W	23	0.005	0.080	28.70	29.59

The values obtained are as explained for Eqs. 10 and 11.

The fits were performed with the additional implicit assumption that *r*_∞_ and *ν*_∞_ of membrane-bound W-viscosin are approximately constant, i.e., independent of the W-viscosin content in the membrane. The fit curves are in good agreement with the data (Fig. 4), indicating that the underlying assumptions had been warranted to a good approximation.

Note that this finding of largely composition-independent order and relaxation is by no means trivial. Membrane probes such as DPH and di-methylaminonaphthalene derivatives typically show a continuous decrease of *r*_∞_- and *ν*_∞_-like parameters, respectively, upon increasing membrane content of surfactant-like perturbants (72,73). The fundamental difference is that these membrane probes report the overall, average state of the membrane that is not significantly perturbed as long as the perturbant is dilute and the average distance between the perturbant and probe is large. In strong contrast, W-viscosins combine perturbant and reporter probe in one molecule and report local perturbance already in the dilute state.

Hence, the fit values of *r*_∞_^m^ and *ν*_∞_^m^ can directly be interpreted as measures of locally perturbed order and dielectric relaxation at the different label positions. In other words, the approach presented here should permit comparing all these local perturbation parameters of a peptide acting, for example, on membranes of different lipid composition. Such a study (which is well beyond the scope of this paper) could help understanding of the membrane (and hence, organism) selectivity of the antimicrobial activity of a peptide. Understanding the local perturbation (as compared to the global one of the whole membrane) has the advantage that leakage-inducing defect formation, a typical mode of action of membrane-active peptides, is a very local phenomenon that needs only one or a few sites to be effective so that W-viscosin action might not be accompanied by a significant global membrane disordering (12). While the ability to probe local perturbance at the site of the W-viscosins is certainly a valuable step forward, it should be kept in mind that a liposome or bacterial membrane typically contains at least thousands of antimicrobial peptide molecules when, finally, a few of these molecules induce a membrane defect resulting in leakage and biological activity. The local order and relaxation at the very site of a defect may yet differ from the local perturbance at the sites of the vast majority of currently inactive peptides. Unfortunately, the latter will govern virtually all experimental data on the peptide structure, position, orientation, dynamics, and so forth.

A first glance at the individual results shows, as expected, higher mobility and stronger environmental relaxation of the W-viscosins in buffer compared to the membrane-embedded state. Slightly enhanced *r*_∞_^aq^ of Trp1 and *ν*_∞_^aq^ of Trp1 and Trp4 in comparison to other W-viscosins could be explained by a partial intra- or intermolecular coverage of these fluorophores (see below). The relaxation in the membrane-bound state (expressed in terms of *ν*_∞_^m^) of the different W-viscosins will be discussed next.

### Extent of dipolar relaxation is dominated by Trp-water interactions for most but not all Trp positions

The fact that the extent of dipolar relaxation is similar for Trp1 and Trp7 but lower (higher *ν*_∞_^m^) for Trp4 (Fig. 4 *B* and Table 2) is in line with the localization and resulting water exposure of these residues as displayed in Fig. 1 *E* (*open symbols*). The reorientation of water dipoles (bulk or in hydration shell) is well known as a key mechanism of dipolar relaxation. However, the predominant orientation of Trp5 pointing toward the membrane center (“down” orientation) observed in MD and its relatively weak water exposure (Fig. 1 *E*) do not explain the maximal relaxation effect of Trp5 (Fig. 4 *B*). A possible explanation is offered above with the finding that Trp5 has a second characteristic “up” orientation sticking out toward the membrane surface. If this orientation, which appeared less populated in MD, would nevertheless dominate the TR fluorescence result, this would be in accord with the particularly low *ν*_∞_^m^ of L5W.

**Figure 5:**
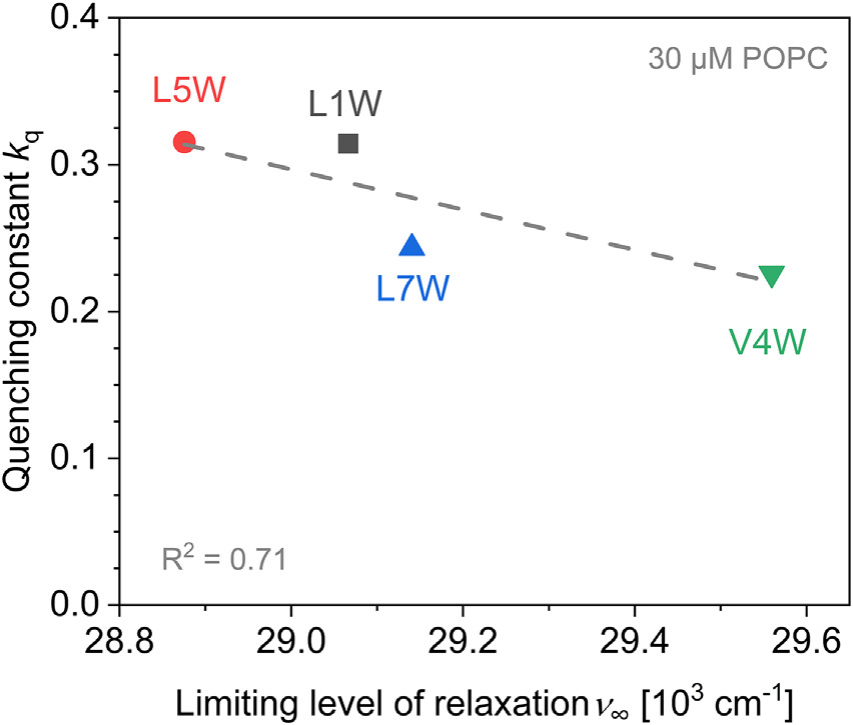
The apparent quenching rate constant in the presence of 30 *μ*M POPC-LUVs, *k*_q_, shown as a function of the fitted limiting relaxation level (center of gravity of TR spectra after infinite time), *ν*_∞_. The gray dashed line represents the linear fit of data points. The quality of the fit is represented by *R*^2^. To see this figure in color, go online.

To test this hypothesis experimentally, we studied the fluorescence quenching of the different Trp positions by acrylamide. This is a potent, highly water-soluble quencher known to be able to report on the depth of Trp incorporation in a membrane (74,75,76). Accessibility to the quencher should be directly related to accessibility to water. The results in Fig. 5 confirm that there is, indeed, a correlation between the apparent rate constant of dynamic quenching, *k*_q_, and *ν*_∞_^m^. The highest exposure of Trp5 among the W-viscosins to quencher and water seems to be in line with an “up” orientation.

It should, however, be emphasized that the correlation between water accessibility and relaxation does not imply that it is actually the reorientation of water that causes the relaxation. This correlation has a strong confounder, the presence of polar groups of peptide or lipid in a given position that both attract water and serve as an alternative mode of dipolar relaxation. An approach to test whether the water itself is responsible for relaxation is based on an H_2_O/D_2_O comparison.

**Figure 6:**
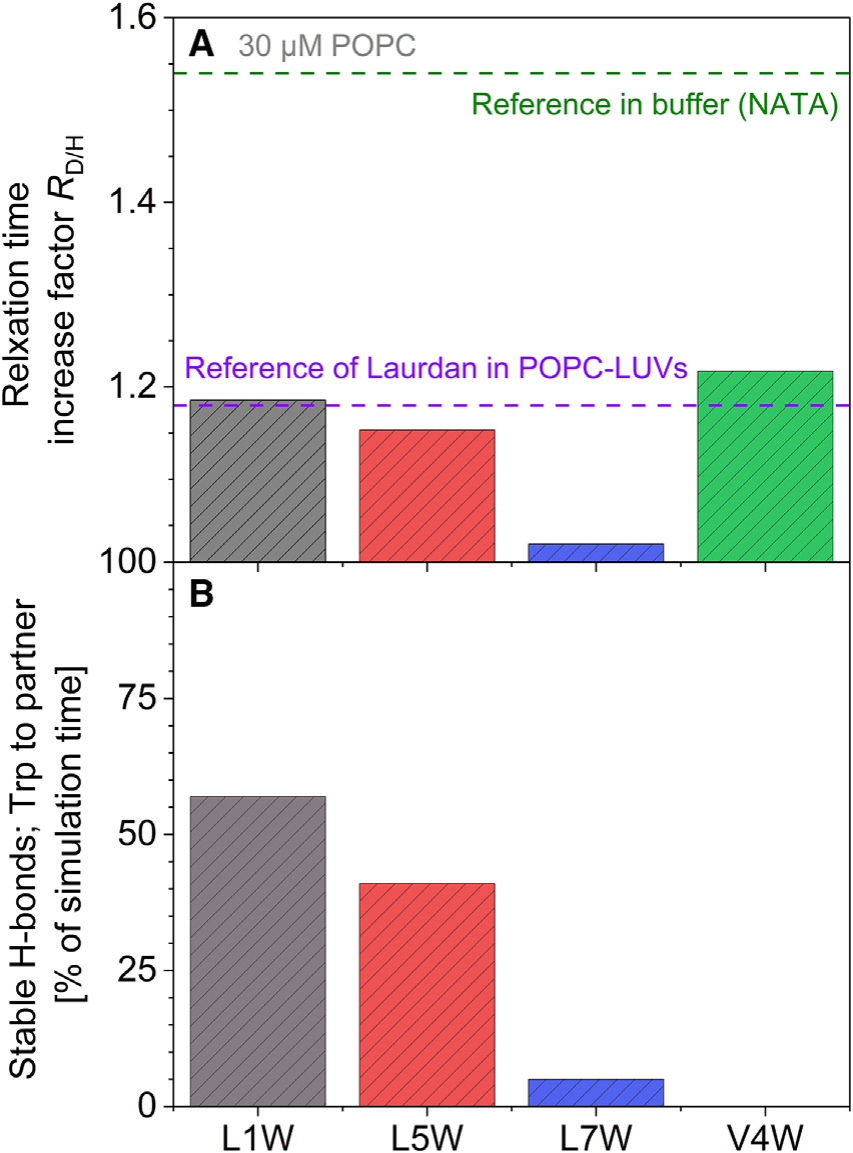
Differences in W-viscosins environment sensed by Trp (*A*) or resulting from MD simulations (*B*). (*A*) The ratio of relaxation time of W-viscosins detected in 30 *μ*M POPC-LUVs prepared with buffer based on heavy water or normal water. L1W data are shown in gray, L5W in red, L7W in blue, and V4W in green. The violet grid line shows the ratio level for literature data of Laurdan in POPC membrane prepared with D_2_O or H_2_O at the Martin Hof group (79) and represents an average effect to be expected for a molecule inserted near the carbonyl group region of the membrane. The green grid line shows the ratio level for water-soluble Trp derivate NATA solubilized in buffer and represents the effect to be expected for pure solvent relaxation. (*B*) Occurrence of stable hydrogen bonds (H-bonds) between the Trp and other molecules as a percentage of the MD simulation time. All observed stable H-bonds are between the nitrogen of the indole of Trp and the oxygens of the carbonyl group of lipids. No stable H-bonds to water molecules occurred. Note the complete absence of H-bonds between V4W and the lipids. To see this figure in color, go online.

Water and heavy water (D_2_O) have nearly identical polarity but were shown to differ in the strength of hydrogen bonds (77). As a consequence, dipolar relaxation is possible to a similar extent in both but slower in heavy water. For virtually fully buffer-exposed Trp in NATA, switching from buffer prepared with normal water to buffer prepared with heavy water increases the relaxation time by a factor of *R*_D/H_ = *t*_rel_(D_2_O)/*t*_rel_(H_2_O) = 1.53 (78). A similarly high *R*_D/H_ is expected whenever relaxation proceeds via bulk-like water reorientation only. For the mostly membrane-inserted W-viscosins in the presence of 30 *μ*M lipid, the heavy-water effect was reduced to about *R*_D/H_ ≈ 1.2 for Trp1, Trp5, and Trp4 as displayed in Fig. 6 *A*. This is in line with the fact that in the membrane-embedded state of a peptide, interactions of Trp with lipids and neighboring amino acids contribute to dipolar relaxation, and the remaining water molecules in the vicinity of the fluorophore are altered with respect to their mobility. Interestingly, a similar *R*_D/H_ of 1.18 has been reported for another probe, Laurdan, embedded primarily in the carbonyl region of POPC membranes (79). This suggests the Trp residues at positions 1, 4, and 5 sense a similar environment and similar dipolar relaxation effects as Laurdan, which is discussed to reflect primarily the mobility of the hydrated lipid carbonyl group (16).

L7W shows a fundamentally different behavior; its relaxation is virtually unaffected by switching to heavy water. In this case, relaxation does not seem to arise from water to a significant extent. Notably, Trp in L7W is flanked by two serines—polar residues likely to reorient according to the dipole of the excited state of Trp. Conversely, the proximity of glutamic acid to the Trp in L1W seems not to affect the relaxation time ratio in the same way. The significance of the slightly higher *R*_D/H_ of Trp4 is not established, but it may still be instructive to discuss this value. Trp4 shows the weakest extent of relaxation, since it is embedded in the acyl chain region of the membrane. A higher *R*_D/H_ may mean that small amounts of water molecules interacting with the *π*-electron system of Trp may, although scarce, be still more active in relaxation than polar groups of the lipid that seem outside the reach of Trp4.

Lipid-Trp hydrogen bonds were quantified in the MD simulation (Fig. 6 *B*). The occurrence of hydrogen bonds (H-bonds) is shown as a percentage of the time they were present during the last 50 ns of the simulation (Fig. 6 *B*). Closer inspection reveals that all observed H-bonds occur between the nitrogen of the indole of Trp and the oxygens of the carbonyl group of the lipid molecules. No continuous H-bonds to water molecules could be detected. In line with the speculation in the previous paragraph, Trp4 is unable to interact with the lipid carbonyls. Trp7, which was argued to relax without substantial involvement of water according to *R*_D/H_ ≈ 1, also does not seem to interact much with the lipid carbonyls. This supports the idea that in contrast to the other W-viscosins and despite the large hydration shell (see Fig. 1 *E*), the relaxation of Trp7 is governed by intramolecular interactions with Ser6 and Ser8.

### MD simulations and TR fluorescence complement each other

Although the interpretation of MD simulations and TR fluorescence results leads to similar findings, some crucial differences between both methods have to be kept in mind.

Further to the different limitations and error sources of the two approaches, a main issue arises from the coexistence of two or more states of a molecule or moiety. Here, examples for such cases would be the partitioning of W-viscosins between buffer and membrane, equilibria of W-viscosin self-association, or the coexistence between two orientations of the Trp residue of L5W resulting in a more superficial or more membrane-embedded localization.

For MD simulations, it is always a challenge to properly represent the equilibrium between such alternative states given the limited number of molecules and time represented by a simulation. Fluorescence parameters obtained in a cuvette should typically involve good statistics but may be differently sensitive to signals from the different states. Steady-state data represent different states weighted according to their quantum yields, which for Trp and many other fluorophores are reduced with increasing exposure to water. Amplitude-averaged parameters of TR measurements eliminate the effects of different lifetimes but would still be affected by static quenching phenomena. Finally, fluorescence parameters reflect the properties of the excited state of the fluorophore. As excitation goes along with a significant change of the dipole moment, the optimal localization of an excited-state Trp should differ from that of that in the ground state. Depending on the timescales of Trp relocalization and fluorescence, this may also affect the experimental result.

The latter phenomenon may explain why the preferred position of Trp of L5W seems to differ between the two methods. According to TR fluorescence, Trp5 is mostly exposed to the membrane surface. In the MD simulation, Trp5 reoriented between two characteristic insertion depths (Table 3), favoring the membrane-embedded “down” orientation. One general way to explain a stronger representation of the “up” orientation in TR fluorescence would be a static quenching process that reduces the fluorescence amplitude of the “down” Trp. In comparison, a reduction in lifetime from a dynamic quenching process would affect only steady-state fluorescence data. Another potentially relevant phenomenon would be the directed reorientation of Trp5 after excitation. Excitation leads to a higher dipole moment that might render the more polar environment in the surface-oriented “up” localization more favorable than in the ground state. During the simulation time, it took the Trp approximately 1 ns to overcome a distance of 1 Å. If proceeding at a similar speed, excitation-induced relocalization favoring the superficial state may become visible as an additional relaxation mechanism and reduce *ν*_∞_ and, perhaps, *r*_∞_.

**Table 3:** Distance from the membrane center of the W-viscosins and their Trp, determined by fitting a Gaussian function to the backbone electron density in the last 50 ns of MD simulation

	Distance from the membrane center (Å)	
	Peptide	Trp
Viscosin	15.2 ± 3.4	–
L1W	15.7 ± 3.1	14.7 ± 3.1
V4W	14.3 ± 4.5	10.5 ± 3.7
L5W^a^	15.2 ± 2.8	14.0 ± 1.3
	11.8 ± 3.2	10.4 ± 3.2
L7W	14.3 ± 3.4	13.3 ± 2.6

a Peptide shows occurrences where it inserts deeper into the membrane.

### TR spectral width reveals heterogeneous relaxation behavior of Trp4 but not Trp5

Textbooks (80) describe two principal modes of relaxation, continuous versus two-state. A continuous shift of the spectrum to longer wavelengths with time is, for example, expected for a dye in a homogeneous solvent where after a given time, the environment of all excited dye molecules has relaxed to the same extent. Such a shift is indicated by a largely constant spectral width. A two-state relaxation may cause the decrease of an emission peak at the expense of a new one appearing at a higher wavelength. Even if the two peaks are not visibly separated, the coexistence of the two spectra at intermediate time will render the overall spectrum broader. Such a transient broadening will also be seen if two fractions of a dye, i.e., in water and the membrane, relax at different rates. At intermediate times, the fractions emit at different wavelengths, and the spectrum is broadened. As the faster-relaxing fraction becomes relaxed, the spectrum narrows again.

**Figure 7:**
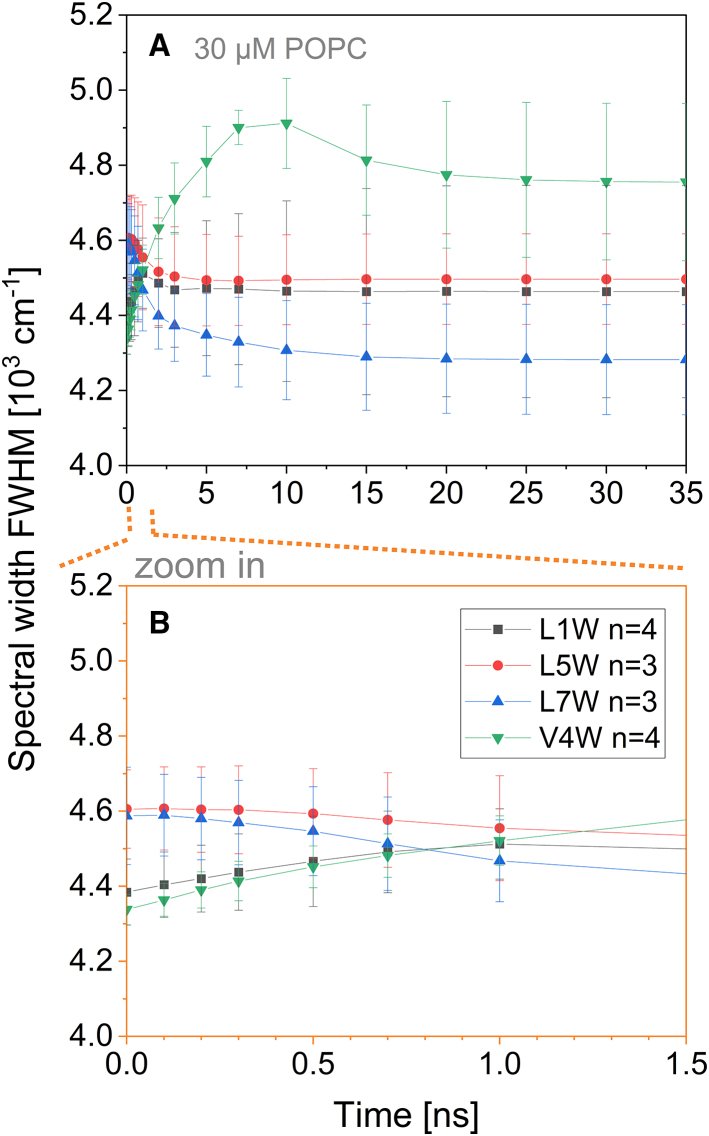
(*A*) The spectral width at half-maximum intensity (FWHM) of W-viscosins in 30 *μ*M POPC-LUVs as a function of time after excitation. (*B*) A zoomed-in section of (*A*) from 0 to 1.5 ns. Gray squares indicate data for L1W, downward green triangles V4W, red circles L5W, and upward blue triangles L7W. Symbols represent an average of actual data points. *n* represents the number of replicates and the error bars the standard deviation. Lines are to guide the eye only. To see this figure in color, go online.

The changes in the width of the spectra throughout the relaxation process (TR full width at half maximum, TR-FWHM) were shown to be indicative of the level of heterogeneity of the environment of the fluorophore (13,78). Fig. 7 shows such profiles for the W-viscosins.

Literature values of spectral width for Laurdan in POPC membrane and Trp in various media are used for reference. For Laurdan, reported TR-FWHMs show a transient maximum at around 1 ns and a spectral width change of ≈2 10^3^ cm^−1^ (13). The transient maximum of Trp in a heterogeneous environment of diethylether and water was shown to be somewhat similar (i.e., transient maximum at ≈1 ns; spectral width change of ≈2 10^3^ cm^−1^), but much broader (FWHM of the transient peak ≈6 ns for Trp in comparison to 1–2 ns for Laurdan) (78). The highest change of spectral width reported in this study is relatively small, <0.5 10^3^ cm^−1^.

TR-FWHMs of L1W and L5W already reach a constant value after the first 1–2 ns. The small changes in spectral width over time are in reasonable agreement with the continuous model of solvent relaxation (80), where the spectra do not change their shape considerably (<0.1 10^3^ cm^−1^) during the shift.

A very prominent transient maximum of the FWHM has been observed for V4W after 7 ns. Seven nanoseconds is much slower than the usual solvent relaxation reported for hydrated POPC membranes. In other words, there are two or more fractions of Trp4 sensing relaxation at different rates, at least one extremely slowly. This observation harmonizes previous, partially confusing pieces of evidence to suggest a consistent scenario. To recall, we had stated that the environment of Trp4 relaxes to a small extent (high *ν*_∞_^m^ compared to other W-viscosins) and largely by water reorientation (high *R*_D/H_, no H-bonds to lipid). However, during the MD simulation, the number of water molecules in contact with Trp4 was reported to be ≈0.4, which translates to one water molecule being present in the Trp environment for 40% of the simulation time. In the fluorescence experiments, where many V4W peptides are observed simultaneously, the Trp4s can be divided into two distinct fractions: Trps that are and Trps that are not in contact with a water molecule at the time of excitation. This is exactly what is required for the FWHM maximum. The water-interacting fraction of Trp4 may sense relaxation at a reasonable rate, even if the extent is rather weak. The water-free environment of Trp4 cannot relax until a water molecule comes into contact with Trp—a process that will proceed via diffusion and could be promoted by the fact that the excited-state Trp attracts water due to its increased polarity. This diffusive or active recruitment of relaxing water then accounts for the component that needs to relax in order to narrow the spectrum down again.

The spectrum of L7W does not show a transient maximum of FWHM but becomes narrower with time by about 0.3 10^3^ cm^−1^. A possible explanation for this type of TR-FWHM is a starting state involving a Trp population with a broad distribution of degrees of relaxation. With time, the unrelaxed “flank” of the spectrum vanishes as the environments of these Trps also relax. This seems in line with the hypothesis of Trp7 relaxation to occur intramolecularly with respect to the flanking Ser residues. In contrast to a dye in a free solvent, the mutual orientations of the adjacent side chains of a compact cyclic peptide are restricted in orientation and motion (note high values for *r*_∞_, Fig. 4 *A*). A fraction with an “excited-state relaxed” mutual orientation of Ser-Trp-Ser may exist in the ground state despite non-optimal dipole-dipole interactions simply because of steric reasons.

### “Relaxation by water recruitment” model explains the peculiar sensitivity of only Trp4 for collective membrane perturbation effects of W-viscosins

**Figure 8:**
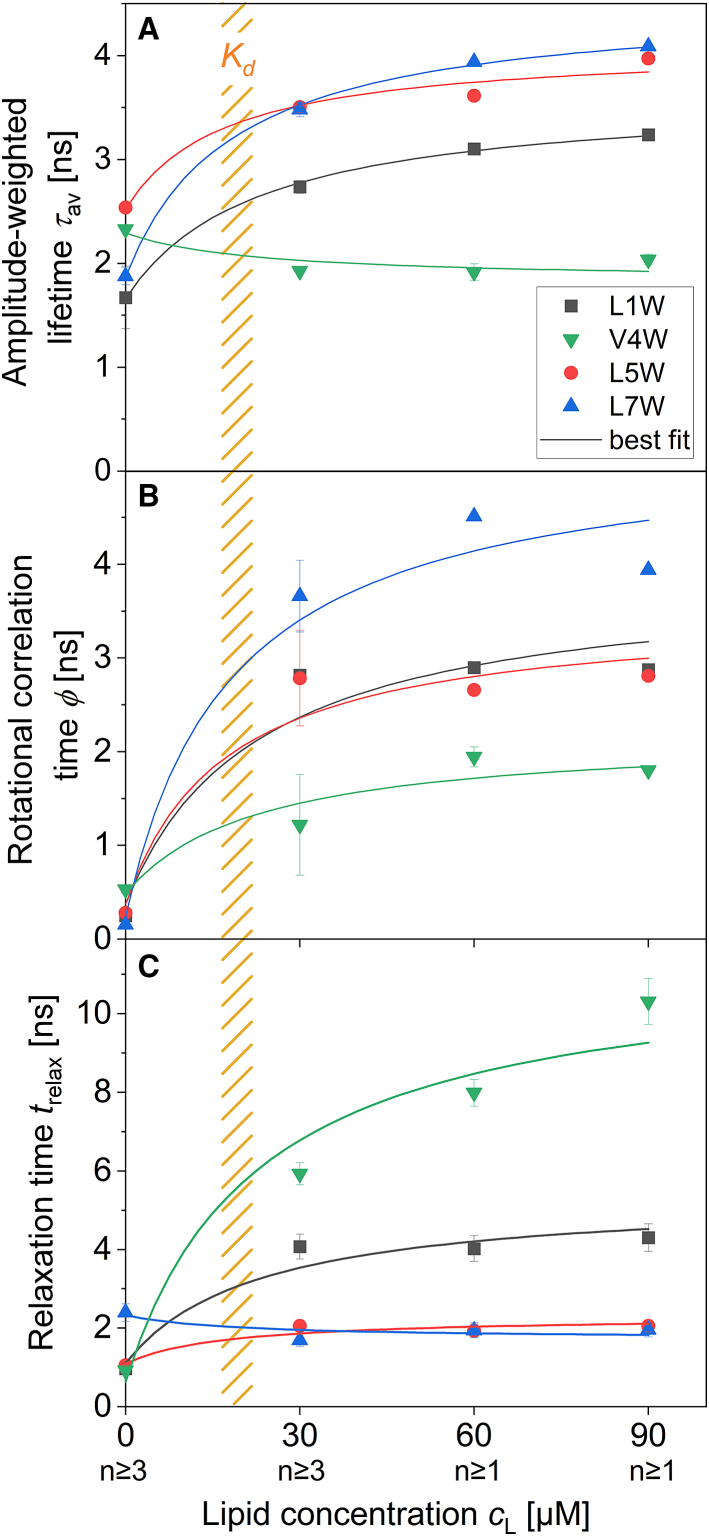
The dynamics of Trp fluorescence (*A*: amplitude-weighted lifetime, *τ*_av_; *B*: rotational correlation time, *φ*; *C*: relaxation time, *τ*_relax_) as a function of lipid concentration, *c*_L_. The number of replicates is displayed under the respective *x*-axis label. The solid lines show the best fit calculated for a W-viscosin population distributed between the buffer and the membrane according to Eqs. 10 and 11. The orange shaded area marks the lipid concentration range at which half of the W-viscosins are bound to the membrane, the dissociation constant *K*_d_. Error bars are standard errors of the mean of replicates. To see this figure in color, go online.

Fig. 8 collects the kinetic parameters representing the speed of motions and relaxation in the membrane, the rotational correlation time *φ*, the relaxation time *t*_relax,_ and the amplitude-averaged fluorescence lifetime, *τ*_av_, as a function of lipid concentration *c*_L_. The figure practically complements Fig. 4, which presented the extent of these motions as specified by *r*_∞_ and *ν*_∞_.

First, Trp4 shows the fastest angular motion but the slowest relaxation of all W-viscosins studied. This can be explained by the “relaxation by water recruitment” model proposed above: on the one hand, the weak (essentially van der Waals only) interactions allow for a fast movement of Trp4 in its rather disordered, hydrophobic environment in the acyl chain region of the membrane. On the other hand, the slow component of relaxation does not represent the mobility of the Trp or that of the lipid chains. Instead, it is thought to describe the speed of water recruitment within or to this hydrophobic region. Such slow dynamics of water molecules in the hydrophobic region of the membrane were previously detected with other fluorophores (81).

Second, the *t*_relax_ of V4W cannot be explained solely by partitioning of V4W into the membrane. The data points with corresponding error bars in Fig. 8 *C* are not overlapping with the fit describing the theoretical *t*_relax_ resulting alone from averaging the parameter over the mix of V4W in buffer and membrane. This is the one and only parameter compiled in Figs. 4 and 8 that responds sensitively to the W-viscosin content within the membrane. Relaxation speeds up from a rate of 1/*t*_relax_ = 0.10 ns^−1^ to 0.17 ns^−1^ as the local peptide-to-lipid mole ratio within the membrane, *R*_b_ (estimated assuming *K*_d_ ≈ 20 *μ*M, see above) increases from 0.06 (at 90 *μ*M lipid) to leakage-inducing 0.13 (at 30 *μ*M). In other words, *t*_relax_ of V4W shows a significant concerted action of two or more W-viscosin molecules to perturb the membrane as it approaches leakage. This perturbation is, apparently, accompanied by an enhanced penetration and/or mobility of water into the hydrophobic core of the membrane. This is very plausibly a process needed for opening a leak in the membrane.

**Figure 9:**
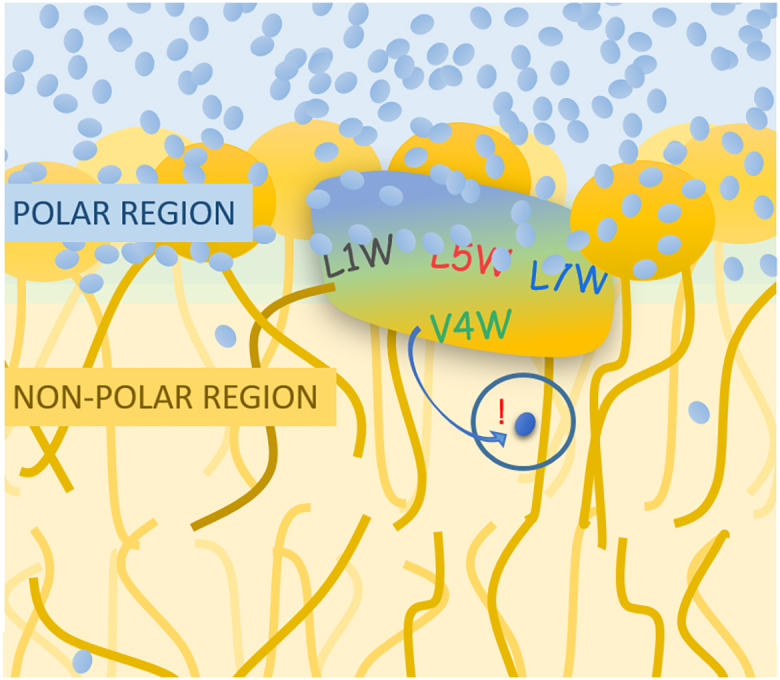
Schematic representation of the Trp inserted deeply into the hydrophobic core of the membrane being able to sense membrane disorder by differences of dynamics of diffusing water. The peptide body is marked by a tricolored oval. The color transition from blue to yellow indicates the separation of polar (*blue*) and non-polar (*yellow*) amino acids in this conformation of the peptide. The acyl chain is depicted as a brown line. The position of Trp is marked on the peptide with the exchange code (wild-type residue–position of the residue–mutant residue; L1W, *gray*; L5W, *red*; L7W, *blue*; V4W, *green*). The different regions of the membrane are labeled by the expected polarity of the environment, i.e., acyl chain region is non-polar (*core*) and the headgroup region is polar (*surface*). Blue ovals indicate water molecules. Trp position in V4W is located in the non-polar region of the peptide, and the membrane is able to sense diffusing water. The mobility of water in the membrane core changes with the membrane disorder. To see this figure in color, go online.

The fact that this cooperative perturbation is only seen for V4W does not mean it was special to this analog. Instead, it is only to be sensed in the most crucial region for membrane permeability—the most tightly packed hydrophobic region close to the interface—where Trp4 resides (illustrated in Fig. 9). This argument is already supported by the fact that leakage experiments do not suggest higher activity of V4W than of other W-viscosins. In addition, specific V4W-V4W interactions were ruled out to be crucial by a test showing that a progressive decrease in *t*_relax_ is not only sensed if further V4W is added from *R*_b_ of 0.08–0.13 but also if non-fluorescent wild-type viscosin is added to V4W (see Fig. ESI6 for details).

One may wonder why the concerted membrane perturbation by W-viscosins, which is to be considered a prerequisite for their leakage-inducing activity, is represented so sensitively by increased relaxation rates in the hydrocarbon region but neither by the local order and dynamics (*r*_∞_, *φ*) nor the final extent of relaxation (*ν*_∞_). When it comes to order and dynamics, this shows an interesting parallel to the lack of a disordering effect of another CLiP, surfactin, as studied by solid-state deuterium NMR of the acyl chains in selectively chain-deuterated lipids. Despite being described as a detergent-like molecule, surfactin did not disorder lipid chains in a detergent-like manner but caused a collective chain tilt accommodating the surfactin molecule without compromising order significantly (82). A similar behavior of the viscosin analogs could explain the lack of sensitivity of TR-anisotropy parameters to leakage-inducing perturbation. The lack of a concentration effect on *ν*_∞_ can, again, be explained by the discreteness of the “recruitment of water” concept developed above. Stronger water penetration into the hydrophobic core and a faster motion of the water will speed up the recruitment of a water molecule to an excited Trp, i.e., reduce the *t*_relax_ of the Trp4’s environment. However, as long as most Trps recruit only one water molecule, the extent of relaxation will stay the same.

### W-viscosins in the buffer: V4W aggregates and Trp1 covered by acyl chain

The values at *c*_L_ = 0 in Figs. 4 and 8 have already revealed key properties of Trps in W-viscosins in the buffer as compared to the membrane-bound state. Mostly, they move faster (lower *φ*) and more freely (lower *r*_∞_), and show a faster (short *t*_relax_) and stronger (lower *ν*_∞_) dipolar relaxation. All this is in line with what one expects for water-exposed Trp attached to a rather small molecule in solution, but some outliers indicate specific effects.

**Figure 10:**
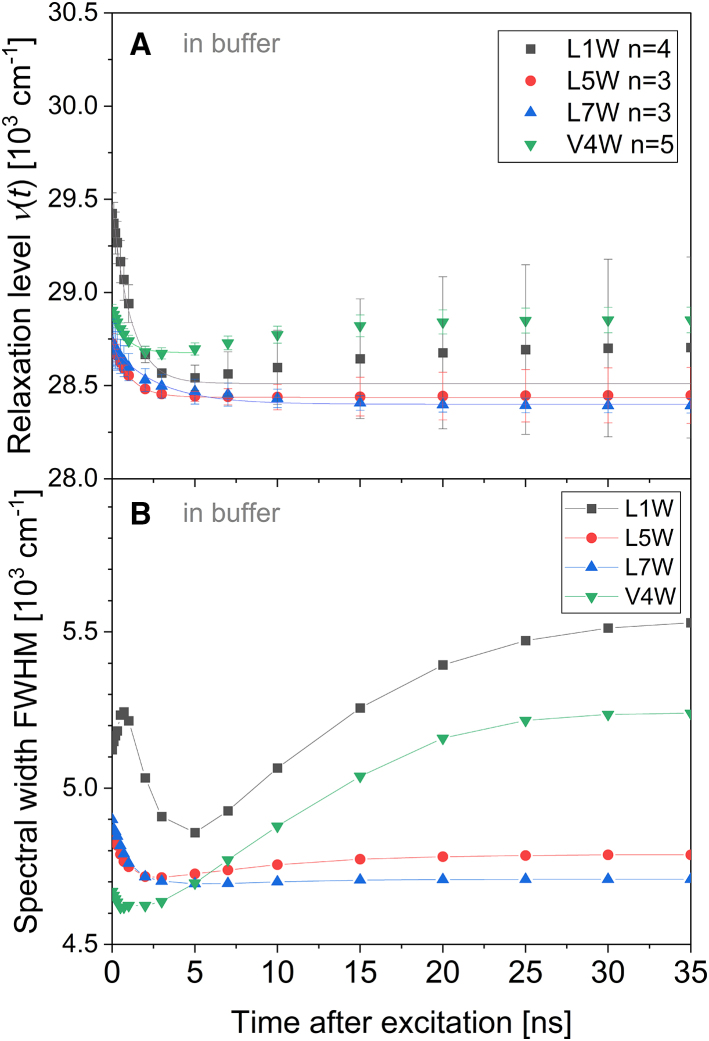
The relaxation process around W-viscosins in the buffer. (*A*) The relaxation level depicted as the spectral center of gravity of TRES is plotted as a function of time. Lines represent the monoexponential fit of data points. Note that V4W data could not be fitted monoexponentially, so only the data between 0 and 5 ns were considered. Note the high deviation between the fit and data for L1W. *n* indicates the number of independent replicates performed; error bars represent the corresponding standard deviation. (*B*) Exemplary data of the spectral width at the half-maximum intensity (FWHM; time-dependent change of the spectral width) as a function of time. Lines are to guide the eye only. The curves shown were chosen as the most representative ones, but the progression of FWHM varies from replicate to replicate. To see this figure in color, go online.

To provide more detailed insight, Fig. 10 *A* shows the relaxation level of W-viscosins in the buffer as a function of time. The relaxation level *ν*(*t*) is shown as the spectral center of gravity of TRES at distinct time points. Fig. 10 *B* shows the TR-FWHM of representative curves. Trp5 and Trp7 detect strong (lowest *ν*_∞_), continuous (80), and homogeneous (only slightly and quickly decreasing FWHM) relaxation in line with exposure to bulk water in solution. The very fast bulk solvent relaxation of the buffer cannot be detected with our instrumentation due to instrumental limits (maximal resolution up to 5 ps in an ideal environment, expected resolution in a given environment at about 250 ps). The relaxation of water fixed loosely on the exterior of the peptide may happen gradually, from the nearest layer to the next molecular layer. This process, reported to take from several to tens of picoseconds in NMR and MD simulation studies (83), may produce a local maximum in TR-FWHM under ideal circumstances. With our temporal resolution limits, we expected to see no or only a small part (the right limb) of this maximum for all peptides.

Trp in V4W and L1W senses higher energy levels of *ν*_∞_, indicating a less polar environment. Both their *ν*_∞_ and FWHM show a minimum after a few nanoseconds and then increase again.

The rise after 2–3 ns in TR-FWHM of V4W is very high and highly constant. This leads to the assumption of a subpopulation in another, less polar environment than bulk solvent. This is consistent with the quenching of Trp lifetime in buffer. The *τ*_av_ of L1W, L5W, and L7W are indeed shorter in buffer than in the membrane, but this is not the case for V4W (Fig. 8 *A*). Thus, Trp in V4W is partially not exposed to water.

**Figure 11:**
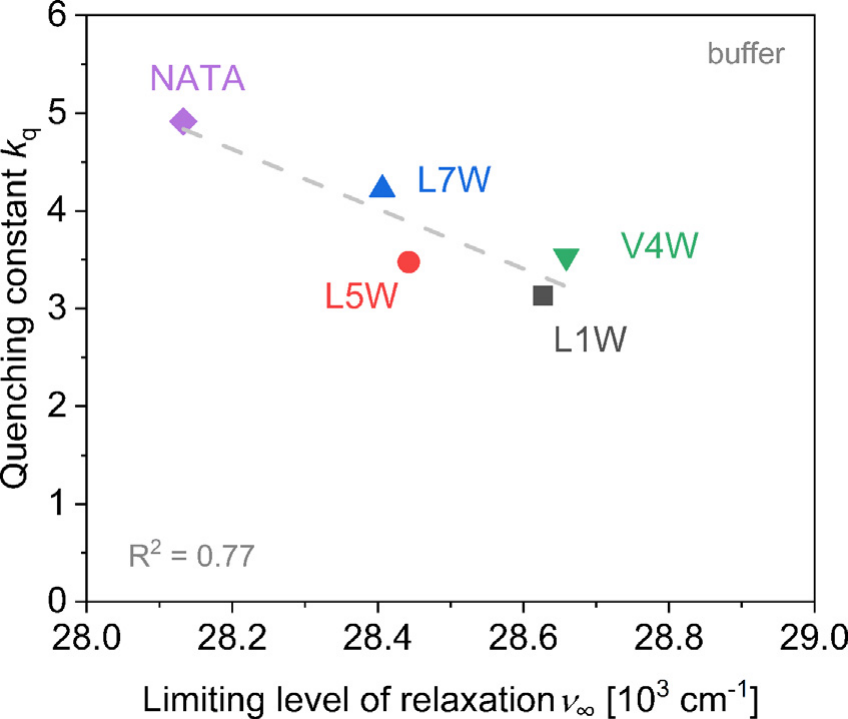
The apparent quenching rate constant, *k*_q_, shown as a function of the spectral center of gravity after infinite time, *ν*_∞_, for L1W (*gray squares*), V4W (*downward green triangles*), L5W (*red circles*), and L7W (*upward blue triangles*) in buffer. Gray dashed line represents linear fit of data points. Quality of the fit is represented by *R*^2^. Data for NATA (*violet diamond*) was additionally included for the test for data correlation of the buffer measurements. To see this figure in color, go online.

To gain additional clarity on this matter, the quenching experiments with acrylamide were performed with W-viscosins in buffer. The results are shown in Fig. 11. NATA is the maximally quenched, fully buffer-exposed control sample. Trp5 is quenched less than Trp7, probably due to the more efficient shielding of Trp by the peptide. Trp of L7W may have less possibility to bury itself into the peptide interior due to the flanking by two polar serins. Trp in L1W is least quenched and most shielded, likely due to the shielding effect of the acyl chain in its vicinity. Trp4 may be buried in the peptide, or multiple monomers may aggregate and shield V4W from the buffer.

## Conclusion

TR fluorescence of Trp as an intrinsic fluorophore or a supposedly conservative label (replacing a non-polar side chain) can provide a wealth of information about the interactions of a peptide with a membrane and specific conditions at the site of Trp. Whereas the interpretation of individual data is challenging given the complexity of Trp fluorescence, a combination of many observables of TCSPC measurements may give rise to a fairly consistent and clear picture. The findings obtained here may serve as a blueprint for interpreting parameters found for Trps in other membrane-binding peptides.

To illustrate the power of the approach, let us look at individual labels. For viscosin, probably the most informative label was Trp4, replacing a valine embedded within the acyl chain region of the membrane and, apparently, without contact to lipid carbonyls or polar groups from the peptide. The extent of dipolar relaxation should allow comparison of the local membrane perturbation of different membranes. Even more importantly, the rate of relaxation of Trp4 was found to be sensitive to concentration-dependent, concerted membrane perturbation that appears to be a prerequisite for membrane leakage and antimicrobial action. A “water recruitment” hypothesis is presented to account for this observation: Trp4 residues that do not interact with any water molecule in the ground state will become more polar upon excitation but show dipolar relaxation only as a water molecule diffuses to their site.

Trp7 showed a very unusual relaxation behavior, being essentially independent of water reorientation. Instead, the residue is flanked by two Ser residues, which restrict its mobility. This seems to enforce some Trp7 to already assume an orientation in the ground state that becomes the relaxed orientation in the excited state. Trp-Ser interactions also seem to govern further relaxation.

Trp5, which is attached to the peptide backbone in a position located at the membrane surface, seems to be distributed between two main orientations. MD simulations favor the more membrane-embedded “down” orientation, but experimental data mainly reflect a highly water-exposed “up” orientation.

Trp1 shows a more standard behavior in the membrane—it interacts with water and lipid carbonyls and relaxes in a homogeneous, continuous fashion. Its proximity to a Glu residue did not result in any peculiar effects, but the adjacent attachment of the lipidic chain might give rise to a hydrophobic covering of Trp1 in solution.

## Author contributions

I.C. and J.S. performed TR fluorescence-based research and analyzed the data. P.M. synthesized the W-viscosin under the supervision of A.M. and J.C.M. V.D.R. and N.G. performed and analyzed MD simulations and concentration determination under guidance and advice from J.C.M. I.C. and J.S. drafted the manuscript. H.H. initiated and supervised the work. H.H., I.C., N.G., and J.C.M. edited the manuscript. All authors read and approved the final manuscript.

## Supporting material

Document S1. Supporting text, Figs. ESI1–ESI6, and Figs. S1–S84

Document S2. Article plus supporting material
