# Supplementary material for: Time-resolved fluorescence of tryptophan characterizes membrane perturbation by cyclic lipopeptides
Source: Biophys J. 2024 Jun 22;123(16):2557–73. doi: 10.1016/j.bpj.2024.06.022 (PMC11365112; doi:10.1016/j.bpj.2024.06.022)
Supplement: Document S1. Supporting text, Figs. ESI1–ESI6, and Figs. S1–S84 [file mmc1.pdf]

Biophysical Journal, Volume *123*

Supplemental information

Time-resolved fluorescence of tryptophan characterizes membrane perturbation by cyclic lipopeptides

Iulia Carabadjac, Jessica Steigenberger, Niels Geudens, Vic De Roo, Penthip Muangkaew, Annemieke Madder, José C. Martins, and Heiko Heerklotz

Electronic Supplementary Information to

Time-resolved fluorescence of tryptophan characterizes membrane perturbation by cyclic lipopeptides

Iulia Carabadjac[1#], Jessica Steigenberger[1#], Niels Geudens[2], Vic De Roo[2,3], Penthip Muangkaew[3], Annemieke Madder[3], José C. Martins[2], Heiko Heerklotz[1,4,5*]*

[1] Institute of Pharmaceutical Sciences, University of Freiburg, Hermann-Herder-Str. 9, 79104 Freiburg, Germany.[2] NMR and Structure Analysis Research Group, Department of Organic and Macromolecular Chemistry, Ghent University, Ghent, Belgium. [3] Organic and Biomimetic Chemistry Research Group, Department of Organic and Macromolecular Chemistry, Ghent University, Ghent, Belgium. [4] Center for Biological Signaling Studies (BIOSS), Faculty of Biology, University of Freiburg, Schänzlestr. 18, 79104 Freiburg, Germany. [5] Leslie Dan Faculty of Pharmacy, University of Toronto, 144 College Street, Toronto, ON, Canada M5s 3M2

Contents:

PART A: FLUORESCENCE

1. Chemical synthesis of viscosin and analogues

2. Time-resolved emission spectra (TRES) measurements

3. Reasons to use v_∞ for TRES analysis

4. Time-resolved anisotropy measurements: Settings and procedure

5. Time-resolved quenching assay: Settings and procedure

6. Membrane permeabilization and kinetics by TCSPC: Settings and procedure

7. Membrane partitioning: Settings and procedure

8. DLS measurement results

9. Measurements with addition of viscosin

10. References part A

PART B: MOLECULAR DYNAMICS

11. Introduction

12. MD simulations of viscosin in POPC bilayer over longer simulation times, starting in different localizations

    250 ns MD simulation of 'wild-type' viscosin, starting in the aqueous phase

    500 ns MD simulation of 'wild-type' viscosin, starting at the bilayer-water interface

13. Impact of the starting location and orientation of viscosin within the bilayer

    50 ns MD simulation of viscosin initially at the bilayer-water interface

    50 ns MD simulation of viscosin initially in the interfacial region

    50 ns MD simulation of viscosin initially located at the center of the bilayer

    150 ns MD simulation of viscosin initially oriented upside down in the interfacial region

14. MD simulations of the tryptophan-labelled lipopeptides – supporting information

    MD simulation of L1W

    MD simulation of V4W

    MD simulation of L5W

    MD simulation of L7W

15. References part B

part A: Fluorescence

1. Chemical synthesis of viscosin and analogues

All procedures for the synthesis and purification of specific building blocks as well as solid-phase peptide synthesis, and cyclization release of the viscosin-analogs were carried out as described previously for the total synthesis of pseudodesmin A and its analogues[1].

2. Time-resolved emission spectra (TRES) measurements

Measurements were performed with the help of the TRES wizard tool. To this end, lifetime decay histograms of all samples were recorded for 25 s per nm over a detection wavelength range of 290 – 450 nm in 2 nm steps selected by Omni-λ300 Grating monochromator with an integration bin-width ranging from 5 to 10 nm. Trp was selectively excited at 280 nm with a repetition frequency of 20 MHz by a vertically polarized (V) VisUV laser. Excitation and detection attenuators were controlled by the TRES wizard (set to 100%) of the EasyTau software to yield reasonable detection count rates (<1% of sync rate) to avoid pile-up effects. Emission was detected by the PMA-C 175-M single-photon detector module with an emission polarizer set to 54.7° magic angle (M). The instrument response function (IRF) was acquired with a light scattering standard Ludox®-HS 40 (Sigma-Aldrich) solution (5 µl in 2 ml ultrapure water, width resolution ~50 ps) at 280 nm (VM polarization). Acquired lifetime decays were fit tri-exponentially by Equation ESI-1 with all lifetimes $\tau_1 - \tau_3$ set globally. Examples of fluorescence decays of all peptides are shown in Fig. ESI-1.

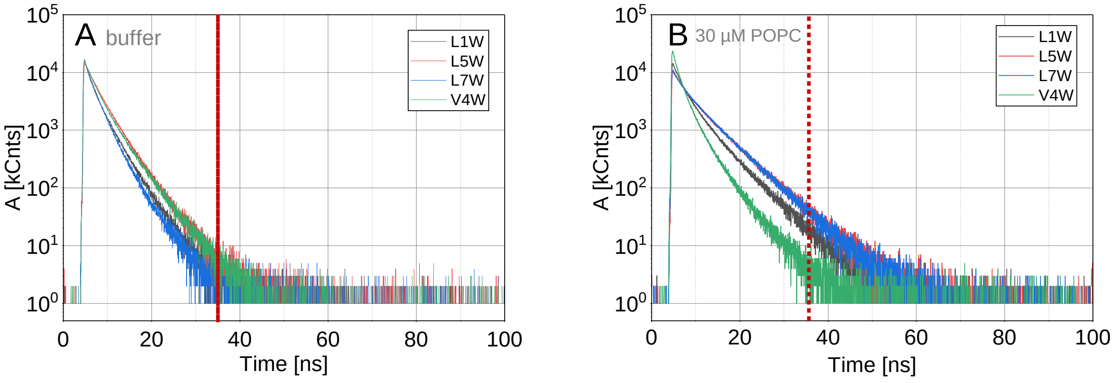

Figure ESI-1: Exemplary fluorescence decays of W-viscosins in buffer (A) and with 30 µM POPC-LUVs (B). The intensity A in kCnts is plotted as a function of time in ns. The red line marks the approximate fluorescence intensity level after 35 ns after excitation, which is used as the limit for time-resolved anisotropy and time-resolved spectral shift analysis. Note that at this time point the fluorescence is almost fully decayed but still not at the level of the dark count.

In the second step, the peak-normalized emission spectra at specific time points after excitation (TRES) were created using the fit data of the TR lifetime decays. For data analysis, it is important to include TRES at times long enough after excitation to ensure that the limiting spectral shift has been reached and recorded. However, the data must also be sufficiently well resolved. The temporal limit was set to 35 ns after excitation (Figure ESI-1). This time interval corresponds to five times the longest lifetime of the fluorophore (longest lifetime ~ 6-8 ns for all W-peptides) and with that to a time point, where almost all fluorescence is decayed, but the signal not yet equal to the dark count.

The variable $F(\lambda,t)$ represents the total integral counts at a certain wavelength and time, t, after the excitation pulse. A is the pre-exponential factor (amplitude) and τ_i is the decay time at a certain wavelength λ, respectively. No molecular significance is assigned to the components i. The number of components is increased until the fit doesn't become better (in this study three components were used, which corresponds to literature number for Trp lifetimes, if Trp interacts with the environment[2]).

$$F(\lambda,t) = \frac{H(\lambda)\cdot \Sigma_i A_i(\lambda)\cdot e^{-\frac{t}{\tau_i}}}{CF(\lambda)} \qquad \text{ESI-1}$$

$F(\lambda,t)$ is corrected by the factor $H(\lambda)$, which is defined as $H(\lambda) = \frac{F_{SS}(\lambda)}{\Sigma_i A_i(\lambda)\cdot\tau_i}$, so that the time-integrated intensity at each wavelength $I(\lambda)$ becomes equal to the steady state intensity $F_{ss}(\lambda)$ at that wavelength. Equation ESI-1 is normalized by a correction factor, CF, to account for the wavelength-dependent grating and detector sensitivity, $DS(\lambda)$. CF is calculated by $CF = \frac{I(\lambda)}{I(\lambda)\cdot DS(\lambda)}$.

Additionally, the amplitude-averaged lifetime was calculated according to:

$$\tau_{av} = \frac{\sum_{i=1}^{3} A_i\cdot\tau_i}{\sum_{i=1}^{3} A_i} \qquad \text{ESI-2}$$

where A denotes the integral of the amplitude over the whole spectrum.

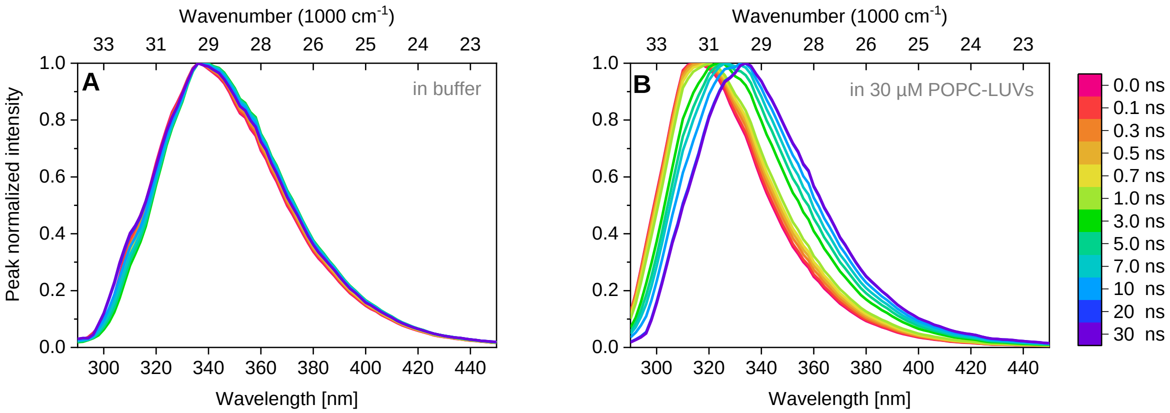

Figure ESI-2: Exemplary TRES of V4W in buffer (A) and with 30 µM POPC-LUVs (B). The peak normalized intensity is plotted as a function of emission wavelength (lower X-axes) or wavenumber (upper X-axes) at distinct time points from 0 up to 35 ns.

3. Reasons to use ν∞ for TRES analysis

$\nu(t)$ of all examined W-viscosins are shown in Figure ESI-3.

In general, $\nu(0)$ can be estimated by measurements of the absorption and emission spectra in completely non-polar solvents[3–5]. However, this was not possible for the W-viscosins for three reasons: First, Trp has two different, nearly isoenergetic excited states (^1L_a and ^1L_b) in the presence or absence of

H-bonding to N-H of the indole, hence two very different shapes of absorption and emission spectra in the polar and non-polar environment. Second, the W-viscosins tend to self-assemble in neat solvents with low dielectric constants, making it impossible to access $\nu(0)$ of the monomers apart from that of the aggregates, where Trp may be buried in the interior. Moreover, the estimation of $\nu(0)$ at low temperatures (below -60 °C) in not-crystallizing solvents such as glycerol or propylene glycol is not possible with available spectroscopic equipment in most laboratories. Thus, it is not possible to resolve the initial energy of the unrelaxed excited state by the measurements with our experimental setup. Furthermore, $\nu(0)$ may also differ for Trp in different positions in W-viscosin according to residues in its neighborhood, which would not provide us with any useful information about the environment of W-viscosin. Due to the uncertainty about $\nu(0)$, $\Delta\nu$ cannot be calculated for W-viscosins.

4. Time-resolved anisotropy measurements: Settings and procedure

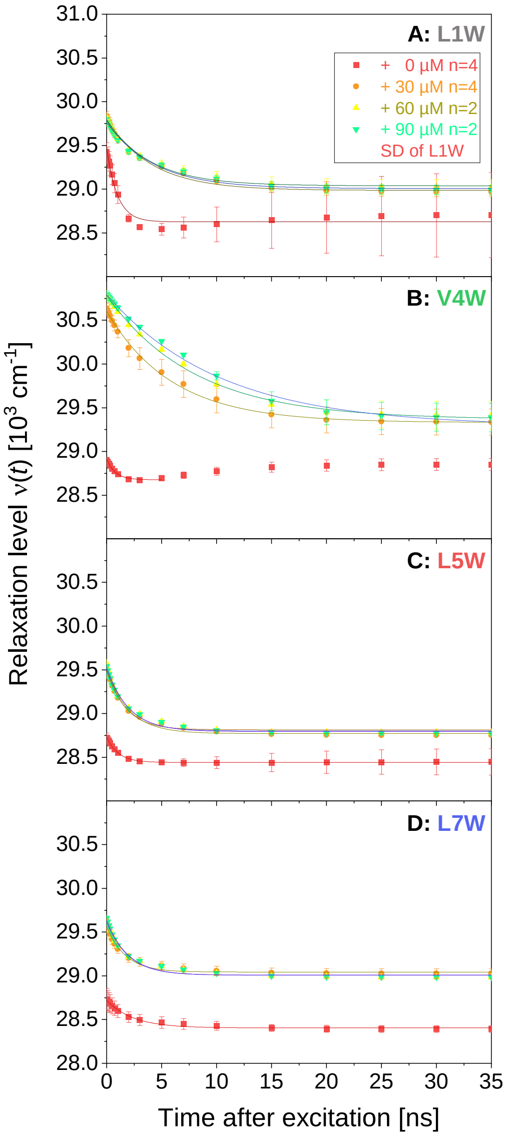

Figure ESI-3: The relaxation level showed as spectral center of gravity of TRES plotted as a function of time. Different panels show the results for L1W (A), V4W (B), L5W (C), and L7W (D) with 0 (red squares), 15 (orange circles), 30 (yellow triangles up), 60 (green triangles down), or 90 µM (blue diamonds) POPC-LUVs. Symbols represent actual data points. Lines represent mono-exponential fit of data points. The letter n indicates the number of independent replicates performed.

Time-resolved anisotropy (TR-anisotropy) was measured using the same instrumental setup as for TRES experiments. Emission was recorded at 340 nm. The emission polarizer was set to 0° (vertical, V), 54.7° (magic angle, M), and 90° (horizontal, H), respectively. To avoid fluorescence bleaching effects, a total number of six measurement cycles, accumulated for 5 s each, was performed. Measurements were performed using a script so that all data of a series and the G Factor were acquired with the same settings. The G Factor (correction for differences in the sensitivity of the detection system for vertically and horizontally polarized light) was determined at 340 nm emission wavelength for each series of experiments with NATA in TRIS buffer (absorption < 0.1 at 340 nm), a fluorophore solution displaying an anisotropy of ~ 0.

5. Time-resolved quenching assay: Settings and procedure

Fluorescence lifetime decays of W-viscosins and NATA in buffer or in 30 µM POPC LUVs at five concentrations of acrylamide (0, 0.1, 0.2, 0.3, 0.4 M for W-Viscosins and 0, 0.05, 0.1, 0.15, 0.2 M for NATA) were acquired after 4 to 8 h of incubation at 25°C. The fluorescence lifetime of Trp was recorded up to 10^4 peak counts. The resulting histograms were fitted globally over the 5 acrylamide concentrations with Equation ESI-3.

$$I(t) = \int_{-\infty}^{t} IRF(t') \sum_{\mathrm{i}=1}^{n_{\mathrm{Exp}}} A_{\mathrm{i}} \cdot e^{-\frac{t-t'}{\tau_{\mathrm{i}}}} \cdot dt' \qquad \text{ESI-3}$$

The intensity of fluorescence over time, denoted as $I(t)$, is expressed as a function of time, where t' represents the time shift of the Instrument Response Function (IRF) in nanoseconds. Time is represented as t, A_{i} is the pre-exponential factor of decay component i, τ_{i} denotes the lifetime of decay component i in nanoseconds, and n_{Exp} represents the number of exponential terms.

Decay parameters were adjusted iteratively until the reduced χ^2 reached its local minimum. Specific conditions were imposed, requiring all amplitudes to be greater than zero. The τ_{i} values were globally fitted and constrained to a fixed value. Uniform fitting was applied to all decays.

6. Membrane permeabilization and kinetics by TCSPC: Settings and procedure

The fluorescence lifetime decays of calcein were acquired with the FluoTime100 spectrometer (PicoQuant, Berlin, Germany) using TCSPC. Calcein was excited with a 467 nm pulsed-laser diode (LDH-P-C-470, PicoQuant, Berlin, Germany) operated by a PDL 800-D laser driver (pulse width: 20 ps; repetition rate: 20 MHz). Lifetime decays were recorded for 30 s (resolution of 25 ps per bin) using a PMA 175-N detector (PicoQuant, Berlin, Germany) and an OG530 long-pass filter. Detection attenuators were set to ensure a detection rate of < 1% of excitation pulses. The IRF was measured by a Ludox HS-40 solution (5 µl in 2 ml ultrapure water) and used for reconvolution fitting.

Peptide-induced membrane leakage does not lead to only two calcein populations. It is much more likely that all liposomes are damaged to a different extent and therefore lose different amounts of calcein. Experience has shown that a free biexponential fit provides the most reproducible and robust results. Therefore, decay curves were fitted biexponentially with the FluoFit software (PicoQuant) according to Equation ESI-4. The fluorescence lifetime of each population is described by τ. The exponential pre-factor B provides information about the amount of calcein present in each fraction. The subscripts E and F represent the encapsulated and free fractions of calcein, respectively.

$$B(t) = B_{\mathrm{E}} \cdot e^{-\frac{t}{\tau_{\mathrm{E}}}} + B_{\mathrm{F}} \cdot e^{-\frac{t}{\tau_{\mathrm{F}}}} \quad \text{ESI-4}$$

7. Membrane partitioning: Settings and procedure

Measurements followed a common script to keep instrumental settings the same throughout all experiments. Samples were excited with the VisUV at 280 nm with the excitation attenuator set to position 5 (open) and excitation polarization to 0°. The laser pulse frequency was set to 80 MHz and laser intensity to 7.2. Emission was recorded with emission polarizer set to magic angle (54.7°), detection attenuator set to 100% in the wavelength range from 290 to 450 nm with a step width of 2.0 nm and an integration time of 1.0 s per point. All measurements were performed in two runs to exclude fluorescence bleaching effects. The fluorescence intensities of the W-viscosins (F_{W}) at respective lipid concentrations, c_{L}, were measured after 2-hour incubation time. All samples were incubated at 25°C, protected from light and stirred during incubation and measurement. The fluorescence intensity of TRIS buffer was measured as blank F_{B}. The fluorescence intensities of NATA were recorded immediately (< five minutes) upon lipid addition. F_{W} were corrected for F_{B} and the wavelength-dependent detector sensitivity (DS) by Equation ESI-5 to create the steady-state emission spectra of all samples, I_{W}.

$$I_{\mathrm{W}} = (F_{\mathrm{W}} - F_{\mathrm{B}}) \cdot DS \quad \text{ESI-5}$$

Emission spectra of NATA samples were used to correct the emission spectra of W-viscosins at respective lipid concentrations, $I'_{\mathrm{W}}(c_{\mathrm{L}})$, for scattering light effects by Equation ESI-6.

$$I'_{\mathrm{W}}(c_{\mathrm{L}}) = I_{\mathrm{W}}(c_{\mathrm{L}}) \cdot \frac{I_{\mathrm{NATA}}}{I_{\mathrm{NATA}}(c_{\mathrm{L}})} \quad \text{ESI-6}$$

The resulting spectra are shown in Figure ESI-5.

The partition coefficient of the W-viscosins between buffer and the POPC-LUVs was investigated. Here, the fact that the fluorescence intensity of tryptophan at any given wavelength correlates linearly with the amount of membrane-bound W-viscosin was exploited. In general, tryptophan membrane binding is accompanied by an increase in the fluorescence intensity and a blue shift of the emission[6–8]. However, light scattering has the opposite effect. Scattered light reduces the fluorescence intensity because it attenuates both the excitation and emission light. In addition, it can lead to an apparent red shift of the emission spectrum. For this reason, light scattering may obscure the effect of membrane binding of the CLiP on the emission spectrum[6]. To correct for these effects of scattered light, emission spectra of NATA samples with increasing lipid concentration were recorded in addition to emission spectra of samples with W-viscosins. It was assumed that NATA would not bind to the membrane. Surprisingly, the intensity of the emission spectra of NATA changed with incubation time. Samples after two hours incubation showed a biexponential lifetime decay and increasing fluorescence intensities with increasing lipid concentration, c_{L}, hence indications for binding of NATA to the membrane. Fluorescence data obtained immediately upon lipid addition and mixing did not reveal any signs of NATA binding to the membrane, as the fluorescence lifetime decay displayed a mono-

exponential decay with a constant lifetime independent of c_L. Moreover, the emission spectra showed the expected decrease in the fluorescence intensity with increasing c_L. Therefore, these data were used to subtract scattering light effects from the emission spectra of the W-viscosins.

8. DLS measurement results

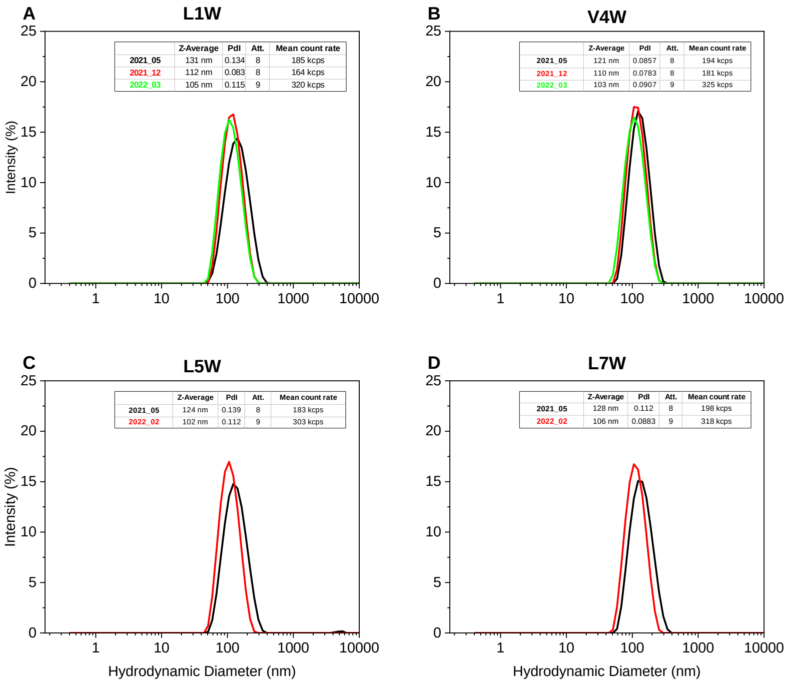

Figure ESI-4: DLS measurements of ~ 6.6 µM W-viscosins with 30 µM POPC-LUVs . The intensity distribution of samples with L1W (A), V4W (B), L5W (C) and L7W (D) are displayed. Tables list the Z-average of the hydrodynamic diameter, the polydispersity index (PDI), attenuator settings, and the mean count rate.

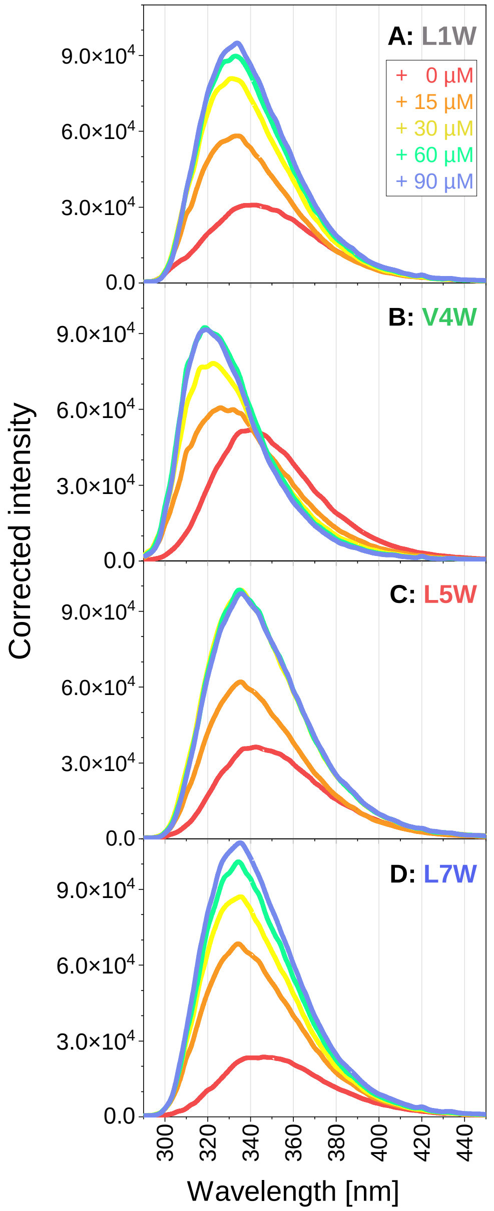

Figure ESI-5: The emission spectra of L1W (A), V4W (B), L5W (C), and L7W (D) with either 0 µM (red), 15 µM (orange), 30 µM (yellow), 60 µM (green), and 90 µM (blue) POPC-LUVs after 2 hours incubation time. The emission spectra are corrected for scattering light effects.

9. Measurements with addition of viscosin

Let us assume, that the increase in t_{relax} of V4W is indeed a result of some kind of specific peptide-peptide interaction in the membrane, which is possible for V4W, but not for the other W-viscosins. Then, the addition of other W-viscosins to the V4W-sample should disorder the membrane further, but not increase the amount of specific V4W-V4W interactions. For t_{relax} depending *on the specific peptide-peptide interaction* nothing should change. For t_{relax} depending on *the membrane order*, the value should decrease.

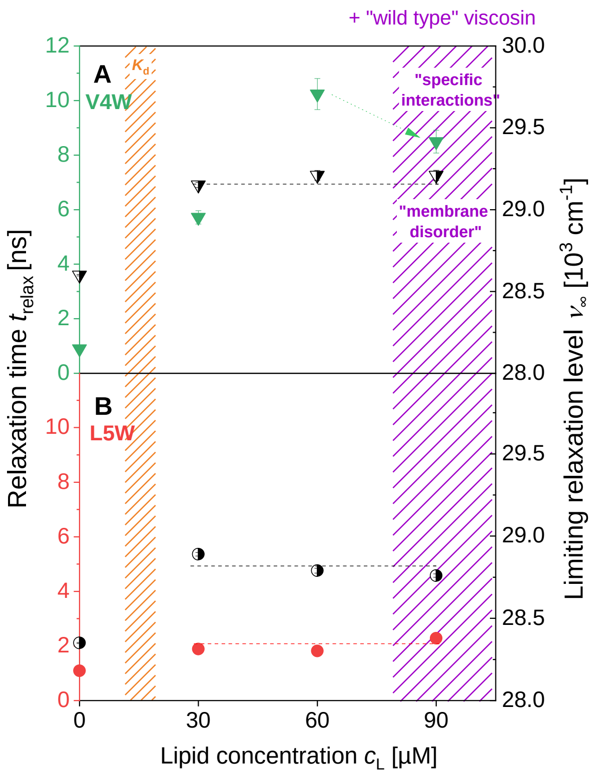

*Figure ESI-6: **A** shows on the left Y-axes the dipolar relaxation time, t_{relax}, (green triangles down) of V4W in buffer, at 30 µM lipid (P/L~1:5), 90 µM lipid (P/L~1:14), and at 90 µM lipid with the same amount of V4W + "wild type" viscosin up to a of P/L~1:5 (equivalent to V4W at 30 µM lipid alone). The right Y-axes displays the corresponding limiting level of relaxation depicted as the spectral center of gravity of TRES, ν_∞ (black triangles half-filled). **B** shows the results for L5W samples (red and black filled and half-filled circles, respectively). The violet background indicates the sample with "wild-type" viscosin added. The orange shaded area marks the lipid concentration range at which half of the W-viscosins are bound to the membrane, the dissociation constant K_d. All lines and arrows are to guide the eye only. The text boxes in A indicate the areas, where t_{relax} would have been expected after "wild-type" viscosin addition to the sample for the different hypotheses of the origin of t_{relax} change upon changes in concentration. The upper text box marks the area expected for specific peptide-peptide interaction; the lower box indicates where t_{relax} would have been if viscosin had been as membrane-active as V4W and led to the same amount of membrane disorder.*

Instead of W-viscosins, the "wild-type" viscosin was used. Viscosin has the advantage of being "spectroscopically silent" because no fluorescent residues are present, but still being membrane-active. This means that the addition of viscosin to V4W will not disturb the fluorescence signal from Trp of V4W. Since viscosin is less membrane perturbing than V4W (Figure 2 of main manuscript) a slighter decrease of t_{relax} than to the level of V4W is expected. Note that because of slightly different K_d of viscosin (~ 3 µM[9]) and V4W (~ 20 µM) the lipid to peptide ratio of L/P = 5 isn't the same for V4W

alone or V4W and viscosin. As a control, we added the same amount of viscosin to L5W samples as well. The impact of the non-fluorescent viscosin on t_{relax} is shown in Figure ESI-6 for both W-viscosins (A: V4W, B: L5W). The observed decrease in t_{relax} of V4W, but not for L5W, supports our hypothesis about t_{relax} being independent of specific peptide-peptide interactions.

10. References

(1) De Vleeschouwer, M.; Van Kersavond, T.; Verleysen, Y.; Sinnaeve, D.; Coenye, T.; Martins, J. C.; Madder, A. Identification of the Molecular Determinants Involved in Antimicrobial Activity of Pseudodesmin A, a Cyclic Lipopeptide From the Viscosin Group. *Front. Microbiol.* **2020**, *11* (April), 1–14. https://doi.org/10.3389/fmicb.2020.00646.

(2) Ghisaidoobe, A.; Chung, S. Intrinsic Tryptophan Fluorescence in the Detection and Analysis of Proteins: A Focus on Förster Resonance Energy Transfer Techniques. *Int. J. Mol. Sci.* **2014**, *15* (12), 22518–22538. https://doi.org/10.3390/ijms151222518.

(3) Horng, M. L.; Gardecki, J. A.; Papazyan, A.; Maroncelli, M. Subpicosecond Measurements of Polar Solvation Dynamics: Coumarin 153 Revisited. *J. Phys. Chem.* **1995**, *99* (48), 17311–17337. https://doi.org/10.1021/j100048a004.

(4) Fee, R. S.; Maroncelli, M. Estimating the Time-Zero Spectrum in Time-Resolved Emmsion Measurements of Solvation Dynamics. *Chem. Phys.* **1994**, *183* (2–3), 235–247. https://doi.org/10.1016/0301-0104(94)00019-0.

(5) Jimenez, R.; Fleming, G. R.; Kumar, P. V.; Maroncelli, M. Femtosecond Solvation Dynamics of Water. *Nature* **1994**, *369* (6480), 471–473. https://doi.org/10.1038/369471a0.

(6) Ladokhin, A. S.; Jayasinghe, S.; White, S. H. How to Measure and Analyze Tryptophan Fluorescence in Membranes Properly, and Why Bother? *Anal. Biochem.* **2000**, *285* (2), 235–245. https://doi.org/10.1006/abio.2000.4773.

(7) Lakowicz, J. R. *Principles of Fluorescence Spectroscopy*; 2006.

(8) Yammine, A.; Gao, J.; Kwan, A. H. Tryptophan Fluorescence Quenching Assays for Measuring Protein-Ligand Binding Affinities: Principles and a Practical Guide. *bio-protocol* **2019**, *9*. https://doi.org/10.21769/BioProtoc.3253.

(9) Steigenberger, J.; Verleysen, Y.; Geudens, N.; Madder, A.; Martins, J. C.; Heerklotz, H. Complex Electrostatic Effects on the Selectivity of Membrane-Permeabilizing Cyclic Lipopeptides. *Biophys. J.* **2023**, *122* (6), 950–963. https://doi.org/10.1016/j.bpj.2022.07.033.

part B: Molecular Dynamics

Summary: The lipopeptide MD simulations were analyzed through comprehensive monitoring of total energy, temperature, and density across the MD simulations. Additionally, the stability and convergence of the membrane bilayer were evaluated using the "area per lipid" metric, complemented by monitoring of RMSD and hydration patterns around each residue's alpha carbon. The starting location and orientation for the lipopeptide in each simulation was chosen based on a long duration MD simulation (250 ns) of the insertion of viscosin from the aqueous phase into a pre-equilibrated and solvated POPC membrane. Starting from the inserted position, a second 500 ns MD simulation revealed a stable disposition with respect to the POPC membrane without significant fluctuations in location or orientation. Therefore, all simulations starting from the same initial position as used in this work were limited to 100 ns. Test simulations where viscosin was placed at different positions in the POPC membrane (phosphatidyl head group, glycerol backbone or POPC bilayer center) demonstrated viscosin adopts the same final disposition well within the first 50 ns of the simulation time. A simulation starting from an upside down orientation (i.e. hydrophobic side chains turned to the aqueous phase) showed that reorientation to the expected disposition is complete within 80 ns. Thus, these simulations revealed that 100 ns simulation time is sufficient to capture the dynamics of the system, and bias linked to the initial location and orientation of the lipopeptide is suitably addressed.

11. Introduction

Original MD simulations as described in the manuscript for all W-viscosins (Trp-analogues of viscosin) were 100 ns in duration with the various W-viscosin analogues initially positioned with the same orientation and with its center at the level of the glycerol backbone, referred to as the interfacial region hereafter. The position and orientation was based on previous experience with simulating cyclic lipodepsipeptides in house, and is substantiated hereafter (section 2). The procedures used are described in the materials and methods section of the original and now reworked manuscript. Relevant information with respect to all MD simulations described in the manuscript are collected in section 4 and could be included as sup. mat in the reworked manuscript, if so desired.

Section 2 of this document presents two longer duration MD simulations using ‘wild-type’-viscosin. In a first, 250 ns simulation, viscosin was placed well above the pre-equilibrated and solvated POPC bilayer, and allowed unforced insertion, which occurred around 100 ns into the simulation. A second, 500 ns simulation starts from viscosin inserted at the bilayer-water interface in the same way as described above for the Trp-viscosin analogues, i.e. with the molecular center positioned at the level of the phosphatidylcholine head groups and an orientation fitting amphipathic interaction expectations. The simulation time was extended from 100 ns to 500 ns to allow 450 ns of trajectory monitoring, as opposed to the 50 ns monitoring in the original MD simulations of the Trp-viscosin analogues, thus allowing to assess peptide-bilayer behavior over a nine-fold longer observation period and potential bias towards the initial conditions. During the extended simulation time, no additional events of significance are seen to occur compared to the shorter MD simulation.

Section 3 reports on 50 ns simulations on wt-viscosin, where its location within the bilayer was varied (3.1-3.3) or it was turned upside down at the interfacial region (3.4), thus allowing to establish the relaxation time required for viscosin to reach a stable location and orientation within the bilayer.

For each simulation trajectory, a variety of data as a function of the simulation time is extracted and collected hereafter. The system's (i) total energy, (ii) temperature and (iii) density is shown to demonstrate the evolution and stability of simulation parameters; (iv) the area per lipid metric is utilized to evaluate bilayer stability; (v) the total number of hydration water molecules is obtained by summing the number of water molecules within a 5 Å sphere of each Cα carbon, including that of the acyl chain while (vi) the heavy atom RMSD (including the acyl chain) of the lipopeptide with respect to its structure at t = 0 is used to assess conformational stability; (vii) the water exposure of individual Cα carbons within a 5 Å shell averaged over the simulation trajectory is graphed as a normalized average ± s.d. (1.0 = for residue with highest exposure – see also below), while (viii) the electron density along the z axis of the simulation box (averaged over the final 50 ns (section 2) or 10 ns (section 3) of the simulation) as a function of distance from the bilayer center for the phosphocholine (PC) and oleoyl- and palmitoyl (OL, PA) aliphatic chains and for the peptide.

With respect to the time-averaged water exposure graphs, we previously found that the water exposure values for individual Cα carbons shows a good to excellent fit with experimental NMR data gauging water exposure via paramagnetic relaxation probes[10]. More specifically, viscosinamide, the natural Gln2 analogue of viscosin (having Glu2) was investigated in DPC micelles and in the presence of water-soluble paramagnetic relaxation agents (=PRA; GdDTPA was used) using NMR spectroscopy. The PRAs quench ^{1}H resonance intensity to an extent that depends on the average proximity of the PRA to the ^{1}H monitored. As the PRAs are only soluble in the aqueous phase and do not associate or dissolve in DPC the quenching occurs to a degree that reflects relative exposure to these PRAs and therefore to the water phase. The figure 6 in Geudens *et al*[10] shows a confrontation of the degree of quenching of CαH, represented in a normalized fashion, with the normalized exposure of individual amino acids to water molecules within a 5 Å shell of their the Cα as collected from the final 200 ns unrestrained MD simulations of viscosinamide in DPC micelles in the AMBER environment. A good to excellent match is found, a finding which could also be extended to larger lipopeptide structures (unpublished data). Clearly, Gln2 and Ser6, occurring on the same face of the helix, are exposed to the PRA agent, while Val4 is buried towards the bilayer center and has little if any exposure, indicating an average location in accordance to expectations for an amphipathic helical structure. Taken all together, we are confident that the water exposure extracted from MD simulations can be used as a good indicator of overall orientation of a peptide with respect to the micelle- and by extension bilayer-water interface.

Finally, snapshots of the simulation are provided where appropriate. All together we propose from the data provided that the initial location and orientation of viscosin and its derivatives chosen during all simulations does not lead to significant bias when focusing analysis on the final 50 ns of a 100 ns MD simulation, starting from lipopeptides located with their center at the level of the phosphatidylcholine head groups and expected amphipathic orientation.

12. Assessing the impact of the starting position of viscosin with respect to the POPC bilayer

All simulations in this section were performed using the same protocol, the only difference being the initial location of viscosin (aqueous vs. bilayer-water interface) and duration (250 ns vs. 500 ns respectively) of the simulation. These simulations allow to assess possible bias of the results towards the initial conformation and, once bilayer associated, the minimal time required to effectively sample the behavior of viscosin at this interface. The simulations described below show from a fully solvated state in the aqueous phase, viscosin positions itself with respect to the bilayer as can be expected from

its amphipathic character. It also shows a highly similar trajectory for the bilayer associated state irrespective of the initial position. Both simulations show that, starting from a bilayer associated initial state, extension of the trajectory from 100 ns to 500 ns does not introduce new events as to the behavior of viscosin, indicating that possible kinetic trapping close to the initial conformation is unlikely (see also section 3). As such, these simulations thus address concerns regarding the impact of the initial state, and the simulation time used to describe the behavior of viscosins within the solvated and pre-equilibrated POPC bilayer, notably establishing whether kinetic trapping.

MD simulation of 'wild-type' viscosin, starting in the aqueous phase

This MD simulation was conducted with wild-type viscosin situated in the aqueous phase, not interacting with the solvated POPC bilayer. Figures 1-3 depict the stability of simulation parameters (total energy, temperature, density) over time. The "area per lipid" metric was utilized to evaluate membrane stability and convergence (Figure 4). The simulation, intended for to last 250 ns was cut short to ~225 ns due to an unplanned interruption, and not restarted as no evolution was apparent, while insertion had already taken place. Approximately 94 ns into the simulation, the 9-amino acid lipopeptide integrated itself into the membrane bilayer, as clearly evident from the reduction in water exposure (Figure 5). The fluctuations in RMSD during insertion of viscosin is similar to those in the period before insertion indicating the conformation is not significantly impacted during insertion (Figure 6). Analysis of hydration around each residue's alpha carbon offered insights into the lipopeptide's orientation and insertion depth (Figure 7), a validation previously demonstrated with a closely related lipopeptide, viscosinamide A. From this analysis, we conclude that viscosin integrates into the membrane bilayer remaining close to the surface, with Val4 being the most shielded from the aqueous phase. Lastly, electron density analysis, also employed in the manuscript, establishes the peptide's distance from the membrane center at 13.5 ± 3.8 Å. (Figure 8). From the electron density distribution of the peptide vs. the bilayer in the final 50 ns, we can see that the peptide is located within the interfacial region, i.e. at the level of the glycerol backbone between the lipid chains and the phosphatidylcholine head groups (place where the black and grey lines in the top graph intersect).

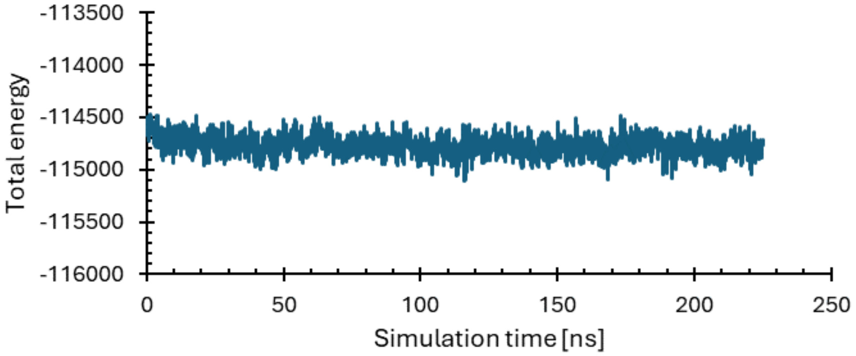

Figure 1: Total energy of the simulation as a function of simulation time.

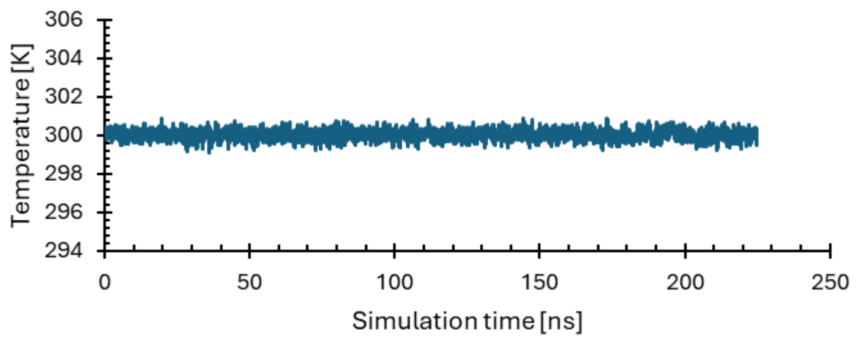

Figure 2: Temperature as a function of simulation time.

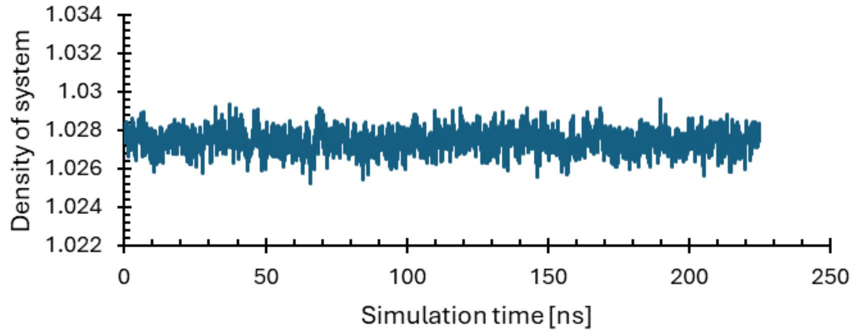

Figure 3: Density of the system as a function of simulation time.

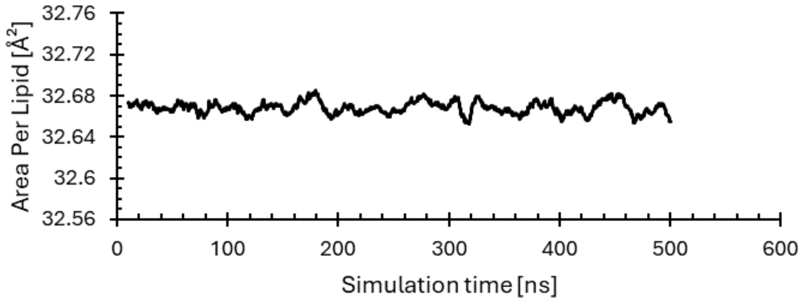

Figure 4: Area per lipid as a function of simulation time.

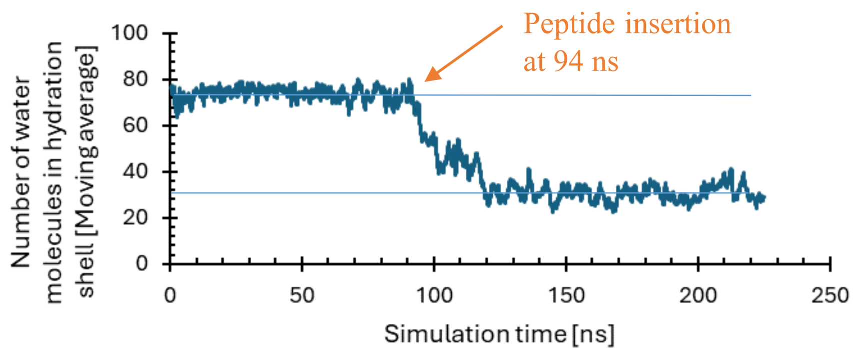

Figure 5: Total number of water molecules surrounding all viscosin alpha carbons within a 5 Å sphere as a function of simulation time.

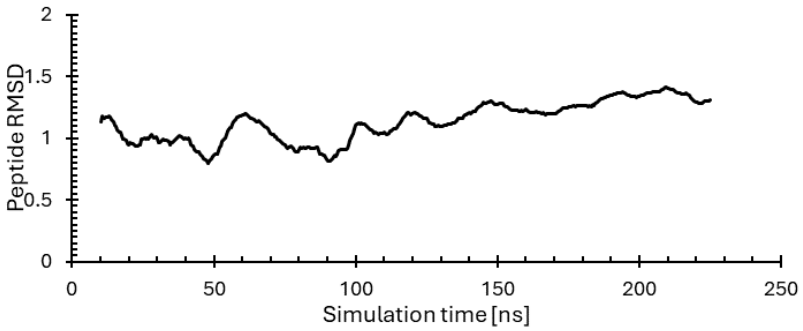

Figure 6: Heavy atom Root-Mean-Square-Deviation (RMSD) of the complete lipopeptide as a function of simulation time with respect to the initial structure.

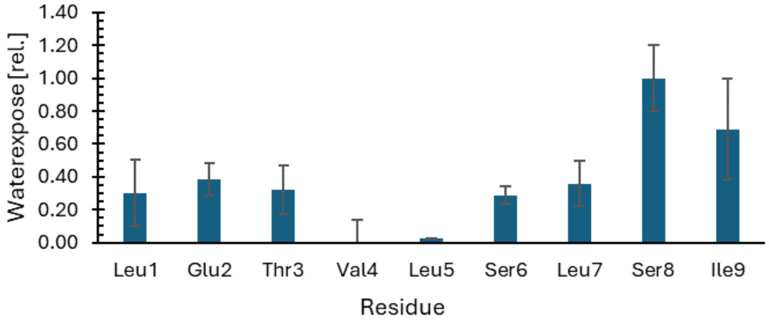

Figure 7: Normalized water exposure of the individual alpha carbons of the lipopeptide .

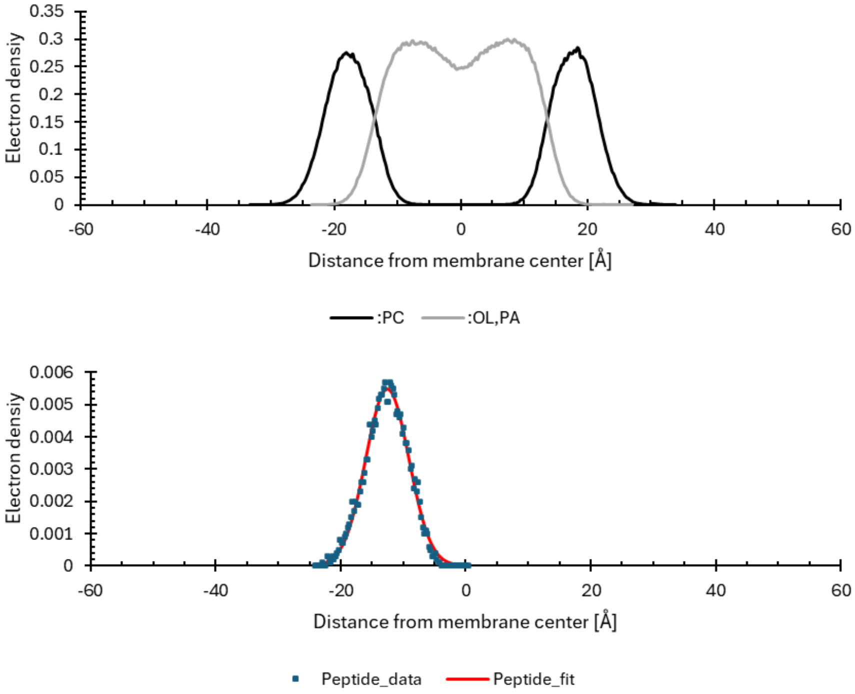

Figure 8: Electron density along the z axis of the simulation box (averaged over the final 50 ns of the simulation), as a function of distance from membrane center. Top: electron density of the phosphocholine (PC) and oleoyl- and palmitoyl (OL, PA) aliphatic chains of the POPC bilayer. Bottom: Electron density calculated for of the peptide (datapoints) and it's fitted Gaussian function (red curve).

Snapshots of the MD trajectory illustrating the system at t = 0 and at 10 ns interval between 90 ns and 130 ns during insertion of viscosin into to bilayer:

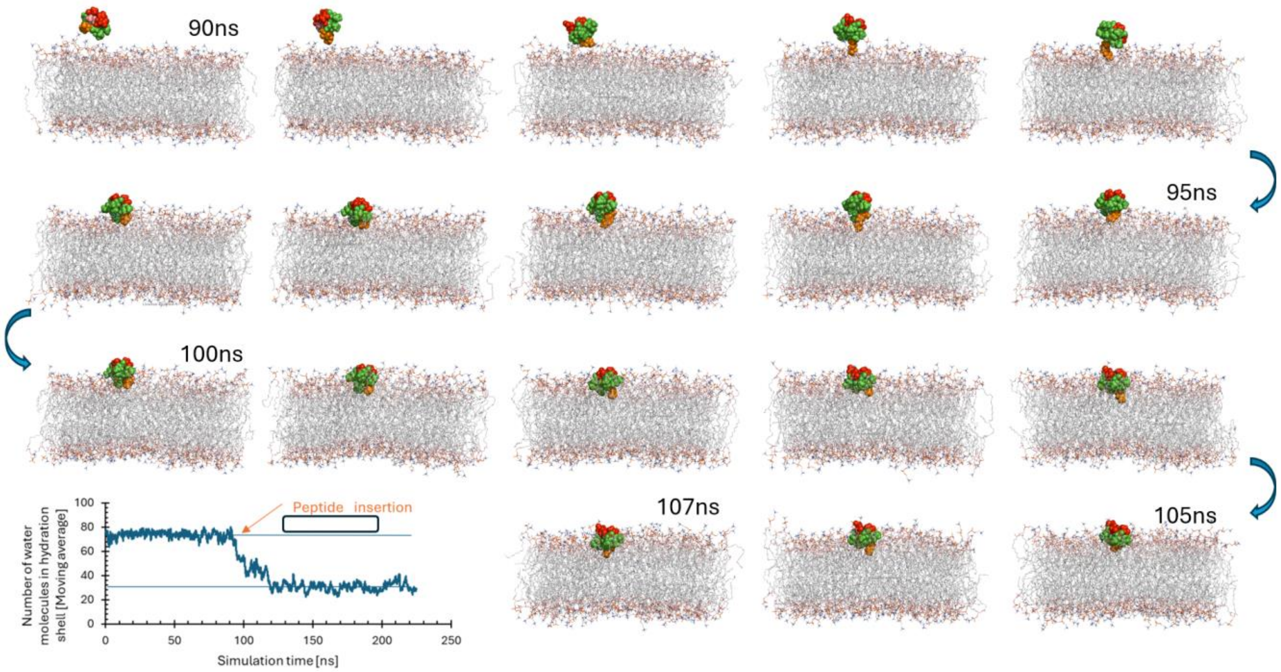

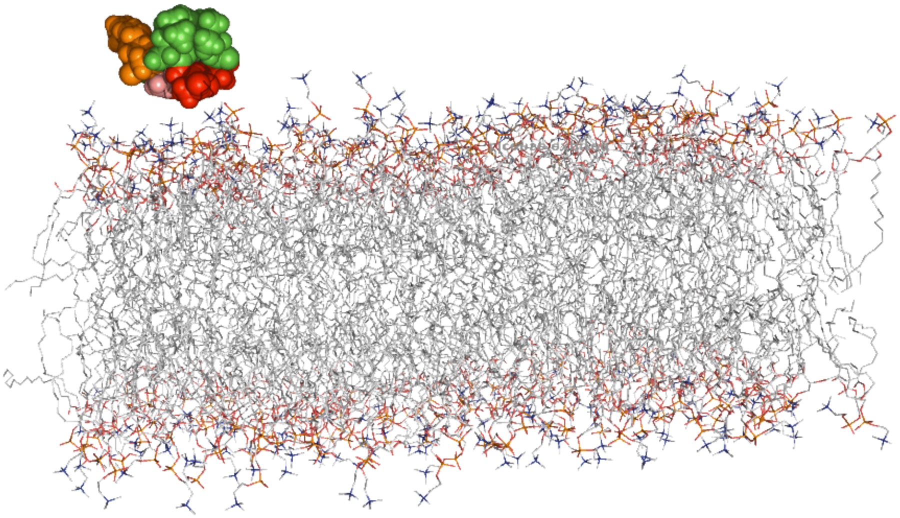

MD simulation of 'wild-type' viscosin, starting at the membrane interface

A second MD simulation was performed for wild-type viscosin positioned at the interface between the aqueous and membrane phases, i.e. with the molecular center at the level of the phosphatidylcholine head group. This positioning was also used for the four tryptophan analogues as described in the manuscript. To sample the evolution in the disposition with respect to the POPC bilayer, the simulation

duration was extended to 500 ns. As before, figures 9-11 demonstrate stability of total energy, temperature, and density over the trajectory. Here again, the "area per lipid" served as a metric for assessing membrane stability and convergence, remaining stable throughout the simulation (Figure 12). From the electron density distribution of the peptide vs. the bilayer, we can see that here also, the peptide is seen to be located within the interfacial region, i.e. at the level of the glycerol backbone between the lipid chains and the phosphatidylcholine head groups (place where the black and grey lines in the top graph intersect). Electron density analysis establishes the peptide's distance from the membrane center at 15.2 ± 3.4 Å (Figure 16). The number of water molecules hydrating the alpha carbons mirrored the results from the previous simulation (section 2.1) starting with viscosin in the aqueous phase (Figure 13). The RMSD of the lipopeptide also shows a constant fluctuation around the same value throughout the simulation, as before (Figure 14). Furthermore, quantitative analysis of hydration around each residue's alpha carbon reiterated that viscosin is surface-bound on the membrane bilayer (Figure 15), with Val4 most shielded from the aqueous phase, consistent with the simulation where viscosin was initially placed in the aqueous phase.

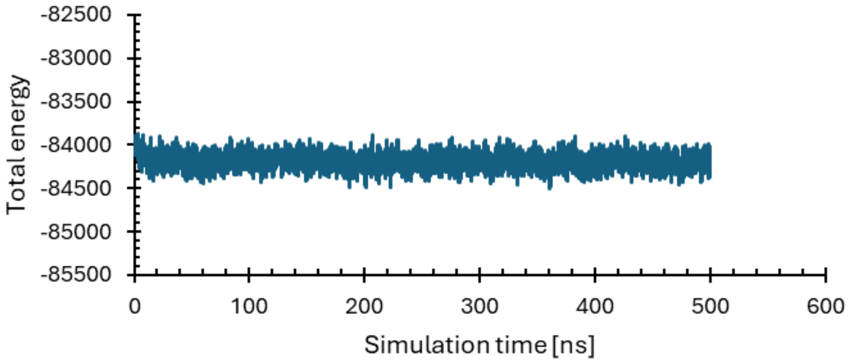

Figure 9: Total energy of the simulation, as a function of simulation time.

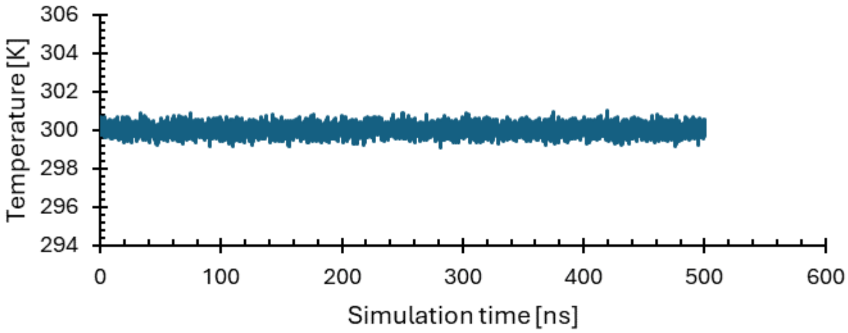

Figure 10: Temperature as a function of simulation time.

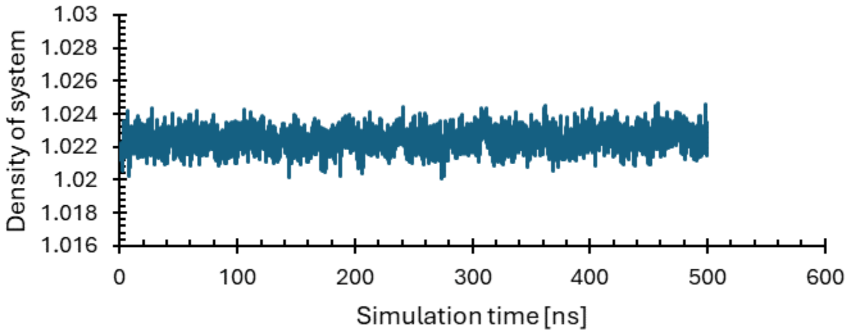

Figure 11: Density of the system as a function of simulation time.

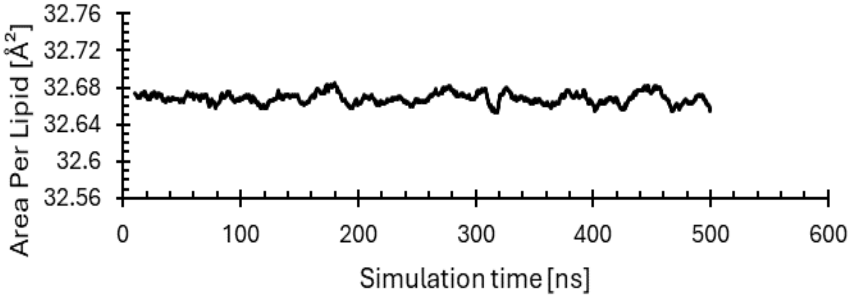

Figure 12: Area per lipid as a function of simulation time.

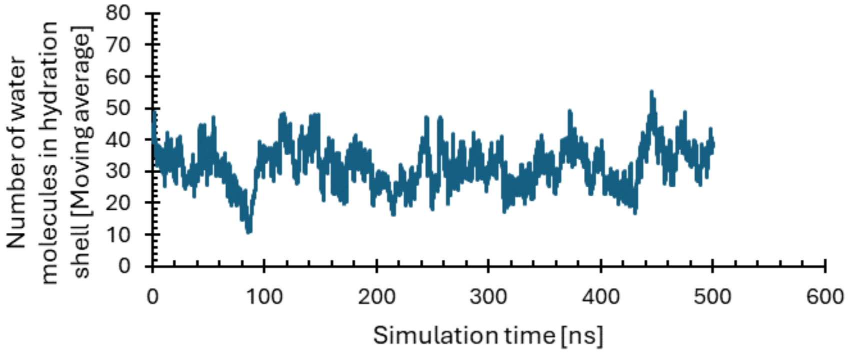

Figure 13: Total number of water molecules surrounding all viscosin alpha carbons within a 5 Å sphere as a function of simulation time

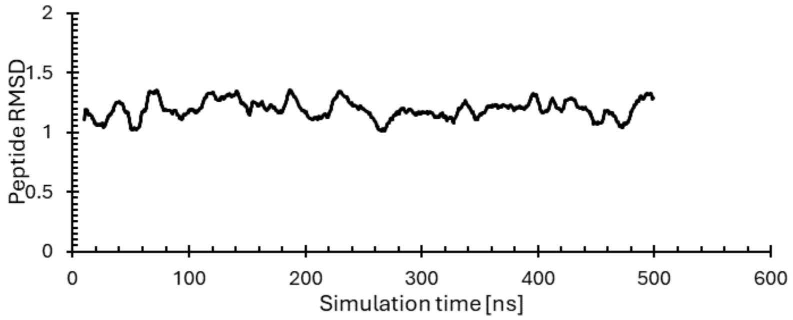

Figure 14: Heavy atom Root-Mean-Square-Deviation (RMSD) of the lipopeptide as a function of simulation time.

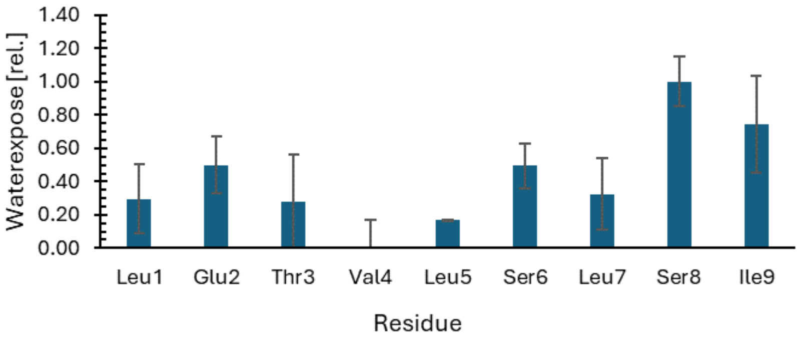

Figure 15: Normalized water exposure of the individual alpha carbons of the lipopeptide.

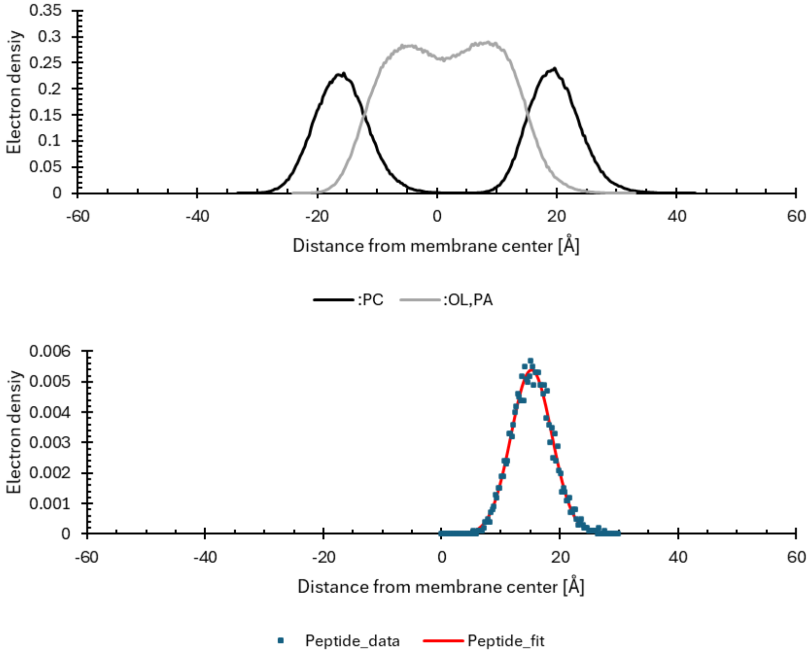

Figure 16: Electron density along the z axis of the simulation box (averaged over the final 50 ns of the simulation), as a function of distance from membrane center. Top: electron density of the phosphocholine (:PC) and oleoyl- and palmitoyl (:OL,:PA) aliphatic chains of the membrane bilayer. Bottom: Electron density of the peptide (datapoints) and it's fitted Gaussian function (red curve).

13. Impact of the starting location and orientation of viscosin within the bilayer

All simulations in this section were performed using the same protocol, the only difference being the initial location (3.1-3.3) and orientation (3.4) with respect to the bilayer. These allow to assess the time required for viscosin to equilibrate towards a final position and orientation within the membrane, and provides further support for limiting MD simulations of W-viscosins to 100 ns timescales, focusing on the final 50 ns for trajectory analysis. Given the number of simulations performed, and the outcome, 50 ns appeared sufficient to provide the required input for addressing simulation concerns with respect to the location of the lipopeptide. For reorientation concerns, we used a 150 ns simulation time was used. We show that the impact of the initial state, kinetic trapping of structures and reorientational time scales of viscosins within the solvated and pre-equilibrated POPC bilayer should be no cause for concern when 100 ns simulation times, such as used in the W-viscosin simulations, are applied.

50 ns MD simulation of viscosin initially at bilayer-water interface (phosphatidylcholine head group)

In the first of three simulations, the lipopeptide is once again positioned at the interface between the aqueous and membrane phases, i.e. with the molecular center positioned at the level of the phosphatidylcholine head group. This parallels the initial orientation of the W-derivatives in the manuscript. Results closely resemble those observed previously in 2.2. Figures 17-19 demonstrate the stability of this simulation over time. We tracked total energy, temperature, and density to ensure consistent behavior. The "area per lipid" was again employed as a metric for assessing membrane stability and convergence, maintaining stability throughout the simulation (Figure 20). In this simulation, the peptide positions itself within the interfacial region, similar to previous simulations. The number of water molecules hydrating the peptides' alpha carbons remains consistent with previous findings (Figure 21). The RMSD of the lipopeptide also remains consistent throughout the simulation (Figure 22). Additionally, quantitative analysis of hydration around each residue's alpha carbon once again confirms that viscosin is bound to the membrane bilayer surface, with Val4 most shielded from the aqueous phase (Figure 23). Lastly, electron density analysis establishes the peptide's distance from the membrane center at 13.5 ± 3.6 Å (Figure 24). Figure 25 provides a visual representation of viscosin's location at the beginning and end of the MD simulation.

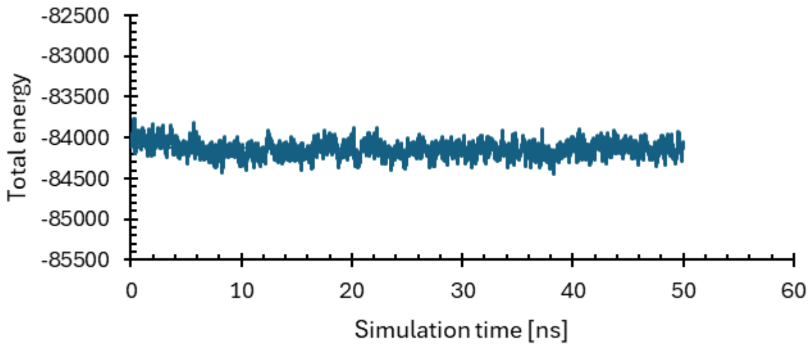

Figure 17: Total energy of the simulation, as a function of simulation time.

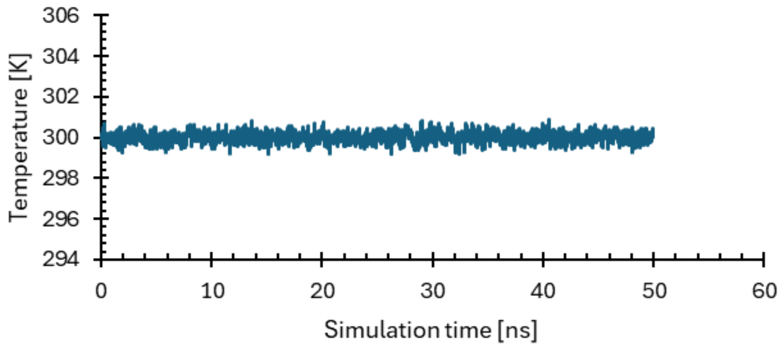

Figure 18: Temperature as a function of simulation time.

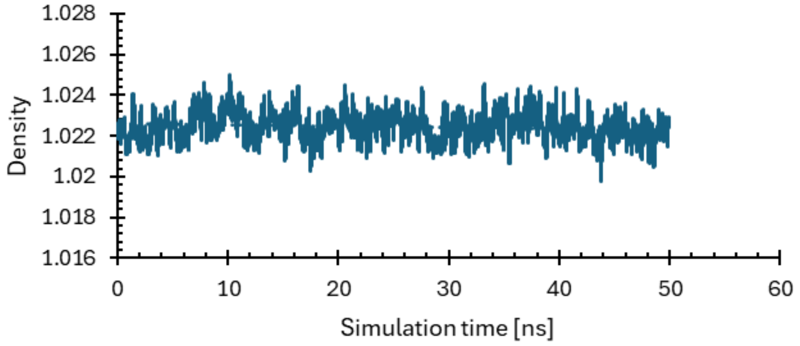

Figure 19: Density of the system as a function of simulation time.

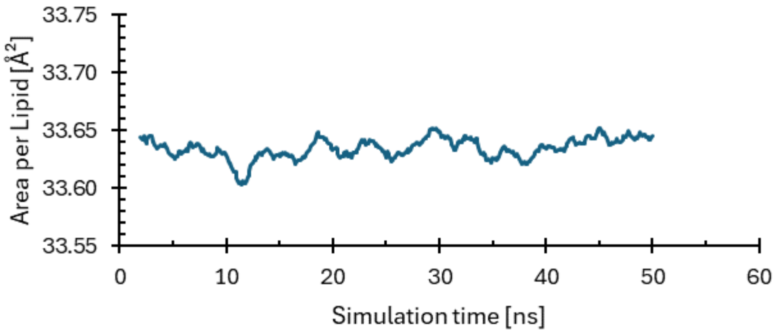

Figure 20: Area per lipid as a function of simulation time.

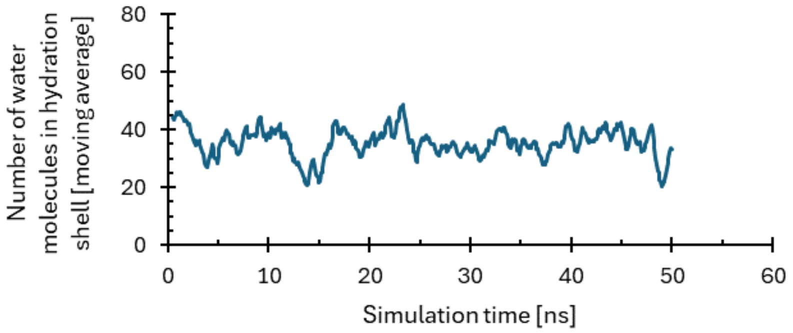

Figure 21: Total number of water molecules surrounding all viscosin alpha carbons within a 5 Å sphere as a function of simulation time.

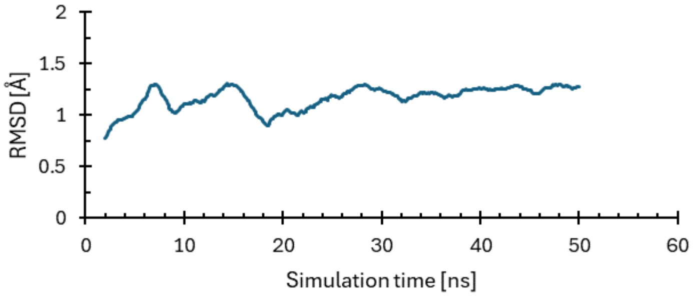

Figure 22: Heavy atom Root-Mean-Square-Deviation (RMSD) of the lipopeptide as a function of simulation time.

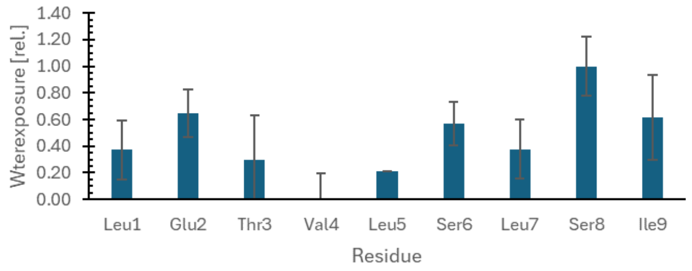

Figure 23: Total number of water molecules surrounding all viscosin alpha carbons within a 5 Å sphere as a function of simulation time.

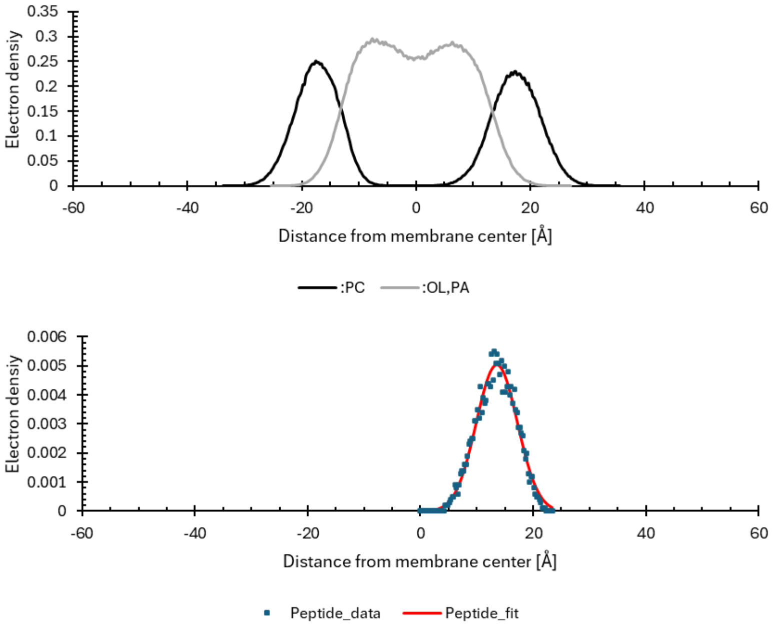

Figure 24: Electron density along the z axis of the simulation box (averaged over the final 10 ns of the simulation), as a function of distance from membrane center. Top: electron density of the phosphocholine (:PC) and oleoyl- and palmitoyl (:OL,:PA) aliphatic chains of the membrane bilayer. Bottom: Electron density of the peptide (datapoints) and it's fitted Gaussian function (red curve).

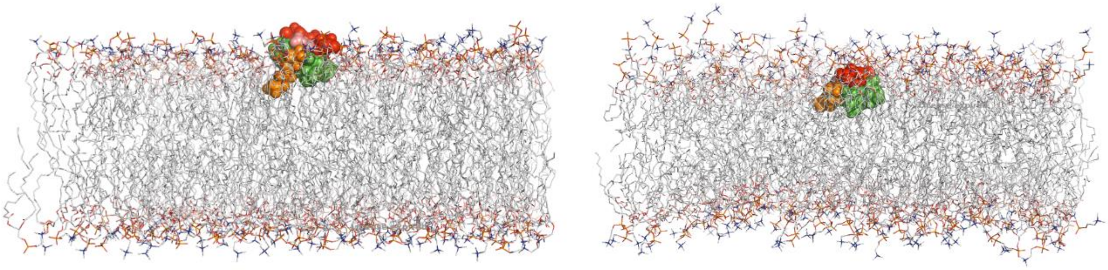

Figure 25: Snapshot of the simulation box at the start (left) and end (right) of the MD simulation.

50 ns MD simulation of viscosin initially at the interfacial region (level of the glycerol backbone)

In the second of three simulations, the lipopeptide is positioned between the polar head groups and the aliphatic tails of the POPC bilayer, i.e. with its center at the level of the glycerol backbone. Figures 26-28 depict the stability of this simulation over time. We monitored total energy, temperature, and density to ensure consistent behavior. Once again, the "area per lipid" served as a metric for evaluating membrane stability and convergence, maintaining stability throughout the simulation (Figure 29). In this simulation, the peptide positions itself within the interfacial region, similar to previous simulations. The number of water molecules hydrating the alpha carbons remains consistent with previous findings (Figure 30). The RMSD of the lipopeptide also remains stable throughout the simulation (Figure 31). Additionally, quantitative analysis of hydration around each residue's alpha carbon once again confirms that viscosin is bound to the membrane bilayer surface, with Val4 most shielded from the aqueous phase (Figure 32). Finally, electron density analysis, as employed in the manuscript, establishes the peptide's distance from the membrane center at 15.3 ± 3.4 Å (Figure 33). Figure 34 provides a visual depiction of viscosin's location at the beginning and end of the MD simulation.

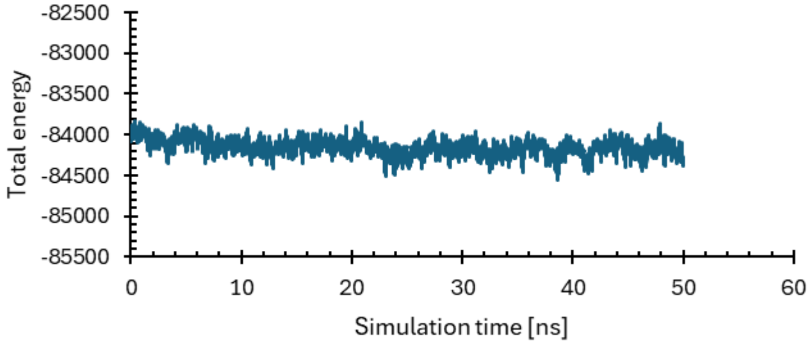

Figure 26: Total energy of the simulation, as a function of simulation time.

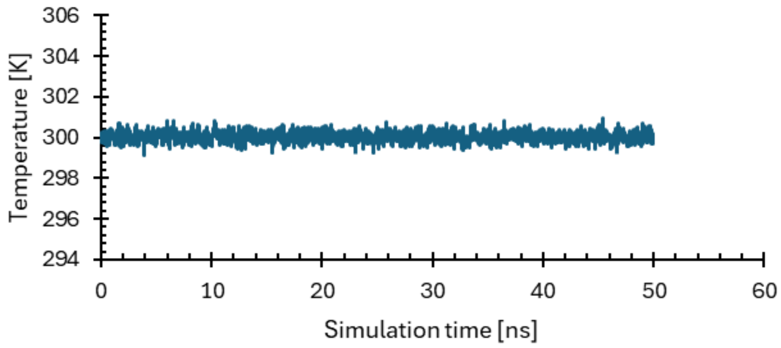

Figure 27: Temperature as a function of simulation time.

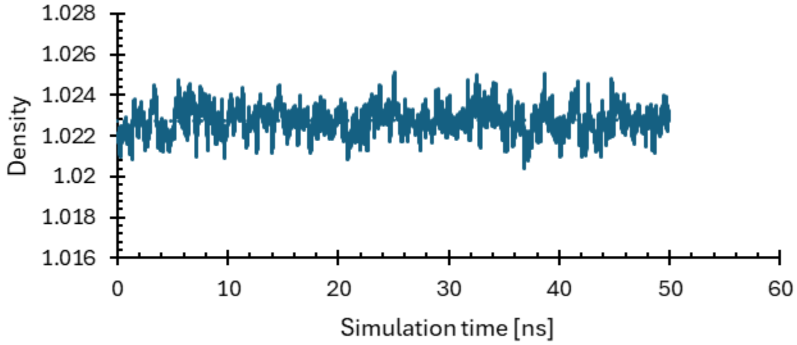

Figure 28: Density of the system as a function of simulation time.

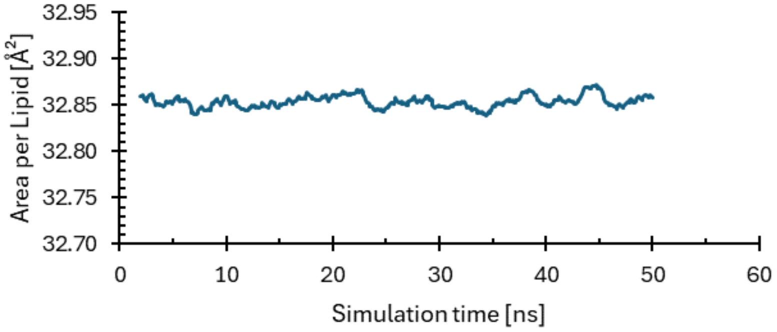

Figure 29: Area per lipid as a function of simulation time.

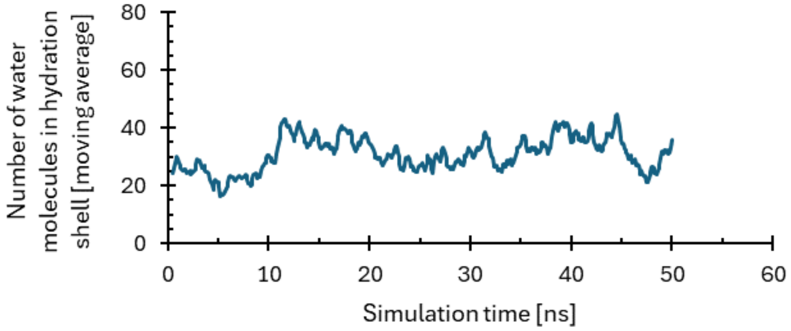

Figure 30: Total number of water molecules surrounding all viscosin alpha carbons within a 5 Å sphere as a function of simulation time.

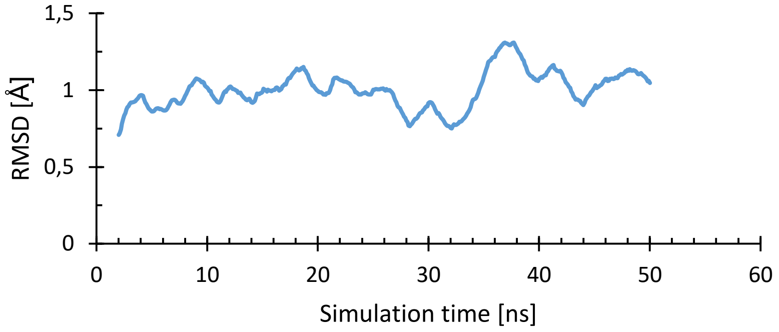

Figure 31: Heavy atom Root-Mean-Square-Deviation (RMSD) of the lipopeptide as a function of simulation time.

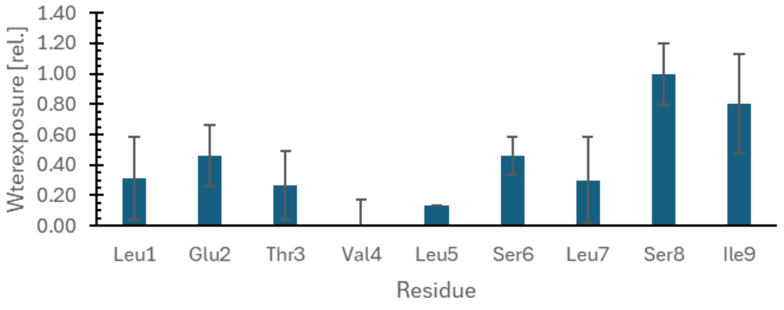

Figure 32: Normalized water exposure of the individual alpha carbons of the lipopeptide.

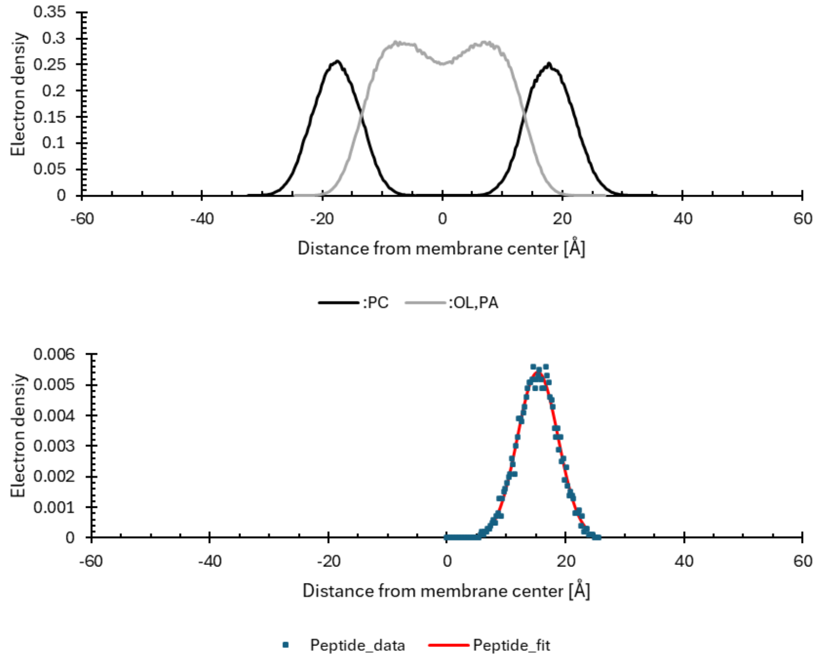

Figure 33: Electron density along the z axis of the simulation box (averaged over the final 10 ns of the simulation), as a function of distance from membrane center. Top: electron density of the phosphocholine (:PC) and oleoyl- and palmitoyl (:OL,:PA) aliphatic chains of the membrane bilayer. Bottom: Electron density of the peptide (datapoints) and it's fitted Gaussian function (red curve).

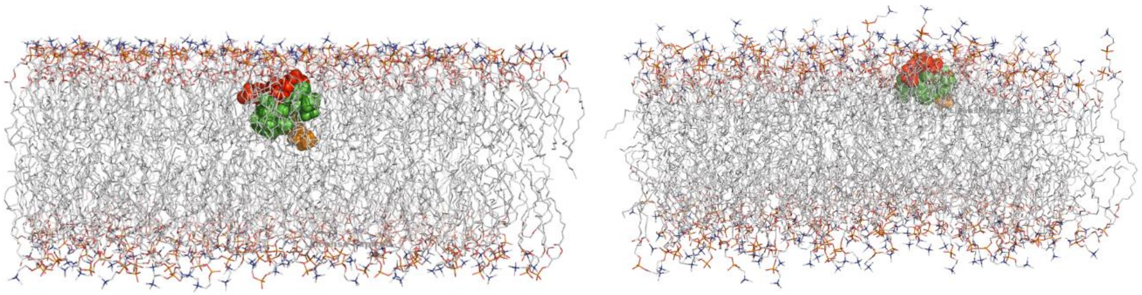

Figure 34: Snapshot of the simulation box at the start (left) and end (right) of the MD simulation.

50 ns MD simulation of viscosin initially located at the center of the bilayer

In the third simulation, the lipopeptide is placed with the molecular center in the middle of the hydrophobic core of the POPC bilayer. Figures 35-37 provide insight into the stability of this simulation over time. We closely monitored total energy, temperature, and density to ensure consistent behavior. Once again, the "area per lipid" served as a key indicator for evaluating membrane stability and convergence, maintaining stability throughout the simulation (Figure 38 In this simulation, the peptide positions itself within the interfacial region, similar to previous simulations. The number of water molecules surrounding the lipopeptide's alpha carbons remains consistent with previous observations (Figure 39). Similarly, the RMSD of the lipopeptide exhibits stability throughout the simulation (Figure 40). Additionally, quantitative analysis of hydration around each residue's alpha carbon reaffirms that viscosin is firmly located close to the membrane bilayer surface, with Val4 predominantly shielded from the aqueous phase (Figure 41). Finally, electron density analysis, as utilized in the manuscript, determines the peptide's distance from the membrane center to be at 14.2 ± 2.8 Å (Figure 42). Figure 43 visually depicts viscosin's position at the beginning and end of the MD simulation.

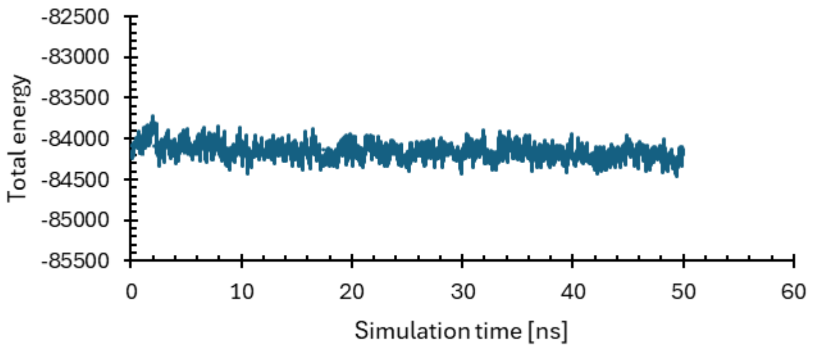

Figure 35: Total energy of the simulation, as a function of simulation time.

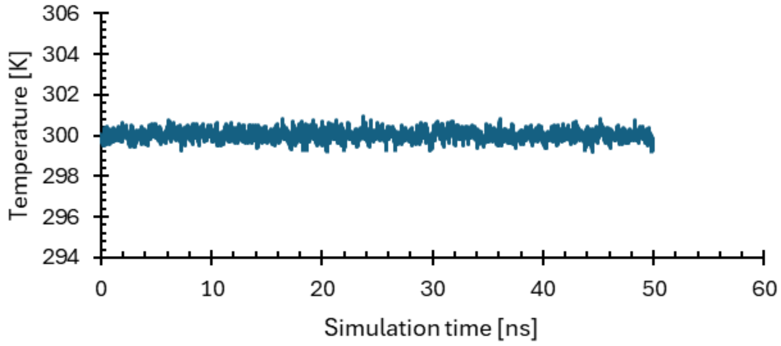

Figure 36: Temperature as a function of simulation time.

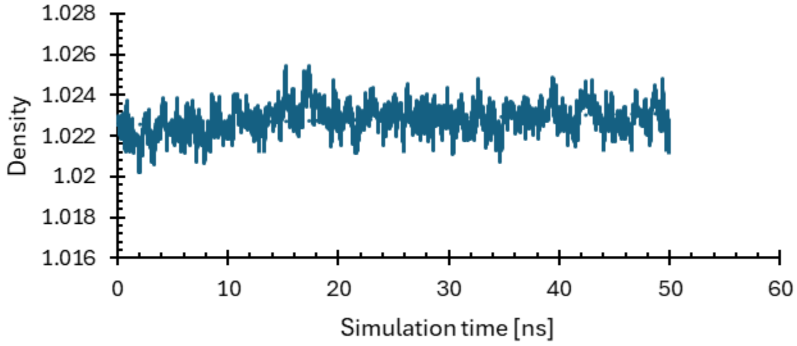

Figure 37: Density of the system as a function of simulation time.

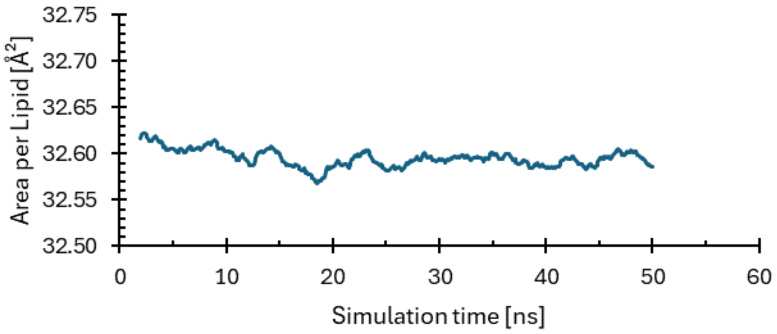

Figure 38: Area per lipid as a function of simulation time.

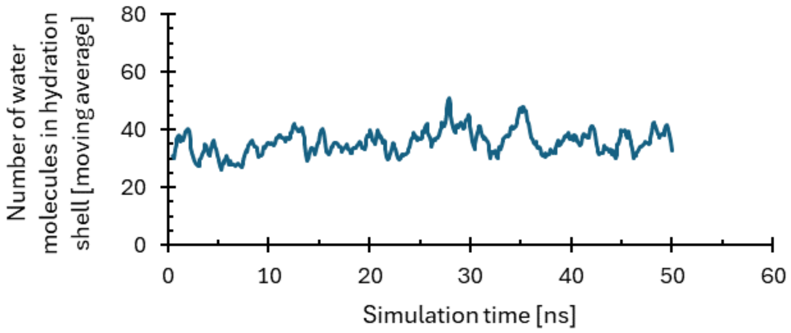

Figure 39: Total number of water molecules surrounding all viscosin alpha carbons within a 5 Å sphere as a function of simulation time.

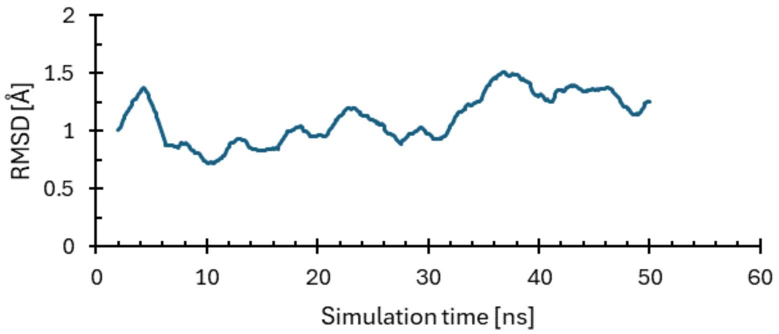

Figure 40: Heavy atom Root-Mean-Square-Deviation (RMSD) of the lipopeptide as a function of simulation time.

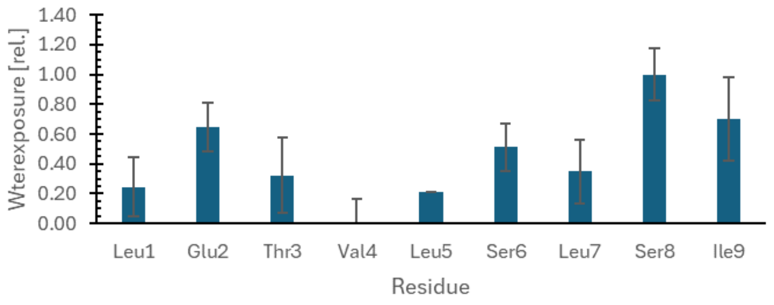

Figure 41: Normalized water exposure of the individual alpha carbons of the lipopeptide.

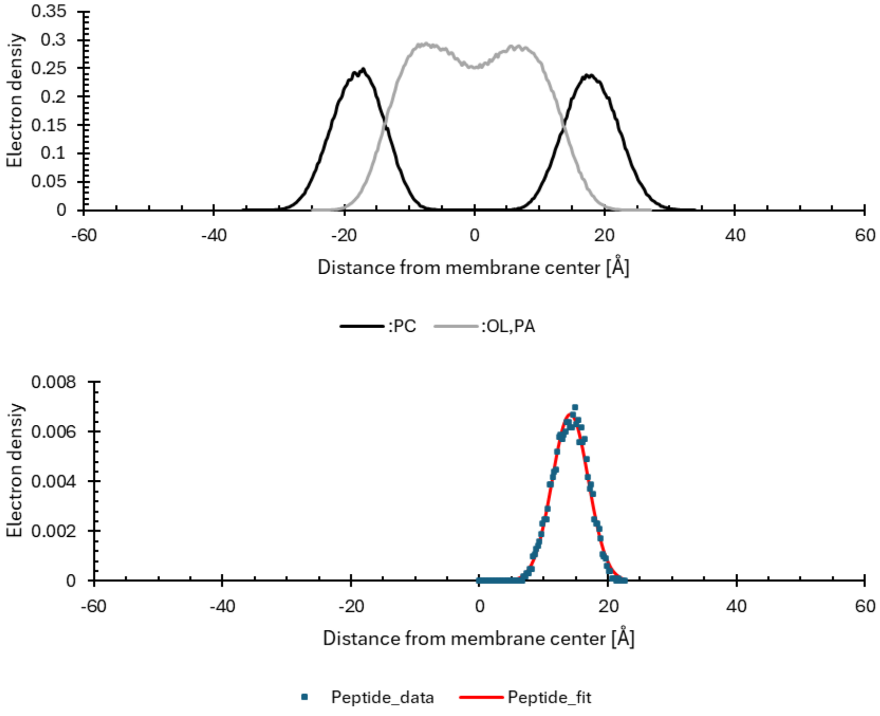

Figure 42: Electron density along the z axis of the simulation box (averaged over the final 10 ns of the simulation), as a function of distance from membrane center. Top: electron density of the phosphocholine (:PC) and oleoyl- and palmitoyl (:OL,:PA) aliphatic chains of the membrane bilayer. Bottom: Electron density of the peptide (datapoints) and it's fitted Gaussian function (red curve).

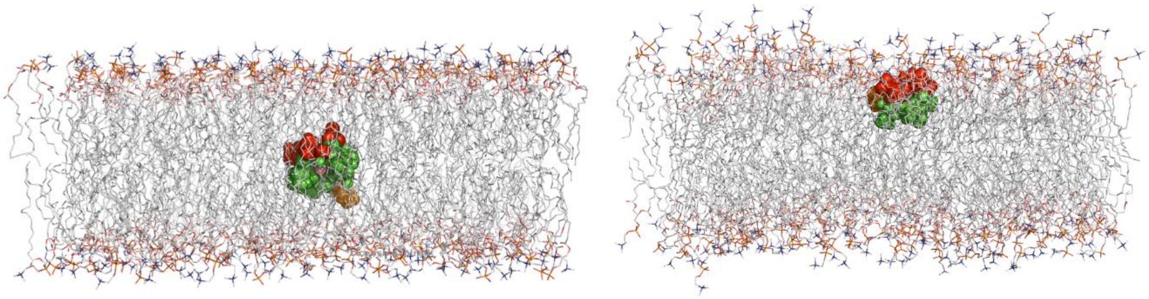

Figure 43: Snapshot of the simulation box at the start (left) and end (right) of the MD simulation.

150 ns MD simulation of viscosin oriented upside down at the bilayer-water interface.

In this simulation, the lipopeptide is again positioned between the polar head groups and the aliphatic tails of the POPC bilayer i.e. with its center at the level of the glycerol backbone (similar as simulation 3.2). Additionally, the lipopeptide is rotated 180°, such that the hydrophilic face of the lipopeptide is now facing the hydrophobic center of the membrane bilayer. As it can be expected reorientation to the normal amphipathic interaction may require more time, the simulation was performed over 150 ns. Figures 44-46 provide insight into the stability of this simulation over time. We closely monitored total energy, temperature, and density to ensure consistent behavior. Once again, the "area per lipid" served as a key indicator for evaluating membrane stability and convergence, maintaining stability throughout the simulation (Figure 47). In this simulation, the peptide positions itself within the interfacial region, similar to previous simulations. The number of water molecules surrounding the lipopeptide's alpha carbons remains consistent with previous observations (Figure 48). Similarly, the RMSD of the lipopeptide exhibits stability throughout the simulation (Figure 49). Additionally, quantitative analysis of hydration around each residue's alpha carbon reaffirms that viscosin is firmly positioned at the membrane bilayer surface, with Val4 predominantly shielded from the aqueous phase (Figure 50). Consequently, starting with the lipopeptide rotated 180°, reorientation is complete after 80 ns. The final orientation matches very well with those observed before. Finally, electron density analysis, as utilized in the manuscript, determines the peptide's distance from the membrane center to be at 16.4 ± 3.4 Å (Figure 51). Figure 52 visually depicts viscosin's position at the beginning and end of the MD simulation.

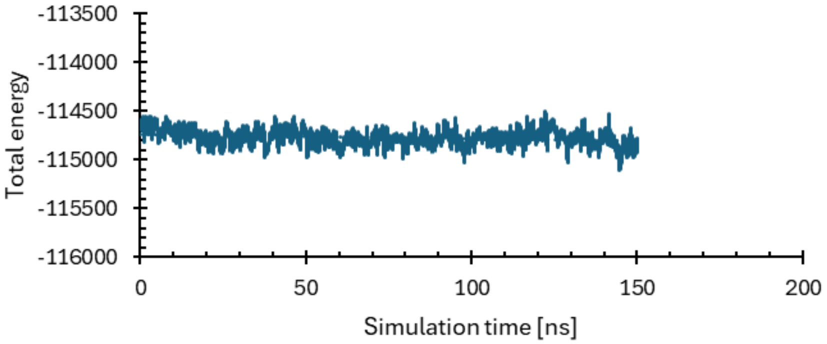

Figure 44: Total energy of the simulation, as a function of simulation time.

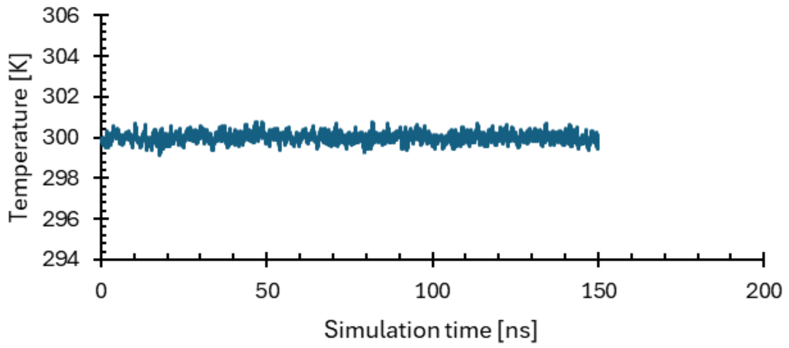

Figure 45: Temperature as a function of simulation time.

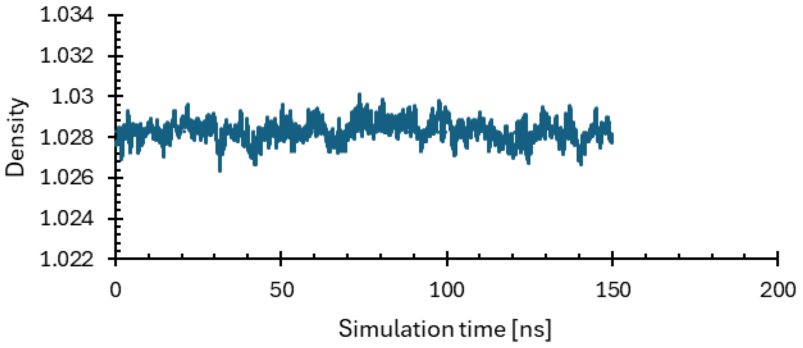

Figure 46: Density of the system as a function of simulation time.

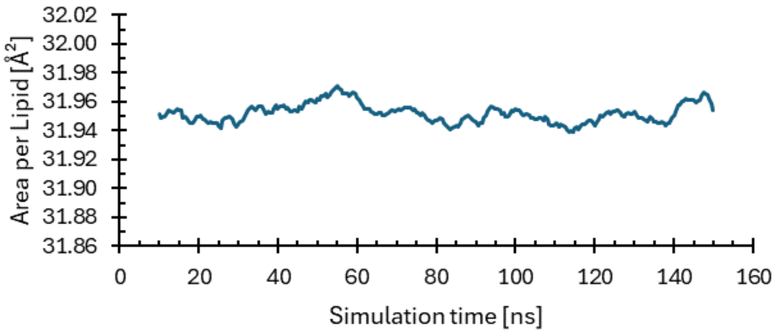

Figure 47: Area per lipid as a function of simulation time.

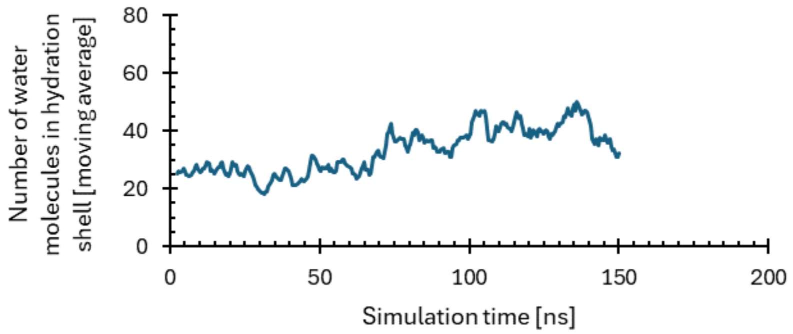

Figure 48: Total number of water molecules surrounding all viscosin alpha carbons within a 5 Å sphere as a function of simulation time.

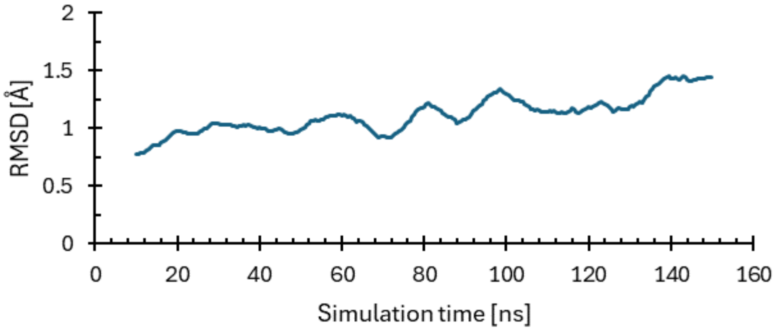

Figure 49: Heavy atom Root-Mean-Square-Deviation (RMSD) of the lipopeptide as a function of simulation time.

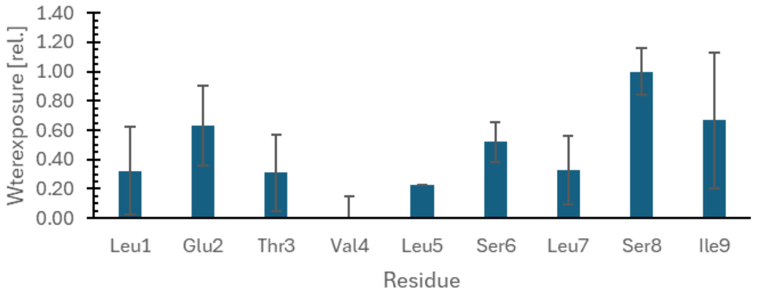

Figure 50: Normalized water exposure of the individual alpha carbons of the lipopeptide.

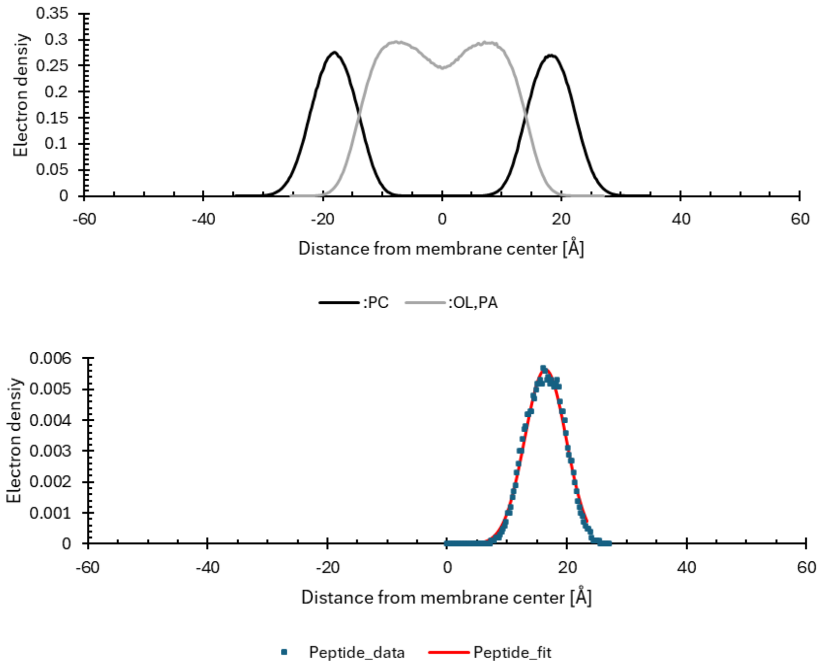

Figure 51: Electron density along the z axis of the simulation box (averaged over the final 50 ns of the simulation), as a function of distance from membrane center. Top: electron density of the phosphocholine (:PC) and oleoyl- and palmitoyl (:OL,:PA) aliphatic chains of the membrane bilayer. Bottom: Electron density of the peptide (datapoints) and it's fitted Gaussian function (red curve).

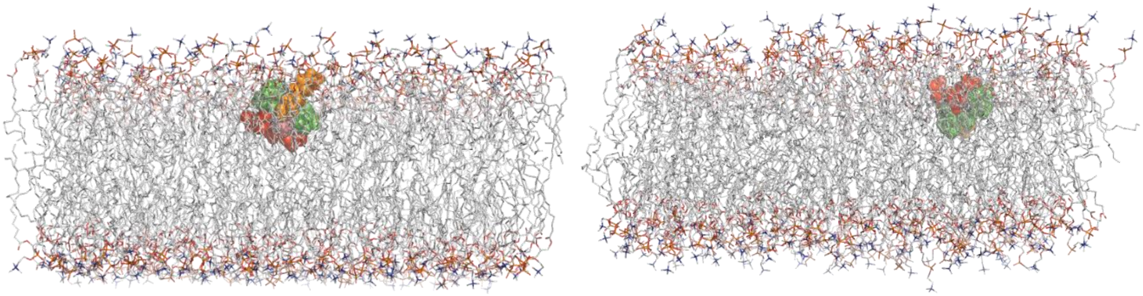

Figure 52: Snapshot of the simulation box at the start (left) and end (right) of the MD simulation.

14. MD simulations of the tryptophan-labelled lipopeptides

Lastly, we offer supplementary data regarding the MD simulations of the tryptophan-labeled lipopeptides, as included in the manuscript. The coordinates for all starting structures (including that of wild-type viscosin) and associated simulation input files are available from a GitHub repository (https://github.com/nmrstruc/BiophysJ-W-viscosins).

MD simulation of L1W

We closely monitored the total energy, temperature, and density of the MD simulations for the L1W-labelled viscosin system to ensure consistent behavior (Figures 53-55). The "area per lipid" served as a metric for evaluating membrane stability and convergence, maintaining stability throughout the simulation (Figure 56). In this simulation, the peptide positions itself within the interfacial region (Figure 57), similar to previous simulations. The number of water molecules surrounding the alpha carbons remained consistent with previous findings (Figure 58). Similarly, the RMSD of the lipopeptide exhibited stability throughout the simulation (Figure 59). Finally, quantitative analysis of hydration around each residue's alpha carbon reaffirmed that viscosin remained surface-bound on the membrane bilayer, with Val4 most shielded from the aqueous phase (Figure 60).

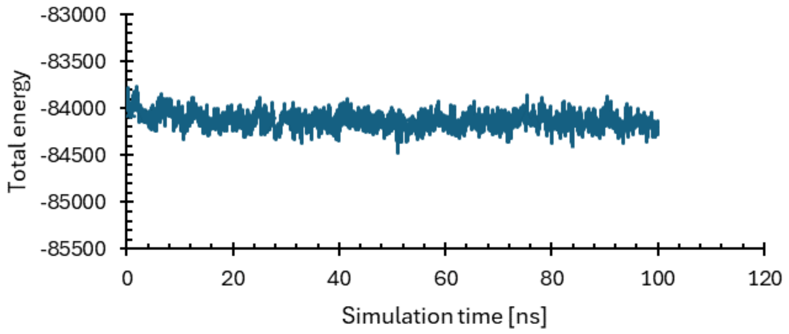

Figure 53: Total energy of the simulation, as a function of simulation time.

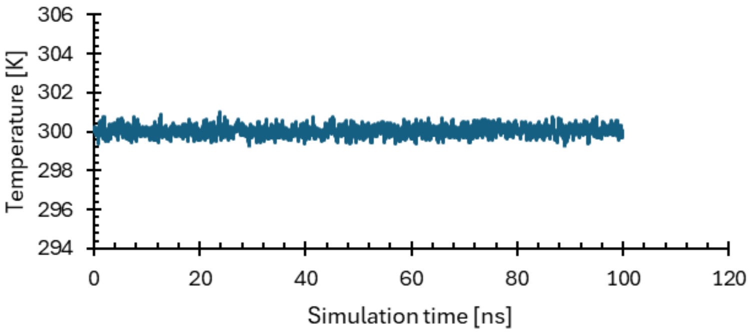

Figure 54: Temperature as a function of simulation time.

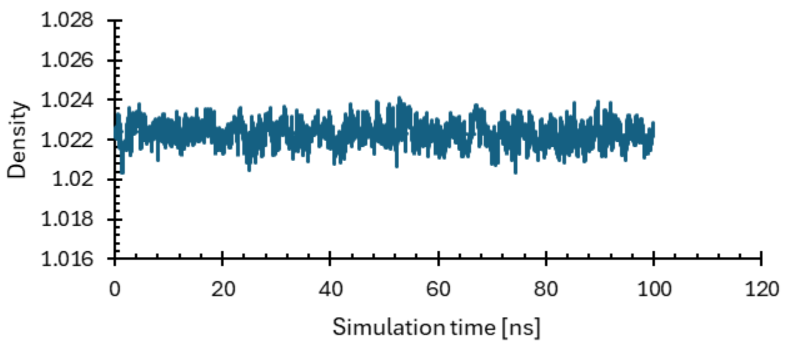

Figure 55: Density of the system as a function of simulation time.

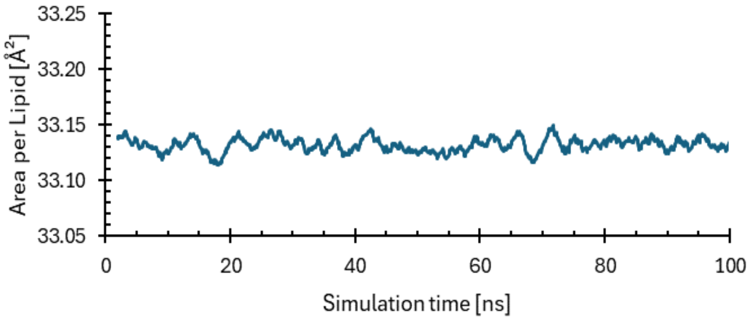

Figure 56: Area per lipid as a function of simulation time.

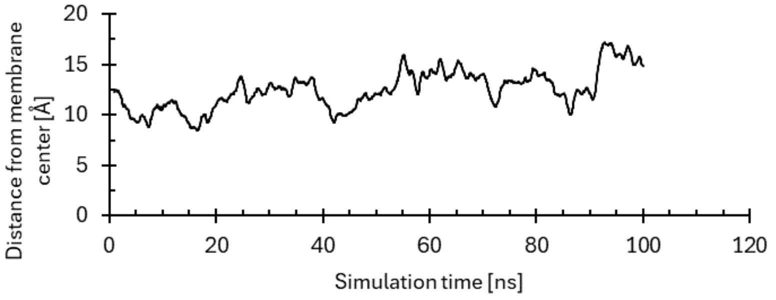

Figure 57: Distance of lipopeptide center of mass from the membrane center, as a function of simulation time.

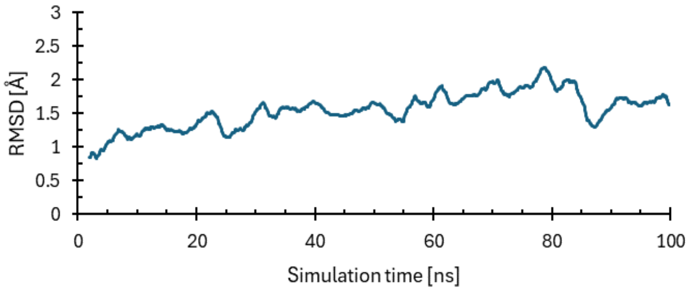

Figure 58: Heavy atom Root-Mean-Square-Deviation (RMSD) of the lipopeptide as a function of simulation time.

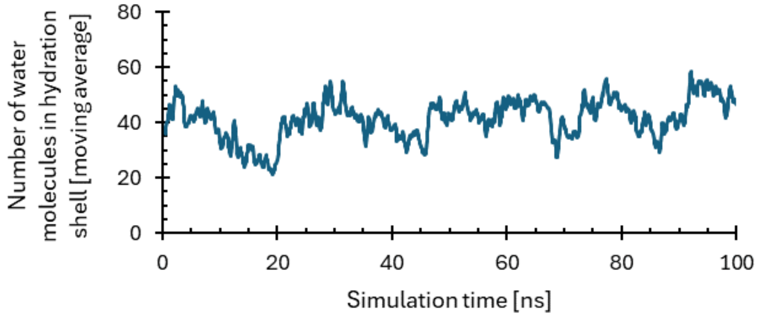

Figure 59: Total number of water molecules surrounding all viscosin alpha carbons within a 5 Å sphere as a function of simulation time.

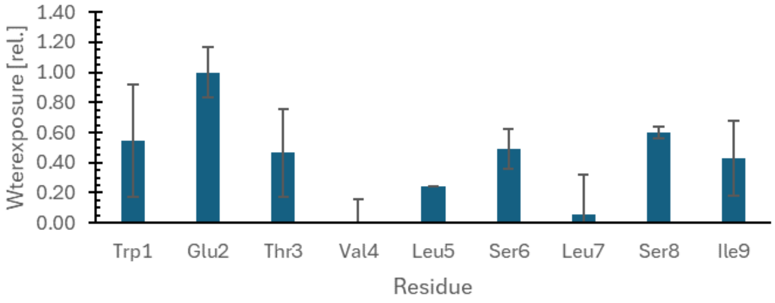

Figure 60: Normalized water exposure of the individual alpha carbons of the lipopeptide.

MD simulation of V_4W

We monitored total energy, temperature, and density of the MD simulations of the V4W-labelled viscosin system to ensure consistent behavior (Figures 61-63). The 'area per lipid' is used as a metric for assessing membrane stability and convergence, remaining stable throughout the simulation (Figure 64). In this simulation, the peptide positions itself within the interfacial region (*Figure 65*), similar to previous simulations. The number of water molecules hydrating the alpha carbons is similar to before. (Figure 67). The RMSD of the lipopeptide also remains stable throughout the simulation (Figure 66). Finally, quantitative analysis of hydration around each residues' alpha carbon again indicates that viscosin is surface-bound on the membrane bilayer, with the fourth residue (now Trp4) largely shielded from the aqueous phase (Figure 68).

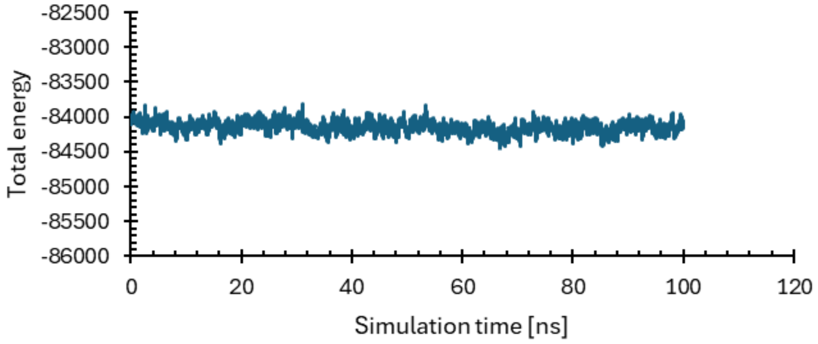

Figure 61: Total energy of the simulation, as a function of simulation time.

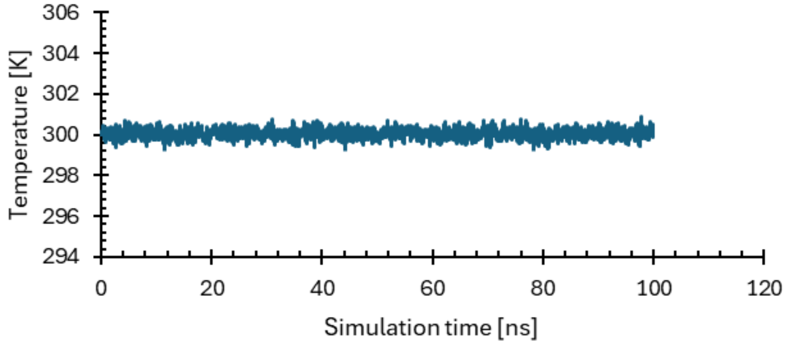

Figure 62: Temperature as a function of simulation time.

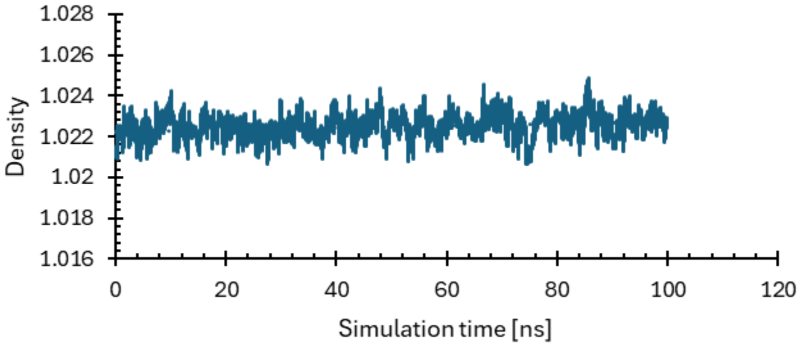

Figure 63: Density of the system as a function of simulation time.

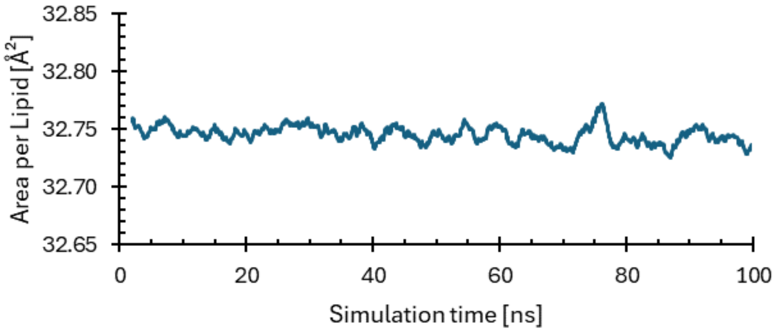

Figure 64: Area per lipid as a function of simulation time.

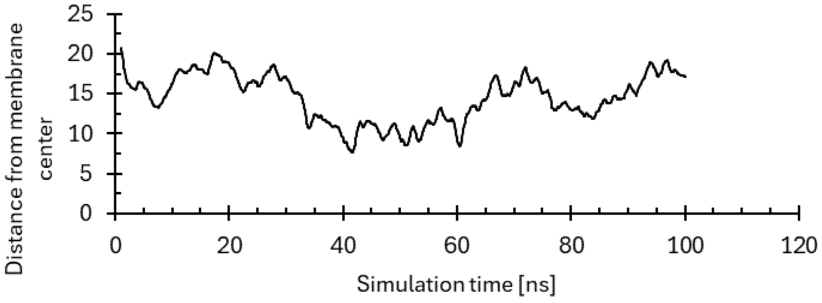

Figure 65: Distance of lipopeptide center of mass from the membrane center, as a function of simulation time.

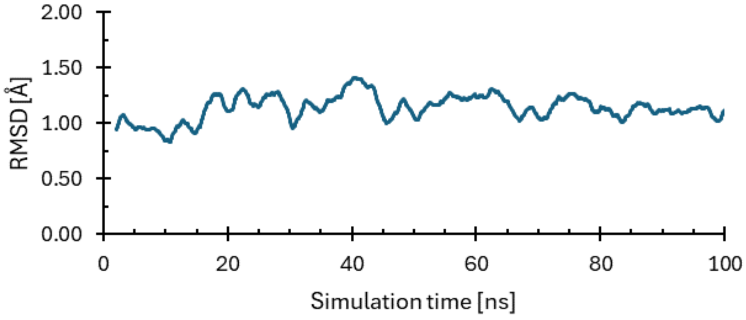

Figure 66: Heavy atom Root-Mean-Square-Deviation (RMSD) of the lipopeptide as a function of simulation time.

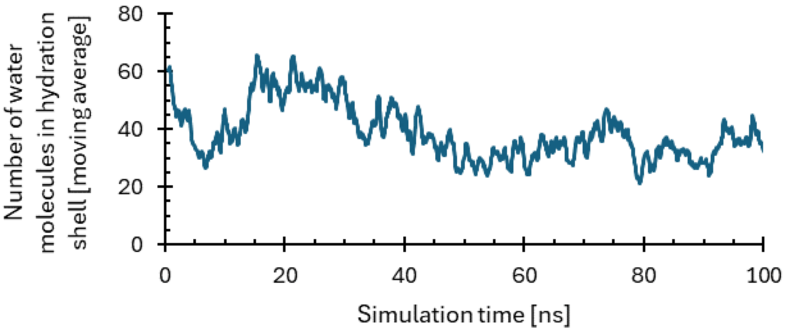

Figure 67: Total number of water molecules surrounding all viscosin alpha carbons within a 5 Å sphere as a function of simulation time.

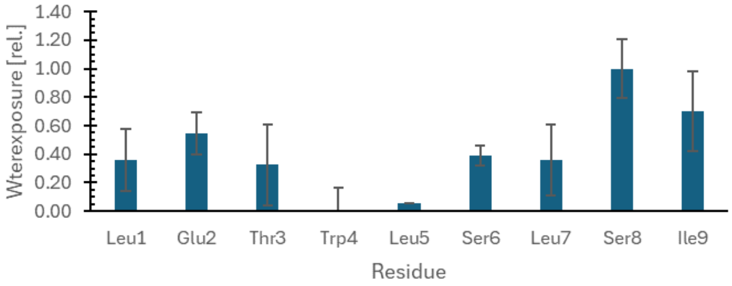

Figure 68: Normalized water exposure of the individual alpha carbons of the lipopeptide, the original peptide sequence is indicated.

MD simulation of L5W

We monitored total energy, temperature, and density of the MD simulations of the L5W-labelled viscosin system to ensure consistent behavior (Figures 69-71). The 'area per lipid' is used as a metric for assessing membrane stability and convergence, remaining stable throughout the simulation (Figure 72). In this simulation, the peptide positions itself within the interfacial region (*Figure 73*), similar to previous simulations. The number of water molecules hydrating the alpha carbons is similar to before. (Figure 74). The RMSD of the lipopeptide also remains stable throughout the simulation (Figure 75). Finally, quantitative analysis of hydration around each residues' alpha carbon again indicates that viscosin is surface-bound on the membrane bilayer, with Val4 largely shielded from the aqueous phase (Figure 76).

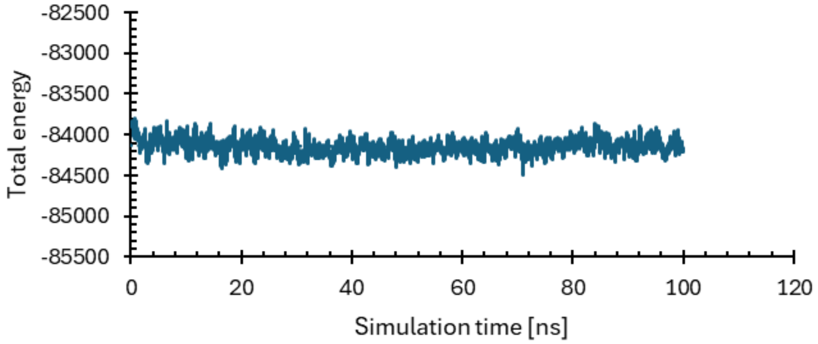

Figure 69: Total energy of the simulation, as a function of simulation time.

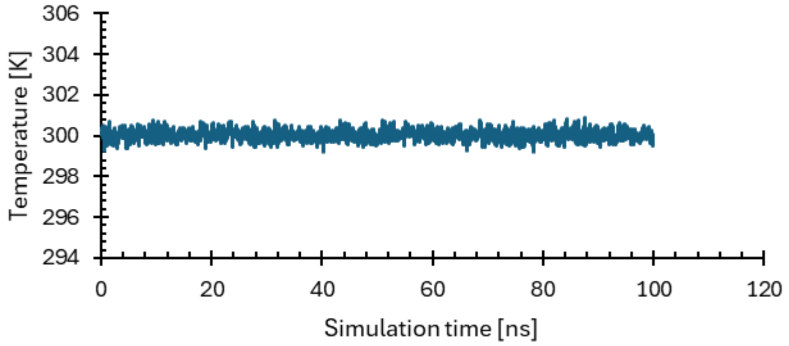

Figure 70: Temperature as a function of simulation time.

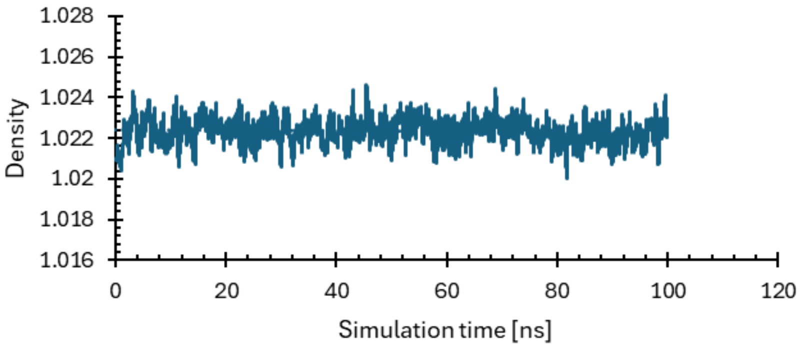

Figure 71: Density of the system as a function of simulation time.

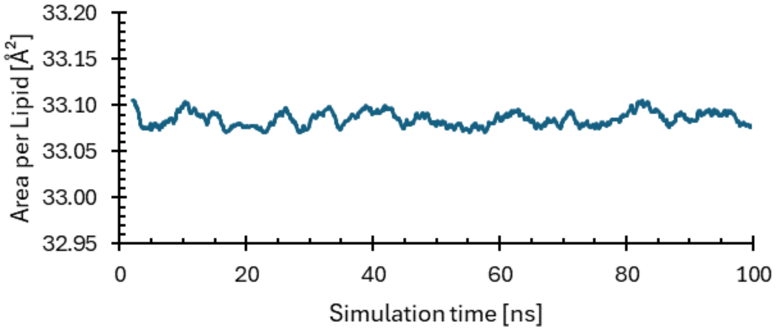

Figure 72: Area per lipid as a function of simulation time.

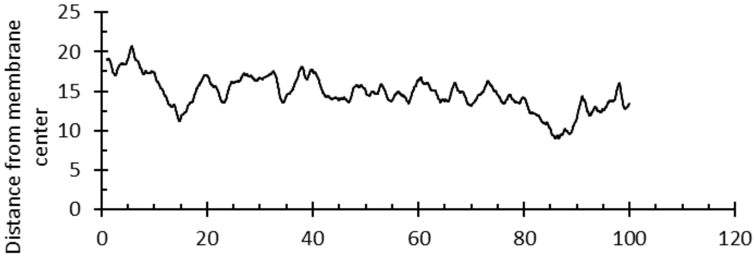

Figure 73: Distance of lipopeptide center of mass from the membrane center, as a function of simulation time.

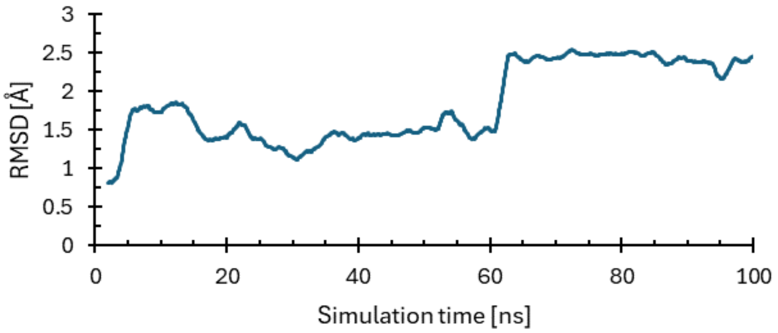

Figure 74: Heavy atom Root-Mean-Square-Deviation (RMSD) of the lipopeptide as a function of simulation time.

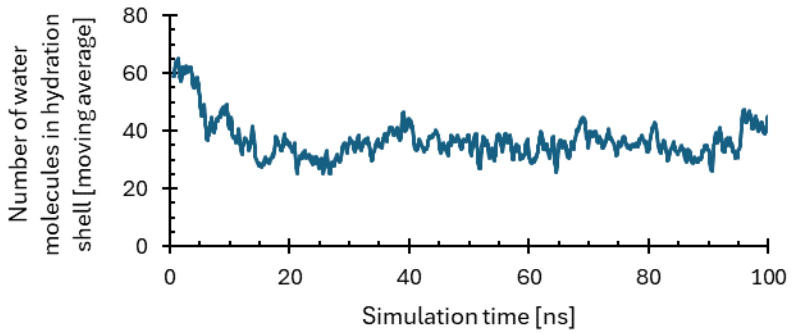

Figure 75: Total number of water molecules surrounding all viscosin alpha carbons within a 5 Å sphere as a function of simulation time.

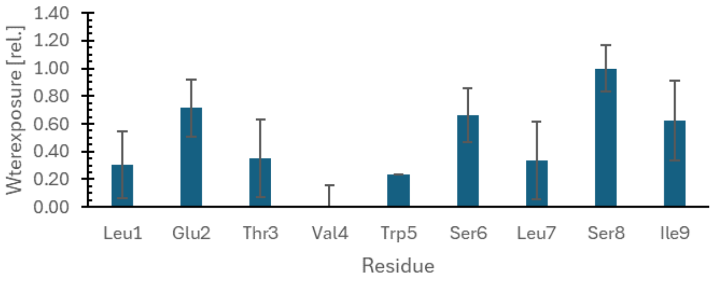

Figure 76: Normalized water exposure of the individual alpha carbons of the lipopeptide.

MD simulation of L7W

We monitored total energy, temperature, and density of the MD simulatins of the L7W-labelled viscosin system to ensure consistent behavior (Figures 77-79). The 'area per lipid' is used as a metric for assessing membrane stability and convergence, remaining stable throughout the simulation (Figure 80). In this simulation, the peptide positions itself within the interfacial region (*Figure 81*), similar to previous simulations. The number of water molecules hydrating the alpha carbons is similar to before. (Figure 82). The RMSD of the lipopeptide also remains stable throughout the simulation (Figure 83). Finally, quantitative analysis of hydration around each residues' alpha carbon again indicates that viscosin is surface-bound on the membrane bilayer, with Val4 largely shielded from the aqueous phase (Figure 84).

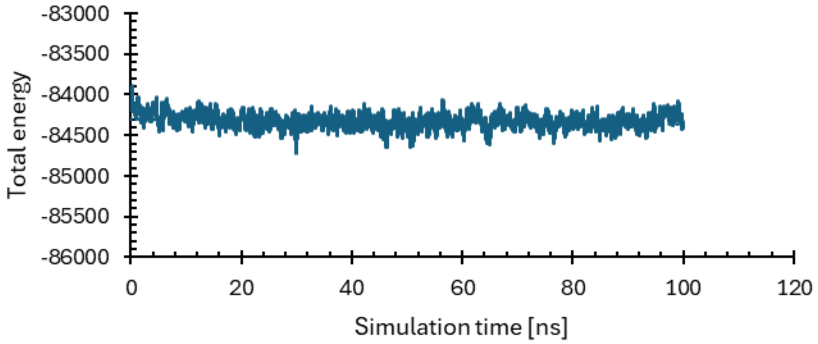

Figure 77: Total energy of the simulation, as a function of simulation time.

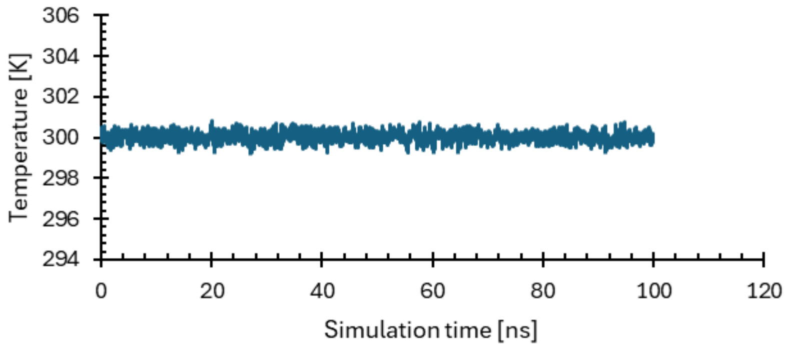

Figure 78: Temperature as a function of simulation time.

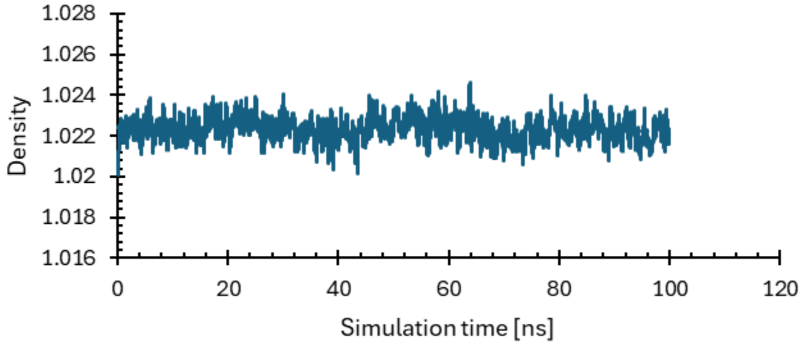

Figure 79: Density of the system as a function of simulation time.

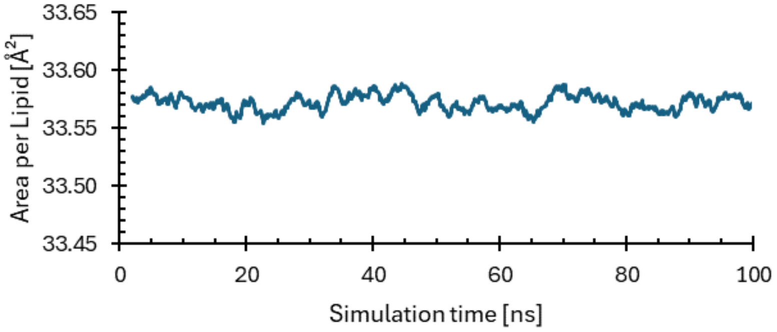

Figure 80: Area per lipid as a function of simulation time.

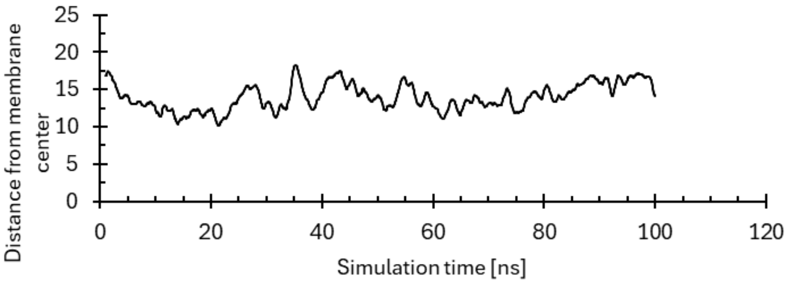

Figure 81: Distance of lipopeptide center of mass from the membrane center, as a function of simulation time.

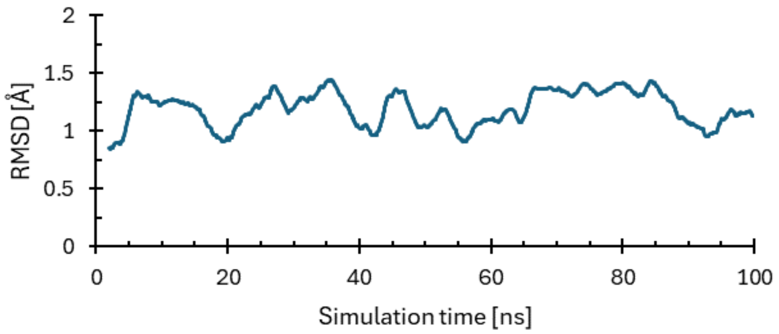

Figure 82: Heavy atom Root-Mean-Square-Deviation (RMSD) of the lipopeptide as a function of simulation time.

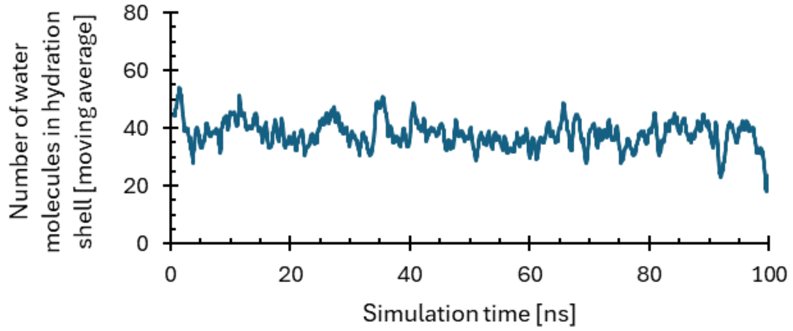

Figure 83: Total number of water molecules surrounding all viscosin alpha carbons within a 5 Å sphere as a function of simulation time.

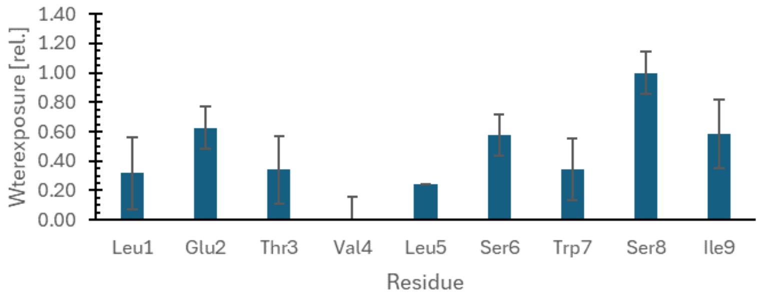

Figure 84: Normalized water exposure of the individual alpha carbons of the lipopeptide.

15. References

(10) N. Geudens, B. Kovacs, D. Sinnaeve, F. E. Oni, M. Höfte, J. C. Martins, *Molecules* **2019**, *24*.
