# Supplementary material for: Time-resolved fluorescence of tryptophan characterizes membrane perturbation by cyclic lipopeptides
Source: Biophys J. 2024 Jun 22;123(16):2557–73. doi: 10.1016/j.bpj.2024.06.022 (PMC11365112; doi:10.1016/j.bpj.2024.06.022)
Supplement: Document S2. Article plus supporting material [file mmc2.pdf]

Biophysical Journal
Article

Biophysical Society

Time-resolved fluorescence of tryptophan characterizes membrane perturbation by cyclic lipopeptides

Iulia Carabadjac,[1,*] Jessica Steigenberger,[1] Niels Geudens,[2] Vic De Roo,[2,3] Penthip Muangkaew,[3] Annemieke Madder,[3] José C. Martins,[2] and Heiko Heerklotz[1,4,5,*]

[1]Institute of Pharmaceutical Sciences, University of Freiburg, Freiburg, Germany; [2]NMR and Structure Analysis Research Group, Department of Organic and Macromolecular Chemistry, Ghent University, Ghent, Belgium; [3]Organic and Biomimetic Chemistry Research Group, Department of Organic and Macromolecular Chemistry, Ghent University, Ghent, Belgium; [4]Center for Biological Signaling Studies (BIOSS), University of Freiburg, Freiburg, Germany; and [5]Leslie Dan Faculty of Pharmacy, University of Toronto, Toronto, ON, Canada

ABSTRACT Viscosin is a membrane-permeabilizing, cyclic lipopeptide (CLiP) produced by *Pseudomonas* species. Here, we have studied four synthetic analogs (L1W, V4W, L5W, and L7W), each with one leucine (Leu; L) or valine residue exchanged for tryptophan (Trp; W) by means of time-resolved fluorescence spectroscopy of Trp. To this end, we recorded the average fluorescence lifetime, rotational correlation time and limiting anisotropy, dipolar relaxation time and limiting extent of relaxation, rate constant of acrylamide quenching, effect of H_2O-D_2O exchange, and time-resolved half-width of the spectrum in the absence and presence of POPC (1-palmitoyl-2-oleoyl-*sn*-glycero-3-phosphocholine) liposomes. Structure, localization, and hydration of the peptides were described by molecular dynamics simulations. The combination of the parameters provides a good description of the molecular environments of the Trp positions and the behavior of viscosin as a whole. Of particular value for characterizing the impact of viscosin on the membrane is the dipolar relaxation of Trp4 in V4W, which is deeply embedded in the hydrophobic core. The limiting relaxation level represents the membrane perturbation—unlike typical membrane probes—at the site of the perturbant. Fractions of Trp4 relax at different rates; the one not in contact with water upon excitation relaxes via recruitment of a water molecule on the 10-ns timescale. This rate is sensitive to the concerted membrane perturbation by more than one lipopeptide, which appears at high lipopeptide concentration and is assumed a prerequisite for the final formation of a membrane-permeabilizing defect. Trp7 relaxes primarily with respect to neighboring Ser residues. Trp5 flips between a membrane-inserted and surface-exposed orientation.

SIGNIFICANCE Tryptophan (Trp) is a very advantageous—intrinsic, or at the very least, conservative—label for peptide or protein studies. In particular, time-resolved experiments yield a multitude of observable parameters which, in turn, provide a wealth of information on structural and dynamic properties. At the same time, the low excitation wavelength and complex, multi-exponential fluorescence may render it challenging to handle. Here we demonstrate the diverse effects seen for Trp labels at four positions of a small cyclic lipopeptide and how all these parameters can be combined to provide a consistent picture of its membrane insertion and perturbation. This approach should be generally applicable to studying membrane effects of peptides and proteins.

INTRODUCTION

Time-resolved (TR) fluorescence has been utilized for decades to analyze the biological activity of membrane-active biomolecules and agents. In contrast to steady-state methods, TR measurements are more informative and less prone to artifacts, such as inner filter or light-scattering effects (1). Molecular processes on the timescale of nanoseconds are accessible through TR measurements. Understanding such molecular movements helps elucidate the mechanisms of action of active compounds.

A multitude of assays and methods using TR methods exist. Calcein leakage assays are used to characterize membrane permeabilization by antimicrobial peptides (2–4), synthetic alternatives (5–7), and (bio)surfactants (8–10).

Submitted February 13, 2024, and accepted for publication June 20, 2024.

*Correspondence: iulia.carabadjac@gmail.com or heiko.heerklotz@pharmazie.uni-freiburg.de

Iulia Carabadjac and Jessica Steigenberger contributed equally to this work.

Editor: Chris Neale.

https://doi.org/10.1016/j.bpj.2024.06.022

© 2024 Biophysical Society.
This is an open access article under the CC BY license (http://creativecommons.org/licenses/by/4.0/).

Here, the amount of dye trapped in a model membrane such as a large unilamellar vesicle (LUV) is measured in parallel with the amount of released dye. This is possible due to the self-quenching property of calcein, whereby the lifetime of the dye indicates its proximity to another dye molecule. Such assays accurately quantify membrane-damaging activity while offering some insights into mechanisms of action (11). Furthermore, subtler changes in membrane properties can be tracked by direct labeling of the membrane. TR-anisotropy measurements of diphenylhexatriene (DPH) and its derivates spread out over the membrane indicate a general membrane disordering by (bio)surfactants but may miss very local perturbations (12). TR emission spectral shift of Laurdan, Prodan, and other solvent-sensitive dyes was shown to reflect the polarity and mobility of their environment, depending on the localization of the dye in the membrane (13–16).

The disadvantage of these assays is associated with their highly specialized purpose. For instance, calcein cannot report on membrane order, DPH does not reflect membrane hydration, Laurdan cannot gauge membrane perturbation, and so forth. As such, each dye provides narrowly focused and specific information. Therefore, obtaining a comprehensive picture of membrane-targeting activity requires a multitude of measurements with different samples labeled with different dyes. Additionally, none of these dyes gives any direct insights into the molecular movement of the active compound, instead providing information about the lipid environment. Finally, the observations of the membrane-damaging activity are limited by averaging of the fluorescence signal across all areas of the membrane affected and not affected by the active compound. Since membrane damage is often highly localized, the main signal of unperturbed membrane will dominate the signal. The last problem may be resolved by labeling the active compound directly with a fluorescent dye so that the fluorescent signal originates from the compounds' vicinity. However, it can be expected that any kind of label attached to a small antimicrobial peptide also introduces a potential artifact into the measurements. The presence of such artifacts has been suggested by techniques other than fluorescence (17–19).

The use of tryptophan (Trp) as an intrinsic fluorophore eliminates the need for peptide labeling altogether if the peptide of interest naturally contains a Trp residue. Trp is metabolically "expensive" and as such is a rare amino acid, but it is strongly enriched in membrane or transmembrane proteins (20–22). Many studies suggest it plays an important role as a membrane anchor, as the indole ring is mostly found to be positioned near the glycerol backbone in the polar-non-polar interface of a lipid bilayer (23–25). The presence of Trp in a membrane-active peptide would provide signal directly and only from the affected regions of the membrane. If Trp is not present by nature, site-directed mutagenesis makes it possible to exchange a hydrophobic amino acid in the peptide sequence for Trp. While this introduces a relatively small interference in comparison to the attachment of a big fluorescent dye, the position of the "Trp label" still may matter for the biological function or the experimental observations. In some membrane-active peptides, introduction of Trp into the sequence increased biological activity and/or selectivity against specific bacterial strains (26–30). These effects were related to the number and position of Trp residues. The position and environment of Trp may matter for the observed fluorescence signal as well as its interpretation. This is why one of the aims of this study is to demonstrate how the position of Trp affects the observations regarding peptide mobility, peptide environment, and permeabilizing activity.

The fluorescence properties of Trp are highly sensitive to its surroundings (31–33). In comparison to the techniques introduced above, the spectroscopic properties of Trp allow measurements of TR anisotropy, quenching, and spectral shift with a single dye (i.e., Trp). Such a high density of obtained information makes Trp a very powerful fluorophore. At the same time, the complexity of interwoven observation of the movement of Trp, of the movement and polarity of the peptide, and the mobility and polarity of the environment of Trp all at once also comes with highly challenging analysis. With that, the second aim of this study is to dissect the different contributions to their corresponding factors and produce a clear readout of Trp TR fluorescence.

The peptides studied here are synthetic variants of the naturally occurring cyclic lipopeptide (CLiP) viscosin produced by *Pseudomonas fluorescens* (34), in which one hydrophobic amino acid is exchanged for Trp (further referred to as W-viscosins). CLiPs are amphiphilic, membrane-active compounds, produced by bacteria for host defense, swarming motility, or quorum sensing. Their great diversity in activity and structure makes them interesting lead compounds for antimicrobials (35–39) and biocontrol agents (40–43). However, their exact mode of action on a molecular level is mostly unclear. Information about the specific position and orientation of CLiPs in the membrane, and thus about possible interaction partners or mechanism of action, is essential.

In summary, we use the TR intrinsic fluorescence of the amino acid Trp introduced into a membrane-active peptide in four different positions. We compare the differences between the four chemically synthesized, single-Trp peptide analogs (W-viscosins) to each other, showing the relevance of the position of the label in the active compound or the membrane. We provide a comprehensive toolbox for the analysis of peptide mobility, localization, and orientation of the peptide in the membrane and changes in the peptide environment and verify them by comparison to molecular dynamics (MD) simulations. We include experiments for the determination of possible artifacts/distortions of the experimental readout depending on the Trp position in the peptide and the membrane. A combination of TR anisotropy, spectral shift, quenching, and other experiments with

MD simulations makes it possible to create a full picture of peptide-membrane effects.

MATERIALS AND METHODS

Chemicals

The zwitterionic phospholipid 1-palmitoyl-2-oleoyl-*sn*-glycero-3-phosphocholine (POPC) was a kind gift of Lipoid (Ludwigshafen, Germany) and used for liposome preparation. For lipid quantification, hydrogen peroxide solution (30 wt %), H_2SO_4, $(NH_4)_6Mo_7O_{24}\cdot 4H_2O$, and Fiske-Subbarow reducer were purchased from Sigma-Aldrich (St. Louis, MO). K_2HPO_4 was purchased from VWR (Leuven, Belgium). *N*-Acetyl-L-tryptophan amide (NATA) was purchased from Sigma-Aldrich. Tris-H_2O and Tris/D_2O buffer were prepared with EDTA purchased from Sigma-Aldrich, and NaCl, NaOH, HCl, and Tris were purchased from Carl Roth (Karlsruhe, Germany). Deuterium oxide (99.8% D) was purchased from Fisher Scientific (Schwerte, Germany). The solvents $CHCl_3$ and ethanol HPLC grade were bought from Carl Roth. Ultrapure water was prepared by the Arium Pro system (Sartorius, Göttingen, Germany).

Synthesis of the W-viscosins

The four W-viscosins L1W, V4W, L5W, and L7W were synthesized in 2- to 5-mg quantities via a total synthesis approach as described previously for viscosin and other *Pseudomonas* CLiP group members (44–46) (for more details see Section S1 of supporting material). The respective names indicate the type and position of the respective amino acid in naturally occurring viscosin which is replaced by tryptophan (Trp) (Table 1). The details of synthesis, purification, and complete structural characterization are collected in supporting material, Section S1. All W-viscosins were quantified by NMR spectroscopy (ERETIC methodology based on PULCON as described in (47)) and solubilized as 20 mM DMSO (Carl Roth) stock solutions.

Liposome preparation

POPC was dissolved in chloroform for thin lipid film preparation. Chloroform was removed by vacuum centrifugation at 36°C (RVC 2–18 Cdplus; Martin Christ, Osterode am Harz, Germany), and the lipid films were further dried overnight under high vacuum. The air in the vials was replaced by inert argon gas and the lids sealed with parafilm. Lipid films were stored at −20°C.

The lipid films were hydrated with either Tris buffer (110 mM NaCl, 10 mM Tris, 0.5 mM EDTA, pH 7.4 at 25°C) or heavy Tris buffer (110 mM NaCl, 10 mM Tris, 0.5 mM EDTA dissolved in D_2O instead of water, pH 7.4, measured with a glass electrode with all chambers filled with aqueous (H_2O) potassium chloride solution at 25°C without further corrections (48)) at room temperature. Four freeze-thaw cycles were then performed using dry ice and a water bath set at 50°C to increase the unilamellarity of vesicles in the lipid dispersion. The vesicle dispersion was then extruded through two stacked 100-nm polycarbonate membranes (Whatman Nuclepore) using a LIPEX Thermobarrel extruder (Evonik Industries, Essen, Germany) at 25 bar to produce large unilamellar liposomes (LUVs).

All LUVs were characterized with respect to their size (*z*-average of the hydrodynamic diameter 100–120 nm), polydispersity index (<0.1), and lipid content before use. The lipid content was determined according to the Bartlett assay (49), and all LUVs were subsequently adjusted to a common lipid content of 6 mM.

Sample preparation

All samples were prepared according to the "lipid-into-peptide" mixing protocol (8,50,51). Samples for TR fluorescence measurements contained always 6.6 μM of the respective W-viscosin in buffer, with or without the addition of 30, 60, or 90 μM POPC-LUVs. Samples for Trp quenching experiments contained 6.6 μM W-viscosin, 30 μM POPC-LUVs, and increasing amounts of acrylamide (0.1–0.4 mol L^{-1}). Sample concentrations were verified by weighing all pipetted volumes with a precision balance. Samples were prepared and measured in 10 × 10-mm quartz cuvettes (high-precision cell; Hellma Analytics, Müllheim, Germany). Before measurement, all samples containing W-viscosin with POPC-LUVs were incubated for 2 h at 400 rpm at 25°C protected from light. All samples were measured at 25°C and stirred during measurement.

TABLE 1 Amino acid sequence of the cyclic lipopeptides viscosin, L1W, V4W, L5W, and L7W in one-letter code

	Amino acid sequence								
CLiP	1	2	3	4	5	6	7	8	9
Viscosin	L (L)	E (D)	aT (D)	V (D)	L (L)	S (D)	L (L)	S (D)	I (L)
L1W	**W** (L)	E (D)	aT (D)	V (D)	L (L)	S (D)	L (L)	S (D)	I (L)
V4W	L (L)	E (D)	aT (D)	**W** (D)	L (L)	S (D)	L (L)	S (D)	I (L)
L5W	L (L)	E (D)	aT (D)	V (D)	**W** (L)	S (D)	L (L)	S (D)	I (L)
L7W	L (L)	E (D)	aT (D)	V (D)	L (L)	S (D)	**W** (L)	S (D)	I (L)

The macrocycle involves cyclization via ester formation between the C-terminal carbonyl and the side-chain alcohol of D-aThr. Changes in the sequence related to viscosin are highlighted in bold. L/D conformation of amino acids is indicated in parentheses. The β-OH C_{10} fatty acid, attached to the N-terminus of all viscosins, is not mentioned explicitly.

NATA samples were prepared by dissolving the desired amount of dry NATA powder in Tris buffer. The solution was then further diluted so that the attenuation of light (absorbance) by passing through the sample was less than 0.1. The degree of dilution was calculated using Lambert-Beer's law, assuming an extinction coefficient of 5500 L mol^{-1} cm^{-1} for NATA (52) (resulting concentration of 17–20 mM).

Time-resolved fluorescence measurements

All measurements of TR fluorescence decays except calcein leakage assays were performed using time-correlated single-photon counting (TCSPC) with the high-performance fluorescence lifetime spectrometer FluoTime300 (FT300) by PicoQuant (Berlin, Germany). For the measurements at FT300, the VisUV laser (PicoQuant) was used in 280-nm mode. Instrument settings were controlled, and initial data analysis was performed by the EasyTau 2.2.3293 system software (PicoQuant).

Time-resolved emission spectra

Detailed information on settings and measurement procedures are collected in Section S2 of supporting material. Peak-normalized time-resolved emission spectra (TRES) for W-viscosins were calculated at distinct time points after excitation (example in Fig. ESI2). For each point after excitation, the spectral width at half-maximum intensity was determined. Subsequently, the spectral center of gravity in wavenumbers, ν, of each spectrum was calculated according to Eq. 1 for TRES analysis (time-dependent fluorescence shift (TDFS), Fig. ESI3). The spectral center of gravity is proportional to the average energy emission, and its time-dependent change, $\nu(t)$, characterizes the extent and the timescale of the environment relaxation process taking place (1,53,54). We call $\nu(t)$, therefore, the relaxation level.

$$\nu(t) = \frac{\Sigma_\lambda I(\lambda, t) \times \lambda^{-1}}{\Sigma_\lambda I(\lambda, t)}. \quad (1)$$

In the next step, $\nu(t)$ was fitted monoexponentially according to Eq. 2 to determine the dipolar relaxation time, t_{relax}, and the maximum shift of the spectral center of gravity produced by the fluorophore in the fully relaxed

excited state, ν_∞. Because the energy shift ends at the value ν_∞, we call it the limiting relaxation level. C is a pre-exponential factor representing the detectable part of $\Delta\nu$ (see below).

$$\nu(t) = Ce^{-\frac{t}{t_{\text{relax}}}} + \nu_\infty. \quad (2)$$

The stronger the shift $\Delta\nu$, the higher the polarity of the surrounding molecules. This has been shown as a linear proportionality between $\Delta\nu$ and the dielectric measure of the solvent polarity (55). Usually, this time-dependent Stokes shift is quantified by

$$\Delta\nu = \nu(0) - \nu_\infty, \quad (3)$$

where $\nu(0)$ represents the spectral center of gravity of the spectrum produced by the initial Frank-Condon state, i.e., the spectrum before any molecular reorientation has yet occurred. In general, $\nu(0)$ can be estimated by measurements of the absorption and emission spectra in completely nonpolar solvents (55–57). However, this procedure is not applicable for W-viscosins (for details, consult Section S3 of supporting material). Instead of $\Delta\nu$, the limiting relaxation level, ν_∞, was used for the analysis. Note that in a heterogeneous system, not the predominant component but the component with the longest lifetime will dominate ν_∞.

Time-resolved anisotropy

For detailed information on measurement settings, consult Section S4 of supporting material. In brief, the measurements of fluorescence decays were performed at 340 nm with the emission polarizer set to vertical, horizontal, or magic-angle conditions. The resulting decays were reconvoluted with the instrument response function and fitted according to Eq. 4:

$$I(\theta,t) = \frac{k(\theta)}{3}\sum_{i=1}^{3} A_i e^{-\frac{t}{\tau_i}} \times \left[\left(1 + \left(3\cos^2\theta - 1\right)\right.\right.$$
$$\left.\left.\times \left(Be^{-\frac{t}{\varphi}} + r_\infty\right)\right)\right]. \quad (4)$$

The correlational relaxation time φ of the anisotropy decay and the limiting anisotropy r_∞ were set globally. Lifetimes of the fluorophore as determined by TRES or VM curve analysis (i.e., eliminating polarization effects by vertical excitation and magic angle emission polarizers), τ_1–τ_3, were used as input data for the fitting process. A_i describes the amplitude of the fluorescence decay component and B the amplitude of the anisotropy decay. $k(\theta)$ is an experimental matching factor for the polarized fluorescence decay collected at the emission polarizer angle θ. The term $\frac{k(\theta)}{3}$ is defined by Eq. 5, yielding $\frac{k(\theta)}{3} = 1$ for vertical emission polarizer alignment ($\theta = 0°$), $\frac{k(\theta)}{3} = \frac{1}{G}$ for horizontal emission polarizer alignment ($\theta = 90°$), and $\frac{k(\theta)}{3} = \left(\frac{2+G}{3\cdot G}\right)$ for magic-angle alignment ($\theta = 54.7°$).

$$\frac{k(\theta)}{3} = \cos^2\theta + \frac{1}{G}\sin^2\theta. \quad (5)$$

Time-resolved quenching assay

For detailed information of measurement settings, consult Section S5 of supporting material. For simplicity, we evaluated the decays in two steps, beginning with the very good, empirical fit of each decay by the "reconvolution" of a tri-exponential curve (Eq. 6) with the IRF. The "deconvoluted" decays were then reconstructed and fitted globally using a Stern-Volmer model (Eq. 7).

In more detail, the reconvoluted decays were reconstructed with the parameters A_{1-3} and τ_{1-3} acquired by Eq. ESI3 according to Eq. 6,

$$F(t) = A_1 e^{-\frac{t}{\tau_1}} + A_2 e^{-\frac{t}{\tau_2}} + A_3 e^{-\frac{t}{\tau_3}}, \quad (6)$$

where the fluorescence intensity as a function of time is given by $F(t)$, t is the time, and A is the pre-exponential factor of the component with the corresponding lifetime τ. The decay in the absence of quencher acrylamide, $[Q] = 0$, is denoted $F_0(t)$ and properly represented by Eq. 6.

The reconstructed decays obtained in the presence of five different acrylamide concentrations [Q] were then fitted globally with Eq. 7,

$$F_q(t) = F_0(t) \times \left(e^{-tk_q}\right)^{[Q]}, \quad (7)$$

keeping $F_0(t)$ fixed and adjusting the parameter k_q, which is the quenching rate constant in ns^{-1}.

For a highly effective collisional quencher and fully exposed dye in solution, k_q represents the bimolecular quenching constant of the Stern-Volmer equation (58), then approaching the intrinsic rate constant of a diffusion-controlled reaction. In our case, the local quencher concentration and diffusion coefficient at the site of a given Trp differ from that in the bulk. Hence, Eq. 7 using bulk concentrations [Q] will yield an apparent k_q value is the lower; the lower is the exposure of the Trp to the quencher (i.e., local [Q]).

The fit procedure was performed in Excel (Microsoft, Redmond, WA) using its built-in generalized reduced gradient non-linear fit algorithm (Solver).

Membrane permeabilization and kinetics by TCSPC

Peptide-triggered membrane permeabilization was quantified by the changes in the fluorescence lifetime of the self-quenching fluorescent dye calcein encapsulated in LUVs (calcein-LUVs). The theory underlying the calcein leakage assay is explained in detail elsewhere (2,11). In brief, the fluorescence lifetime of calcein depends on its local concentration due to collisional and static self-quenching effects. Therefore, calcein entrapped in liposomes (i.e., high local calcein concentration, shortened fluorescence lifetime) and released calcein (i.e., lower local calcein concentration, longer fluorescence lifetime) can be distinguished from each other and quantified independently. In our case, 0% and 100% membrane permeabilization correspond to fluorescence lifetimes of $\approx$0.4 ns and $\approx$4 ns ($c_{\text{calcein}} \approx$ 70 mM and $c_{\text{calcein}} \approx$ 6 μM), respectively. Encapsulated but increasingly diluted calcein due to leakage events yields lifetimes between 0.4 ns and 4 ns. For a detailed description of settings, measurement procedures, and analysis, consult Section S6 of supporting material.

Subsequently, calcein leakage L can be calculated by Eq. 8. $Q_{\text{Stat}} = 1.2 \pm 0.2$ (11) is an empirical correction factor accounting for amplitude reduction due to some static quenching next to the predominant collisional quenching effects. Not all calcein can be completely removed by size-exclusion chromatography during liposome preparation. This amount of free calcein is determined by a sample without peptide additive and accounted for by B_{F0}.

$$L = \frac{B_F - B_{F0}}{B_F - B_{F0} + Q_{\text{Stat}} B_E}. \quad (8)$$

Membrane partitioning

The membrane distribution behavior of the W-viscosins was determined according to a procedure published in (59) with slight modifications to instrument settings and data analysis. Steady-state emission spectra of W-viscosins and NATA with either 0, 15, 30, 60, or 90 μM POPC-LUVs were recorded with the FT300 (Fig. ESI4). Detailed measurement settings, measurement, and analysis procedures can be found in Section S7 of

supporting material. In brief, the change in fluorescence intensity at a given wavelength correlates linearly with the amount of peptide bound to the membrane (59). Subsequently, the intensities corrected for the scattering light effects (I'_W) for W-viscosins at a certain c_L and six arbitrarily chosen wavelengths (310, 320, 330, 340, 350, and 360 nm) were fitted globally to calculate the membrane partitioning coefficient K (μM^{-1}) of the W-viscosins by Eq. 9. I_{W100} and I_{W0} represent the fluorescence intensity upon complete peptide binding (100%) and no peptide binding at all to the lipid membrane (0%), respectively.

$$I'_W(c_L) = \frac{Kc_L}{1+Kc_L} I'_{W100} + \left(1 - \frac{Kc_L}{1+Kc_L}\right) I'_{W0}. \quad (9)$$

The dissociation constant K_d (μM) was then calculated as the reciprocal of K and used for analysis. High values for K_d represent a low membrane affinity of the peptide and vice versa. The goodness of fit was assessed by performing a support plane analysis and evaluated by the normalized sum of squared residuals (NSSR) derived from the quotient of individual SSR values and the SSR value of the best fit. The procedure used was described by Kemmer and Keller in 2010 (60). An arbitrary cutoff value of NSSR = 1.5 was chosen. Some NSSR plots showed rather flat slopes around the minimum, which resulted in wide error bars.

Molecular dynamics simulation of W-viscosins-POPC bilayer interaction

MD simulations of viscosin and W-viscosin analogs L1W, V4W, L5W, and L7W with a POPC membrane were performed and analyzed as follows. The three-dimensional structure of the tryptophan analogs of viscosin were generated by replacement of the original Leu or Val side chain by a Trp side chain, starting from the NMR-derived structure of "wild-type" viscosin (unpublished data). The LIPID14 and ff14SB force fields (61,62) were used for the unrestrained simulations. Structural parameters of the ester bond as well as the fatty acid chain were identical to those used previously (63).

CHARMM-GUI software (64) was used to create the pre-equilibrated POPC lipid system in a rectangular simulation box. The lipid system consisted of 294 lipids (147 per leaflet) at 300 K (temperature above the phase-transition temperature of POPC) at a NaCl concentration of 0.1 M where approximately 40 TIP3P (65) water molecules per lipid were added for system solvation. In the simulations, individual Trp analogs were placed with their molecular center at the level of the phosphatidylcholine headgroups at the water-bilayer interface of the pre-equilibrated and solvated POPC bilayer. The initial orientation was defined using the stable orientation observed for viscosin at the end of a 100-ns MD simulation using otherwise identical starting conditions. The system was neutralized by the addition of Na^+ ions such that the overall charge of the simulation box is zero. Usage of the SHAKE algorithm kept hydrogen-involving bond lengths constant such that a time step of 2 fs could be used. Langevin dynamics (collision frequency of 1.0 ps^{-1}) was used for temperature scaling. The particle-mesh Ewald summation computed electrostatic interactions (cutoff for non-bonded interactions of 9 Å). The equilibration protocol before simulation consisted of an initial minimization step relaxing the initially generated structure (fixation of solute molecules with strong positional restraint at 100 kJ mol^{-1} Å^{-2}, only positions of solvent molecules and ions were minimized) followed by a two-step heating process (first: sequential heating of system from 0 to 100 K; second: gradual heating to 300 K) while keeping the total volume constant. Lipids were fixated by applying a mild potential energy constraint of 10 kcal mol^{-1} Å^{-2}. Finally, GPU-accelerated MD simulation production runs were carried out using isotropic position scaling (PMEMD) at constant-pressure periodic boundary conditions using anisotropic pressure scaling in the membrane bilayer (xy) plane as part of AMBER18 (66). The total simulation time was 100 ns. This timescale was sufficient to avoid bias or trapping of the lipopeptide coordinates toward the starting structure while ensuring convergence of the MD simulations (for more details see supporting material, Section S11 and following). The lack of bias was established by monitoring a number of parameters in a separate set of MD simulations (E) where either 1) viscosin was placed at different starting positions but with the same orientation with respect to the POPC membrane, and 2) a simulation whereby viscosin was placed upside down at the water-bilayer interface position. From these it was found that viscosin adopts highly similar end locations within the POPC bilayer and this well within the initial 50 ns of the simulation, with reorientation requiring less than 100 ns, thus eliminating concerns for potential bias toward starting conditions. Also, extending the simulation time from 100 ns to 500 ns for viscosin did not result in the occurrence of any additional events or significant departures from the behavior witnessed in the shorter simulations, alleviating potential concerns regarding the simulation timescale (Section S12 of supporting material). Trajectories were visualized with VMD 1.9.3 (67) and analyzed with PTRAJ or CPPTRAJ (68) (both included in AMBER software). The "WATERSHELL" command of CPPTRAJ was adapted and used for the quantification of water molecules within the first and second solvation shell (cutoff set to 3.4 Å and 5.0 Å, respectively) of the Trp residues. Moreover, the distance between the Trp and nearby POPC headgroups was analyzed using radial distribution function analysis. Bilayer thickness could be extracted from the electron density plots of the phosphate groups. Likewise, insertion depth of an individual lipopeptide as a whole, or of an individual Trp residue, was done by performing a Gaussian fitting to their respective backbone electron densities for a selection of points along the simulation trajectory. This provides a measure for their distance from the membrane center. In all analyses, a simulation of wild-type viscosin was used as a reference. Relevant information on the MD simulations of the W-viscosins is collected in Section S14 of supporting material.

RESULTS AND DISCUSSION

W-viscosins share similar structure and localization in the membrane

Members of the viscosin group exhibit a shared overall conformation that is maintained in organic solvents and dodecylphosphocholine micelle solutions (63,69,70). This conformation is characterized by a left-handed α-helix extending from L-Leu1 to D-Ser6, followed by a loop connecting the C-terminus to the middle of the α-helix through an ester (or depsi) bond with the side chain of D-aThr3. This distinctive fold results in a pronounced segregation between hydrophilic and hydrophobic residues, generating an amphipathic surface. Consequently, this structural configuration leads to physicochemical properties pushing the molecule to interact with a membrane surface. The structure, orientation, and insertion depth of the W-viscosins were investigated by MD simulations. All W-viscosins were initially positioned exactly in the same orientation and position, placing its center at the level of the phosphatidyl headgroups and with an orientation in line with its amphipathic character. The duration of the MD simulation was set to 100 ns, giving the peptides sufficient time to adopt their preferred orientation in the POPC membrane bilayer (first 50 ns) before the analysis (last 50 ns).

The POPC bilayer remained planar throughout the simulation, showing no buckling, curving, or other asymmetry manifestations. All W-viscosins maintained the left-handed

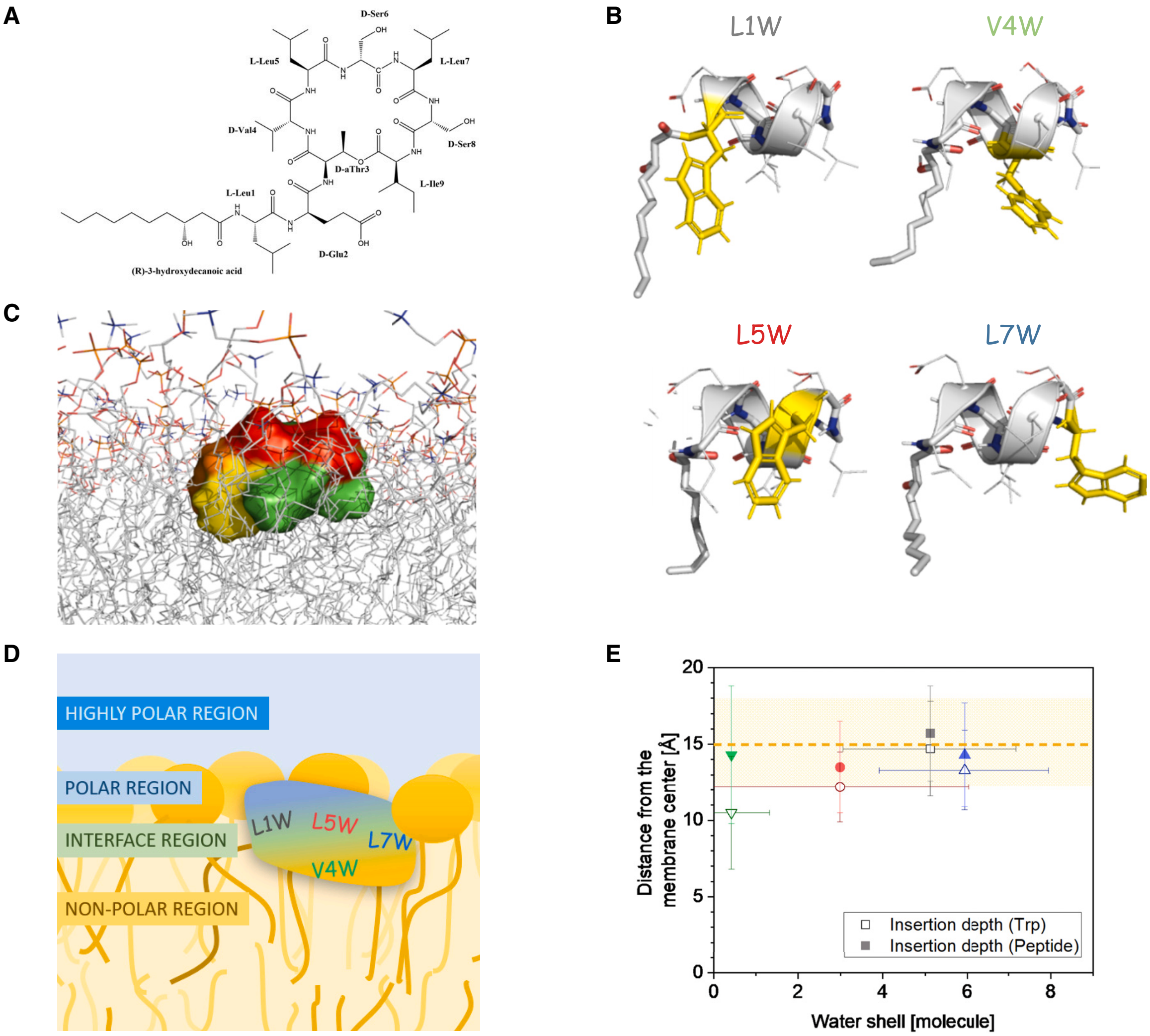

FIGURE 1 Overview of viscosin and the different W-viscosin analogs and of their proposed orientation in the POPC membrane. (*A*) Chemical structure of viscosin. (*B*) Snapshots of the conformation of the W-viscosins reached during MD simulations in the POPC membrane. Trp is shown in thick yellow stick representation. All W-viscosins share a very similar peptide conformation. (*C*) Snapshot of L1W as embedded in a POPC membrane bilayer. The lipid chains of POPC are colored gray, and the headgroup region orange (phosphate), red (glycerol), and blue (choline). The peptide surface map is colored as follows: green, hydrophobic; red, hydrophilic; orange, fatty acid; yellow, Trp. (*D*) Cartoon showing W-viscosins orientation and position in POPC membrane. The peptide body is marked by a tricolored oval. The color transition from blue to yellow indicates the separation of polar (*blue*) and non-polar (*yellow*) amino acids in this conformation of the peptide. The acyl chain is depicted as a brown line. The position of Trp for four W-viscosin peptides is marked as L1W (*gray*), L5W (*red*), L7W (*blue*), and V4W (*green*). The different regions of the membrane are labeled by the expected polarity of the environment. (*E*) Position of the Trp (*open symbols*) or the whole W-viscosin (*solid symbols*) in the POPC membrane and the corresponding number of water molecules in the solvent shell of Trp (within a 5 Å radius around indole) derived from MD simulations. The standard deviation bars indicate the variation during 50 ns of MD trajectory analysis. Color code for Trp as in (*D*). The dashed orange line indicates the average level of the glycerol moiety of lipids. The yellow shaded area marks the standard deviation of the glycerol position. To see this figure in color, go online.

α-helix extending from the first to the sixth amino acid, as observed for viscosin. The W-viscosins also retained the amphipathic conformation in which the hydrophobic residues of the peptide chain are spatially separated from the hydrophilic ones. Consequently, all W-viscosins inserted into the membrane similarly to the wild type, with the hydrophilic residues located between the phospholipid headgroups and the hydrophobic residues positioned between the lipid tails of the lipids, as exemplarily shown in Fig. 1 *C* for L1W.

The localization of the Trp residues follows their attachment to the backbone; the orientation from there is likely affected by the preference of Trp for the hydrophobic-hydrophilic interface (23–25). Snapshots of all four W-viscosins are shown in Fig. 1 *B*; they are schematically represented by the cartoon in Fig. 1 *D*. Trp1 and Trp7 are found in the carbonyl region at intermediate polarity (the hydrophobic-hydrophilic interface). Note that Trp1 is located close to a glutamate residue and the lipidic chain attachment site; Trp7 is flanked by two serine

residues (Fig. 1 *A*). Trp4 is embedded into the membrane's acyl chain region, "below" the carbonyl region. Trp5 alternates between two orientations in the simulation, one closer to the aqueous phase (snorkeling "up," represented in the cartoon Fig. 1 *D*) and one directed more toward the membrane core (reaching "down," snapshot shown in Fig. 1 *B*).

The cartoon representation in Fig. 1 *B* is confirmed by the primary localization of the Trp residues in the membrane (ordinate) and the average number of water molecules in its hydration shell (abscissa) as derived from the MD simulation (Fig. 1 *E*). Naturally, a deeper insertion correlates with a weaker hydration. Here, the point for L5W represents the average orientation of the Trp during the MD run (whereby the "down" orientation is more populated).

The differences in TR fluorescence readout of Trp in W-viscosins are not based on structural labeling artifacts

Since the Trp residues in this study are not native but used as low-impact labels with respect to molecular function, it needs to be shown that labeling does not change the molecular properties of the parent peptide viscosin significantly. To evaluate the effects of the exchange of a wild-type residue for Trp in different positions in the sequence, the general features of the W-peptides are compared with each other and their wild-type parent peptide.

The structure and positioning of the W-viscosins within the membrane are essentially conserved upon W-labeling, as demonstrated above via MD simulations.

Membrane perturbation activity of W-viscosins and wild-type viscosin was tested by the calcein leakage assay of POPC-LUVs (Fig. 2) as demonstrated for viscosin and

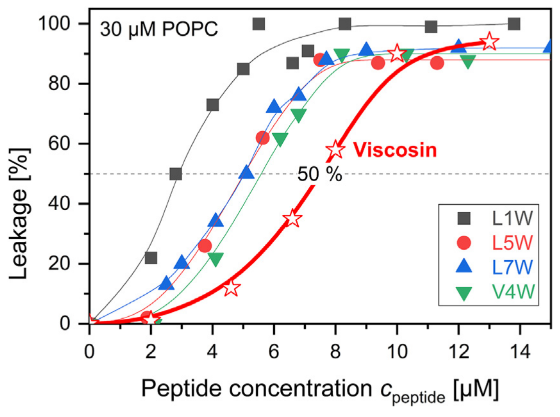

FIGURE 2 Calcein leakage (%) after 2 h of incubation plotted as a function of increasing W-peptide concentration $c_{peptide}$. All experiments were performed with 30 μM POPC-LUVs. Gray squares represent data of L1W, downward green triangles V4W, red circles L5W, upward blue triangles L7W, and red stars wild-type viscosin. Lines were fitted by Hill equation and are to guide the eye only. The dashed line marks the value of 50% leakage. To see this figure in color, go online.

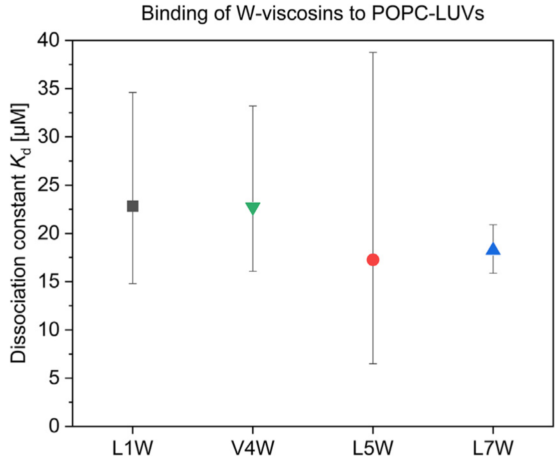

FIGURE 3 The dissociation constant K_d of L1W (*gray square*), V4W (*downward green triangle*), L5W (*red circle*), and L7W (*upward blue triangle*) from the POPC membrane is shown. The error bars are derived by support plane analysis (60) and represent an arbitrary cutoff value of normalized sum of square residuals of 1.5. To see this figure in color, go online.

other analogs previously (4). All W-viscosins cause increasing leakage of 30 μM POPC-LUVs in the low-micromolar range of 2–10 μM. At a concentration of $\approx$7 μM W-viscosins, the membrane is disturbed to a level where essentially all initially trapped calcein leaks out of the vesicle within 2 h. The W-peptides are slightly more active than their parent peptide viscosin, which reaches this level of membrane distortion at 12 μM. Note that activity tests (such as for determination of the minimal inhibitory concentration, MIC) are typically done on a log scale with a twofold dilution series—a factor of 2 in half-active concentration means a minor change on biological activity scales.

Similar observations were reported for the MIC of synthetic W-pseudodesmins, where the activity was mostly conserved within a factor 2 of the parent peptide (46). The structure of pseudodesmin A is related to that of the viscosin with small differences in sequence in position 2 (Glu instead of Gln) and chirality in position 5 (D instead of L).

All W-peptides were shown to have an apparent membrane-water partitioning similar to that of a POPC membrane (Fig. 3) as quantified by a dissociation constant $K_d \approx 20 \pm 15$ μM. For comparison with standard leakage experiments showing membrane-permeabilizing activity, we have performed the experiments here at 30–90 μM lipid. The K_d implies that under these conditions, the peptides are predominantly but not completely membrane-inserted.

Additional dynamic light-scattering experiments were performed with samples of about 6.6 μM W-viscosin at 30 μM lipid (Fig. ESI4). The *z*-average (the intensity-weighted mean hydrodynamic size) of the liposomes and the polydispersity index of the samples did not change significantly before and after the addition of the W-viscosins to the liposomes. This suggests that detergent effects such as vesicle

disruption, fusion, or solubilization to mixed micelles are not yet triggered to a detectable extent at these concentrations.

In summary, the structure of the W-viscosins in the membrane is not affected by the substitution of the selected residue by Trp. The degree of damage caused to the membrane by the peptides is similar for all W-viscosins, as is their partitioning between membrane and buffer.

Limiting anisotropy and relaxation level of Trp report on local effects of W-viscosins

Membrane perturbation is expected to result in membrane disorder, which may also have an impact on membrane hydration and kinetics. The last two mentioned membrane properties are usually detectable by TR anisotropy (12) and TRES (55). Fig. 4 shows the limiting anisotropy, r_∞, and limiting level of relaxation, ν_∞, as a function of lipid concentration, c_L. The values at zero lipid represent the behavior of W-viscosins in an aqueous solution or dispersion. With increasing lipid concentration, increasing fractions of the W-viscosins are taken up into the membranes and, at the same time, the relative content of peptide (per lipid) in the membranes decreases. Although both r_∞ and ν_∞ are not strictly linear-response functions of partitioning (59), the very similar amplitudes and lifetimes of fluorescence decays from the buffer-exposed and the membrane-bound W-viscosins encouraged us to treat them as such. This means that the parameters as observed (superscript obs) were fitted as a linear combination of characteristic contributions from aqueous (superscript aq) and membrane-bound (superscript m) W-viscosins, weighted by the respective fractions (X) of the peptide in water and membrane:

$$r_\infty^{obs}(c_L) \approx X_p^m r_\infty^m + \left(1 - X_p^m\right) r_\infty^{aq} \qquad (10)$$

with

$$X_p^m = \frac{c_L}{K_d\left(1 + \frac{c_L}{K_d}\right)}, \qquad (11)$$

where the dissociation constant K_d is defined as the reciprocal of the mole-ratio partition coefficient (71). For stable fit parameters, K_d was fixed at the values derived from the steady-state experiment described above. The fit results are presented in Table 2.

The fits were performed with the additional implicit assumption that r_∞ and ν_∞ of membrane-bound W-viscosin are approximately constant, i.e., independent of the W-viscosin content in the membrane. The fit curves are in good agreement with the data (Fig. 4), indicating that the underlying assumptions had been warranted to a good approximation.

Note that this finding of largely composition-independent order and relaxation is by no means trivial. Membrane probes such as DPH and di-methylaminonaphthalene derivatives typically show a continuous decrease of r_∞- and ν_∞-like parameters, respectively, upon increasing membrane content of surfactant-like perturbants (72,73). The fundamental difference is that these membrane probes report the overall, average state of the membrane that is not significantly perturbed as long as the perturbant is dilute and the average distance between the perturbant and probe is large. In strong contrast, W-viscosins combine perturbant and reporter probe in one molecule and report local perturbance already in the dilute state.

Hence, the fit values of $r_\infty{}^m$ and $\nu_\infty{}^m$ can directly be interpreted as measures of locally perturbed order and dielectric relaxation at the different label positions. In other

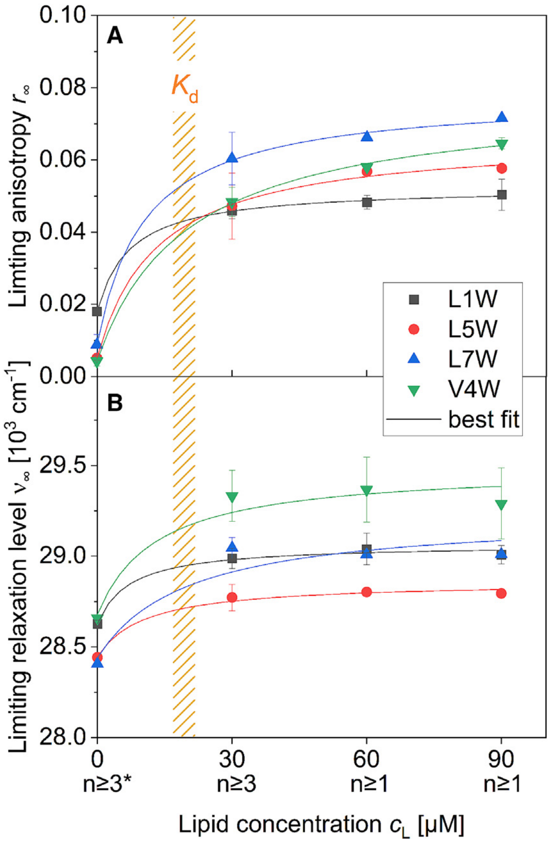

FIGURE 4 Change in properties of Trp (A) and the environment of Trp (B) depending on the amount of present POPC-LUVs. (A) The limiting anisotropy, r_∞, of W-viscosins and (B) the limiting level of relaxation depicted as the spectral center of gravity of time-resolved emission spectra (TRES) in wavenumbers, ν_∞, as a function of lipid concentration, c_L. Number of replicates is displayed under the respective x-axis label. Error bars are standard errors of the mean of replicates. The solid lines show the best fit calculated for a W-viscosin population distributed between the buffer and the membrane according to Eqs. 10 and 11. The orange shaded area marks the lipid concentration range at which half of the W-viscosins are bound to the membrane, the dissociation constant K_d. *The values of the limiting relaxation level at 0 μM lipid are approximate. The monoexponential fit was limited from 0 to 2 ns due to the subsequent rise of ν(t) at longer times and does therefore not represent the full extent of the TR fluorescent shift. For more detail read the last section. To see this figure in color, go online.

TABLE 2 The fit values for the limiting anisotropy in aqueous and lipid environment r_∞^{aq} and r_∞^{m}, respectively, and for the limiting relaxation level in buffer, ν_∞^{aq}, and the membrane, ν_∞^{m}, of the four W-viscosins

	K_d, fixed (μM)	r_∞^{aq}	r_∞^{m}	ν_∞^{aq} (10^3 cm^{-1})	ν_∞^{m} (10^3 cm^{-1})
L1W	23	0.019	0.061	28.64	29.17
L5W	17	0.005	0.070	28.45	28.90
L7W	18	0.010	0.086	28.44	29.21
V4W	23	0.005	0.080	28.70	29.59

The values obtained are as explained for Eqs. 10 and 11.

words, the approach presented here should permit comparing all these local perturbation parameters of a peptide acting, for example, on membranes of different lipid composition. Such a study (which is well beyond the scope of this paper) could help understanding of the membrane (and hence, organism) selectivity of the antimicrobial activity of a peptide. Understanding the local perturbation (as compared to the global one of the whole membrane) has the advantage that leakage-inducing defect formation, a typical mode of action of membrane-active peptides, is a very local phenomenon that needs only one or a few sites to be effective so that W-viscosin action might not be accompanied by a significant global membrane disordering (12). While the ability to probe local perturbance at the site of the W-viscosins is certainly a valuable step forward, it should be kept in mind that a liposome or bacterial membrane typically contains at least thousands of antimicrobial peptide molecules when, finally, a few of these molecules induce a membrane defect resulting in leakage and biological activity. The local order and relaxation at the very site of a defect may yet differ from the local perturbance at the sites of the vast majority of currently inactive peptides. Unfortunately, the latter will govern virtually all experimental data on the peptide structure, position, orientation, dynamics, and so forth.

A first glance at the individual results shows, as expected, higher mobility and stronger environmental relaxation of the W-viscosins in buffer compared to the membrane-embedded state. Slightly enhanced r_∞^{aq} of Trp1 and ν_∞^{aq} of Trp1 and Trp4 in comparison to other W-viscosins could be explained by a partial intra- or intermolecular coverage of these fluorophores (see below). The relaxation in the membrane-bound state (expressed in terms of ν_∞^{m}) of the different W-viscosins will be discussed next.

Extent of dipolar relaxation is dominated by Trp-water interactions for most but not all Trp positions

The fact that the extent of dipolar relaxation is similar for Trp1 and Trp7 but lower (higher ν_∞^{m}) for Trp4 (Fig. 4 *B* and Table 2) is in line with the localization and resulting water exposure of these residues as displayed in Fig. 1 *E* (*open symbols*). The reorientation of water dipoles (bulk or in hydration shell) is well known as a key mechanism of dipolar relaxation. However, the predominant orientation of Trp5 pointing toward the membrane center ("down" orientation) observed in MD and its relatively weak water exposure (Fig. 1 *E*) do not explain the maximal relaxation effect of Trp5 (Fig. 4 *B*). A possible explanation is offered above with the finding that Trp5 has a second characteristic "up" orientation sticking out toward the membrane surface. If this orientation, which appeared less populated in MD, would nevertheless dominate the TR fluorescence result, this would be in accord with the particularly low ν_∞^{m} of L5W.

To test this hypothesis experimentally, we studied the fluorescence quenching of the different Trp positions by acrylamide. This is a potent, highly water-soluble quencher known to be able to report on the depth of Trp incorporation in a membrane (74–76). Accessibility to the quencher should be directly related to accessibility to water. The results in Fig. 5 confirm that there is, indeed, a correlation between the apparent rate constant of dynamic quenching, k_q, and ν_∞^{m}. The highest exposure of Trp5 among the W-viscosins to quencher and water seems to be in line with an "up" orientation.

It should, however, be emphasized that the correlation between water accessibility and relaxation does not imply that it is actually the reorientation of water that causes the relaxation. This correlation has a strong confounder, the presence of polar groups of peptide or lipid in a given position that both attract water and serve as an alternative mode of dipolar relaxation. An approach to test whether the water itself is responsible for relaxation is based on an H_2O/D_2O comparison.

Water and heavy water (D_2O) have nearly identical polarity but were shown to differ in the strength of hydrogen bonds (77). As a consequence, dipolar relaxation is possible to a

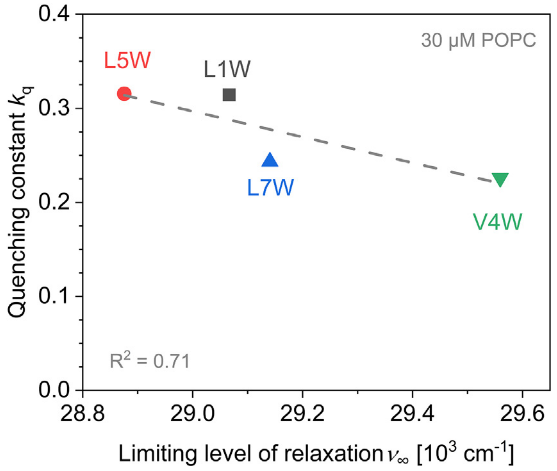

FIGURE 5 The apparent quenching rate constant in the presence of 30 μM POPC-LUVs, k_q, shown as a function of the fitted limiting relaxation level (center of gravity of TR spectra after infinite time), ν_∞. The gray dashed line represents the linear fit of data points. The quality of the fit is represented by R^2. To see this figure in color, go online.

similar extent in both but slower in heavy water. For virtually fully buffer-exposed Trp in NATA, switching from buffer prepared with normal water to buffer prepared with heavy water increases the relaxation time by a factor of $R_{D/H} = t_{rel}(D_2O)/t_{rel}(H_2O) = 1.53$ (78). A similarly high $R_{D/H}$ is expected whenever relaxation proceeds via bulk-like water reorientation only. For the mostly membrane-inserted W-viscosins in the presence of 30 μM lipid, the heavy-water effect was reduced to about $R_{D/H} \approx 1.2$ for Trp1, Trp5, and Trp4 as displayed in Fig. 6 *A*. This is in line with the fact that in the membrane-embedded state of a peptide, interactions of Trp with lipids and neighboring amino acids contribute to dipolar relaxation, and the remaining water molecules in the vicinity of the fluorophore are altered with respect to their mobility. Interestingly, a similar $R_{D/H}$ of 1.18 has been reported for another probe, Laurdan, embedded primarily in the carbonyl region of POPC membranes (79). This suggests the Trp residues at positions 1, 4, and 5 sense a similar environment and similar dipolar relaxation effects as Laurdan, which is discussed to reflect primarily the mobility of the hydrated lipid carbonyl group (16).

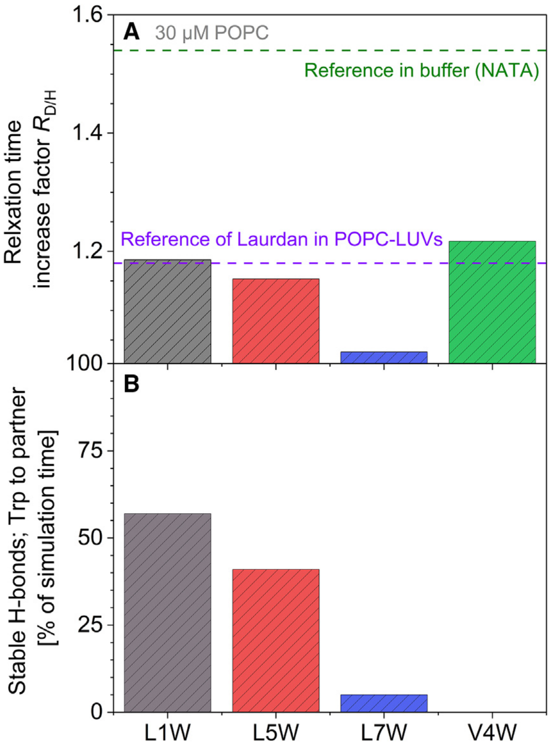

FIGURE 6 Differences in W-viscosins environment sensed by Trp (*A*) or resulting from MD simulations (*B*). (*A*) The ratio of relaxation time of W-viscosins detected in 30 μM POPC-LUVs prepared with buffer based on heavy water or normal water. L1W data are shown in gray, L5W in red, L7W in blue, and V4W in green. The violet grid line shows the ratio level for literature data of Laurdan in POPC membrane prepared with D_2O or H_2O at the Martin Hof group (79) and represents an average effect to be expected for a molecule inserted near the carbonyl group region of the membrane. The green grid line shows the ratio level for water-soluble Trp derivate NATA solubilized in buffer and represents the effect to be expected for pure solvent relaxation. (*B*) Occurrence of stable hydrogen bonds (H-bonds) between the Trp and other molecules as a percentage of the MD simulation time. All observed stable H-bonds are between the nitrogen of the indole of Trp and the oxygens of the carbonyl group of lipids. No stable H-bonds to water molecules occurred. Note the complete absence of H-bonds between V4W and the lipids. To see this figure in color, go online.

L7W shows a fundamentally different behavior; its relaxation is virtually unaffected by switching to heavy water. In this case, relaxation does not seem to arise from water to a significant extent. Notably, Trp in L7W is flanked by two serines—polar residues likely to reorient according to the dipole of the excited state of Trp. Conversely, the proximity of glutamic acid to the Trp in L1W seems not to affect the relaxation time ratio in the same way. The significance of the slightly higher $R_{D/H}$ of Trp4 is not established, but it may still be instructive to discuss this value. Trp4 shows the weakest extent of relaxation, since it is embedded in the acyl chain region of the membrane. A higher $R_{D/H}$ may mean that small amounts of water molecules interacting with the π-electron system of Trp may, although scarce, be still more active in relaxation than polar groups of the lipid that seem outside the reach of Trp4.

Lipid-Trp hydrogen bonds were quantified in the MD simulation (Fig. 6 *B*). The occurrence of hydrogen bonds (H-bonds) is shown as a percentage of the time they were present during the last 50 ns of the simulation (Fig. 6 *B*). Closer inspection reveals that all observed H-bonds occur between the nitrogen of the indole of Trp and the oxygens of the carbonyl group of the lipid molecules. No continuous H-bonds to water molecules could be detected. In line with the speculation in the previous paragraph, Trp4 is unable to interact with the lipid carbonyls. Trp7, which was argued to relax without substantial involvement of water according to $R_{D/H} \approx 1$, also does not seem to interact much with the lipid carbonyls. This supports the idea that in contrast to the other W-viscosins and despite the large hydration shell (see Fig. 1 *E*), the relaxation of Trp7 is governed by intramolecular interactions with Ser6 and Ser8.

MD simulations and TR fluorescence complement each other

Although the interpretation of MD simulations and TR fluorescence results leads to similar findings, some crucial differences between both methods have to be kept in mind.

Further to the different limitations and error sources of the two approaches, a main issue arises from the coexistence of two or more states of a molecule or moiety. Here, examples for such cases would be the partitioning of W-viscosins between buffer and membrane, equilibria of W-viscosin self-association, or the coexistence between two orientations of the Trp residue of L5W resulting in a more superficial or more membrane-embedded localization.

For MD simulations, it is always a challenge to properly represent the equilibrium between such alternative states given the limited number of molecules and time represented

by a simulation. Fluorescence parameters obtained in a cuvette should typically involve good statistics but may be differently sensitive to signals from the different states. Steady-state data represent different states weighted according to their quantum yields, which for Trp and many other fluorophores are reduced with increasing exposure to water. Amplitude-averaged parameters of TR measurements eliminate the effects of different lifetimes but would still be affected by static quenching phenomena. Finally, fluorescence parameters reflect the properties of the excited state of the fluorophore. As excitation goes along with a significant change of the dipole moment, the optimal localization of an excited-state Trp should differ from that of that in the ground state. Depending on the timescales of Trp relocalization and fluorescence, this may also affect the experimental result.

The latter phenomenon may explain why the preferred position of Trp of L5W seems to differ between the two methods. According to TR fluorescence, Trp5 is mostly exposed to the membrane surface. In the MD simulation, Trp5 reoriented between two characteristic insertion depths (Table 3), favoring the membrane-embedded "down" orientation. One general way to explain a stronger representation of the "up" orientation in TR fluorescence would be a static quenching process that reduces the fluorescence amplitude of the "down" Trp. In comparison, a reduction in lifetime from a dynamic quenching process would affect only steady-state fluorescence data. Another potentially relevant phenomenon would be the directed reorientation of Trp5 after excitation. Excitation leads to a higher dipole moment that might render the more polar environment in the surface-oriented "up" localization more favorable than in the ground state. During the simulation time, it took the Trp approximately 1 ns to overcome a distance of 1 Å. If proceeding at a similar speed, excitation-induced relocalization favoring the superficial state may become visible as an additional relaxation mechanism and reduce ν_∞ and, perhaps, r_∞.

TABLE 3 Distance from the membrane center of the W-viscosins and their Trp, determined by fitting a Gaussian function to the backbone electron density in the last 50 ns of MD simulation

	Distance from the membrane center (Å)	
	Peptide	Trp
Viscosin	15.2 ± 3.4	–
L1W	15.7 ± 3.1	14.7 ± 3.1
V4W	14.3 ± 4.5	10.5 ± 3.7
L5W[a]	15.2 ± 2.8	14.0 ± 1.3
	11.8 ± 3.2	10.4 ± 3.2
L7W	14.3 ± 3.4	13.3 ± 2.6

[a]Peptide shows occurrences where it inserts deeper into the membrane.

TR spectral width reveals heterogeneous relaxation behavior of Trp4 but not Trp5

Textbooks (80) describe two principal modes of relaxation, continuous versus two-state. A continuous shift of the spectrum to longer wavelengths with time is, for example, expected for a dye in a homogeneous solvent where after a given time, the environment of all excited dye molecules has relaxed to the same extent. Such a shift is indicated by a largely constant spectral width. A two-state relaxation may cause the decrease of an emission peak at the expense of a new one appearing at a higher wavelength. Even if the two peaks are not visibly separated, the coexistence of the two spectra at intermediate time will render the overall spectrum broader. Such a transient broadening will also be seen if two fractions of a dye, i.e., in water and the membrane, relax at different rates. At intermediate times, the fractions emit at different wavelengths, and the spectrum is broadened. As the faster-relaxing fraction becomes relaxed, the spectrum narrows again.

The changes in the width of the spectra throughout the relaxation process (TR full width at half maximum, TR-FWHM) were shown to be indicative of the level of heterogeneity of the environment of the fluorophore (13,78). Fig. 7 shows such profiles for the W-viscosins.

Literature values of spectral width for Laurdan in POPC membrane and Trp in various media are used for reference. For Laurdan, reported TR-FWHMs show a transient maximum at around 1 ns and a spectral width change of $\approx 2\ 10^3\ \mathrm{cm}^{-1}$ (13). The transient maximum of Trp in a heterogeneous environment of diethylether and water was shown to be somewhat similar (i.e., transient maximum at ≈ 1 ns; spectral width change of $\approx 2\ 10^3\ \mathrm{cm}^{-1}$), but much broader (FWHM of the transient peak ≈ 6 ns for Trp in comparison to 1–2 ns for Laurdan) (78). The highest change of spectral width reported in this study is relatively small, $<0.5\ 10^3\ \mathrm{cm}^{-1}$.

TR-FWHMs of L1W and L5W already reach a constant value after the first 1–2 ns. The small changes in spectral width over time are in reasonable agreement with the continuous model of solvent relaxation (80), where the spectra do not change their shape considerably ($<0.1\ 10^3\ \mathrm{cm}^{-1}$) during the shift.

A very prominent transient maximum of the FWHM has been observed for V4W after 7 ns. Seven nanoseconds is much slower than the usual solvent relaxation reported for hydrated POPC membranes. In other words, there are two or more fractions of Trp4 sensing relaxation at different rates, at least one extremely slowly. This observation harmonizes previous, partially confusing pieces of evidence to suggest a consistent scenario. To recall, we had stated that the environment of Trp4 relaxes to a small extent (high $\nu_\infty{}^m$ compared to other W-viscosins) and largely by water reorientation (high $R_{D/H}$, no H-bonds to lipid). However, during the MD simulation, the number of water molecules in contact with Trp4 was reported to be ≈ 0.4, which translates to one water molecule being present in the Trp

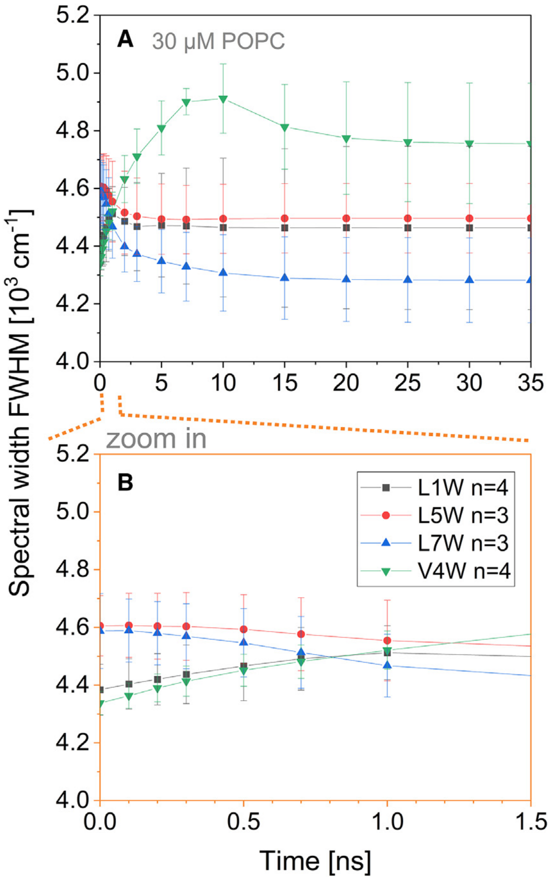

FIGURE 7 (*A*) The spectral width at half-maximum intensity (FWHM) of W-viscosins in 30 μM POPC-LUVs as a function of time after excitation. (*B*) A zoomed-in section of (*A*) from 0 to 1.5 ns. Gray squares indicate data for L1W, downward green triangles V4W, red circles L5W, and upward blue triangles L7W. Symbols represent an average of actual data points. *n* represents the number of replicates and the error bars the standard deviation. Lines are to guide the eye only. To see this figure in color, go online.

environment for 40% of the simulation time. In the fluorescence experiments, where many V4W peptides are observed simultaneously, the Trp4s can be divided into two distinct fractions: Trps that are and Trps that are not in contact with a water molecule at the time of excitation. This is exactly what is required for the FWHM maximum. The water-interacting fraction of Trp4 may sense relaxation at a reasonable rate, even if the extent is rather weak. The water-free environment of Trp4 cannot relax until a water molecule comes into contact with Trp—a process that will proceed via diffusion and could be promoted by the fact that the excited-state Trp attracts water due to its increased polarity. This diffusive or active recruitment of relaxing water then accounts for the component that needs to relax in order to narrow the spectrum down again.

The spectrum of L7W does not show a transient maximum of FWHM but becomes narrower with time by about 0.3 10^3 cm^{-1}. A possible explanation for this type of TR-FWHM is a starting state involving a Trp population with a broad distribution of degrees of relaxation. With time, the unrelaxed "flank" of the spectrum vanishes as the environments of these Trps also relax. This seems in line with the hypothesis of Trp7 relaxation to occur intramolecularly with respect to the flanking Ser residues. In contrast to a dye in a free solvent, the mutual orientations of the adjacent side chains of a compact cyclic peptide are restricted in orientation and motion (note high values for r_∞, Fig. 4 *A*). A fraction with an "excited-state relaxed" mutual orientation of Ser-Trp-Ser may exist in the ground state despite non-optimal dipole-dipole interactions simply because of steric reasons.

"Relaxation by water recruitment" model explains the peculiar sensitivity of only Trp4 for collective membrane perturbation effects of W-viscosins

Fig. 8 collects the kinetic parameters representing the speed of motions and relaxation in the membrane, the rotational correlation time φ, the relaxation time t_{relax}, and the amplitude-averaged fluorescence lifetime, τ_{av}, as a function of lipid concentration c_{L}. The figure practically complements Fig. 4, which presented the extent of these motions as specified by r_∞ and ν_∞.

First, Trp4 shows the fastest angular motion but the slowest relaxation of all W-viscosins studied. This can be explained by the "relaxation by water recruitment" model proposed above: on the one hand, the weak (essentially van der Waals only) interactions allow for a fast movement of Trp4 in its rather disordered, hydrophobic environment in the acyl chain region of the membrane. On the other hand, the slow component of relaxation does not represent the mobility of the Trp or that of the lipid chains. Instead, it is thought to describe the speed of water recruitment within or to this hydrophobic region. Such slow dynamics of water molecules in the hydrophobic region of the membrane were previously detected with other fluorophores (81).

Second, the t_{relax} of V4W cannot be explained solely by partitioning of V4W into the membrane. The data points with corresponding error bars in Fig. 8 *C* are not overlapping with the fit describing the theoretical t_{relax} resulting alone from averaging the parameter over the mix of V4W in buffer and membrane. This is the one and only parameter compiled in Figs. 4 and 8 that responds sensitively to the W-viscosin content within the membrane. Relaxation speeds up from a rate of $1/t_{\mathrm{relax}} = 0.10\ \mathrm{ns}^{-1}$ to $0.17\ \mathrm{ns}^{-1}$ as the local peptide-to-lipid mole ratio within the membrane, R_{b} (estimated assuming $K_{\mathrm{d}} \approx 20\ \mu\mathrm{M}$, see above) increases from 0.06 (at 90 μM lipid) to leakage-inducing 0.13 (at 30 μM). In other words, t_{relax} of V4W shows a significant concerted action of two or more W-viscosin molecules to perturb the membrane as it approaches leakage. This perturbation is, apparently, accompanied by an enhanced penetration and/or mobility of water into the hydrophobic core of the membrane. This is very plausibly a process needed for opening a leak in the membrane.

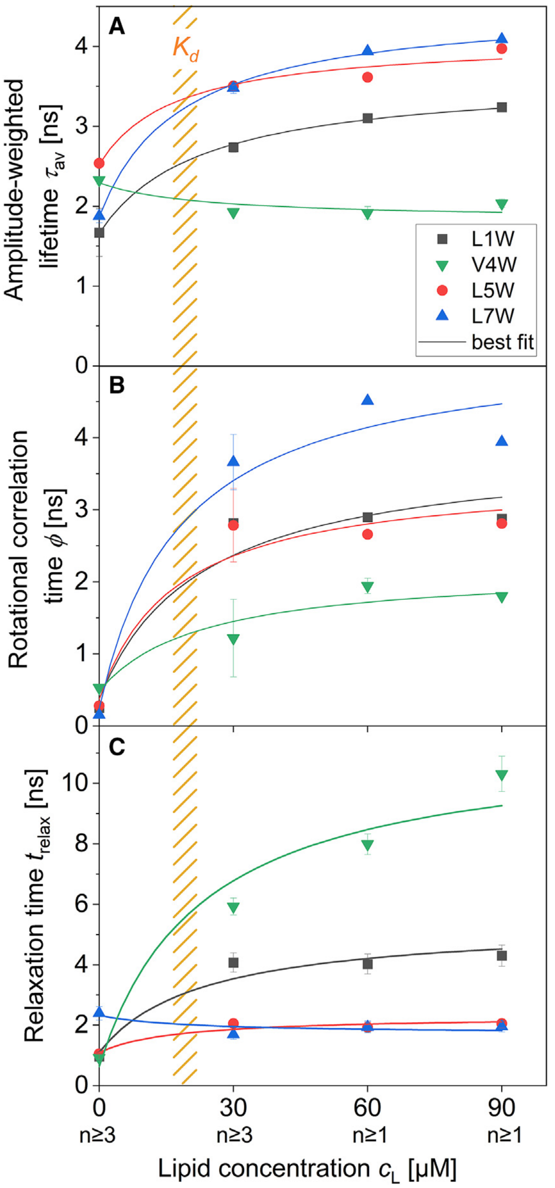

FIGURE 8 The dynamics of Trp fluorescence (*A*: amplitude-weighted lifetime, τ_{av}; *B*: rotational correlation time, φ; *C*: relaxation time, τ_{relax}) as a function of lipid concentration, c_L. The number of replicates is displayed under the respective *x*-axis label. The solid lines show the best fit calculated for a W-viscosin population distributed between the buffer and the membrane according to Eqs. 10 and 11. The orange shaded area marks the lipid concentration range at which half of the W-viscosins are bound to the membrane, the dissociation constant K_d. Error bars are standard errors of the mean of replicates. To see this figure in color, go online.

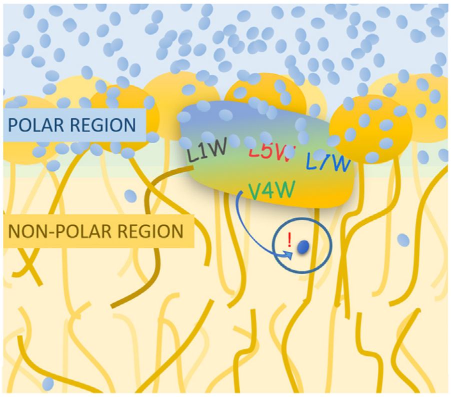

FIGURE 9 Schematic representation of the Trp inserted deeply into the hydrophobic core of the membrane being able to sense membrane disorder by differences of dynamics of diffusing water. The peptide body is marked by a tricolored oval. The color transition from blue to yellow indicates the separation of polar (*blue*) and non-polar (*yellow*) amino acids in this conformation of the peptide. The acyl chain is depicted as a brown line. The position of Trp is marked on the peptide with the exchange code (wild-type residue–position of the residue–mutant residue; L1W, *gray*; L5W, *red*; L7W, *blue*; V4W, *green*). The different regions of the membrane are labeled by the expected polarity of the environment, i.e., acyl chain region is non-polar (*core*) and the headgroup region is polar (*surface*). Blue ovals indicate water molecules. Trp position in V4W is located in the non-polar region of the peptide, and the membrane is able to sense diffusing water. The mobility of water in the membrane core changes with the membrane disorder. To see this figure in color, go online.

The fact that this cooperative perturbation is only seen for V4W does not mean it was special to this analog. Instead, it is only to be sensed in the most crucial region for membrane permeability—the most tightly packed hydrophobic region close to the interface—where Trp4 resides (illustrated in Fig. 9). This argument is already supported by the fact that leakage experiments do not suggest higher activity of V4W than of other W-viscosins. In addition, specific V4W-V4W interactions were ruled out to be crucial by a test showing that a progressive decrease in t_{relax} is not only sensed if further V4W is added from R_b of 0.08–0.13 but also if non-fluorescent wild-type viscosin is added to V4W (see Fig. ESI6 for details).

One may wonder why the concerted membrane perturbation by W-viscosins, which is to be considered a prerequisite for their leakage-inducing activity, is represented so sensitively by increased relaxation rates in the hydrocarbon region but neither by the local order and dynamics (r_∞, φ) nor the final extent of relaxation (ν_∞). When it comes to order and dynamics, this shows an interesting parallel to the lack of a disordering effect of another CLiP, surfactin, as studied by solid-state deuterium NMR of the acyl chains in selectively chain-deuterated lipids. Despite being described as a detergent-like molecule, surfactin did not disorder lipid chains in a detergent-like manner but caused a collective chain tilt accommodating the surfactin molecule without compromising order significantly (82). A similar behavior of the viscosin analogs could explain the lack of sensitivity of TR-anisotropy parameters to leakage-inducing perturbation. The lack of a concentration effect on ν_∞ can, again, be explained by the discreteness of the "recruitment of water" concept developed above. Stronger water penetration into the hydrophobic core and a faster motion of the water will speed up the recruitment of a water molecule to an excited Trp, i.e., reduce the t_{relax} of the Trp4's environment. However, as long as most Trps recruit only one water molecule, the extent of relaxation will stay the same.

W-viscosins in the buffer: V4W aggregates and Trp1 covered by acyl chain

The values at $c_L = 0$ in Figs. 4 and 8 have already revealed key properties of Trps in W-viscosins in the buffer as compared to the membrane-bound state. Mostly, they move faster (lower φ) and more freely (lower r_∞), and show a faster (short t_{relax}) and stronger (lower ν_∞) dipolar relaxation. All this is in line with what one expects for water-exposed Trp attached to a rather small molecule in solution, but some outliers indicate specific effects.

To provide more detailed insight, Fig. 10 *A* shows the relaxation level of W-viscosins in the buffer as a function of time. The relaxation level $\nu(t)$ is shown as the spectral center of gravity of TRES at distinct time points. Fig. 10 *B* shows the TR-FWHM of representative curves. Trp5 and Trp7 detect strong (lowest ν_∞), continuous (80), and homogeneous (only slightly and quickly decreasing FWHM) relaxation in line with exposure to bulk water in solution. The very fast bulk solvent relaxation of the buffer cannot be detected with our instrumentation due to instrumental limits (maximal resolution up to 5 ps in an ideal environment, expected resolution in a given environment at about 250 ps). The relaxation of water fixed loosely on the exterior of the peptide may happen gradually, from the nearest layer to the next molecular layer. This process, reported to take from several to tens of picoseconds in NMR and MD simulation studies (83), may produce a local maximum in TR-FWHM under ideal circumstances. With our temporal resolution limits, we expected to see no or only a small part (the right limb) of this maximum for all peptides.

Trp in V4W and L1W senses higher energy levels of ν_∞, indicating a less polar environment. Both their ν_∞ and FWHM show a minimum after a few nanoseconds and then increase again.

The rise after 2–3 ns in TR-FWHM of V4W is very high and highly constant. This leads to the assumption of a subpopulation in another, less polar environment than bulk solvent. This is consistent with the quenching of Trp lifetime in buffer. The τ_{av} of L1W, L5W, and L7W are indeed shorter in buffer than in the membrane, but this is not the case for V4W (Fig. 8 *A*). Thus, Trp in V4W is partially not exposed to water.

To gain additional clarity on this matter, the quenching experiments with acrylamide were performed with W-viscosins in buffer. The results are shown in Fig. 11. NATA is the maximally quenched, fully buffer-exposed control sample. Trp5 is quenched less than Trp7, probably due to the more efficient shielding of Trp by the peptide. Trp of L7W may have less possibility to bury itself into the peptide interior due to the flanking by two polar serins. Trp in L1W is least quenched and most shielded, likely due to the shielding effect of the acyl chain in its vicinity. Trp4 may be buried in the peptide, or multiple monomers may aggregate and shield V4W from the buffer.

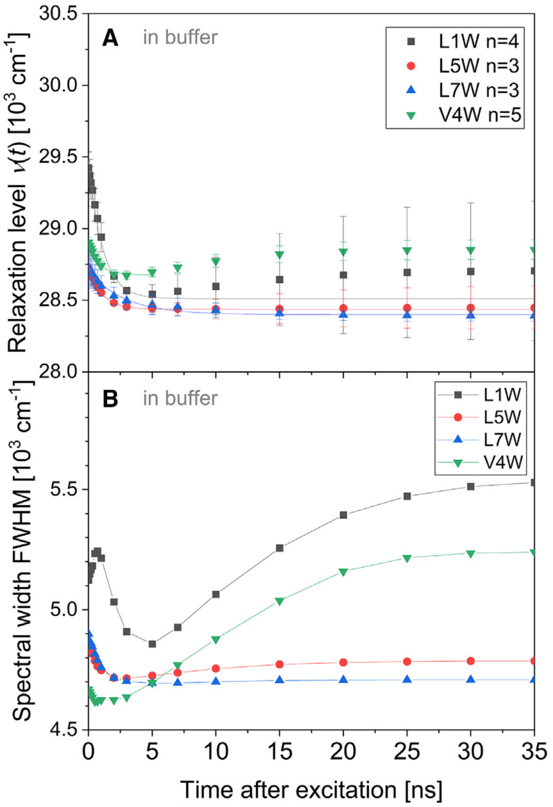

FIGURE 10 The relaxation process around W-viscosins in the buffer. (*A*) The relaxation level depicted as the spectral center of gravity of TRES is plotted as a function of time. Lines represent the monoexponential fit of data points. Note that V4W data could not be fitted monoexponentially, so only the data between 0 and 5 ns were considered. Note the high deviation between the fit and data for L1W. *n* indicates the number of independent replicates performed; error bars represent the corresponding standard deviation. (*B*) Exemplary data of the spectral width at the half-maximum intensity (FWHM; time-dependent change of the spectral width) as a function of time. Lines are to guide the eye only. The curves shown were chosen as the most representative ones, but the progression of FWHM varies from replicate to replicate. To see this figure in color, go online.

CONCLUSION

TR fluorescence of Trp as an intrinsic fluorophore or a supposedly conservative label (replacing a non-polar side chain) can provide a wealth of information about the interactions of a peptide with a membrane and specific conditions at the site of Trp. Whereas the interpretation of individual data is challenging given the complexity of Trp fluorescence, a combination of many observables of TCSPC measurements may give rise to a fairly consistent and clear picture. The findings obtained here may serve as a blueprint for interpreting parameters found for Trps in other membrane-binding peptides.

To illustrate the power of the approach, let us look at individual labels. For viscosin, probably the most informative label was Trp4, replacing a valine embedded within the acyl chain region of the membrane and,

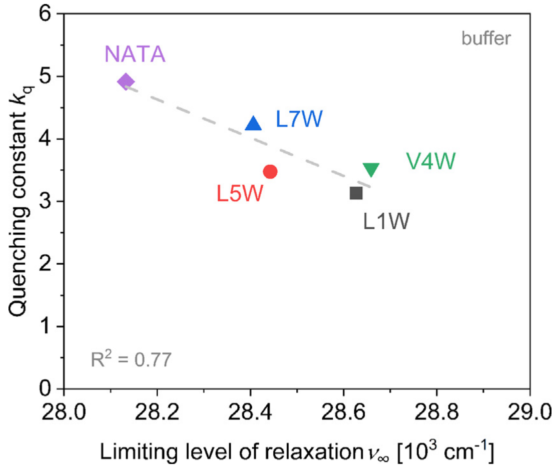

FIGURE 11 The apparent quenching rate constant, k_q, shown as a function of the spectral center of gravity after infinite time, ν_∞, for L1W (*gray squares*), V4W (*downward green triangles*), L5W (*red circles*), and L7W (*upward blue triangles*) in buffer. Gray dashed line represents linear fit of data points. Quality of the fit is represented by R^2. Data for NATA (*violet diamond*) was additionally included for the test for data correlation of the buffer measurements. To see this figure in color, go online.

apparently, without contact to lipid carbonyls or polar groups from the peptide. The extent of dipolar relaxation should allow comparison of the local membrane perturbation of different membranes. Even more importantly, the rate of relaxation of Trp4 was found to be sensitive to concentration-dependent, concerted membrane perturbation that appears to be a prerequisite for membrane leakage and antimicrobial action. A "water recruitment" hypothesis is presented to account for this observation: Trp4 residues that do not interact with any water molecule in the ground state will become more polar upon excitation but show dipolar relaxation only as a water molecule diffuses to their site.

Trp7 showed a very unusual relaxation behavior, being essentially independent of water reorientation. Instead, the residue is flanked by two Ser residues, which restrict its mobility. This seems to enforce some Trp7 to already assume an orientation in the ground state that becomes the relaxed orientation in the excited state. Trp-Ser interactions also seem to govern further relaxation.

Trp5, which is attached to the peptide backbone in a position located at the membrane surface, seems to be distributed between two main orientations. MD simulations favor the more membrane-embedded "down" orientation, but experimental data mainly reflect a highly water-exposed "up" orientation.

Trp1 shows a more standard behavior in the membrane—it interacts with water and lipid carbonyls and relaxes in a homogeneous, continuous fashion. Its proximity to a Glu residue did not result in any peculiar effects, but the adjacent attachment of the lipidic chain might give rise to a hydrophobic covering of Trp1 in solution.

SUPPORTING MATERIAL

Supporting material can be found online at https://doi.org/10.1016/j.bpj.2024.06.022.

AUTHOR CONTRIBUTIONS

I.C. and J.S. performed TR fluorescence-based research and analyzed the data. P.M. synthesized the W-viscosin under the supervision of A.M. and J.C.M. V.D.R. and N.G. performed and analyzed MD simulations and concentration determination under guidance and advice from J.C.M. I.C. and J.S. drafted the manuscript. H.H. initiated and supervised the work. H.H., I.C., N.G., and J.C.M. edited the manuscript. All authors read and approved the final manuscript.

ACKNOWLEDGMENTS

We acknowledge funding from FWO EOS G0G3118N (30650620), MEMCLiP BOF19-GOA-028 UGent (01G02819), and the Deutsche Forschungsgemeinschaft via the Research Training Group 2202 "Transport into and across membranes" (278002225/RTG 2202) and under Germany's Excellence Strategy (CIBSS-EXC2189-Project ID 390939984).

DECLARATION OF INTERESTS

The authors declare no competing interests.

REFERENCES

1. Amaro, M., R. Šachl, …, M. Hof. 2014. Time-Resolved Fluorescence in Lipid Bilayers: Selected Applications and Advantages over Steady State. *Biophys. J.* 107:2751–2760. https://doi.org/10.1016/j.bpj.2014.10.058.
2. Steigenberger, J., Y. Verleysen, …, H. Heerklotz. 2021. The Optimal Lipid Chain Length of a Membrane-Permeabilizing Lipopeptide Results From the Balance of Membrane Partitioning and Local Damage. *Front. Microbiol.* 12:669709–669714. https://doi.org/10.3389/fmicb.2021.669709.
3. Steigenberger, J., C. Mergen, …, H. Heerklotz. 2022. The Effect of Membrane Thickness on the Membrane Permeabilizing Activity of the Cyclic Lipopeptide Tolaasin II. *Front. Mol. Biosci.* 9:1064742. https://doi.org/10.3389/fmolb.2022.1064742.
4. Steigenberger, J., Y. Verleysen, …, H. Heerklotz. 2023. Complex Electrostatic Effects on the Selectivity of Membrane-Permeabilizing Cyclic Lipopeptides. *Biophys. J.* 122:950–963. https://doi.org/10.1016/j.bpj.2022.07.033.
5. Stulz, A., A. Vogt, …, M. Hoernke. 2019. Quantified Membrane Permeabilization Indicates the Lipid Selectivity of Membrane-Active Antimicrobials. *Langmuir.* 35:16366–16376. https://doi.org/10.1021/acs.langmuir.9b01849.
6. Shi, S., A. M. Markl, …, M. Hoernke. 2022. Interplay of Fusion, Leakage, and Electrostatic Lipid Clustering: Membrane Perturbations by a Hydrophobic Antimicrobial Polycation. *Langmuir.* 38:2379–2391. https://doi.org/10.1021/acs.langmuir.1c03445.
7. Shi, S., H. Fan, and M. Hoernke. 2022. Leaky Membrane Fusion: An Ambivalent Effect Induced by Antimicrobial Polycations. *Nanoscale Adv.* 4:5109–5122. https://doi.org/10.1039/D2NA00464J.
8. Heerklotz, H., and J. Seelig. 2007. Leakage and Lysis of Lipid Membranes Induced by the Lipopeptide Surfactin. *Eur. Biophys. J.* 36:305–314. https://doi.org/10.1007/s00249-006-0091-5.
9. Deleu, M., J. Lorent, …, M. P. Mingeot-Leclercq. 2013. Effects of Surfactin on Membrane Models Displaying Lipid Phase Separation.

Biochim. Biophys. Acta. 1828:801–815. https://doi.org/10.1016/j.bbamem.2012.11.007.

10. Deleu, M., J. M. Crowet, …, L. Lins. 2014. Complementary Biophysical Tools to Investigate Lipid Specificity in the Interaction between Bioactive Molecules and the Plasma Membrane: A Review. *Biochim. Biophys. Acta.* 1838:3171–3190. https://doi.org/10.1016/j.bbamem.2014.08.023.
11. Patel, H., C. Tscheka, and H. Heerklotz. 2009. Characterizing Vesicle Leakage by Fluorescence Lifetime Measurements. *Soft Matter.* 5:2849–2851. https://doi.org/10.1039/b908524f.
12. Nazari, M., M. Kurdi, and H. Heerklotz. 2012. Classifying Surfactants with Respect to Their Effect on Lipid Membrane Order. *Biophys. J.* 102:498–506. https://doi.org/10.1016/j.bpj.2011.12.029.
13. Sýkora, J., P. Kapusta, …, M. Hof. 2002. On What Time Scale Does Solvent Relaxation in Phospholipid Bilayers Happen? *Langmuir.* 18:571–574. https://doi.org/10.1021/la011337x.
14. Jurkiewicz, P., L. Cwiklik, …, M. Hof. 2012. Lipid Hydration and Mobility: An Interplay between Fluorescence Solvent Relaxation Experiments and Molecular Dynamics Simulations. *Biochimie.* 94:26–32. https://doi.org/10.1016/j.biochi.2011.06.027.
15. Först, G., L. Cwiklik, …, M. Hof. 2014. Interactions of Beta-Blockers with Model Lipid Membranes: Molecular View of the Interaction of Acebutolol, Oxprenolol, and Propranolol with Phosphatidylcholine Vesicles by Time-Dependent Fluorescence Shift and Molecular Dynamics Simulations. *Eur. J. Pharm. Biopharm.* 87:559–569. https://doi.org/10.1016/j.ejpb.2014.03.013.
16. Scollo, F., H. Evci, …, M. Hof. 2021. What Does Time-Dependent Fluorescence Shift (TDFS) in Biomembranes (and Proteins) Report On? *Front. Chem.* 9:738350. https://doi.org/10.3389/fchem.2021.738350.
17. Cosentino, M., C. Canale, …, A. Diaspro. 2019. AFM-STED Correlative Nanoscopy Reveals a Dark Side in Fluorescence Microscopy Imaging. *Sci. Adv.* 5:eaav8062–8. https://doi.org/10.1126/sciadv.aav8062.
18. Luitz, M. P., A. Barth, …, M. Zacharias. 2017. Covalent Dye Attachment Influences the Dynamics and Conformational Properties of Flexible Peptides. *PLoS One.* 12:e0177139. https://doi.org/10.1371/journal.pone.0177139.
19. Schröder, G. F., U. Alexiev, and H. Grubmüller. 2005. Simulation of Fluorescence Anisotropy Experiments: Probing Protein Dynamics. *Biophys. J.* 89:3757–3770. https://doi.org/10.1529/biophysj.105.069500.
20. Heijne, G. 1986. The Distribution of Positively Charged Residues in Bacterial Inner Membrane Proteins Correlates with the Trans-Membrane Topology. *EMBO J.* 5:3021–3027. https://doi.org/10.1002/j.1460-2075.1986.tb04601.x.
21. Schiffer, M., C. H. Chang, and F. J. Stevens. 1992. The Functions of Tryptophan Residues in Membrane Proteins. *Protein Eng.* 5:213–214. https://doi.org/10.1093/protein/5.3.213.
22. von Heijne, G. 2007. The Membrane Protein Universe: What's out There and Why Bother? *J. Intern. Med.* 261:543–557. https://doi.org/10.1111/j.1365-2796.2007.01792.x.
23. Yau, W.-M., W. C. Wimley, …, S. H. White. 1998. The Preference of Tryptophan for Membrane Interfaces. *Biochemistry.* 37:14713–14718. https://doi.org/10.1021/bi980809c.
24. de Planque, M. R. R., B. B. Bonev, …, J. A. Killian. 2003. Interfacial Anchor Properties of Tryptophan Residues in Transmembrane Peptides Can Dominate over Hydrophobic Matching Effects in Peptide−Lipid Interactions. *Biochemistry.* 42:5341–5348. https://doi.org/10.1021/bi027000r.
25. de Jesus, A. J., and T. W. Allen. 2013. The Role of Tryptophan Side Chains in Membrane Protein Anchoring and Hydrophobic Mismatch. *Biochim. Biophys. Acta.* 1828:864–876. https://doi.org/10.1016/j.bbamem.2012.09.009.
26. Ferreira, A. R., C. Teixeira, …, P. Gameiro. 2021. How Insertion of a Single Tryptophan in the N-Terminus of a Cecropin A-Melittin Hybrid Peptide Changes Its Antimicrobial and Biophysical Profile. *Membranes Basel.* 11:48. https://doi.org/10.3390/membranes11010048.
27. Feng, X., S. Jin, …, Y. Liu. 2020. The Critical Role of Tryptophan in the Antimicrobial Activity and Cell Toxicity of the Duck Antimicrobial Peptide DCATH. *Front. Microbiol.* 11:1146. https://doi.org/10.3389/fmicb.2020.01146.
28. Bi, X., C. Wang, …, D. Shang. 2013. Investigation of the Role of Tryptophan Residues in Cationic Antimicrobial Peptides to Determine the Mechanism of Antimicrobial Action. *J. Appl. Microbiol.* 115:663–672. https://doi.org/10.1111/jam.12262.
29. Choi, J., and E. Moon. 2009. Identification of Novel Bioactive Hexapeptides against Phytopathogenic Bacteria through Rapid Screening of a Synthetic Combinatorial Library. *J. Microbiol. Biotechnol.* 19:792–802.
30. Arias, M., L. T. Nguyen, …, H. J. Vogel. 2014. Position-Dependent Influence of the Three Trp Residues on the Membrane Activity of the Antimicrobial Peptide, Tritrpticin. *Antibiotics.* 3:595–616. https://doi.org/10.3390/antibiotics3040595.
31. van DUUREN, B. L. 1961. Solvent Effects in the Fluorescence of Indole and Substituted Indoles1. *J. Org. Chem.* 26:2954–2960. https://doi.org/10.1021/jo01066a079.
32. Sun, M., and P. S. Song. 1977. Solvent Effects on the Fluorescent States of Indole Derivatives-Dipole Moments. *Photochem. Photobiol.* 25:3–9. https://doi.org/10.1111/j.1751-1097.1977.tb07416.x.
33. Albani, J. R. 2014. Origin of Tryptophan Fluorescence Lifetimes Part 1 . Fluorescence Lifetimes Origin of Tryptophan Free in Solution. *J. Fluoresc.* 24:93–104. https://doi.org/10.1007/s10895-013-1277-8.
34. Laycock, M. V., P. D. Hildebrand, …, J. L. C. Wright. 1991. Viscosin, a Potent Peptidolipid Biosurfactant and Phytopathogenic Mediator Produced by a Pectolytic Strain of Pseudomonas Fluorescens. *J. Agric. Food Chem.* 39:483–489. https://doi.org/10.1021/jf00003a011.
35. Patel, S., S. Ahmed, and J. S. Eswari. 2015. Therapeutic Cyclic Lipopeptides Mining from Microbes: Latest Strides and Hurdles. *World J. Microbiol. Biotechnol.* 31:1177–1193. https://doi.org/10.1007/s11274-015-1880-8.
36. Schneider, T., A. Müller, …, H. Gross. 2014. Cyclic Lipopeptides as Antibacterial Agents – Potent Antibiotic Activity Mediated by Intriguing Mode of Actions. *Int. J. Med. Microbiol.* 304:37–43. https://doi.org/10.1016/j.ijmm.2013.08.009.
37. Geudens, N., and J. C. Martins. 2018. Cyclic Lipodepsipeptides From Pseudomonas Spp. – Biological Swiss-Army Knives. *Front. Microbiol.* 9:1867. https://doi.org/10.3389/fmicb.2018.01867.
38. Götze, S., and P. Stallforth. 2020. Structure, Properties, and Biological Functions of Nonribosomal Lipopeptides from Pseudomonads. *Nat. Prod. Rep.* 37:29–54. https://doi.org/10.1039/C9NP00022D.
39. Balleza, D., A. Alessandrini, and M. J. Beltrán García. 2019. Role of Lipid Composition, Physicochemical Interactions, and Membrane Mechanics in the Molecular Actions of Microbial Cyclic Lipopeptides. *J. Membr. Biol.* 252:131–157. https://doi.org/10.1007/s00232-019-00067-4.
40. Oni, F. E., Q. Esmaeel, …, M. Höfte. 2022. Pseudomonas Lipopeptide-Mediated Biocontrol: Chemotaxonomy and Biological Activity. *Molecules.* 27:372. https://doi.org/10.3390/molecules27020372.
41. Blake, C., M. N. Christensen, and Á. T. Kovács. 2021. Molecular Aspects of Plant Growth Promotion and Protection by Bacillus Subtilis. *Mol. Plant Microbe Interact.* 34:15–25. https://doi.org/10.1094/MPMI-08-20-0225-CR.
42. Ali, N., Z. Pang, …, H. R. El-Seedi. 2022. Lipopeptide Biosurfactants from Bacillus Spp.: Types, Production, Biological Activities, and Applications in Food. *J. Food Qual.* 2022:1–19. https://doi.org/10.1155/2022/3930112.
43. Penha, R. O., L. P. S. Vandenberghe, …, C. R. Soccol. 2020. Bacillus Lipopeptides as Powerful Pest Control Agents for a More Sustainable and Healthy Agriculture: Recent Studies and Innovations. *Planta.* 251:70. https://doi.org/10.1007/s00425-020-03357-7.
44. De Vleeschouwer, M., D. Sinnaeve, …, A. Madder. 2014. Rapid Total Synthesis of Cyclic Lipodepsipeptides as a Premise to Investigate Their Self-Assembly and Biological Activity. *Chem. Eur J.* 20:7766–7775. https://doi.org/10.1002/chem.201402066.
45. De Roo, V., Y. Verleysen, …, J. C. Martins. 2022. An Nuclear Magnetic Resonance Fingerprint Matching Approach for the Identification and Structural Re-Evaluation of Pseudomonas Lipopeptides. *Microbiol. Spectr.* 10:e0126122. https://doi.org/10.1128/spectrum.01261-22.
46. De Vleeschouwer, M., T. Van Kersavond, …, A. Madder. 2020. Identification of the Molecular Determinants Involved in Antimicrobial Activity

of Pseudodesmin A, a Cyclic Lipopeptide From the Viscosin Group. *Front. Microbiol.* 11:646. https://doi.org/10.3389/fmicb.2020.00646.

47. Wider, G., and L. Dreier. 2006. Measuring Protein Concentrations by NMR Spectroscopy. *J. Am. Chem. Soc.* 128:2571–2576. https://doi.org/10.1021/ja055336t.
48. Rubinson, K. A. 2017. Practical Corrections for p(H,D) Measurements in Mixed H2O/D2O Biological Buffers. *Anal. Methods.* 9:2744–2750. https://doi.org/10.1039/c7ay00669a.
49. Bartlett, G. R. 1959. Phosphorus Assay in Column Chromatography. *J. Biol. Chem.* 234:466–468. https://doi.org/10.1016/S0021-9258(18)70226-3.
50. Wieprecht, T., M. Beyermann, and J. Seelig. 1999. Binding of Antibacterial Magainin Peptides to Electrically Neutral Membranes: Thermodynamics and Structure. *Biochemistry.* 38:10377–10387. https://doi.org/10.1021/bi990913+.
51. Coraiola, M., P. Lo Cantore, …, I. Tolaasin. 2006. WLIP and tolaasin I, lipodepsipeptides from Pseudomonas reactans and Pseudomonas tolaasii, permeabilise model membranes. *Biochim. Biophys. Acta.* 1758:1713–1722. https://doi.org/10.1016/j.bbamem.2006.06.023.
52. Pfefferkorn, C. M., and J. C. Lee. 2012. 5-Fluoro-d,l-Tryptophan as a Dual NMR and Fluorescent Probe of α-Synuclein. *Methods Mol. Biol.* 895:197–209. https://doi.org/10.1007/978-1-61779-927-3_14.
53. Lakowicz, J. R., H. Cherek, …, E. Gratton. 1984. Time-Resolved Fluorescence Emission Spectra of Labeled Phospholipid Vesicles, as Observed Using Multi-Frequency Phase-Modulation Fluorometry. *Biochim. Biophys. Acta Biomembr.* 777:183–193. https://doi.org/10.1016/0005-2736(8490419-X.
54. Hutterer, R., A. B. J. Parusel, and M. Hof. 1998. Solvent Relaxation of Fluorescent Labels as a New Tool for the Detection of Polarity and Rigidity Changes in Membranes. *Czech. J. Phys.* 48:435–441. https://doi.org/10.1023/A:1021241808793.
55. Horng, M. L., J. A. Gardecki, …, M. Maroncelli. 1995. Subpicosecond Measurements of Polar Solvation Dynamics: Coumarin 153 Revisited. *J. Phys. Chem.* 99:17311–17337. https://doi.org/10.1021/j100048a004.
56. Fee, R. S., and M. Maroncelli. 1994. Estimating the Time-Zero Spectrum in Time-Resolved Emmsion Measurements of Solvation Dynamics. *Chem. Phys.* 183:235–247. https://doi.org/10.1016/0301-01049400019-0.
57. Jimenez, R., G. R. Fleming, …, M. Maroncelli. 1994. Femtosecond Solvation Dynamics of Water. *Nature.* 369:471–473. https://doi.org/10.1038/369471a0.
58. Stern, O., and M. Volmer. 1919. Über die Abklingzeit der Fluoreszenz. *Phys. Z.* 20:183–188.
59. Ladokhin, A. S., S. Jayasinghe, and S. H. White. 2000. How to Measure and Analyze Tryptophan Fluorescence in Membranes Properly, and Why Bother? *Anal. Biochem.* 285:235–245. https://doi.org/10.1006/abio.2000.4773.
60. Kemmer, G., and S. Keller. 2010. Nonlinear Least-Squares Data Fitting in Excel Spreadsheets. *Nat. Protoc.* 5:267–281. https://doi.org/10.1038/nprot.2009.182.
61. Dickson, C. J., B. D. Madej, …, R. C. Walker. 2014. Lipid14: The Amber Lipid Force Field. *J. Chem. Theor. Comput.* 10:865–879. https://doi.org/10.1021/ct4010307.
62. Maier, J. A., C. Martinez, …, C. Simmerling. 2015. Ff14SB: Improving the Accuracy of Protein Side Chain and Backbone Parameters from Ff99SB. *J. Chem. Theor. Comput.* 11:3696–3713. https://doi.org/10.1021/acs.jctc.5b00255.
63. Geudens, N., B. Kovács, …, J. C. Martins. 2019. Conformation and Dynamics of the Cyclic Lipopeptide Viscosinamide at the Water-Lipid Interface. *Molecules.* 24:1–20. https://doi.org/10.3390/molecules24122257.
64. Wu, E. L., X. Cheng, …, W. Im. 2014. CHARMM-GUI Membrane Builder toward Realistic Biological Membrane Simulations. *J. Comput. Chem.* 35:1997–2004. https://doi.org/10.1002/jcc.23702.
65. Jorgensen, W. L., J. Chandrasekhar, …, M. L. Klein. 1983. Comparison of Simple Potential Functions for Simulating Liquid Water. *J. Chem. Phys.* 79:926–935. https://doi.org/10.1063/1.445869.
66. Lee, T. S., D. S. Cerutti, …, D. M. York. 2018. GPU-Accelerated Molecular Dynamics and Free Energy Methods in Amber18: Performance Enhancements and New Features. *J. Chem. Inf. Model.* 58:2043–2050. https://doi.org/10.1021/acs.jcim.8b00462.
67. Humphrey, W., A. Dalke, and K. Schulten. 1996. VMD: Visual Molecular Dynamics. *J. Mol. Graph.* 14:33–38. https://doi.org/10.1016/0263-78559600018-5.
68. Roe, D. R., and T. E. Cheatham. 2013. PTRAJ and CPPTRAJ: Software for Processing and Analysis of Molecular Dynamics Trajectory Data. *J. Chem. Theor. Comput.* 9:3084–3095. https://doi.org/10.1021/ct400341p.
69. Geudens, N., M. De Vleeschouwer, …, D. Sinnaeve. 2014. Impact of a Stereocentre Inversion in Cyclic Lipodepsipeptides from the Viscosin Group: A Comparative Study of the Viscosinamide and Pseudodesmin Conformation and Self-Assembly. *Chembiochem.* 15:2736–2746. https://doi.org/10.1002/cbic.201402389.
70. Geudens, N., M. N. Nasir, …, M. Deleu. 2017. Membrane Interactions of Natural Cyclic Lipodepsipeptides of the Viscosin Group. *Biochim. Biophys. Acta Biomembr.* 1859:331–339. https://doi.org/10.1016/j.bbamem.2016.12.013.
71. Heerklotz, H. 2008. Interactions of Surfactants with Lipid Membranes. *Q. Rev. Biophys.* 41:205–264. https://doi.org/10.1017/S0033583508004721.
72. Nazari, M., H. Y. Fan, and H. Heerklotz. 2012. Effect of Hydrophobic Interactions on Volume and Thermal Expansivity as Derived from Micelle Formation. *Langmuir.* 28:14129–14136. https://doi.org/10.1021/la302276n.
73. Picardi, M. V., A. Cruz, …, J. Pérez-gil. 2011. Phospholipid Packing and Hydration in Pulmonary Surfactant Membranes and Fi Lms as Sensed by LAURDAN. *BBA - Biomembr.* 1808:696–705. https://doi.org/10.1016/j.bbamem.2010.11.019.
74. Caputo, G. A., and E. London. 2003. Using a Novel Dual Fluorescence Quenching Assay for Measurement of Tryptophan Depth within Lipid Bilayers To Determine Hydrophobic α-Helix Locations within Membranes. *Biochemistry.* 42:3265–3274. https://doi.org/10.1021/bi026696l.
75. Kleinschmidt, J. H., and L. K. Tamm. 1999. Time-Resolved Distance Determination by Tryptophan Fluorescence Quenching: Probing Intermediates in Membrane Protein Folding. *Biochemistry.* 38:4996–5005. https://doi.org/10.1021/bi9824644.
76. Moro, F., F. M. Goñi, and M. A. Urbaneja. 1993. Fluorescence Quenching at Interfaces and the Permeation of Acrylamide and Iodide across Phospholipid Bilayers. *FEBS Lett.* 330:129–132. https://doi.org/10.1016/0014-57939380257-U.
77. Cioni, P., and G. B. Strambini. 2002. Effect of Heavy Water on Protein Flexibility. *Biophys. J.* 82:3246–3253. https://doi.org/10.1016/S0006-34950275666-X.
78. Toptygin, D., R. S. Savtchenko, …, L. Brand. 2001. Homogeneous Spectrally- and Time-Resolved Fluorescence Emission from Single-Tryptophan Mutants of IIA Glc Protein. *J. Phys. Chem. B.* 105:2043–2055. https://doi.org/10.1021/jp003405e.
79. Beranová, L., J. Humpolíčková, …, M. Hof. 2012. Effect of Heavy Water on Phospholipid Membranes : Experimental Confirmation of Molecular Dynamics Simulations. *Phys. Chem. Chem. Phys.* 14:14516–14522. https://doi.org/10.1039/C2CP41275F.
80. Lakowicz, J. R. 2006. Principles of Fluorescence Spectroscopy.
81. Sýkora, J., P. Slavícek, …, M. Hof. 2007. Time-Dependent Stokes Shifts of Fluorescent Dyes in the Hydrophobic Backbone Region of a Phospholipid Bilayer: Combination of Fluorescence Spectroscopy and Ab Initio Calculations. *J. Phys. Chem. B.* 111:5869–5877. https://doi.org/10.1021/jp0719255.
82. Heerklotz, H., T. Wieprecht, and J. Seelig. 2004. Membrane Perturbation by the Lipopeptide Surfactin and Detergents as Studied by Deuterium NMR. *J. Phys. Chem. B.* 108:4909–4915. https://doi.org/10.1021/jp0371938.
83. Zhong, D., S. K. Pal, and A. H. Zewail. 2011. Biological Water: A Critique. *Chem. Phys. Lett.* 503:1–11. https://doi.org/10.1016/j.cplett.2010.12.077.

Biophysical Journal, Volume *123*

Supplemental information

Time-resolved fluorescence of tryptophan characterizes membrane perturbation by cyclic lipopeptides

Iulia Carabadjac, Jessica Steigenberger, Niels Geudens, Vic De Roo, Penthip Muangkaew, Annemieke Madder, José C. Martins, and Heiko Heerklotz

Electronic Supplementary Information to

Time-resolved fluorescence of tryptophan characterizes membrane perturbation by cyclic lipopeptides

Iulia Carabadjac[1#], Jessica Steigenberger[1#], Niels Geudens[2], Vic De Roo[2,3], Penthip Muangkaew[3], Annemieke Madder[3], José C. Martins[2], Heiko Heerklotz[1,4,5*]*

[1] Institute of Pharmaceutical Sciences, University of Freiburg, Hermann-Herder-Str. 9, 79104 Freiburg, Germany.[2] NMR and Structure Analysis Research Group, Department of Organic and Macromolecular Chemistry, Ghent University, Ghent, Belgium. [3] Organic and Biomimetic Chemistry Research Group, Department of Organic and Macromolecular Chemistry, Ghent University, Ghent, Belgium. [4] Center for Biological Signaling Studies (BIOSS), Faculty of Biology, University of Freiburg, Schänzlestr. 18, 79104 Freiburg, Germany. [5] Leslie Dan Faculty of Pharmacy, University of Toronto, 144 College Street, Toronto, ON, Canada M5s 3M2

Contents:

PART A: FLUORESCENCE

1. Chemical synthesis of viscosin and analogues

2. Time-resolved emission spectra (TRES) measurements

3. Reasons to use ν_∞ for TRES analysis

4. Time-resolved anisotropy measurements: Settings and procedure

5. Time-resolved quenching assay: Settings and procedure

6. Membrane permeabilization and kinetics by TCSPC: Settings and procedure

7. Membrane partitioning: Settings and procedure

8. DLS measurement results

9. Measurements with addition of viscosin

10. References part A

PART B: MOLECULAR DYNAMICS

11. Introduction

12. MD simulations of viscosin in POPC bilayer over longer simulation times, starting in different localizations

    250 ns MD simulation of 'wild-type' viscosin, starting in the aqueous phase

    500 ns MD simulation of 'wild-type' viscosin, starting at the bilayer-water interface

13. Impact of the starting location and orientation of viscosin within the bilayer

    50 ns MD simulation of viscosin initially at the bilayer-water interface

    50 ns MD simulation of viscosin initially in the interfacial region

    50 ns MD simulation of viscosin initially located at the center of the bilayer

    150 ns MD simulation of viscosin initially oriented upside down in the interfacial region

14. MD simulations of the tryptophan-labelled lipopeptides – supporting information

    MD simulation of L1W

    MD simulation of V4W

    MD simulation of L5W

    MD simulation of L7W

15. References part B

part A: Fluorescence

1. Chemical synthesis of viscosin and analogues

All procedures for the synthesis and purification of specific building blocks as well as solid-phase peptide synthesis, and cyclization release of the viscosin-analogs were carried out as described previously for the total synthesis of pseudodesmin A and its analogues[1].

2. Time-resolved emission spectra (TRES) measurements

Measurements were performed with the help of the TRES wizard tool. To this end, lifetime decay histograms of all samples were recorded for 25 s per nm over a detection wavelength range of 290 – 450 nm in 2 nm steps selected by Omni-λ300 Grating monochromator with an integration bin-width ranging from 5 to 10 nm. Trp was selectively excited at 280 nm with a repetition frequency of 20 MHz by a vertically polarized (V) VisUV laser. Excitation and detection attenuators were controlled by the TRES wizard (set to 100%) of the EasyTau software to yield reasonable detection count rates (<1% of sync rate) to avoid pile-up effects. Emission was detected by the PMA-C 175-M single-photon detector module with an emission polarizer set to 54.7° magic angle (M). The instrument response function (IRF) was acquired with a light scattering standard Ludox®-HS 40 (Sigma-Aldrich) solution (5 µl in 2 ml ultrapure water, width resolution ~50 ps) at 280 nm (VM polarization). Acquired lifetime decays were fit tri-exponentially by Equation ESI-1 with all lifetimes $\tau_1 - \tau_3$ set globally. Examples of fluorescence decays of all peptides are shown in Fig. ESI-1.

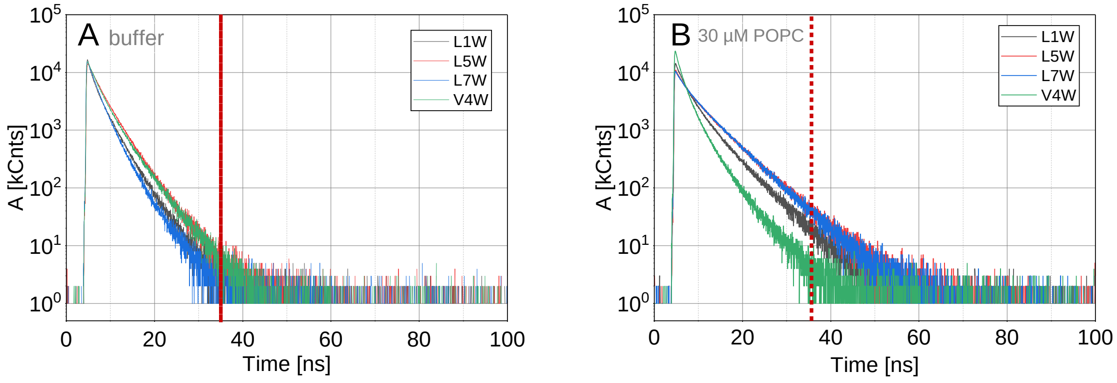

Figure ESI-1: Exemplary fluorescence decays of W-viscosins in buffer (A) and with 30 µM POPC-LUVs (B). The intensity A in kCnts is plotted as a function of time in ns. The red line marks the approximate fluorescence intensity level after 35 ns after excitation, which is used as the limit for time-resolved anisotropy and time-resolved spectral shift analysis. Note that at this time point the fluorescence is almost fully decayed but still not at the level of the dark count.

In the second step, the peak-normalized emission spectra at specific time points after excitation (TRES) were created using the fit data of the TR lifetime decays. For data analysis, it is important to include TRES at times long enough after excitation to ensure that the limiting spectral shift has been reached and recorded. However, the data must also be sufficiently well resolved. The temporal limit was set to 35 ns after excitation (Figure ESI-1). This time interval corresponds to five times the longest lifetime of the fluorophore (longest lifetime ~ 6-8 ns for all W-peptides) and with that to a time point, where almost all fluorescence is decayed, but the signal not yet equal to the dark count.

The variable $F(\lambda,t)$ represents the total integral counts at a certain wavelength and time, t, after the excitation pulse. A is the pre-exponential factor (amplitude) and τ_i is the decay time at a certain wavelength λ, respectively. No molecular significance is assigned to the components i. The number of components is increased until the fit doesn't become better (in this study three components were used, which corresponds to literature number for Trp lifetimes, if Trp interacts with the environment[2]).

$$F(\lambda,t) = \frac{H(\lambda) \cdot \Sigma_{\mathrm{i}} A_{\mathrm{i}}(\lambda) \cdot e^{-\frac{t}{\tau_{\mathrm{i}}}}}{CF(\lambda)} \qquad \text{ESI-1}$$

$F(\lambda,t)$ is corrected by the factor $H(\lambda)$, which is defined as $H(\lambda) = \frac{F_{SS}(\lambda)}{\Sigma_{\mathrm{i}} A_{\mathrm{i}}(\lambda) \cdot \tau_{\mathrm{i}}}$, so that the time-integrated intensity at each wavelength $I(\lambda)$ becomes equal to the steady state intensity $F_{ss}(\lambda)$ at that wavelength. Equation ESI-1 is normalized by a correction factor, CF, to account for the wavelength-dependent grating and detector sensitivity, $DS(\lambda)$. CF is calculated by $CF = \frac{I(\lambda)}{I(\lambda) \cdot DS(\lambda)}$.

Additionally, the amplitude-averaged lifetime was calculated according to:

$$\tau_{\mathrm{av}} = \frac{\sum_{\mathrm{i}=1}^{3} A_{\mathrm{i}} \cdot \tau_{\mathrm{i}}}{\sum_{\mathrm{i}=1}^{3} A_{\mathrm{i}}} \qquad \text{ESI-2}$$

where A denotes the integral of the amplitude over the whole spectrum.

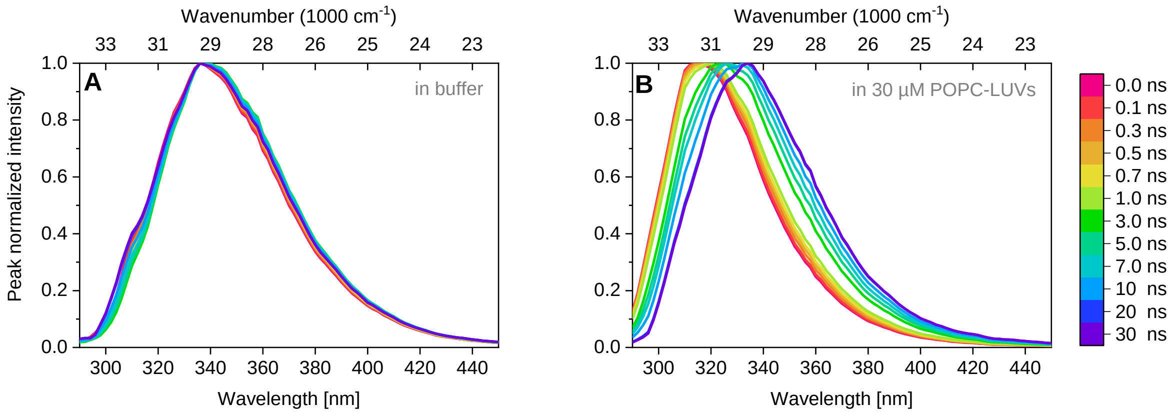

Figure ESI-2: Exemplary TRES of V4W in buffer (A) and with 30 µM POPC-LUVs (B). The peak normalized intensity is plotted as a function of emission wavelength (lower X-axes) or wavenumber (upper X-axes) at distinct time points from 0 up to 35 ns.

3. Reasons to use ν∞ for TRES analysis

$\nu(t)$ of all examined W-viscosins are shown in Figure ESI-3.

In general, $\nu(0)$ can be estimated by measurements of the absorption and emission spectra in completely non-polar solvents[3–5]. However, this was not possible for the W-viscosins for three reasons: First, Trp has two different, nearly isoenergetic excited states (^1L_a and ^1L_b) in the presence or absence of

H-bonding to N-H of the indole, hence two very different shapes of absorption and emission spectra in the polar and non-polar environment. Second, the W-viscosins tend to self-assemble in neat solvents with low dielectric constants, making it impossible to access $\nu(0)$ of the monomers apart from that of the aggregates, where Trp may be buried in the interior. Moreover, the estimation of $\nu(0)$ at low temperatures (below -60 °C) in not-crystallizing solvents such as glycerol or propylene glycol is not possible with available spectroscopic equipment in most laboratories. Thus, it is not possible to resolve the initial energy of the unrelaxed excited state by the measurements with our experimental setup. Furthermore, $\nu(0)$ may also differ for Trp in different positions in W-viscosin according to residues in its neighborhood, which would not provide us with any useful information about the environment of W-viscosin. Due to the uncertainty about $\nu(0)$, $\Delta\nu$ cannot be calculated for W-viscosins.

4. Time-resolved anisotropy measurements: Settings and procedure

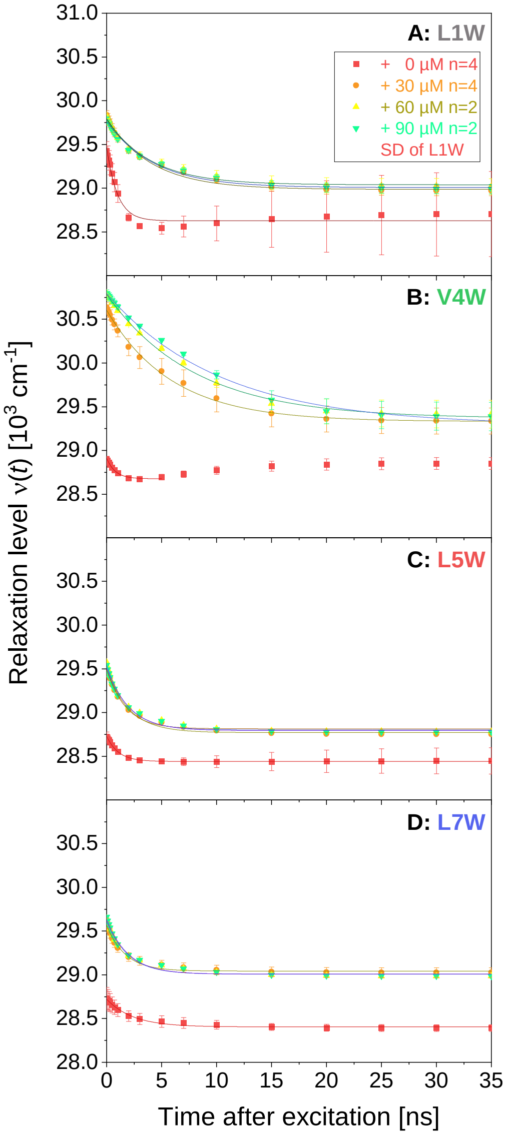

Figure ESI-3: The relaxation level showed as spectral center of gravity of TRES plotted as a function of time. Different panels show the results for L1W (A), V4W (B), L5W (C), and L7W (D) with 0 (red squares), 15 (orange circles), 30 (yellow triangles up), 60 (green triangles down), or 90 µM (blue diamonds) POPC-LUVs. Symbols represent actual data points. Lines represent mono-exponential fit of data points. The letter n indicates the number of independent replicates performed.

Time-resolved anisotropy (TR-anisotropy) was measured using the same instrumental setup as for TRES experiments. Emission was recorded at 340 nm. The emission polarizer was set to 0° (vertical, V), 54.7° (magic angle, M), and 90° (horizontal, H), respectively. To avoid fluorescence bleaching effects, a total number of six measurement cycles, accumulated for 5 s each, was performed. Measurements were performed using a script so that all data of a series and the G Factor were acquired with the same settings. The G Factor (correction for differences in the sensitivity of the detection system for vertically and horizontally polarized light) was determined at 340 nm emission wavelength for each series of experiments with NATA in TRIS buffer (absorption < 0.1 at 340 nm), a fluorophore solution displaying an anisotropy of ~ 0.

5. Time-resolved quenching assay: Settings and procedure

Fluorescence lifetime decays of W-viscosins and NATA in buffer or in 30 µM POPC LUVs at five concentrations of acrylamide (0, 0.1, 0.2, 0.3, 0.4 M for W-Viscosins and 0, 0.05, 0.1, 0.15, 0.2 M for NATA) were acquired after 4 to 8 h of incubation at 25°C. The fluorescence lifetime of Trp was recorded up to 10^4 peak counts. The resulting histograms were fitted globally over the 5 acrylamide concentrations with Equation ESI-3.

$$I(t) = \int_{-\infty}^{t} IRF(t') \sum_{\mathrm{i}=1}^{n_{\mathrm{Exp}}} A_{\mathrm{i}} \cdot e^{-\frac{t-t'}{\tau_{\mathrm{i}}}} \cdot dt' \qquad \text{ESI-3}$$

The intensity of fluorescence over time, denoted as $I(t)$, is expressed as a function of time, where t' represents the time shift of the Instrument Response Function (IRF) in nanoseconds. Time is represented as t, A_{i} is the pre-exponential factor of decay component i, τ_{i} denotes the lifetime of decay component i in nanoseconds, and n_{Exp} represents the number of exponential terms.

Decay parameters were adjusted iteratively until the reduced χ^2 reached its local minimum. Specific conditions were imposed, requiring all amplitudes to be greater than zero. The τ_{i} values were globally fitted and constrained to a fixed value. Uniform fitting was applied to all decays.

6. Membrane permeabilization and kinetics by TCSPC: Settings and procedure

The fluorescence lifetime decays of calcein were acquired with the FluoTime100 spectrometer (PicoQuant, Berlin, Germany) using TCSPC. Calcein was excited with a 467 nm pulsed-laser diode (LDH-P-C-470, PicoQuant, Berlin, Germany) operated by a PDL 800-D laser driver (pulse width: 20 ps; repetition rate: 20 MHz). Lifetime decays were recorded for 30 s (resolution of 25 ps per bin) using a PMA 175-N detector (PicoQuant, Berlin, Germany) and an OG530 long-pass filter. Detection attenuators were set to ensure a detection rate of < 1% of excitation pulses. The IRF was measured by a Ludox HS-40 solution (5 µl in 2 ml ultrapure water) and used for reconvolution fitting.

Peptide-induced membrane leakage does not lead to only two calcein populations. It is much more likely that all liposomes are damaged to a different extent and therefore lose different amounts of calcein. Experience has shown that a free biexponential fit provides the most reproducible and robust results. Therefore, decay curves were fitted biexponentially with the FluoFit software (PicoQuant) according to Equation ESI-4. The fluorescence lifetime of each population is described by τ. The exponential pre-factor B provides information about the amount of calcein present in each fraction. The subscripts E and F represent the encapsulated and free fractions of calcein, respectively.

$$B(t) = B_{\mathrm{E}} \cdot e^{-\frac{t}{\tau_{\mathrm{E}}}} + B_{\mathrm{F}} \cdot e^{-\frac{t}{\tau_{\mathrm{F}}}} \qquad \text{ESI-4}$$

7. Membrane partitioning: Settings and procedure

Measurements followed a common script to keep instrumental settings the same throughout all experiments. Samples were excited with the VisUV at 280 nm with the excitation attenuator set to position 5 (open) and excitation polarization to 0°. The laser pulse frequency was set to 80 MHz and laser intensity to 7.2. Emission was recorded with emission polarizer set to magic angle (54.7°), detection attenuator set to 100% in the wavelength range from 290 to 450 nm with a step width of 2.0 nm and an integration time of 1.0 s per point. All measurements were performed in two runs to exclude fluorescence bleaching effects. The fluorescence intensities of the W-viscosins (F_{W}) at respective lipid concentrations, c_{L}, were measured after 2-hour incubation time. All samples were incubated at 25°C, protected from light and stirred during incubation and measurement. The fluorescence intensity of TRIS buffer was measured as blank F_{B}. The fluorescence intensities of NATA were recorded immediately (< five minutes) upon lipid addition. F_{W} were corrected for F_{B} and the wavelength-dependent detector sensitivity (DS) by Equation ESI-5 to create the steady-state emission spectra of all samples, I_{W}.

$$I_{\mathrm{W}} = (F_{\mathrm{W}} - F_{\mathrm{B}}) \cdot DS \qquad \text{ESI-5}$$

Emission spectra of NATA samples were used to correct the emission spectra of W-viscosins at respective lipid concentrations, I'_{W} (c_{L}), for scattering light effects by Equation ESI-6.

$$I'_{\mathrm{W}}(c_{\mathrm{L}}) = I_{\mathrm{W}}(c_{\mathrm{L}}) \cdot \frac{I_{\mathrm{NATA}}}{I_{\mathrm{NATA}}(c_{\mathrm{L}})} \qquad \text{ESI-6}$$

The resulting spectra are shown in Figure ESI-5.

The partition coefficient of the W-viscosins between buffer and the POPC-LUVs was investigated. Here, the fact that the fluorescence intensity of tryptophan at any given wavelength correlates linearly with the amount of membrane-bound W-viscosin was exploited. In general, tryptophan membrane binding is accompanied by an increase in the fluorescence intensity and a blue shift of the emission[6–8]. However, light scattering has the opposite effect. Scattered light reduces the fluorescence intensity because it attenuates both the excitation and emission light. In addition, it can lead to an apparent red shift of the emission spectrum. For this reason, light scattering may obscure the effect of membrane binding of the CLiP on the emission spectrum[6]. To correct for these effects of scattered light, emission spectra of NATA samples with increasing lipid concentration were recorded in addition to emission spectra of samples with W-viscosins. It was assumed that NATA would not bind to the membrane. Surprisingly, the intensity of the emission spectra of NATA changed with incubation time. Samples after two hours incubation showed a biexponential lifetime decay and increasing fluorescence intensities with increasing lipid concentration, c_{L}, hence indications for binding of NATA to the membrane. Fluorescence data obtained immediately upon lipid addition and mixing did not reveal any signs of NATA binding to the membrane, as the fluorescence lifetime decay displayed a mono-

exponential decay with a constant lifetime independent of c_L. Moreover, the emission spectra showed the expected decrease in the fluorescence intensity with increasing c_L. Therefore, these data were used to subtract scattering light effects from the emission spectra of the W-viscosins.

8. DLS measurement results

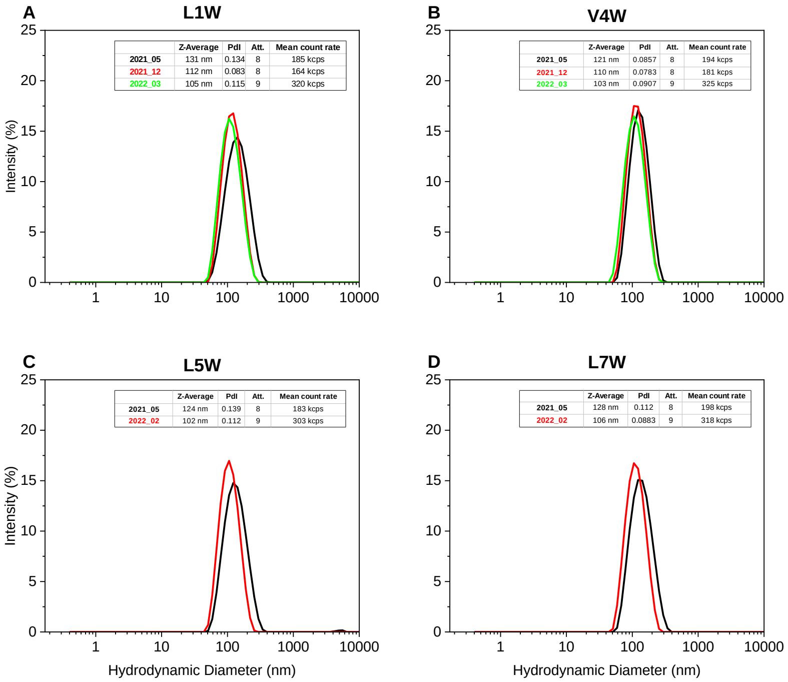

Figure ESI-4: DLS measurements of ~ 6.6 µM W-viscosins with 30 µM POPC-LUVs . The intensity distribution of samples with L1W (A), V4W (B), L5W (C) and L7W (D) are displayed. Tables list the Z-average of the hydrodynamic diameter, the polydispersity index (PDI), attenuator settings, and the mean count rate.

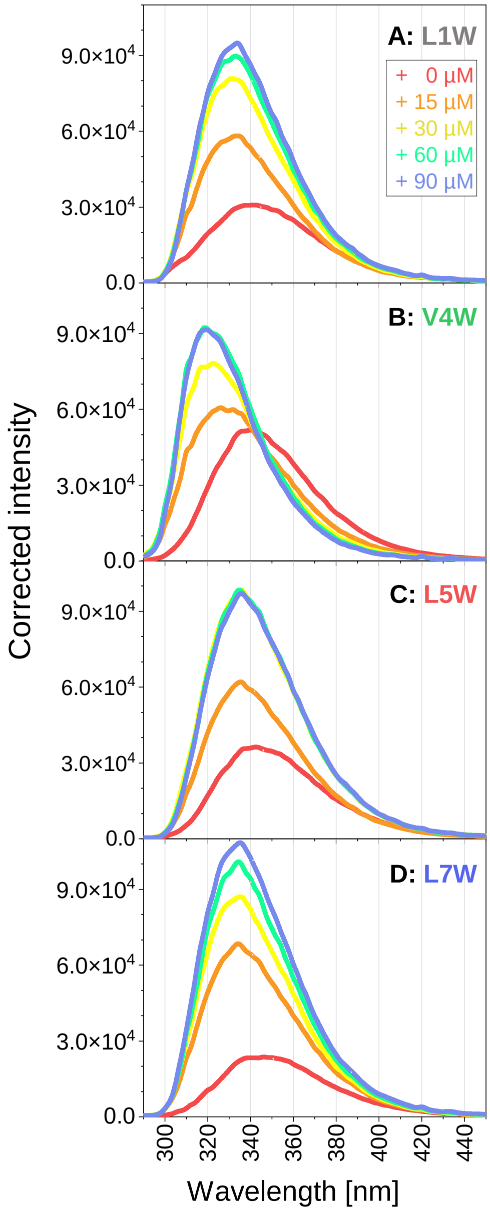

Figure ESI-5: The emission spectra of L1W (A), V4W (B), L5W (C), and L7W (D) with either 0 µM (red), 15 µM (orange), 30 µM (yellow), 60 µM (green), and 90 µM (blue) POPC-LUVs after 2 hours incubation time. The emission spectra are corrected for scattering light effects.

9. Measurements with addition of viscosin

Let us assume, that the increase in t_{relax} of V4W is indeed a result of some kind of specific peptide-peptide interaction in the membrane, which is possible for V4W, but not for the other W-viscosins. Then, the addition of other W-viscosins to the V4W-sample should disorder the membrane further, but not increase the amount of specific V4W-V4W interactions. For t_{relax} depending *on the specific peptide-peptide interaction* nothing should change. For t_{relax} depending on *the membrane order*, the value should decrease.

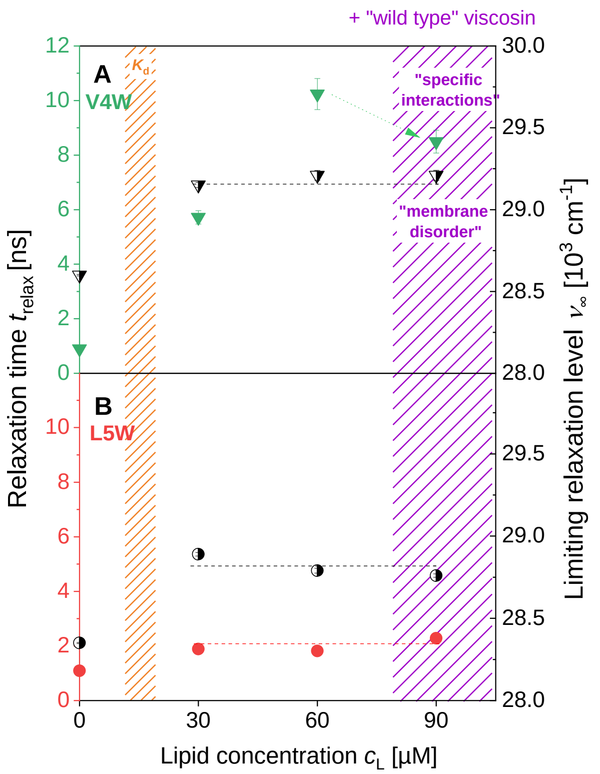

*Figure ESI-6: **A** shows on the left Y-axes the dipolar relaxation time, t_{relax}, (green triangles down) of V4W in buffer, at 30 µM lipid (P/L~1:5), 90 µM lipid (P/L~1:14), and at 90 µM lipid with the same amount of V4W + "wild type" viscosin up to a of P/L~1:5 (equivalent to V4W at 30 µM lipid alone). The right Y-axes displays the corresponding limiting level of relaxation depicted as the spectral center of gravity of TRES, ν_∞ (black triangles half-filled). **B** shows the results for L5W samples (red and black filled and half-filled circles, respectively). The violet background indicates the sample with "wild-type" viscosin added. The orange shaded area marks the lipid concentration range at which half of the W-viscosins are bound to the membrane, the dissociation constant K_d. All lines and arrows are to guide the eye only. The text boxes in A indicate the areas, where t_{relax} would have been expected after "wild-type" viscosin addition to the sample for the different hypotheses of the origin of t_{relax} change upon changes in concentration. The upper text box marks the area expected for specific peptide-peptide interaction; the lower box indicates where t_{relax} would have been if viscosin had been as membrane-active as V4W and led to the same amount of membrane disorder.*

Instead of W-viscosins, the "wild-type" viscosin was used. Viscosin has the advantage of being "spectroscopically silent" because no fluorescent residues are present, but still being membrane-active. This means that the addition of viscosin to V4W will not disturb the fluorescence signal from Trp of V4W. Since viscosin is less membrane perturbing than V4W (Figure 2 of main manuscript) a slighter decrease of t_{relax} than to the level of V4W is expected. Note that because of slightly different K_d of viscosin (~ 3 µM[9]) and V4W (~ 20 µM) the lipid to peptide ratio of L/P = 5 isn't the same for V4W

alone or V4W and viscosin. As a control, we added the same amount of viscosin to L5W samples as well. The impact of the non-fluorescent viscosin on t_{relax} is shown in Figure ESI-6 for both W-viscosins (A: V4W, B: L5W). The observed decrease in t_{relax} of V4W, but not for L5W, supports our hypothesis about t_{relax} being independent of specific peptide-peptide interactions.

10. References

(1) De Vleeschouwer, M.; Van Kersavond, T.; Verleysen, Y.; Sinnaeve, D.; Coenye, T.; Martins, J. C.; Madder, A. Identification of the Molecular Determinants Involved in Antimicrobial Activity of Pseudodesmin A, a Cyclic Lipopeptide From the Viscosin Group. *Front. Microbiol.* **2020**, *11* (April), 1–14. https://doi.org/10.3389/fmicb.2020.00646.

(2) Ghisaidoobe, A.; Chung, S. Intrinsic Tryptophan Fluorescence in the Detection and Analysis of Proteins: A Focus on Förster Resonance Energy Transfer Techniques. *Int. J. Mol. Sci.* **2014**, *15* (12), 22518–22538. https://doi.org/10.3390/ijms151222518.

(3) Horng, M. L.; Gardecki, J. A.; Papazyan, A.; Maroncelli, M. Subpicosecond Measurements of Polar Solvation Dynamics: Coumarin 153 Revisited. *J. Phys. Chem.* **1995**, *99* (48), 17311–17337. https://doi.org/10.1021/j100048a004.

(4) Fee, R. S.; Maroncelli, M. Estimating the Time-Zero Spectrum in Time-Resolved Emmsion Measurements of Solvation Dynamics. *Chem. Phys.* **1994**, *183* (2–3), 235–247. https://doi.org/10.1016/0301-0104(94)00019-0.

(5) Jimenez, R.; Fleming, G. R.; Kumar, P. V.; Maroncelli, M. Femtosecond Solvation Dynamics of Water. *Nature* **1994**, *369* (6480), 471–473. https://doi.org/10.1038/369471a0.

(6) Ladokhin, A. S.; Jayasinghe, S.; White, S. H. How to Measure and Analyze Tryptophan Fluorescence in Membranes Properly, and Why Bother? *Anal. Biochem.* **2000**, *285* (2), 235–245. https://doi.org/10.1006/abio.2000.4773.

(7) Lakowicz, J. R. *Principles of Fluorescence Spectroscopy*; 2006.

(8) Yammine, A.; Gao, J.; Kwan, A. H. Tryptophan Fluorescence Quenching Assays for Measuring Protein-Ligand Binding Affinities: Principles and a Practical Guide. *bio-protocol* **2019**, *9*. https://doi.org/10.21769/BioProtoc.3253.

(9) Steigenberger, J.; Verleysen, Y.; Geudens, N.; Madder, A.; Martins, J. C.; Heerklotz, H. Complex Electrostatic Effects on the Selectivity of Membrane-Permeabilizing Cyclic Lipopeptides. *Biophys. J.* **2023**, *122* (6), 950–963. https://doi.org/10.1016/j.bpj.2022.07.033.

part B: Molecular Dynamics

Summary: The lipopeptide MD simulations were analyzed through comprehensive monitoring of total energy, temperature, and density across the MD simulations. Additionally, the stability and convergence of the membrane bilayer were evaluated using the "area per lipid" metric, complemented by monitoring of RMSD and hydration patterns around each residue's alpha carbon. The starting location and orientation for the lipopeptide in each simulation was chosen based on a long duration MD simulation (250 ns) of the insertion of viscosin from the aqueous phase into a pre-equilibrated and solvated POPC membrane. Starting from the inserted position, a second 500 ns MD simulation revealed a stable disposition with respect to the POPC membrane without significant fluctuations in location or orientation. Therefore, all simulations starting from the same initial position as used in this work were limited to 100 ns. Test simulations where viscosin was placed at different positions in the POPC membrane (phosphatidyl head group, glycerol backbone or POPC bilayer center) demonstrated viscosin adopts the same final disposition well within the first 50 ns of the simulation time. A simulation starting from an upside down orientation (i.e. hydrophobic side chains turned to the aqueous phase) showed that reorientation to the expected disposition is complete within 80 ns. Thus, these simulations revealed that 100 ns simulation time is sufficient to capture the dynamics of the system, and bias linked to the initial location and orientation of the lipopeptide is suitably addressed.

11. Introduction

Original MD simulations as described in the manuscript for all W-viscosins (Trp-analogues of viscosin) were 100 ns in duration with the various W-viscosin analogues initially positioned with the same orientation and with its center at the level of the glycerol backbone, referred to as the interfacial region hereafter. The position and orientation was based on previous experience with simulating cyclic lipodepsipeptides in house, and is substantiated hereafter (section 2). The procedures used are described in the materials and methods section of the original and now reworked manuscript. Relevant information with respect to all MD simulations described in the manuscript are collected in section 4 and could be included as sup. mat in the reworked manuscript, if so desired.

Section 2 of this document presents two longer duration MD simulations using ‘wild-type’-viscosin. In a first, 250 ns simulation, viscosin was placed well above the pre-equilibrated and solvated POPC bilayer, and allowed unforced insertion, which occurred around 100 ns into the simulation. A second, 500 ns simulation starts from viscosin inserted at the bilayer-water interface in the same way as described above for the Trp-viscosin analogues, i.e. with the molecular center positioned at the level of the phosphatidylcholine head groups and an orientation fitting amphipathic interaction expectations. The simulation time was extended from 100 ns to 500 ns to allow 450 ns of trajectory monitoring, as opposed to the 50 ns monitoring in the original MD simulations of the Trp-viscosin analogues, thus allowing to assess peptide-bilayer behavior over a nine-fold longer observation period and potential bias towards the initial conditions. During the extended simulation time, no additional events of significance are seen to occur compared to the shorter MD simulation.

Section 3 reports on 50 ns simulations on wt-viscosin, where its location within the bilayer was varied (3.1-3.3) or it was turned upside down at the interfacial region (3.4), thus allowing to establish the relaxation time required for viscosin to reach a stable location and orientation within the bilayer.

For each simulation trajectory, a variety of data as a function of the simulation time is extracted and collected hereafter. The system's (i) total energy, (ii) temperature and (iii) density is shown to demonstrate the evolution and stability of simulation parameters; (iv) the area per lipid metric is utilized to evaluate bilayer stability; (v) the total number of hydration water molecules is obtained by summing the number of water molecules within a 5 Å sphere of each Cα carbon, including that of the acyl chain while (vi) the heavy atom RMSD (including the acyl chain) of the lipopeptide with respect to its structure at t = 0 is used to assess conformational stability; (vii) the water exposure of individual Cα carbons within a 5 Å shell averaged over the simulation trajectory is graphed as a normalized average ± s.d. (1.0 = for residue with highest exposure – see also below), while (viii) the electron density along the z axis of the simulation box (averaged over the final 50 ns (section 2) or 10 ns (section 3) of the simulation) as a function of distance from the bilayer center for the phosphocholine (PC) and oleoyl- and palmitoyl (OL, PA) aliphatic chains and for the peptide.

With respect to the time-averaged water exposure graphs, we previously found that the water exposure values for individual Cα carbons shows a good to excellent fit with experimental NMR data gauging water exposure via paramagnetic relaxation probes[10]. More specifically, viscosinamide, the natural Gln2 analogue of viscosin (having Glu2) was investigated in DPC micelles and in the presence of water-soluble paramagnetic relaxation agents (=PRA; GdDTPA was used) using NMR spectroscopy. The PRAs quench ^{1}H resonance intensity to an extent that depends on the average proximity of the PRA to the ^{1}H monitored. As the PRAs are only soluble in the aqueous phase and do not associate or dissolve in DPC the quenching occurs to a degree that reflects relative exposure to these PRAs and therefore to the water phase. The figure 6 in Geudens *et al* [10] shows a confrontation of the degree of quenching of CαH, represented in a normalized fashion, with the normalized exposure of individual amino acids to water molecules within a 5 Å shell of their the Cα as collected from the final 200 ns unrestrained MD simulations of viscosinamide in DPC micelles in the AMBER environment. A good to excellent match is found, a finding which could also be extended to larger lipopeptide structures (unpublished data). Clearly, Gln2 and Ser6, occurring on the same face of the helix, are exposed to the PRA agent, while Val4 is buried towards the bilayer center and has little if any exposure, indicating an average location in accordance to expectations for an amphipathic helical structure. Taken all together, we are confident that the water exposure extracted from MD simulations can be used as a good indicator of overall orientation of a peptide with respect to the micelle- and by extension bilayer-water interface.

Finally, snapshots of the simulation are provided where appropriate. All together we propose from the data provided that the initial location and orientation of viscosin and its derivatives chosen during all simulations does not lead to significant bias when focusing analysis on the final 50 ns of a 100 ns MD simulation, starting from lipopeptides located with their center at the level of the phosphatidylcholine head groups and expected amphipathic orientation.

12. Assessing the impact of the starting position of viscosin with respect to the POPC bilayer

All simulations in this section were performed using the same protocol, the only difference being the initial location of viscosin (aqueous vs. bilayer-water interface) and duration (250 ns vs. 500 ns respectively) of the simulation. These simulations allow to assess possible bias of the results towards the initial conformation and, once bilayer associated, the minimal time required to effectively sample the behavior of viscosin at this interface. The simulations described below show from a fully solvated state in the aqueous phase, viscosin positions itself with respect to the bilayer as can be expected from

its amphipathic character. It also shows a highly similar trajectory for the bilayer associated state irrespective of the initial position. Both simulations show that, starting from a bilayer associated initial state, extension of the trajectory from 100 ns to 500 ns does not introduce new events as to the behavior of viscosin, indicating that possible kinetic trapping close to the initial conformation is unlikely (see also section 3). As such, these simulations thus address concerns regarding the impact of the initial state, and the simulation time used to describe the behavior of viscosins within the solvated and pre-equilibrated POPC bilayer, notably establishing whether kinetic trapping.

MD simulation of 'wild-type' viscosin, starting in the aqueous phase

This MD simulation was conducted with wild-type viscosin situated in the aqueous phase, not interacting with the solvated POPC bilayer. Figures 1-3 depict the stability of simulation parameters (total energy, temperature, density) over time. The "area per lipid" metric was utilized to evaluate membrane stability and convergence (Figure 4). The simulation, intended for to last 250 ns was cut short to ~225 ns due to an unplanned interruption, and not restarted as no evolution was apparent, while insertion had already taken place. Approximately 94 ns into the simulation, the 9-amino acid lipopeptide integrated itself into the membrane bilayer, as clearly evident from the reduction in water exposure (Figure 5). The fluctuations in RMSD during insertion of viscosin is similar to those in the period before insertion indicating the conformation is not significantly impacted during insertion (Figure 6). Analysis of hydration around each residue's alpha carbon offered insights into the lipopeptide's orientation and insertion depth (Figure 7), a validation previously demonstrated with a closely related lipopeptide, viscosinamide A. From this analysis, we conclude that viscosin integrates into the membrane bilayer remaining close to the surface, with Val4 being the most shielded from the aqueous phase. Lastly, electron density analysis, also employed in the manuscript, establishes the peptide's distance from the membrane center at 13.5 ± 3.8 Å. (Figure 8). From the electron density distribution of the peptide vs. the bilayer in the final 50 ns, we can see that the peptide is located within the interfacial region, i.e. at the level of the glycerol backbone between the lipid chains and the phosphatidylcholine head groups (place where the black and grey lines in the top graph intersect).

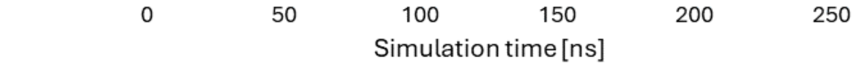

Figure 1: Total energy of the simulation as a function of simulation time.

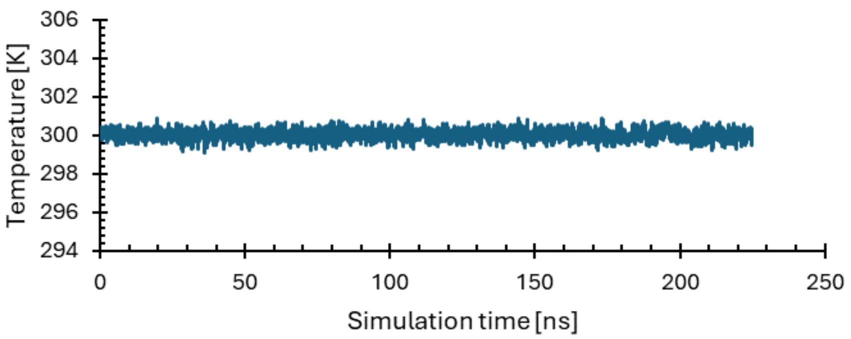

Figure 2: Temperature as a function of simulation time.

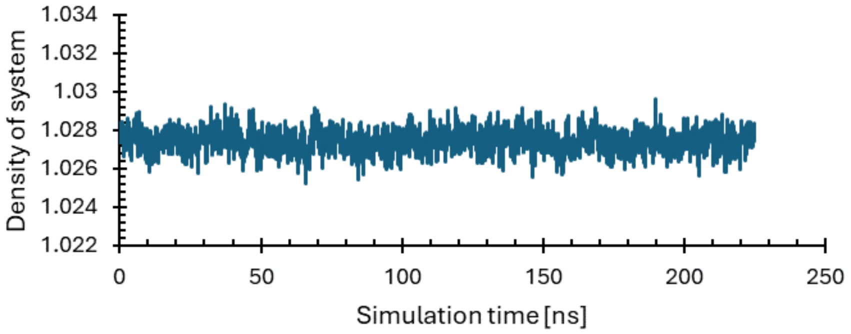

Figure 3: Density of the system as a function of simulation time.

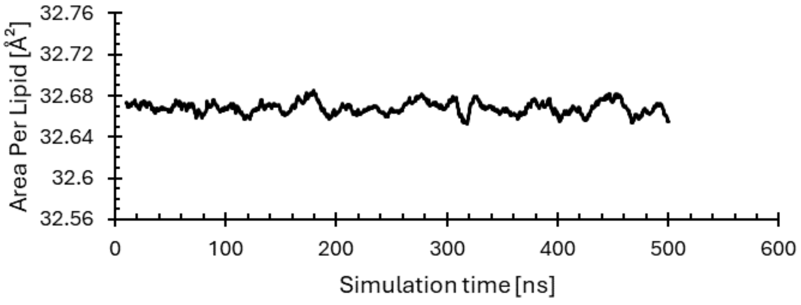

Figure 4: Area per lipid as a function of simulation time.

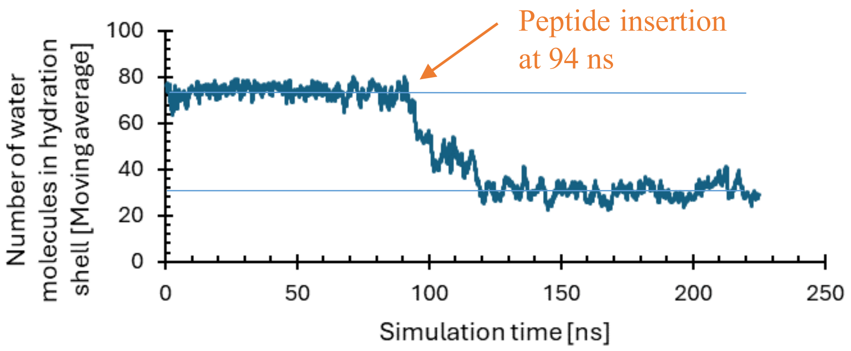

Figure 5: Total number of water molecules surrounding all viscosin alpha carbons within a 5 Å sphere as a function of simulation time.

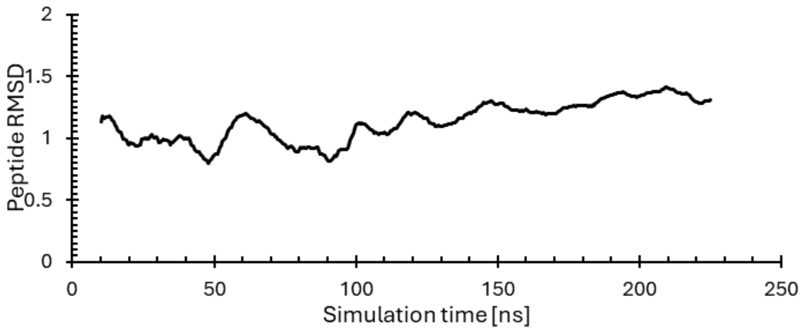

Figure 6: Heavy atom Root-Mean-Square-Deviation (RMSD) of the complete lipopeptide as a function of simulation time with respect to the initial structure.

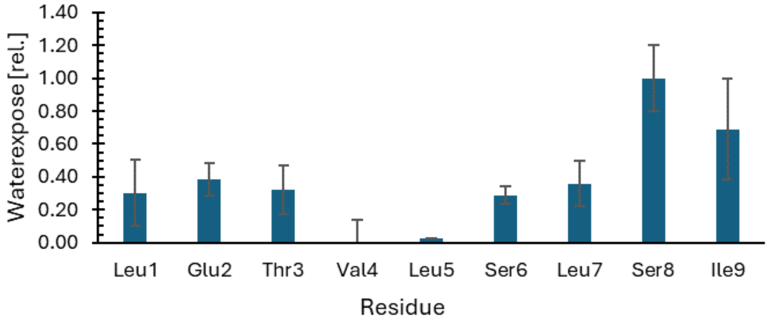

Figure 7: Normalized water exposure of the individual alpha carbons of the lipopeptide .

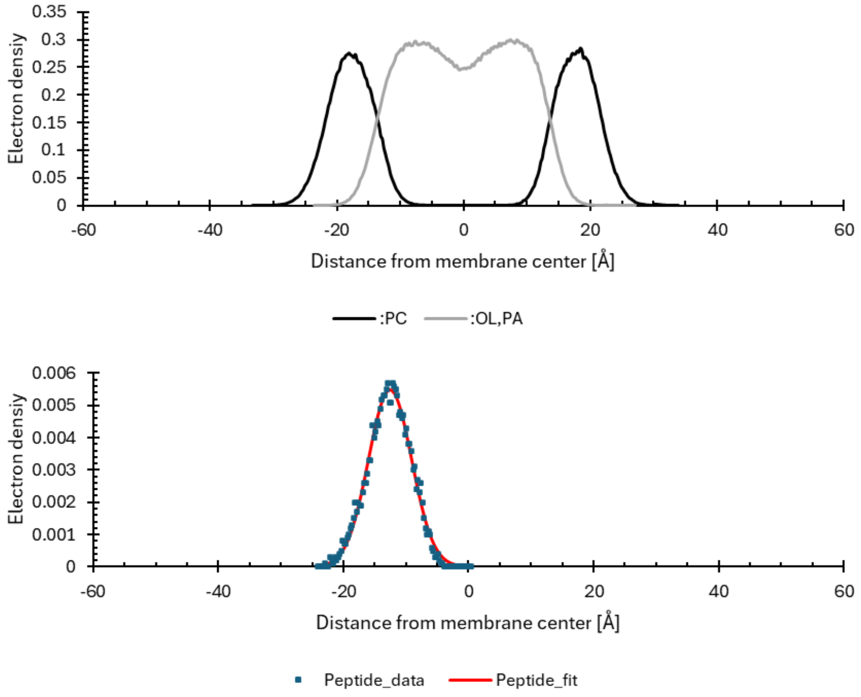

Figure 8: Electron density along the z axis of the simulation box (averaged over the final 50 ns of the simulation), as a function of distance from membrane center. Top: electron density of the phosphocholine (PC) and oleoyl- and palmitoyl (OL, PA) aliphatic chains of the POPC bilayer. Bottom: Electron density calculated for of the peptide (datapoints) and it's fitted Gaussian function (red curve).

Snapshots of the MD trajectory illustrating the system at t = 0 and at 10 ns interval between 90 ns and 130 ns during insertion of viscosin into to bilayer:

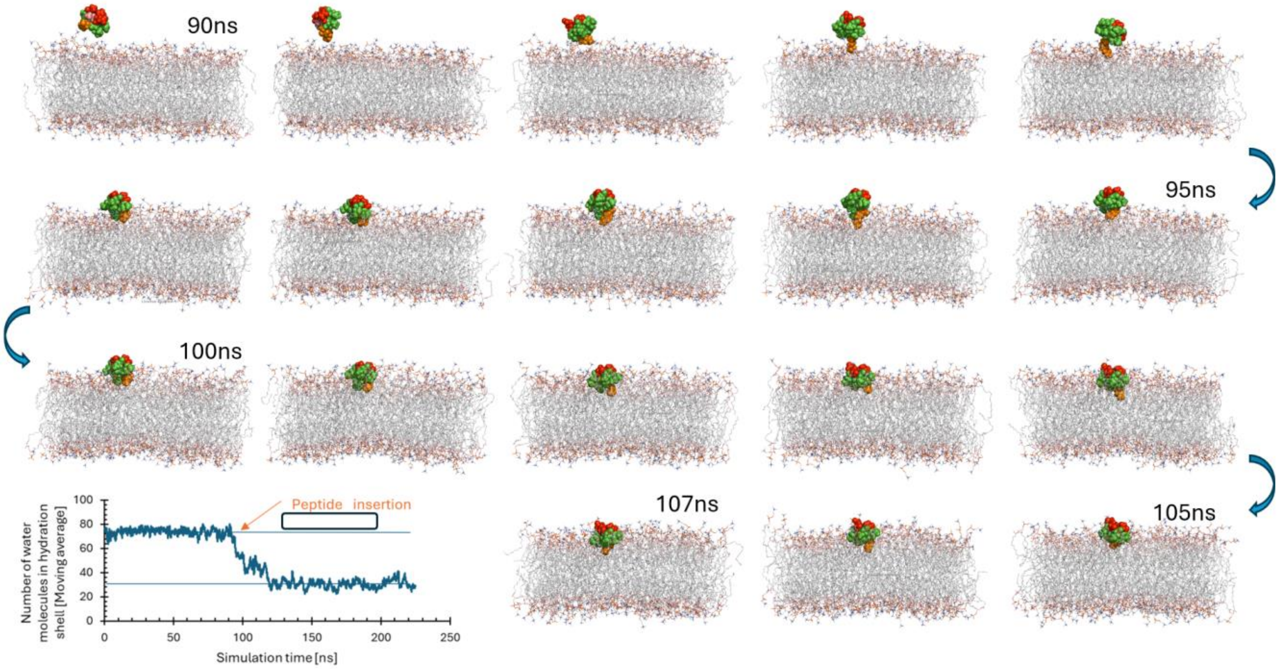

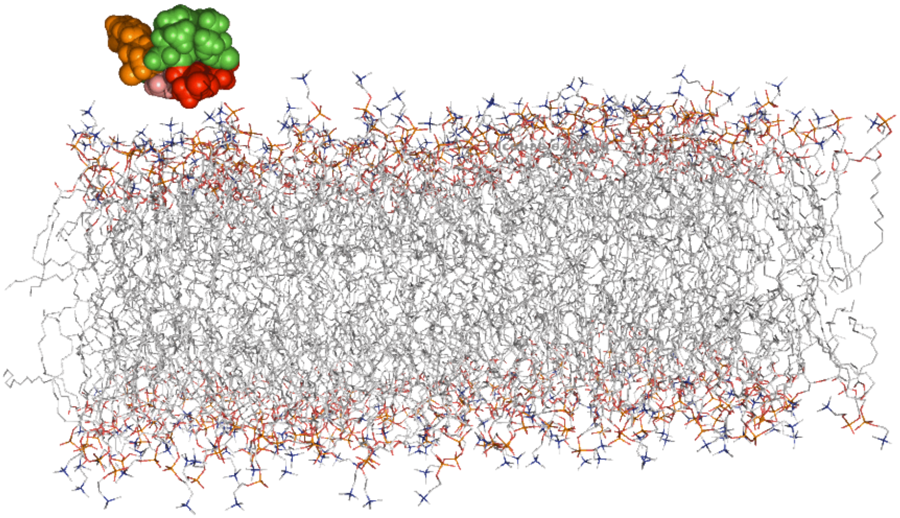

MD simulation of 'wild-type' viscosin, starting at the membrane interface

A second MD simulation was performed for wild-type viscosin positioned at the interface between the aqueous and membrane phases, i.e. with the molecular center at the level of the phosphatidylcholine head group. This positioning was also used for the four tryptophan analogues as described in the manuscript. To sample the evolution in the disposition with respect to the POPC bilayer, the simulation

duration was extended to 500 ns. As before, figures 9-11 demonstrate stability of total energy, temperature, and density over the trajectory. Here again, the "area per lipid" served as a metric for assessing membrane stability and convergence, remaining stable throughout the simulation (Figure 12). From the electron density distribution of the peptide vs. the bilayer, we can see that here also, the peptide is seen to be located within the interfacial region, i.e. at the level of the glycerol backbone between the lipid chains and the phosphatidylcholine head groups (place where the black and grey lines in the top graph intersect). Electron density analysis establishes the peptide's distance from the membrane center at 15.2 ± 3.4 Å (Figure 16). The number of water molecules hydrating the alpha carbons mirrored the results from the previous simulation (section 2.1) starting with viscosin in the aqueous phase (Figure 13). The RMSD of the lipopeptide also shows a constant fluctuation around the same value throughout the simulation, as before (Figure 14). Furthermore, quantitative analysis of hydration around each residue's alpha carbon reiterated that viscosin is surface-bound on the membrane bilayer (Figure 15), with Val4 most shielded from the aqueous phase, consistent with the simulation where viscosin was initially placed in the aqueous phase.

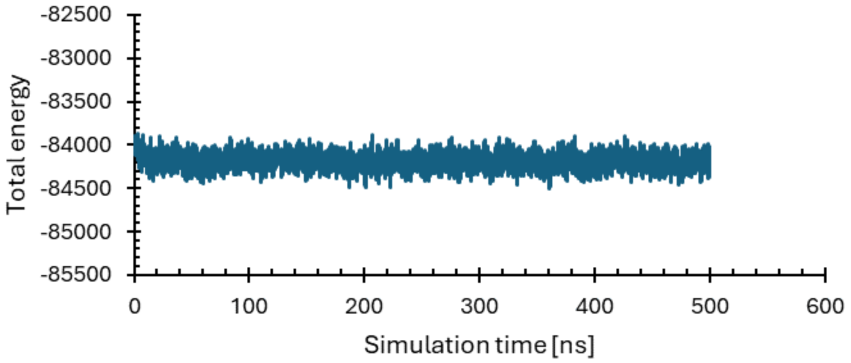

Figure 9: Total energy of the simulation, as a function of simulation time.

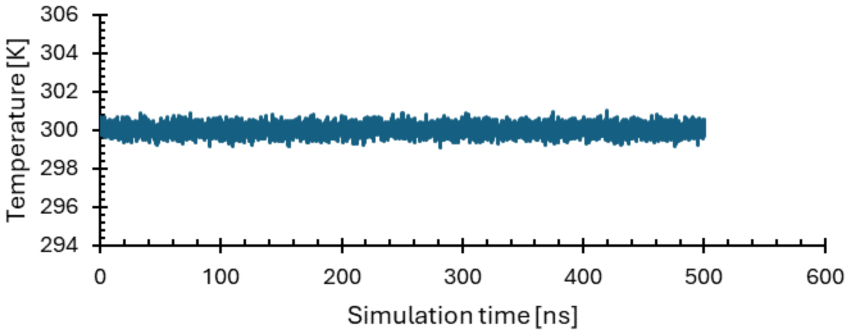

Figure 10: Temperature as a function of simulation time.

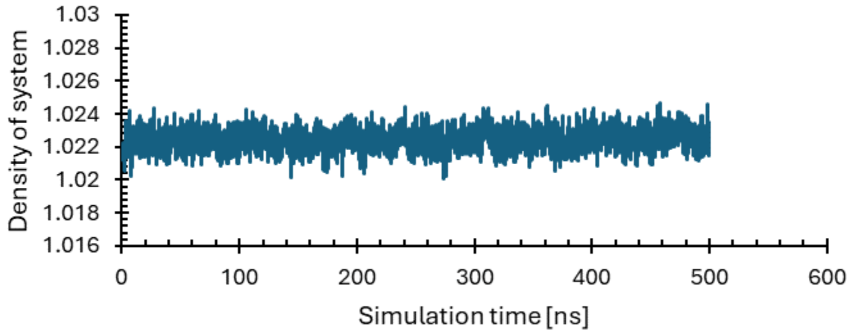

Figure 11: Density of the system as a function of simulation time.

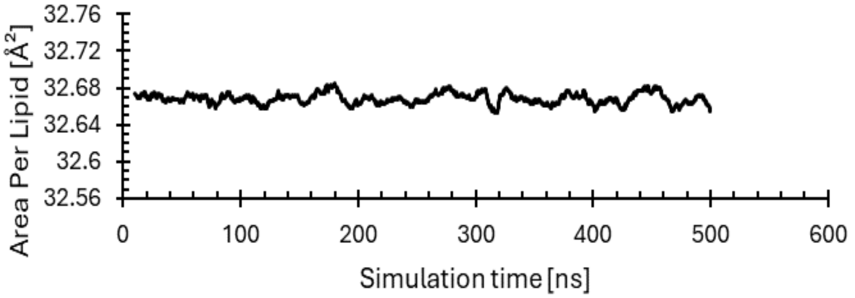

Figure 12: Area per lipid as a function of simulation time.

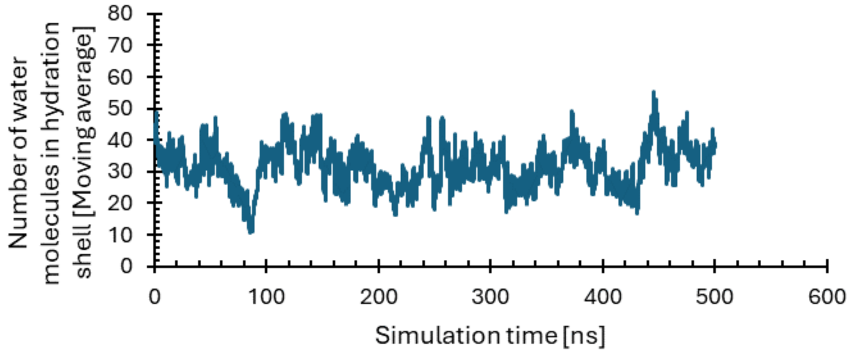

Figure 13: Total number of water molecules surrounding all viscosin alpha carbons within a 5 Å sphere as a function of simulation time

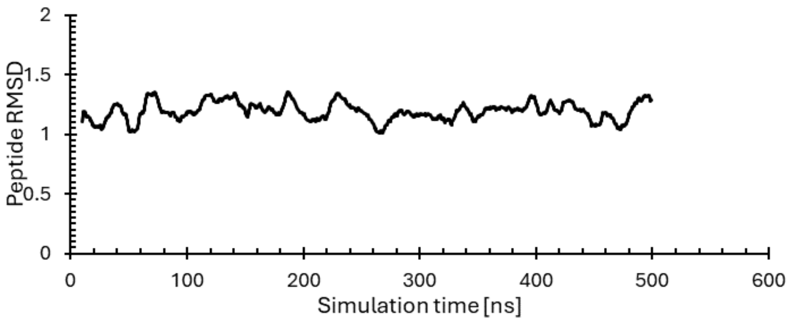

Figure 14: Heavy atom Root-Mean-Square-Deviation (RMSD) of the lipopeptide as a function of simulation time.

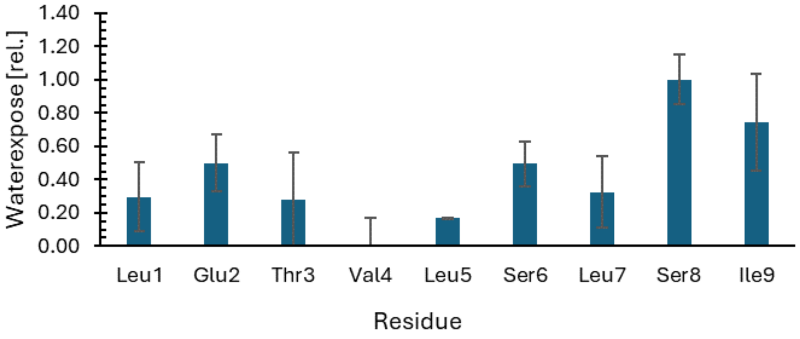

Figure 15: Normalized water exposure of the individual alpha carbons of the lipopeptide.

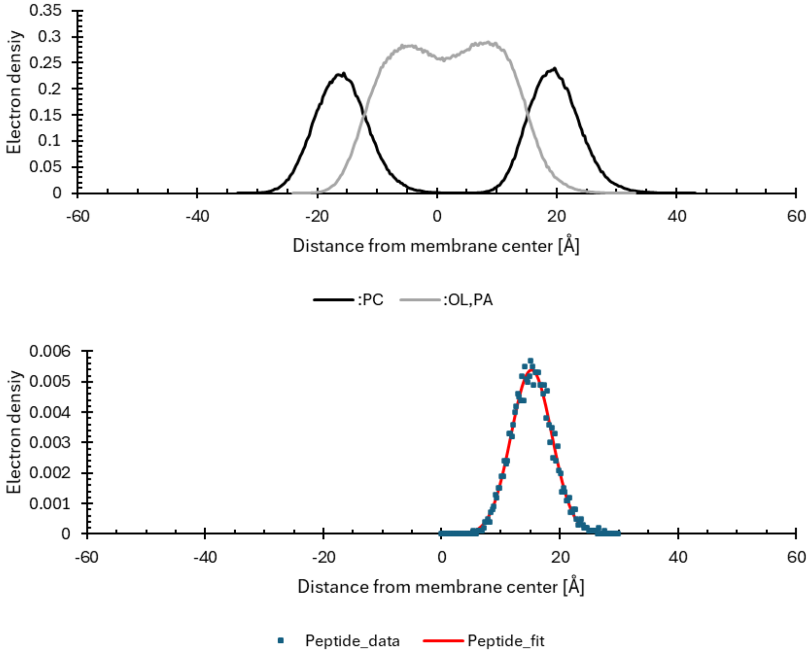

Figure 16: Electron density along the z axis of the simulation box (averaged over the final 50 ns of the simulation), as a function of distance from membrane center. Top: electron density of the phosphocholine (:PC) and oleoyl- and palmitoyl (:OL,:PA) aliphatic chains of the membrane bilayer. Bottom: Electron density of the peptide (datapoints) and it's fitted Gaussian function (red curve).

13. Impact of the starting location and orientation of viscosin within the bilayer

All simulations in this section were performed using the same protocol, the only difference being the initial location (3.1-3.3) and orientation (3.4) with respect to the bilayer. These allow to assess the time required for viscosin to equilibrate towards a final position and orientation within the membrane, and provides further support for limiting MD simulations of W-viscosins to 100 ns timescales, focusing on the final 50 ns for trajectory analysis. Given the number of simulations performed, and the outcome, 50 ns appeared sufficient to provide the required input for addressing simulation concerns with respect to the location of the lipopeptide. For reorientation concerns, we used a 150 ns simulation time was used. We show that the impact of the initial state, kinetic trapping of structures and reorientational time scales of viscosins within the solvated and pre-equilibrated POPC bilayer should be no cause for concern when 100 ns simulation times, such as used in the W-viscosin simulations, are applied.

50 ns MD simulation of viscosin initially at bilayer-water interface (phosphatidylcholine head group)

In the first of three simulations, the lipopeptide is once again positioned at the interface between the aqueous and membrane phases, i.e. with the molecular center positioned at the level of the phosphatidylcholine head group. This parallels the initial orientation of the W-derivatives in the manuscript. Results closely resemble those observed previously in 2.2. Figures 17-19 demonstrate the stability of this simulation over time. We tracked total energy, temperature, and density to ensure consistent behavior. The "area per lipid" was again employed as a metric for assessing membrane stability and convergence, maintaining stability throughout the simulation (Figure 20). In this simulation, the peptide positions itself within the interfacial region, similar to previous simulations. The number of water molecules hydrating the peptides' alpha carbons remains consistent with previous findings (Figure 21). The RMSD of the lipopeptide also remains consistent throughout the simulation (Figure 22). Additionally, quantitative analysis of hydration around each residue's alpha carbon once again confirms that viscosin is bound to the membrane bilayer surface, with Val4 most shielded from the aqueous phase (Figure 23). Lastly, electron density analysis establishes the peptide's distance from the membrane center at 13.5 ± 3.6 Å (Figure 24). Figure 25 provides a visual representation of viscosin's location at the beginning and end of the MD simulation.

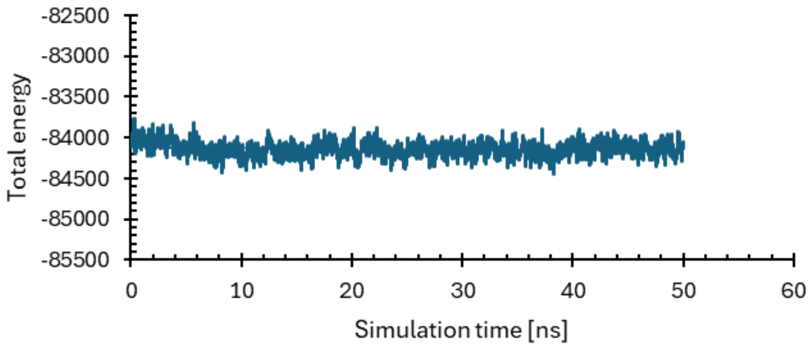

Figure 17: Total energy of the simulation, as a function of simulation time.

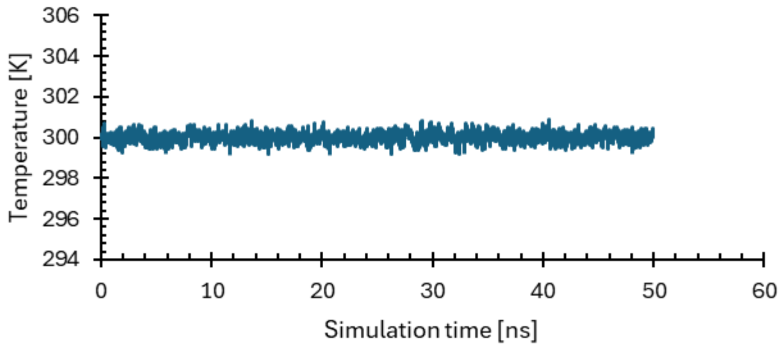

Figure 18: Temperature as a function of simulation time.

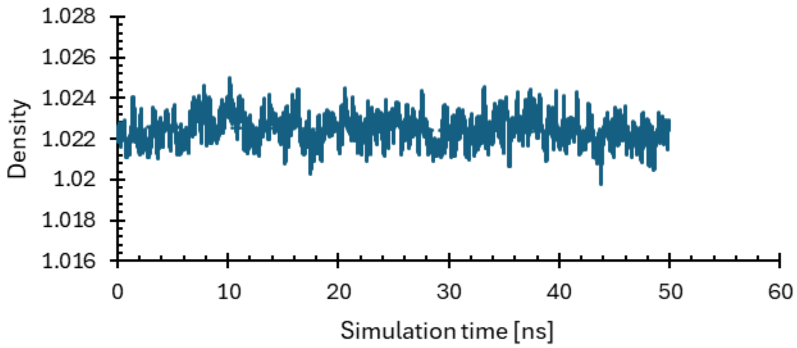

Figure 19: Density of the system as a function of simulation time.

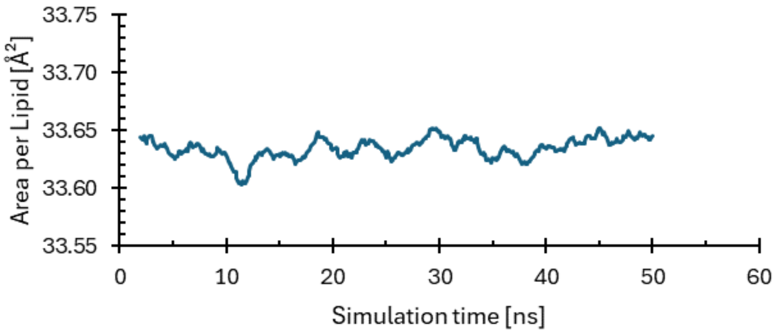

Figure 20: Area per lipid as a function of simulation time.

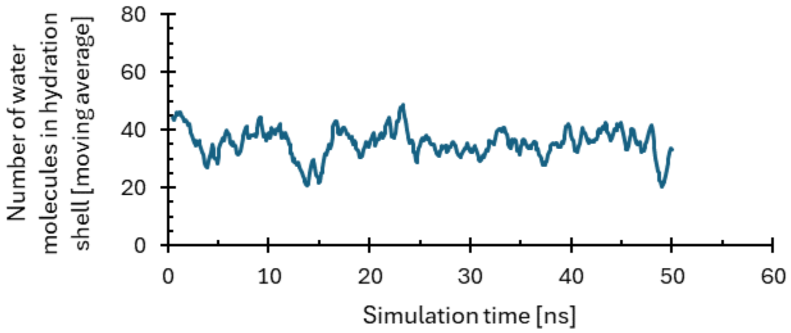

Figure 21: Total number of water molecules surrounding all viscosin alpha carbons within a 5 Å sphere as a function of simulation time.

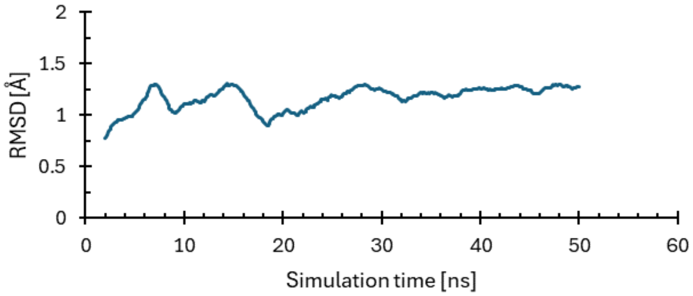

Figure 22: Heavy atom Root-Mean-Square-Deviation (RMSD) of the lipopeptide as a function of simulation time.

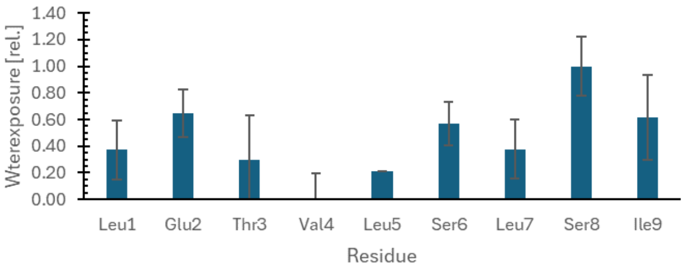

Figure 23: Total number of water molecules surrounding all viscosin alpha carbons within a 5 Å sphere as a function of simulation time.

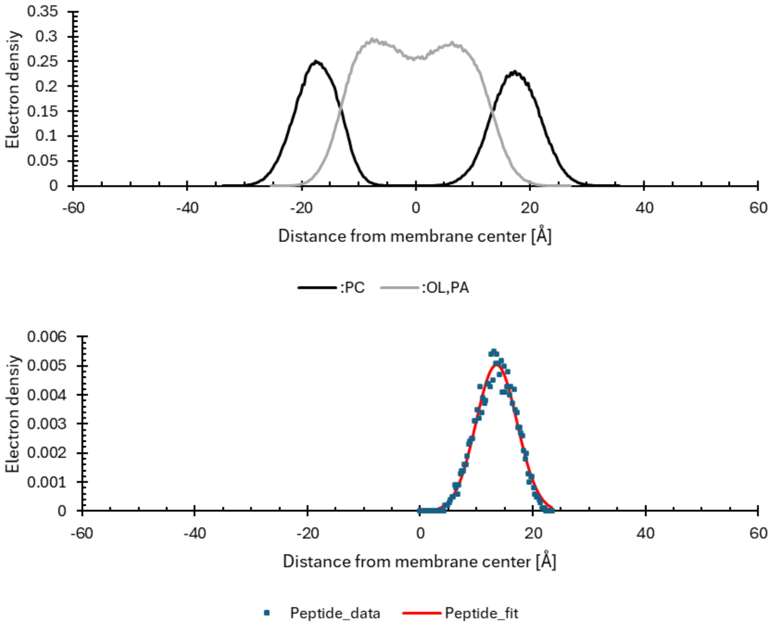

Figure 24: Electron density along the z axis of the simulation box (averaged over the final 10 ns of the simulation), as a function of distance from membrane center. Top: electron density of the phosphocholine (:PC) and oleoyl- and palmitoyl (:OL,:PA) aliphatic chains of the membrane bilayer. Bottom: Electron density of the peptide (datapoints) and it's fitted Gaussian function (red curve).

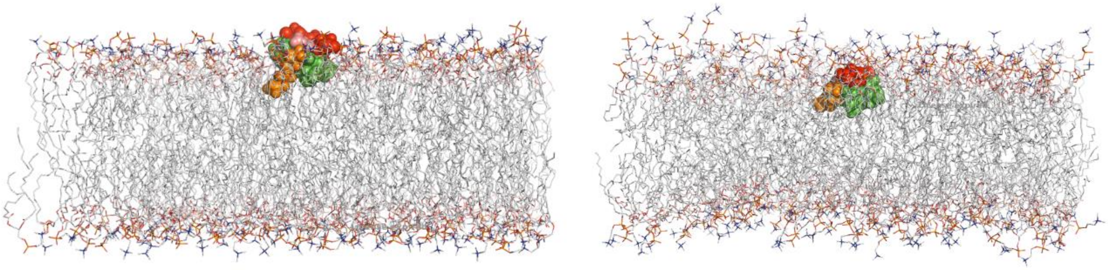

Figure 25: Snapshot of the simulation box at the start (left) and end (right) of the MD simulation.

50 ns MD simulation of viscosin initially at the interfacial region (level of the glycerol backbone)

In the second of three simulations, the lipopeptide is positioned between the polar head groups and the aliphatic tails of the POPC bilayer, i.e. with its center at the level of the glycerol backbone. Figures 26-28 depict the stability of this simulation over time. We monitored total energy, temperature, and density to ensure consistent behavior. Once again, the "area per lipid" served as a metric for evaluating membrane stability and convergence, maintaining stability throughout the simulation (Figure 29). In this simulation, the peptide positions itself within the interfacial region, similar to previous simulations. The number of water molecules hydrating the alpha carbons remains consistent with previous findings (Figure 30). The RMSD of the lipopeptide also remains stable throughout the simulation (Figure 31). Additionally, quantitative analysis of hydration around each residue's alpha carbon once again confirms that viscosin is bound to the membrane bilayer surface, with Val4 most shielded from the aqueous phase (Figure 32). Finally, electron density analysis, as employed in the manuscript, establishes the peptide's distance from the membrane center at 15.3 ± 3.4 Å (Figure 33). Figure 34 provides a visual depiction of viscosin's location at the beginning and end of the MD simulation.

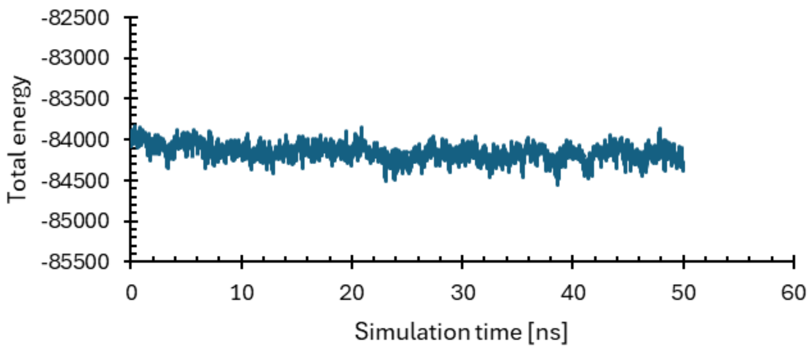

Figure 26: Total energy of the simulation, as a function of simulation time.

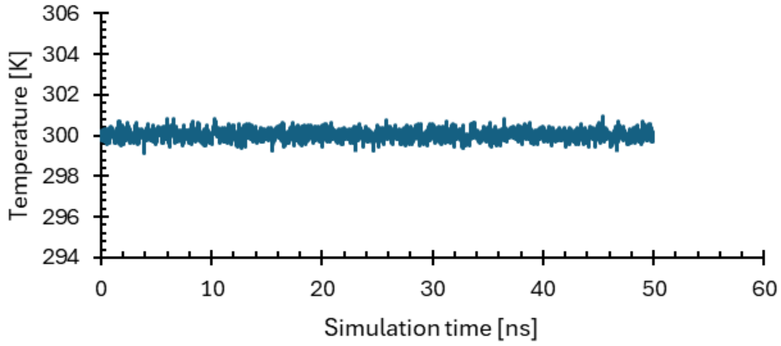

Figure 27: Temperature as a function of simulation time.

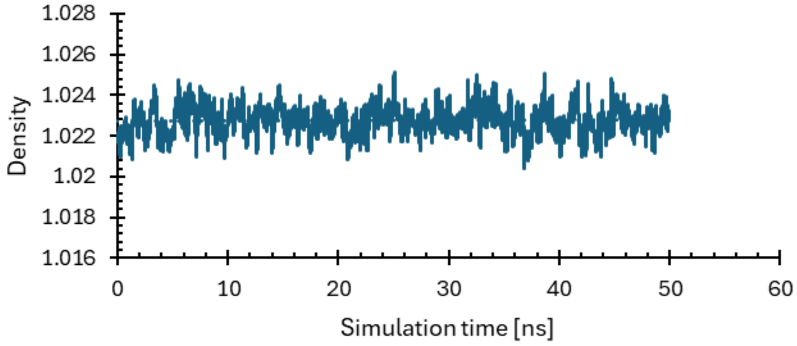

Figure 28: Density of the system as a function of simulation time.

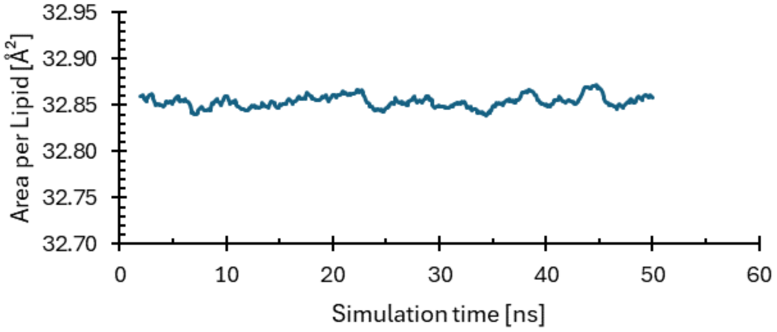

Figure 29: Area per lipid as a function of simulation time.

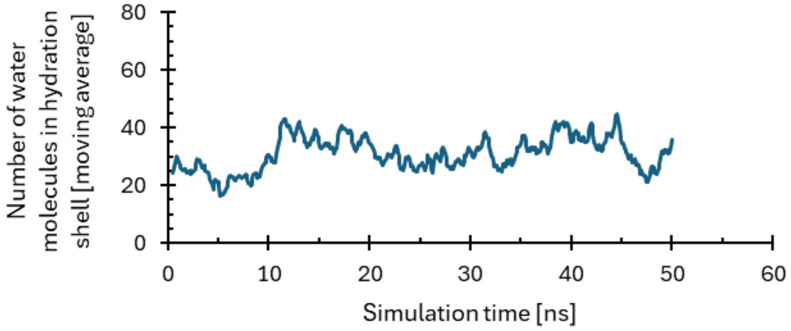

Figure 30: Total number of water molecules surrounding all viscosin alpha carbons within a 5 Å sphere as a function of simulation time.

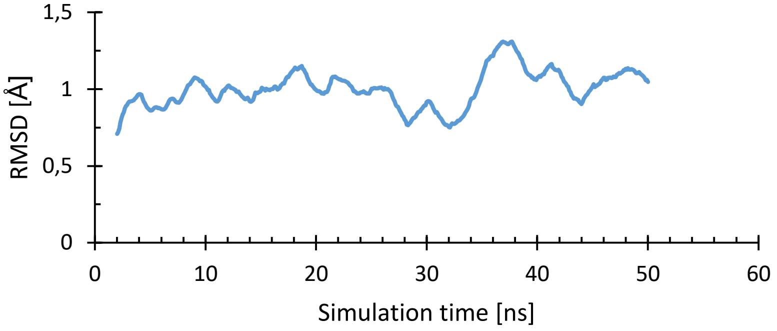

Figure 31: Heavy atom Root-Mean-Square-Deviation (RMSD) of the lipopeptide as a function of simulation time.

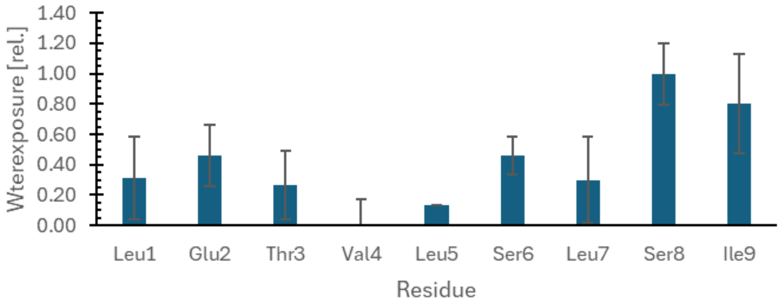

Figure 32: Normalized water exposure of the individual alpha carbons of the lipopeptide.

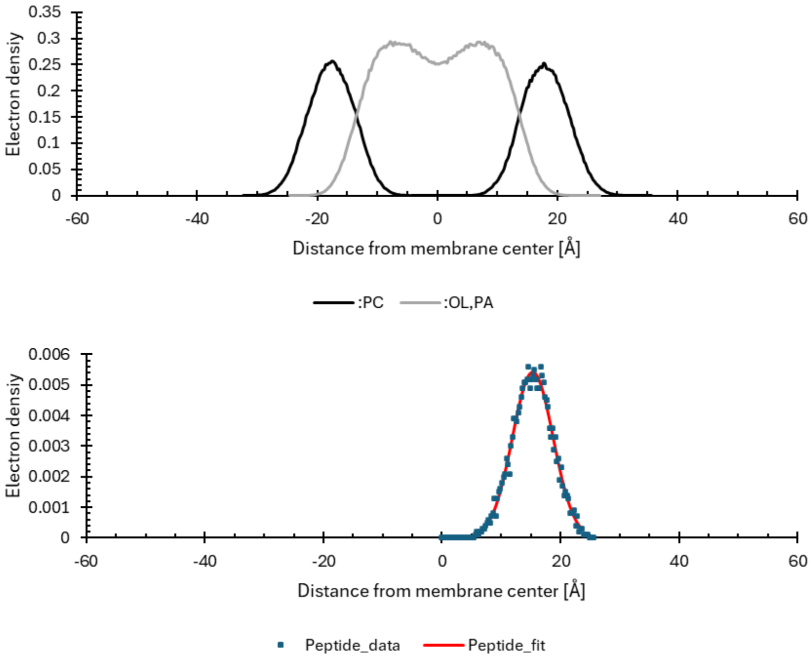

Figure 33: Electron density along the z axis of the simulation box (averaged over the final 10 ns of the simulation), as a function of distance from membrane center. Top: electron density of the phosphocholine (:PC) and oleoyl- and palmitoyl (:OL,:PA) aliphatic chains of the membrane bilayer. Bottom: Electron density of the peptide (datapoints) and it's fitted Gaussian function (red curve).

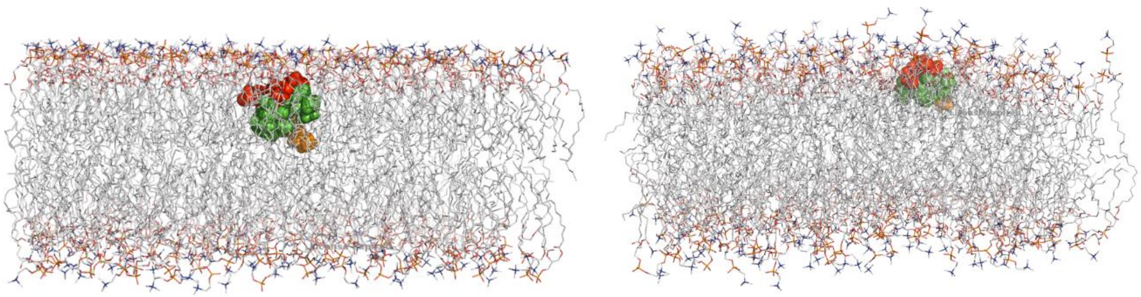

Figure 34: Snapshot of the simulation box at the start (left) and end (right) of the MD simulation.

50 ns MD simulation of viscosin initially located at the center of the bilayer

In the third simulation, the lipopeptide is placed with the molecular center in the middle of the hydrophobic core of the POPC bilayer. Figures 35-37 provide insight into the stability of this simulation over time. We closely monitored total energy, temperature, and density to ensure consistent behavior. Once again, the "area per lipid" served as a key indicator for evaluating membrane stability and convergence, maintaining stability throughout the simulation (Figure 38 In this simulation, the peptide positions itself within the interfacial region, similar to previous simulations. The number of water molecules surrounding the lipopeptide's alpha carbons remains consistent with previous observations (Figure 39). Similarly, the RMSD of the lipopeptide exhibits stability throughout the simulation (Figure 40). Additionally, quantitative analysis of hydration around each residue's alpha carbon reaffirms that viscosin is firmly located close to the membrane bilayer surface, with Val4 predominantly shielded from the aqueous phase (Figure 41). Finally, electron density analysis, as utilized in the manuscript, determines the peptide's distance from the membrane center to be at 14.2 ± 2.8 Å (Figure 42). Figure 43 visually depicts viscosin's position at the beginning and end of the MD simulation.

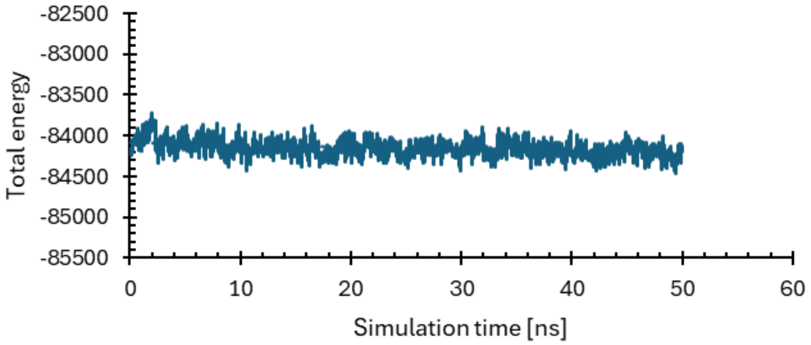

Figure 35: Total energy of the simulation, as a function of simulation time.

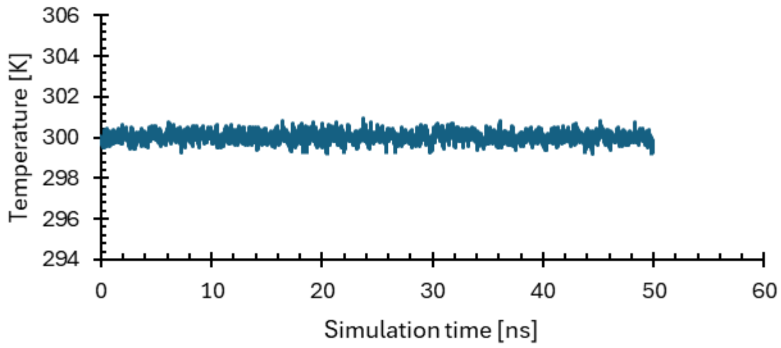

Figure 36: Temperature as a function of simulation time.

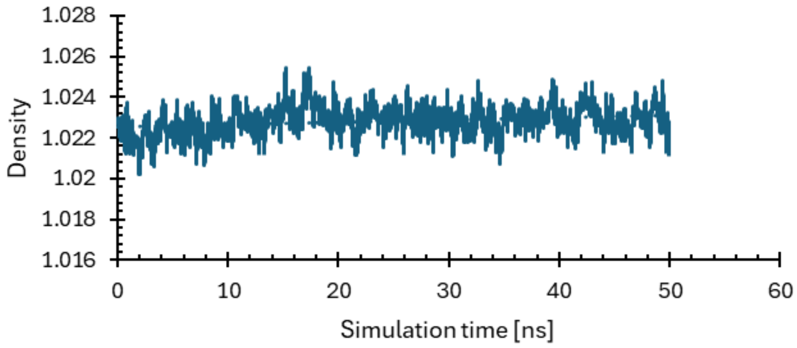

Figure 37: Density of the system as a function of simulation time.

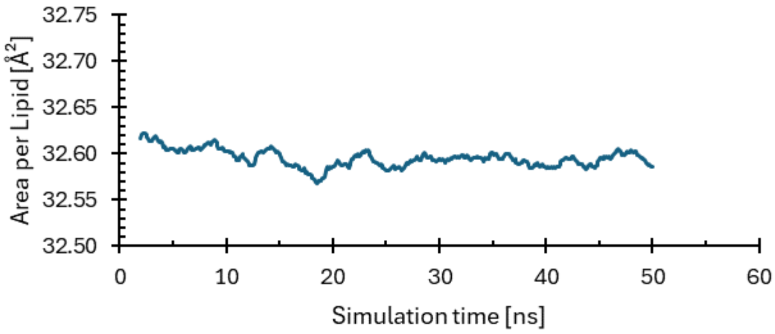

Figure 38: Area per lipid as a function of simulation time.

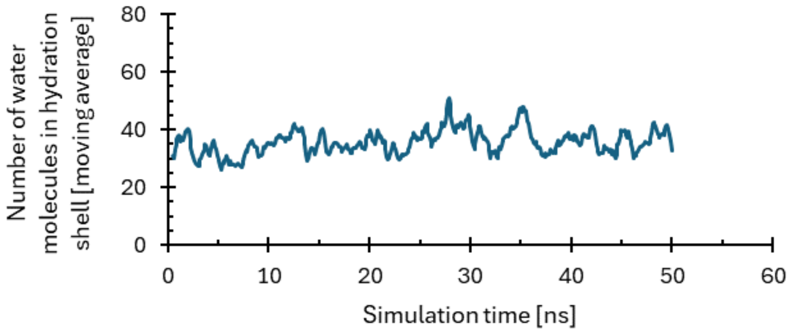

Figure 39: Total number of water molecules surrounding all viscosin alpha carbons within a 5 Å sphere as a function of simulation time.

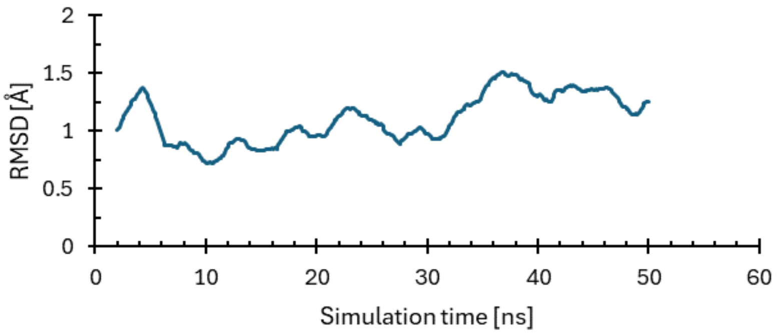

Figure 40: Heavy atom Root-Mean-Square-Deviation (RMSD) of the lipopeptide as a function of simulation time.

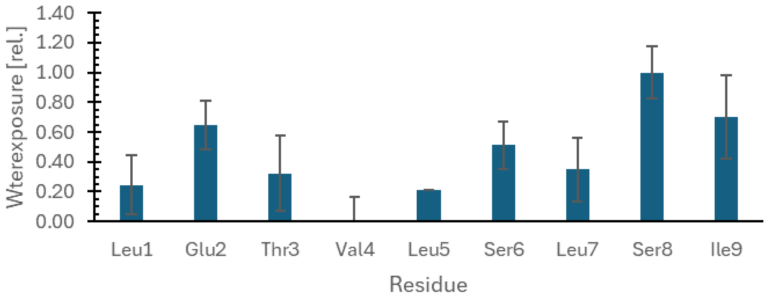

Figure 41: Normalized water exposure of the individual alpha carbons of the lipopeptide.

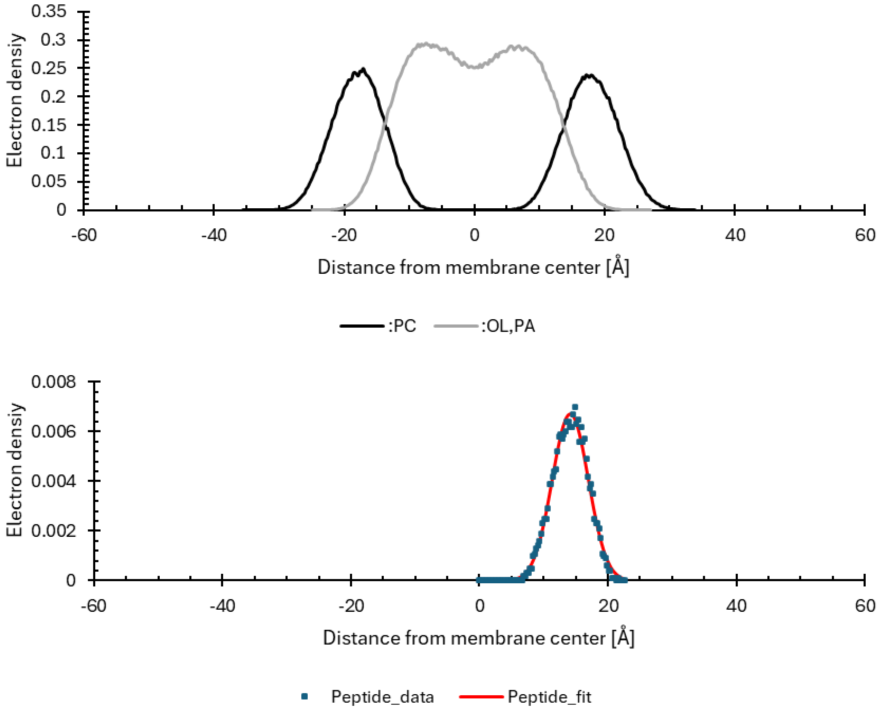

Figure 42: Electron density along the z axis of the simulation box (averaged over the final 10 ns of the simulation), as a function of distance from membrane center. Top: electron density of the phosphocholine (:PC) and oleoyl- and palmitoyl (:OL,:PA) aliphatic chains of the membrane bilayer. Bottom: Electron density of the peptide (datapoints) and it's fitted Gaussian function (red curve).

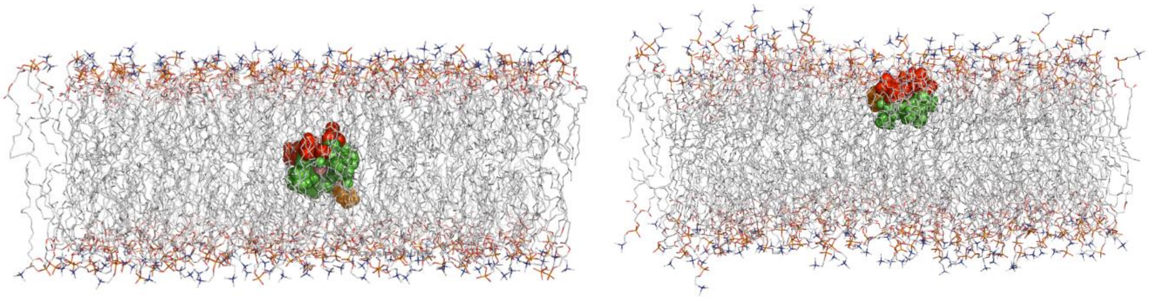

Figure 43: Snapshot of the simulation box at the start (left) and end (right) of the MD simulation.

150 ns MD simulation of viscosin oriented upside down at the bilayer-water interface.

In this simulation, the lipopeptide is again positioned between the polar head groups and the aliphatic tails of the POPC bilayer i.e. with its center at the level of the glycerol backbone (similar as simulation 3.2). Additionally, the lipopeptide is rotated 180°, such that the hydrophilic face of the lipopeptide is now facing the hydrophobic center of the membrane bilayer. As it can be expected reorientation to the normal amphipathic interaction may require more time, the simulation was performed over 150 ns. Figures 44-46 provide insight into the stability of this simulation over time. We closely monitored total energy, temperature, and density to ensure consistent behavior. Once again, the "area per lipid" served as a key indicator for evaluating membrane stability and convergence, maintaining stability throughout the simulation (Figure 47). In this simulation, the peptide positions itself within the interfacial region, similar to previous simulations. The number of water molecules surrounding the lipopeptide's alpha carbons remains consistent with previous observations (Figure 48). Similarly, the RMSD of the lipopeptide exhibits stability throughout the simulation (Figure 49). Additionally, quantitative analysis of hydration around each residue's alpha carbon reaffirms that viscosin is firmly positioned at the membrane bilayer surface, with Val4 predominantly shielded from the aqueous phase (Figure 50). Consequently, starting with the lipopeptide rotated 180°, reorientation is complete after 80 ns. The final orientation matches very well with those observed before. Finally, electron density analysis, as utilized in the manuscript, determines the peptide's distance from the membrane center to be at 16.4 ± 3.4 Å (Figure 51). Figure 52 visually depicts viscosin's position at the beginning and end of the MD simulation.

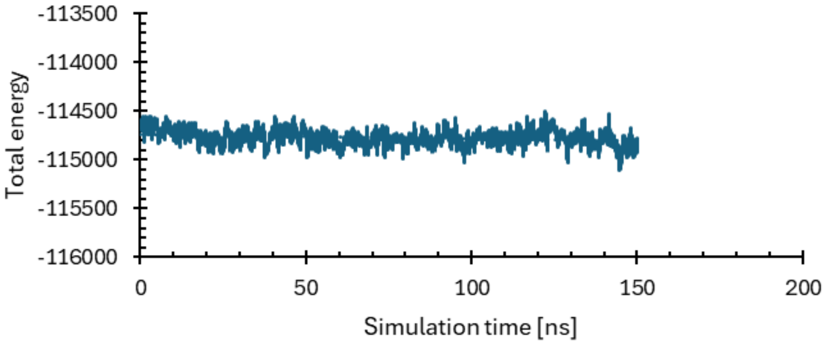

Figure 44: Total energy of the simulation, as a function of simulation time.

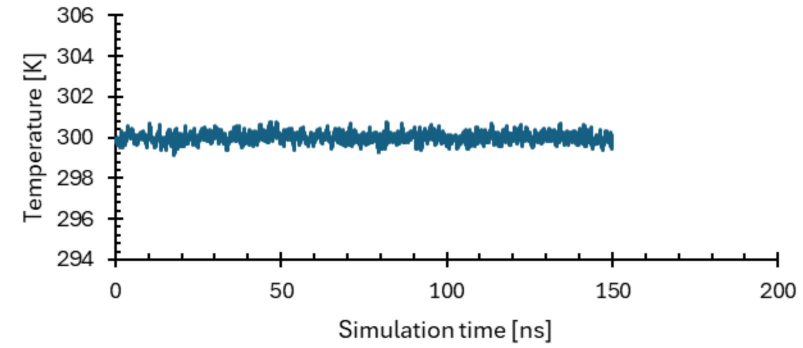

Figure 45: Temperature as a function of simulation time.

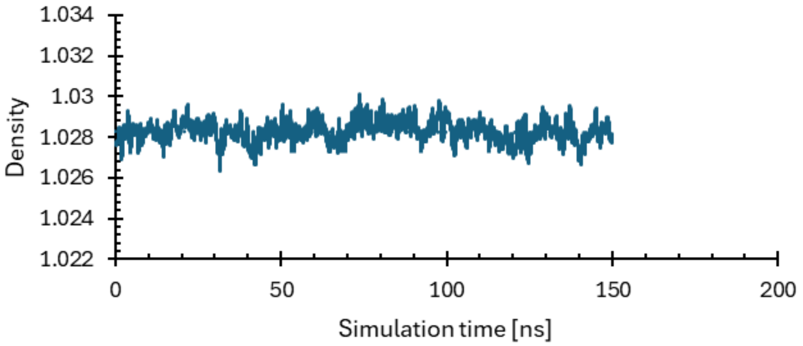

Figure 46: Density of the system as a function of simulation time.

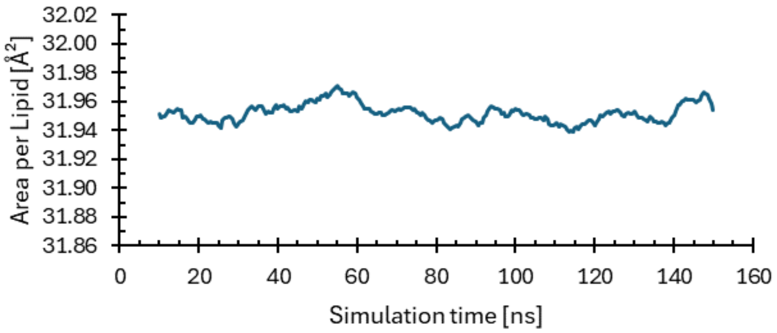

Figure 47: Area per lipid as a function of simulation time.

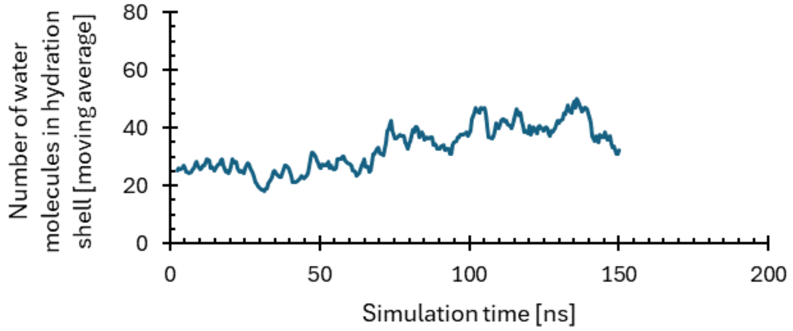

Figure 48: Total number of water molecules surrounding all viscosin alpha carbons within a 5 Å sphere as a function of simulation time.

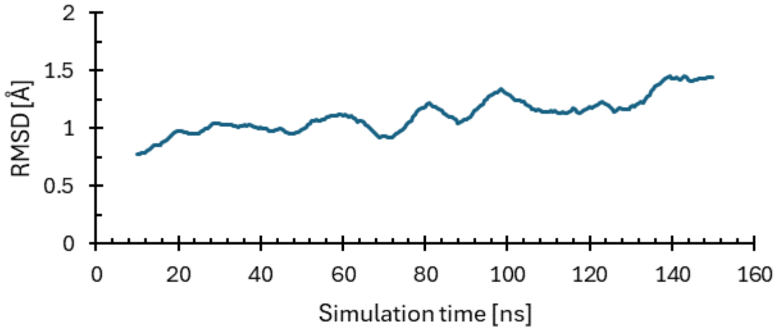

Figure 49: Heavy atom Root-Mean-Square-Deviation (RMSD) of the lipopeptide as a function of simulation time.

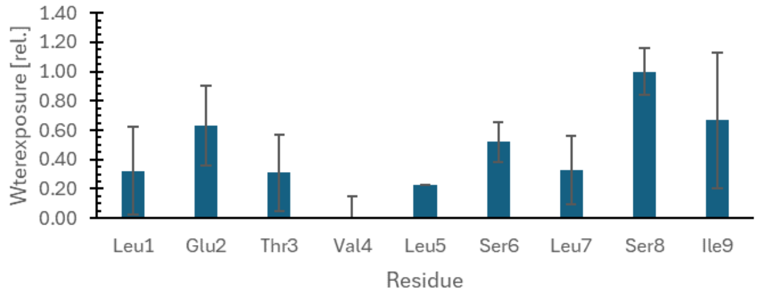

Figure 50: Normalized water exposure of the individual alpha carbons of the lipopeptide.

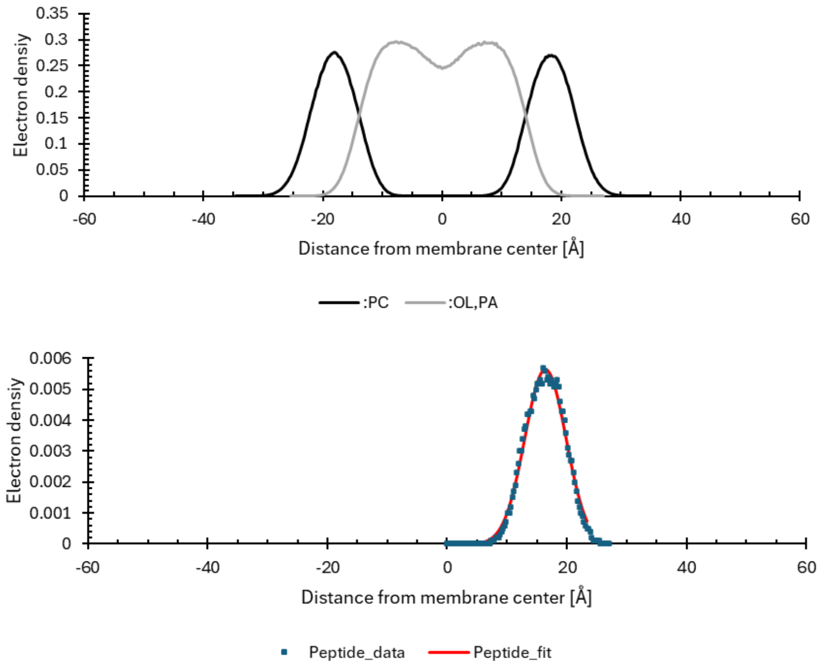

Figure 51: Electron density along the z axis of the simulation box (averaged over the final 50 ns of the simulation), as a function of distance from membrane center. Top: electron density of the phosphocholine (:PC) and oleoyl- and palmitoyl (:OL,:PA) aliphatic chains of the membrane bilayer. Bottom: Electron density of the peptide (datapoints) and it's fitted Gaussian function (red curve).

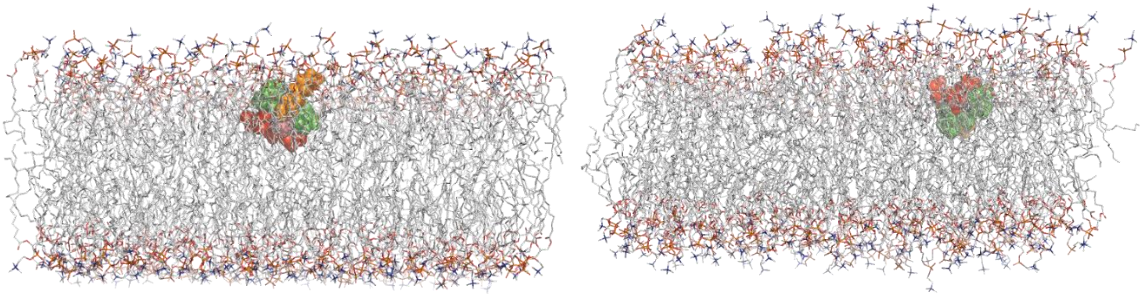

Figure 52: Snapshot of the simulation box at the start (left) and end (right) of the MD simulation.

14. MD simulations of the tryptophan-labelled lipopeptides

Lastly, we offer supplementary data regarding the MD simulations of the tryptophan-labeled lipopeptides, as included in the manuscript. The coordinates for all starting structures (including that of wild-type viscosin) and associated simulation input files are available from a GitHub repository (https://github.com/nmrstruc/BiophysJ-W-viscosins).

MD simulation of L1W

We closely monitored the total energy, temperature, and density of the MD simulations for the L1W-labelled viscosin system to ensure consistent behavior (Figures 53-55). The "area per lipid" served as a metric for evaluating membrane stability and convergence, maintaining stability throughout the simulation (Figure 56). In this simulation, the peptide positions itself within the interfacial region (Figure 57), similar to previous simulations. The number of water molecules surrounding the alpha carbons remained consistent with previous findings (Figure 58). Similarly, the RMSD of the lipopeptide exhibited stability throughout the simulation (Figure 59). Finally, quantitative analysis of hydration around each residue's alpha carbon reaffirmed that viscosin remained surface-bound on the membrane bilayer, with Val4 most shielded from the aqueous phase (Figure 60).

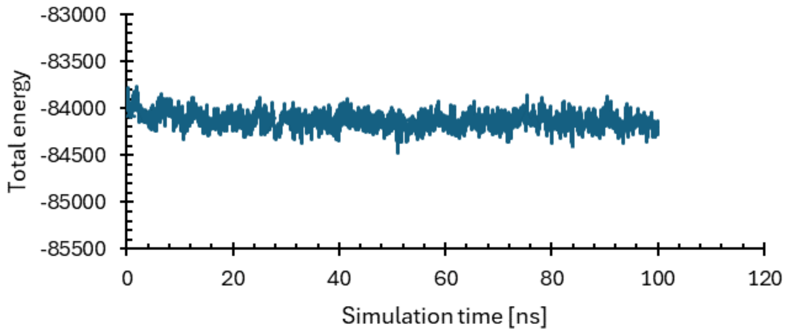

Figure 53: Total energy of the simulation, as a function of simulation time.

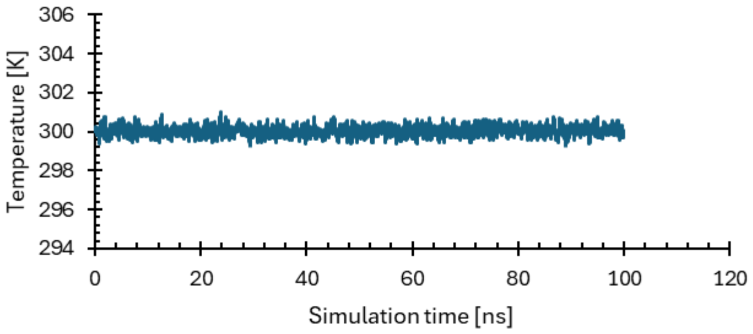

Figure 54: Temperature as a function of simulation time.

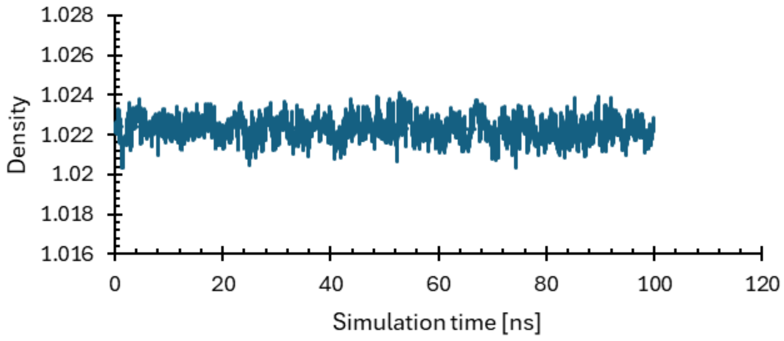

Figure 55: Density of the system as a function of simulation time.

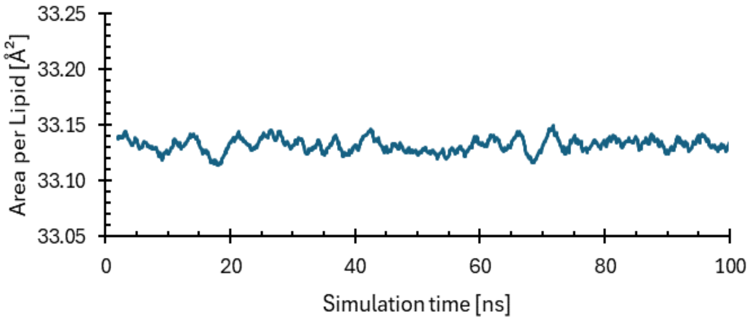

Figure 56: Area per lipid as a function of simulation time.

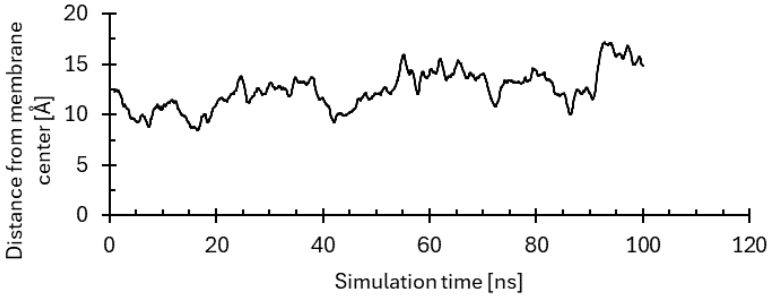

Figure 57: Distance of lipopeptide center of mass from the membrane center, as a function of simulation time.

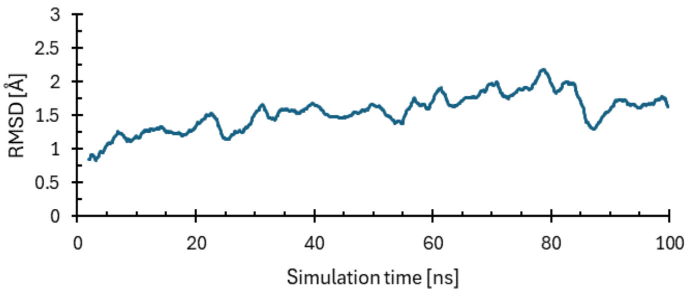

Figure 58: Heavy atom Root-Mean-Square-Deviation (RMSD) of the lipopeptide as a function of simulation time.

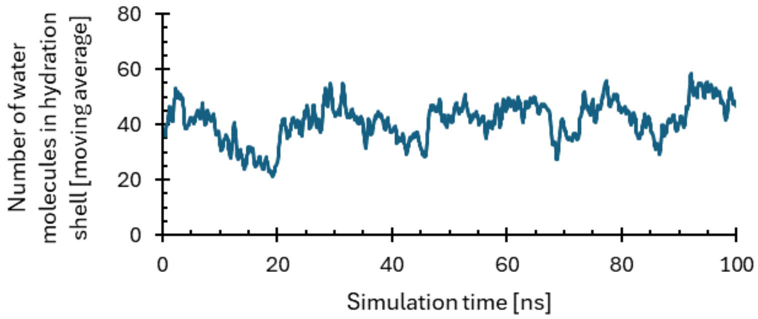

Figure 59: Total number of water molecules surrounding all viscosin alpha carbons within a 5 Å sphere as a function of simulation time.

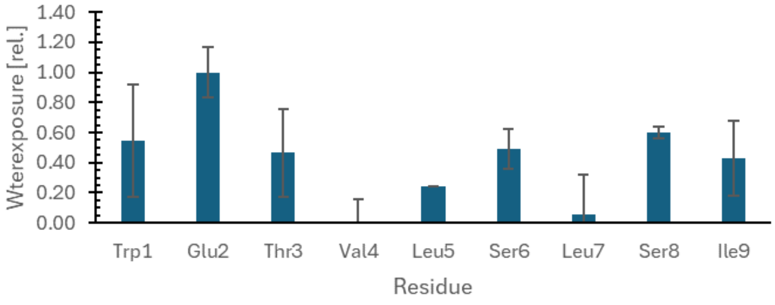

Figure 60: Normalized water exposure of the individual alpha carbons of the lipopeptide.

MD simulation of V_4W

We monitored total energy, temperature, and density of the MD simulations of the V4W-labelled viscosin system to ensure consistent behavior (Figures 61-63). The 'area per lipid' is used as a metric for assessing membrane stability and convergence, remaining stable throughout the simulation (Figure 64). In this simulation, the peptide positions itself within the interfacial region (*Figure 65*), similar to previous simulations. The number of water molecules hydrating the alpha carbons is similar to before. (Figure 67). The RMSD of the lipopeptide also remains stable throughout the simulation (Figure 66). Finally, quantitative analysis of hydration around each residues' alpha carbon again indicates that viscosin is surface-bound on the membrane bilayer, with the fourth residue (now Trp4) largely shielded from the aqueous phase (Figure 68).

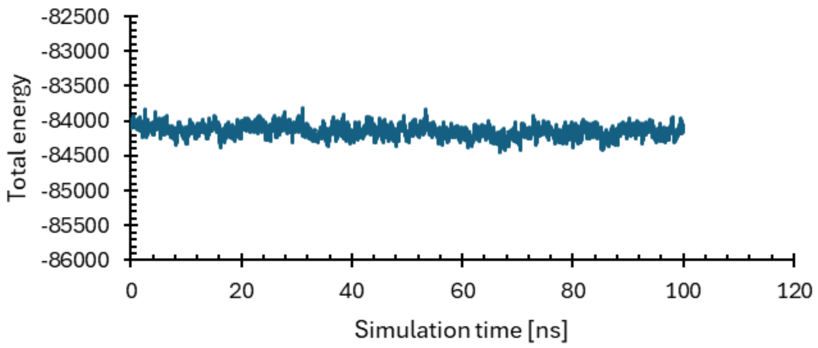

Figure 61: Total energy of the simulation, as a function of simulation time.

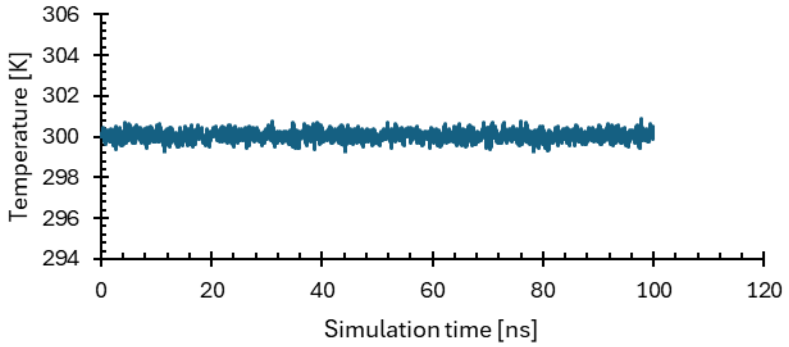

Figure 62: Temperature as a function of simulation time.

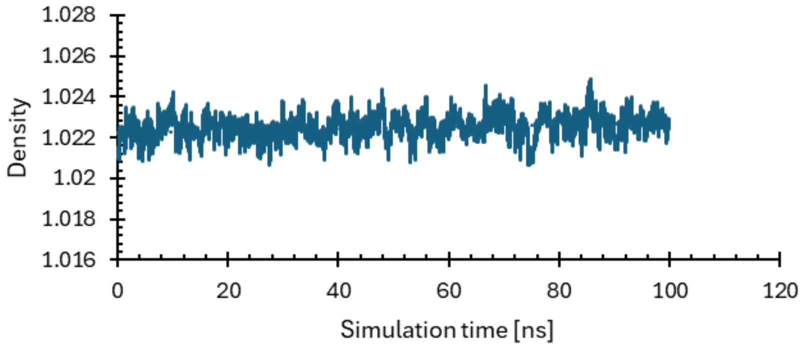

Figure 63: Density of the system as a function of simulation time.

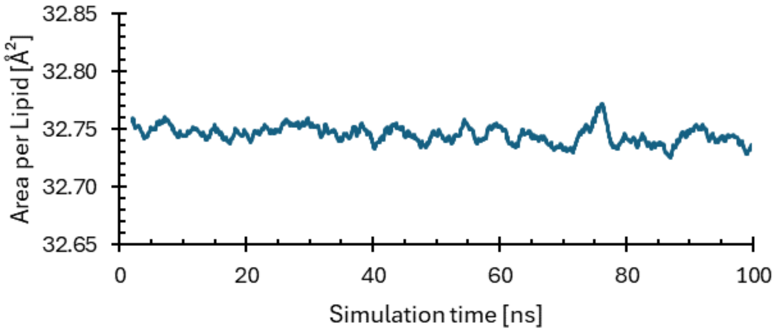

Figure 64: Area per lipid as a function of simulation time.

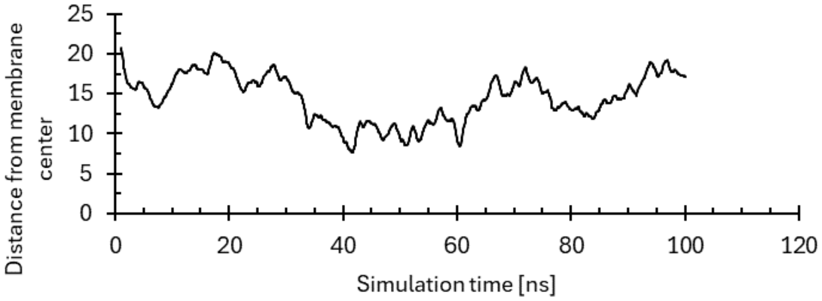

Figure 65: Distance of lipopeptide center of mass from the membrane center, as a function of simulation time.

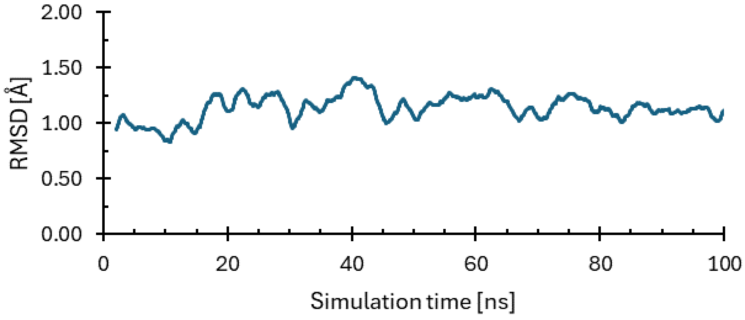

Figure 66: Heavy atom Root-Mean-Square-Deviation (RMSD) of the lipopeptide as a function of simulation time.

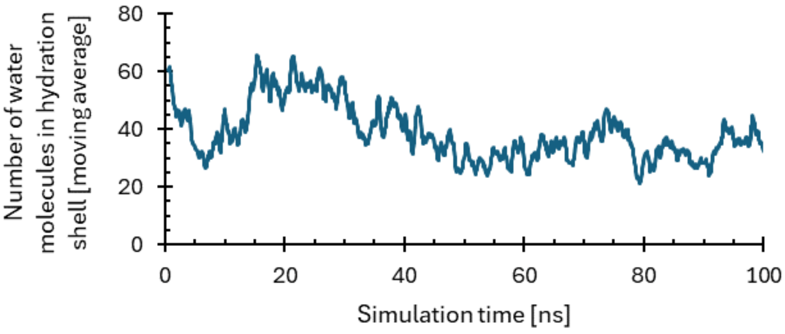

Figure 67: Total number of water molecules surrounding all viscosin alpha carbons within a 5 Å sphere as a function of simulation time.

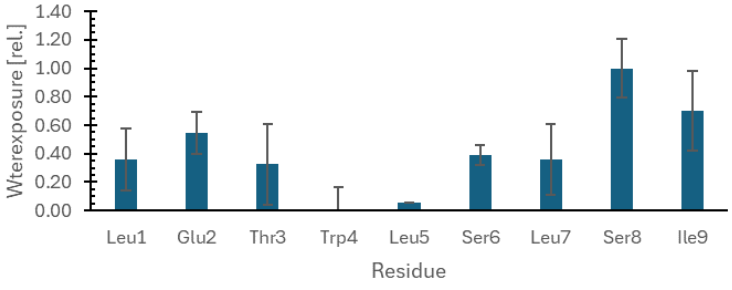

Figure 68: Normalized water exposure of the individual alpha carbons of the lipopeptide, the original peptide sequence is indicated.

MD simulation of L5W

We monitored total energy, temperature, and density of the MD simulations of the L5W-labelled viscosin system to ensure consistent behavior (Figures 69-71). The 'area per lipid' is used as a metric for assessing membrane stability and convergence, remaining stable throughout the simulation (Figure 72). In this simulation, the peptide positions itself within the interfacial region (*Figure 73*), similar to previous simulations. The number of water molecules hydrating the alpha carbons is similar to before. (Figure 74). The RMSD of the lipopeptide also remains stable throughout the simulation (Figure 75). Finally, quantitative analysis of hydration around each residues' alpha carbon again indicates that viscosin is surface-bound on the membrane bilayer, with Val4 largely shielded from the aqueous phase (Figure 76).

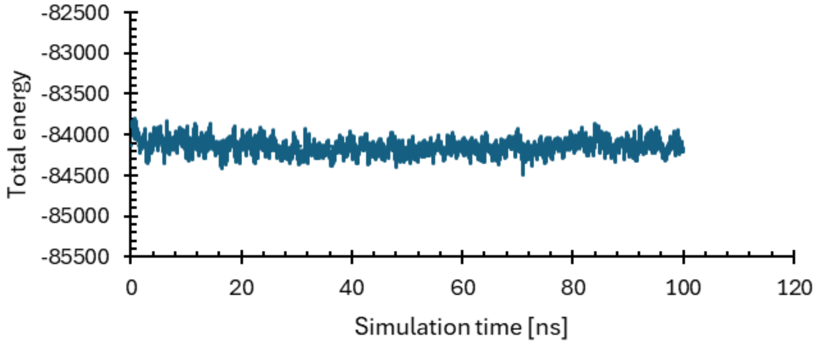

Figure 69: Total energy of the simulation, as a function of simulation time.

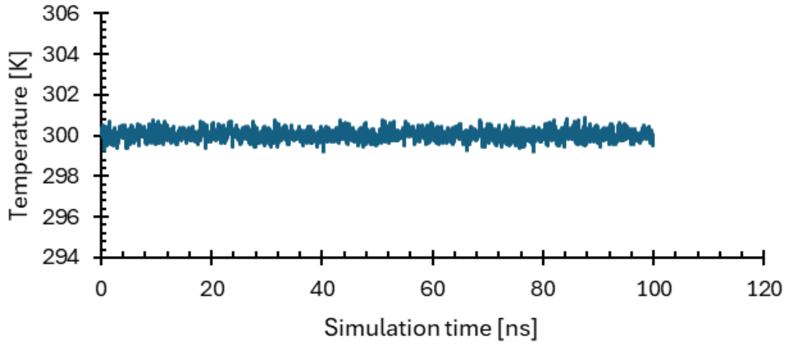

Figure 70: Temperature as a function of simulation time.

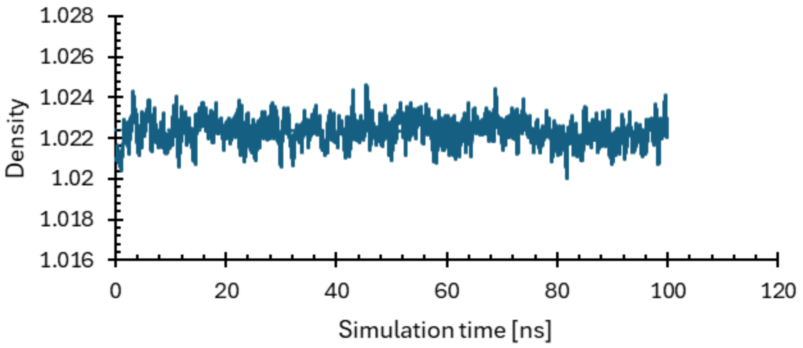

Figure 71: Density of the system as a function of simulation time.

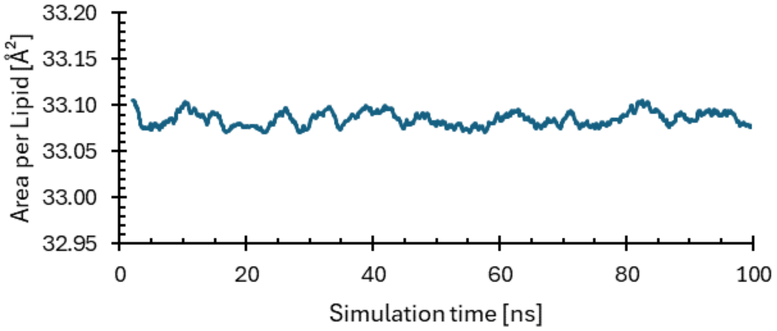

Figure 72: Area per lipid as a function of simulation time.

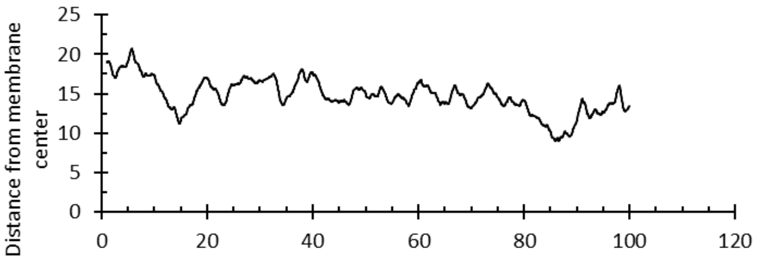

Figure 73: Distance of lipopeptide center of mass from the membrane center, as a function of simulation time.

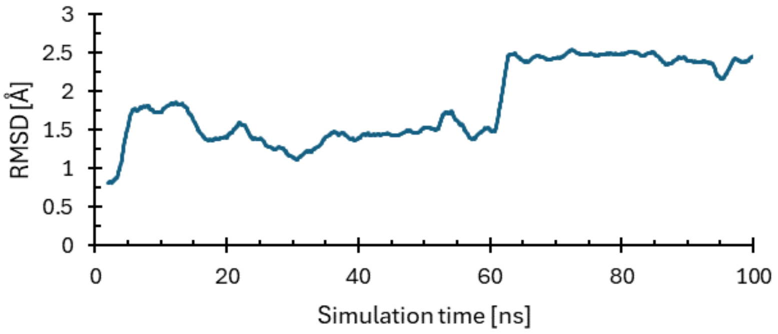

Figure 74: Heavy atom Root-Mean-Square-Deviation (RMSD) of the lipopeptide as a function of simulation time.

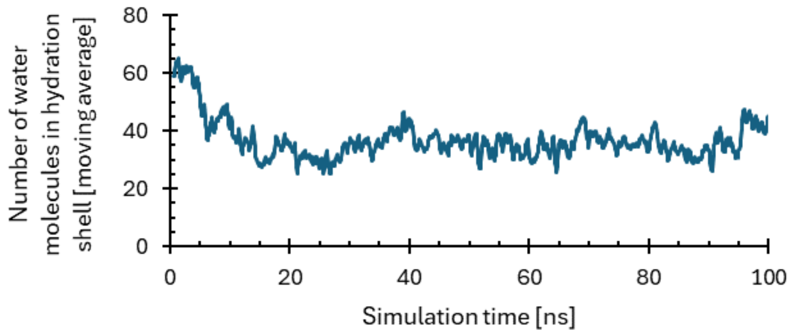

Figure 75: Total number of water molecules surrounding all viscosin alpha carbons within a 5 Å sphere as a function of simulation time.

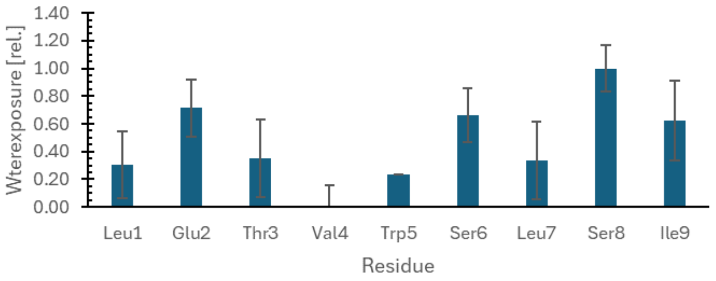

Figure 76: Normalized water exposure of the individual alpha carbons of the lipopeptide.

MD simulation of L7W

We monitored total energy, temperature, and density of the MD simulatins of the L7W-labelled viscosin system to ensure consistent behavior (Figures 77-79). The 'area per lipid' is used as a metric for assessing membrane stability and convergence, remaining stable throughout the simulation (Figure 80). In this simulation, the peptide positions itself within the interfacial region (*Figure 81*), similar to previous simulations. The number of water molecules hydrating the alpha carbons is similar to before. (Figure 82). The RMSD of the lipopeptide also remains stable throughout the simulation (Figure 83). Finally, quantitative analysis of hydration around each residues' alpha carbon again indicates that viscosin is surface-bound on the membrane bilayer, with Val4 largely shielded from the aqueous phase (Figure 84).

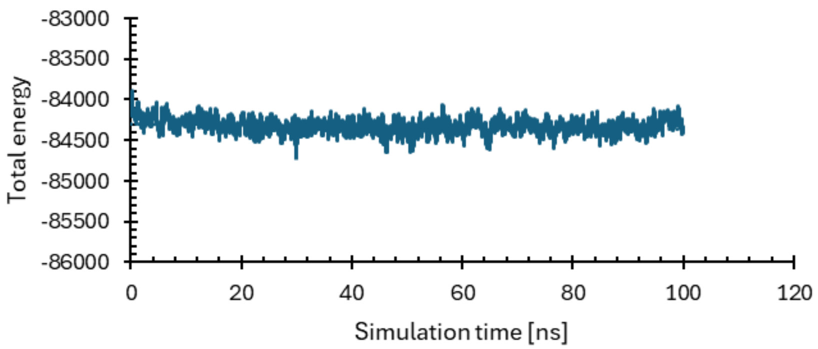

Figure 77: Total energy of the simulation, as a function of simulation time.

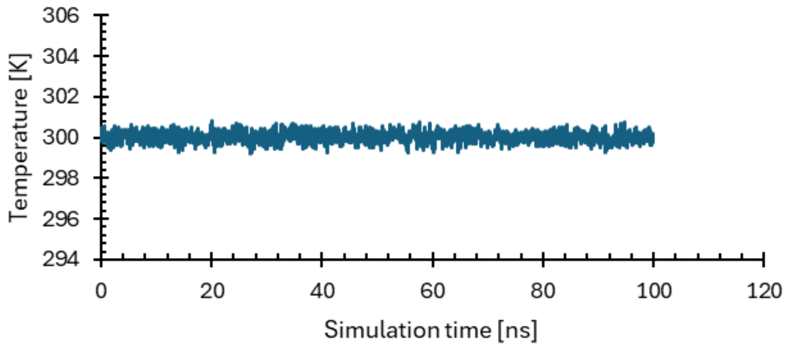

Figure 78: Temperature as a function of simulation time.

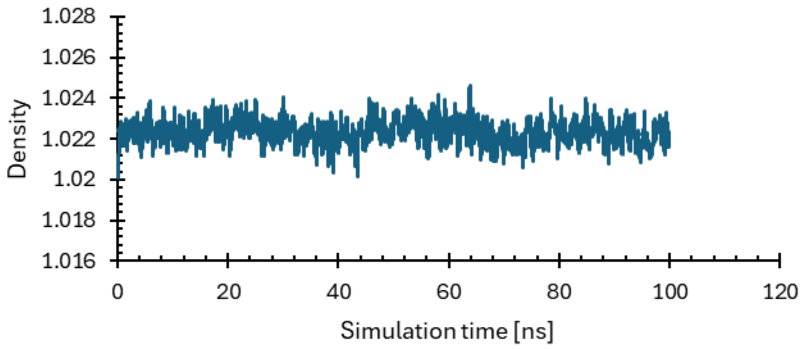

Figure 79: Density of the system as a function of simulation time.

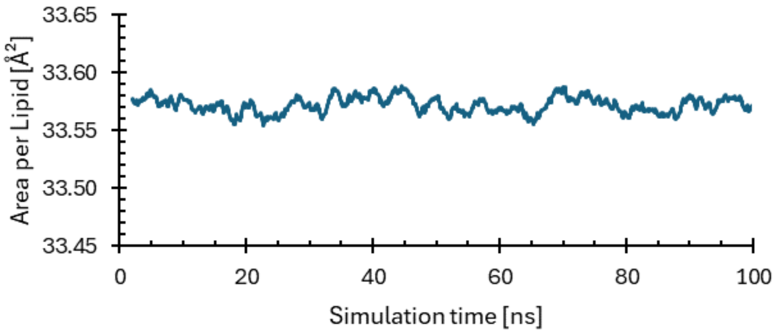

Figure 80: Area per lipid as a function of simulation time.

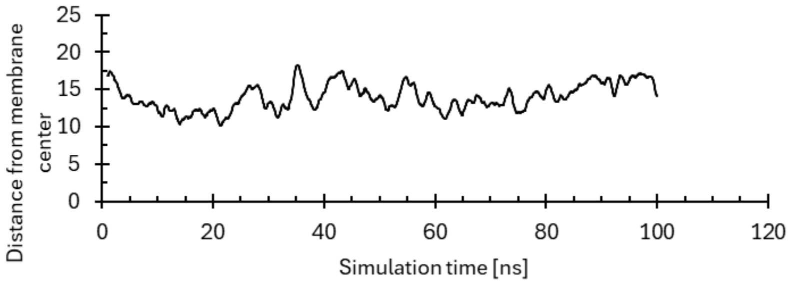

Figure 81: Distance of lipopeptide center of mass from the membrane center, as a function of simulation time.

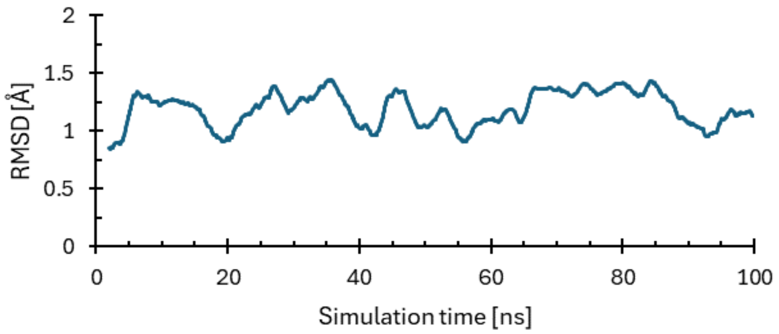

Figure 82: Heavy atom Root-Mean-Square-Deviation (RMSD) of the lipopeptide as a function of simulation time.

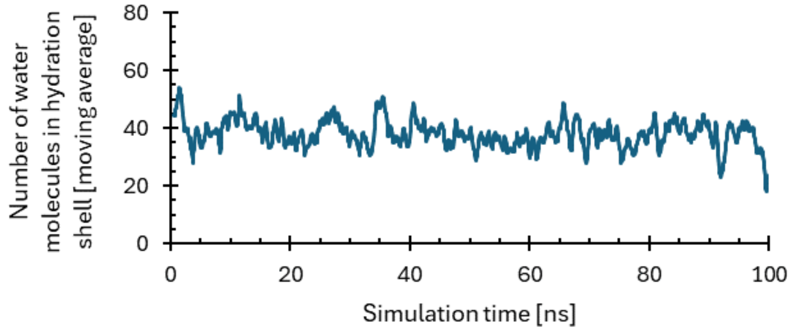

Figure 83: Total number of water molecules surrounding all viscosin alpha carbons within a 5 Å sphere as a function of simulation time.

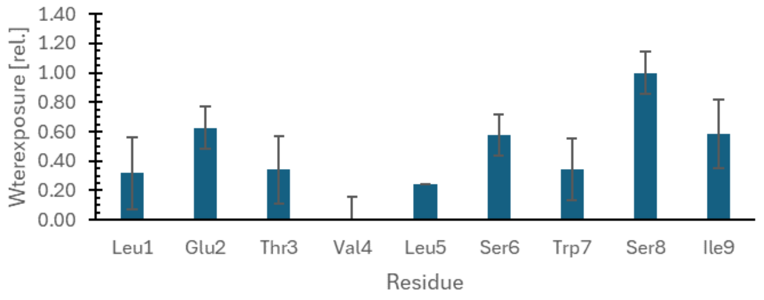

Figure 84: Normalized water exposure of the individual alpha carbons of the lipopeptide.

15. References

(10) N. Geudens, B. Kovacs, D. Sinnaeve, F. E. Oni, M. Höfte, J. C. Martins, *Molecules* **2019**, *24*.
